# 1-Hydroxyanthraquinones Containing Aryl Substituents as Potent and Selective Anticancer Agents

**DOI:** 10.3390/molecules25112547

**Published:** 2020-05-29

**Authors:** Nafisa S. Sirazhetdinova, Victor A. Savelyev, Tatyana S. Frolova, Dmitry S. Baev, Lyubov S. Klimenko, Ivan V. Chernikov, Olga S. Oleshko, Teresa A. Sarojan, Andrey G. Pokrovskii, Elvira E. Shults

**Affiliations:** 1 Laboratory of Medicinal Chemistry, N.N. Vorozhtsov Novosibirsk Institute of Organic Chemistry, Siberian Branch of the Russian Academy of Sciences, Lavrentyev Ave, 9, 630090 Novosibirsk, Russia; snafisas@mail.ru (N.S.S.); vicsav@nioch.nsc.ru (V.A.S.); baev@nioch.nsc.ru (D.S.B.); 2 The Federal Research Center Institute of Cytology and Genetics, Acad. Lavrentyev Ave., 10, 630090 Novosibirsk, Russia; frolova@bionet.nsc.ru; 3 Novosibirsk State University, Pirogova Str. 1, 630090 Novosibirsk, Russia; oleshko_os@mail.ru (O.S.O.); 111.st.13@rambler.ru (T.A.S.); agpok@inbox.ru (A.G.P.); 4 Yugra State University, 628012 Khanty-Mansiysk, Russia; l_klimenko@ugrasu.ru; 5 Institute of Chemical Biology and Fundamental Medicine Siberian Branch of the Russian Academy of Sciences, Lavrentyev Ave, 9, 630090 Novosibirsk, Russia; chernikovivanv@gmail.com

**Keywords:** anthraquinones, Suzuki cross-coupling reaction, cytotoxicity, DNA binding

## Abstract

A series of 1,2-, 1,4-disubstituted or 1,2,4-trisubstituted anthraquinone-based compounds was designed, synthesized, characterized and biologically evaluated for anticancer efficacy. 2- or 4-arylated 1-hydroxy-9,10-antraquinones (anthracene-9,10-diones) were prepared by Suzuki–Miyaura cross-coupling reaction of 1-hydroxy-2-bromoanthraquinone, 1-hydroxy-4-iodoanthraquinone or 1-hydroxy-2,4-dibromoanthraquinone with arylboronic acids. The cross-coupling reaction of 2,4-dibromo-9,10-anthraquinone with arylboronic acids provide a convenient approach to 2,4-bis arylated 1-hydroxyanthraquinones with a variety of aryl substituent in the 2 and 4 position. The cytotoxicity of new anthraquinone derivatives was evaluated using the conventional MTT assays. The data revealed that six of the aryl substituted compounds among the entire series **3**, **15**, **16**, **25**, **27**, **28** were comparable potent with the commercially available reference drug doxorubicin on the human glioblastoma cells SNB-19, prostate cancer DU-145 or breast cancer cells MDA-MB-231 and were relatively safe towards human telomerase (h-TERT)immortalized lung fibroblasts cells. The results suggested that the in vitro antitumor activity of synthesized 2-aryl, 4-aryl- and 2,4-diaryl substituted 1-hydroxyanthraquinones depends on the nature of the substituent within the cyclic backbone. Docking interaction of 2-, 4-substituted and 2,4-disubstituted 1-hydroxyanthraquinones indicates intercalative mode of binding of compounds with DNA topoisomerase. The interaction with the DNA of 4-aryl-**13**, **15**, **16** and 4-(furan-3-yl)-**23** 1-hydroxyanthraquinones was experimentally confirmed through a change in electroforetic mobility. Further experiments with 1-hydroxy-4-phenyl-anthraquinone **13** demonstrated that the compound induced cell cycle arrest at sub-G1 phase in DU-145 cells in the concentration 1.1 μM, which is probably achieved by inducing apoptosis. 4-Arylsubstituted 1-hydroxyanthraquinones **13** and **16** induced the enhancement of DNA synthesis on SNB19 cell lines.

## 1. Introduction

The anthraquinone (anthracene-9,10-dione), a polycyclic aromatic core, is an important structural motif in a large number of organic molecules, is prevalent in nature. Several functionalized anthraquinones are well known for their diverse and profound biological activities. Since the discovery of the synthetic anthraquinone antitumor drug mitoxantrone, that is clinically used for the treatment of a variety of cancers [1], various 9,10-anthraquinone derivatives have been investigated and used as antiviral [2], antibacterial [3], anticancer [4] and anti-inflammatory agents [5]. 2-Aryl and 4-aryl substituted anthraquinones were isolated from the natural sources [6]. From these compounds the natural 4-arylanthraquinones—knipholones – are of interest. Knipholone and isoknipholone have recently been reported to exhibit good antitumoral activities against several cancer cells, some of them comparable to that of etoposide [4,7]. Knipholone and its related derivatives have been reported to exhibit significant activities against the malaria parasite, *Plasmodium falciparum* [6]. Based on these grounds, the search for efficient and versatile synthetic methodologies leading to variously substituted 9,10-anthraquinones deserves great attention.

A brief literature survey revealed that the routes for the construction of anthraquinone core are primarily based upon five categories, such as Friedel–Crafts condensations of benzene derivatives with functionalized phthalic anhydrides or phthaloyl dichlorides [8], Hauser annulations of cyanophthalides with cyclohexenones [9], Diels–Alder reactions [10,11], cross-coupling reaction and transition metal-mediated reactions [5,12] and biomimetic aldol condensations [13]. However, some annulation processes suffer from serious limitations which include the poorly efficient, and require several synthetic steps, harsh reaction conditions or using of substrates that are synthetically demanding. For the atropo-enantioselective total synthesis of axially chiral 4-arylanthraquinone knipholone-type natural products, the “lactone concept” has been applied [14].

9,10-Anthraquinones functionalized with amide-, alkylamino-, arylamino- and alkoxy-type groups have been successful obtained by reactions involving easily available 9,10-anthraquinones bearing amino [15,16] or hydroxy [17] groups, or anthraquinone derivatives bearing halogen atoms [18,19,20,21,22], or tosyloxy [23] reactive sites. For synthesis of amino substituted anthraquinones, which are increasingly widely used in practice, more and more attention has been drawn to the development of C-N coupling processes, for example, the copper(0)-catalyzed Ullmann-type reaction of bromo/chloro anthraquinones with a variety of amines [5,24,25,26] or the Pd-catalyzed Buchwald-Hartwig cross coupling reaction [27,28]. Currently, the most successfully developed method of functionalization of the anthraquinone core represent the Pd-catalyzed C-C cross-coupling reaction of 9,10-anthraquinones bearing suitable leaving groups, including anthraquinoyl triflates and bistriflates [29,30,31,32,33], halides [34,35] or boronic acid pinacol ester of 9,10-anthraquinone [36,37]. These processes enable the synthesis of site-specific organic materials for photonics and electronics [28,38,39,40] as well as biological active compounds [5,14,32,34] with 9,10-anthraquinone units.

In the framework of our studies dealing with the development of convenient routes to functionalization of some plant metabolites or their derivatives [41,42,43,44], we report herein the synthesis of a range of 1-hydroxy substituted anthraquinones containing an aryl substituent in the 2 or 4 (or 2 and 4 simultaneously) position of the anthraquinone core. As a starting compound, we used the 1-hydroxy-4-iodoanthraquinone (**1**), 1-hydroxy-2-bromoanthraquinone (**2**) or 1-hydroxy-2,4-dibromoanthraquinones (**3**) which were obtained from 1-hydroxy-9,10-anthraquinone or 4-amino-1-hydroxy-9,10-anthraquinone by the known procedures [45,46]. The Pd-catalyzed Suzuki–Miyaura cross-coupling reaction of the mentioned compounds with aryl boronic acids was the main route of synthesis. Taking into account the interest to substituted 4-arylanthraquinones as anticancer agents [4,6,7], we evaluated the cytotoxicity of the synthesized compounds toward a panel of cancer cell lines in vitro and also obtained some data about the potential mechanism of action of the new compounds.

Antracenedione drugs are known to exert their cytotoxic effects through interaction with DNA resulting in modification of its structure hence inhibition of its replication. Anthraquinone mitoxantrone is a potent synthetic anticancer drug which blocks DNA synthesis by inhibiting the function of DNA topoisomerase II. This compound inhibits the activities of both enzyme isoforms: topoisomerase IIα [47,48] and topoisomerase II*β* [1,49]. Several anthraquinone pharmacophores can realize their anticancer activity by affecting other molecular targets, such as proteins. Purpurin (1,2,4-trihydroxy-9,10-anthraquinone) is a non-competitive inhibitor of adipocyte-derived leucine aminopeptidase (A-LAP) which play a crucial role in angiogenesis [50]. Emodin (1,3,8-trihydroxy-6-methylanthraquinone) was characterized as a significant inhibitor of cell proliferation, presumably via down regulation of excision repair cross-complementary 1 (ERCC1) and DNA recombinase protein Rad51 [51], but its 2,4-dibromo derivatives exert their anti-proliferative activity at least in part, by inhibition of ATP citrate lyase (ACL), plays a critical role in generating cytosolic acetyl CoA [34]. Emodin and 2-chloroemodin were also considered as potential targets of dioxygenases (ALKBH 2, 3 proteins and FTO) overexpression blockers [52]. There was therefore value in a targeted preparation and investigation of novel hydroxyl-aryl substituted anthraquinones. The hydroxy substituent in the anthraquinones will be necessary for further improve the low druggability of the anthraquinone core.

## 2. Results and Discussion

### 2.1. Chemical Synthesis

**Scheme 1:**
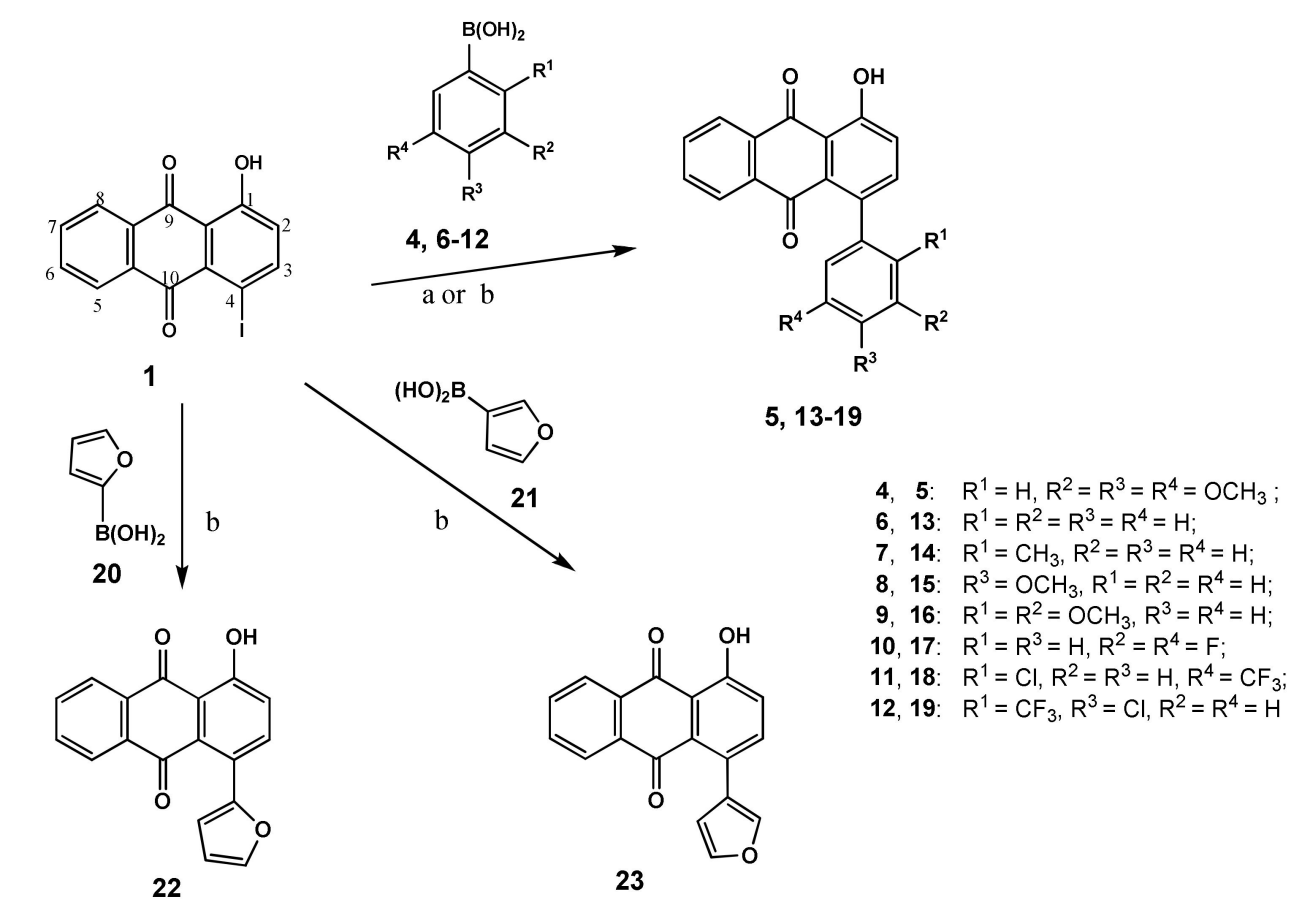
Synthesis of 4-substituted 1-hydroxyanthraquinones **5**, **13**–**19, 22**, **23**. Reagents and conditions: (a) 1.2 equiv. **4**, Pd(PPh_3_)_4_, K_2_CO_3_, Bu_4_NBr, dioxane, 100 °C, 11 h; (b) (1.2 equiv. **4**, **6–12**, **20**, **21**), Pd(PPh_3_)_4_, K_2_CO_3_, Bu_4_NBr, PhMe-H_2_O (5:1), 100 °C, 3–6 h.

2,6-Diiodo-9,10-anthraquinones [35], and bistriflates of 1,2-dihydroxy- [32,53], 1,3-dihydroxy- [33,53] and 2,6-dihydroxy-9,10-anthraquinones [31] were successfully involved in Suzuki–Miyaura reaction to synthesize bis-aryl(hetaryl)anthraquinones. The Suzuki reaction of 1-hydroxy-4-iodo-9,10-anthraquinone **1** with 3,4,5-trimethoxyphenyl-boronic acid (**4**) was used as the model reaction to optimize the conditions (Scheme 1). The reaction of **1** and **4** (4 equiv) in the presence of Pd(PPh_3_)_4_ (5 mol %) as the catalyst and NaHCO_3_ as the base in the conditions described for the cross-coupling reaction for 2-iodoemodine with aryl boronic acids (toluene-ethanol-water, 80 °C, 12 h) [34] led to 4-aryl-1-hydroxyanthraquinone **5** isolated in 89% yield. The coupling reaction of compound **1** with 1.2 equv. of boronic acid **4** under the mentioned conditions, led to the decreasing of the isolation yield to 76%. An efficient way to improve the yield of the cross-coupling reaction products was the addition of tetraalkylammonium salts to the reaction mixture as proposed by Jeffery [54]. The reaction of 1-hydroxy-4-iodo-9,10-anthraquinone **1** with 3,4,5-trimethoxyphenyl-boronic acid (**4**) (1,2 equiv) in the presence of Pd(PPh_3_)_4_ (10 mol %) as catalyst and K_2_CO_3_ (4 equiv.) as the base with the addition of ammonium salts (Bu_4_NBr) (1 equiv.) in dioxane proceeds by heating for 11 h (TLC) with the formation of the 4-aryl-1-hydroxyanthraquinone **5** in 85% yield. Performing the reaction in toluene-water requires shorter time (3 h) and ensures increased the isolated yield of compound **5** to 95%. In this condition, the reaction of 1-hydroxy-4-iodo-9,10-anthraquinone **1** with phenyl-**6**, *o*-tolyl-**7**, 4-methoxyphenyl- **8**, 2,3-dimethoxyphenyl-**9** or 3,5-difluorophenyl-**10** boronic acid led to the corresponding 4-aryl-1-hydroxyanthraquinones **13**–**17** in the yield 84%–92%. The reaction with 2-chloro-5-trifluorophenyl- **11** or 4-chloro-2-trifluorophenylboronic acid **12** with 4-iodoanthraquinone **1** required a longer reaction time (5.5–6 h) and afforded compounds **18**, **19** in the yield 45%–53%. The Suzuki cross coupling of **1** with 2-furylboronic acid or 3-furylboronic acid **20**, **21** gave 1-hydroxy-4-furylanthraquinones **22** or **23** in 84%–85% yield.

**Scheme 2:**
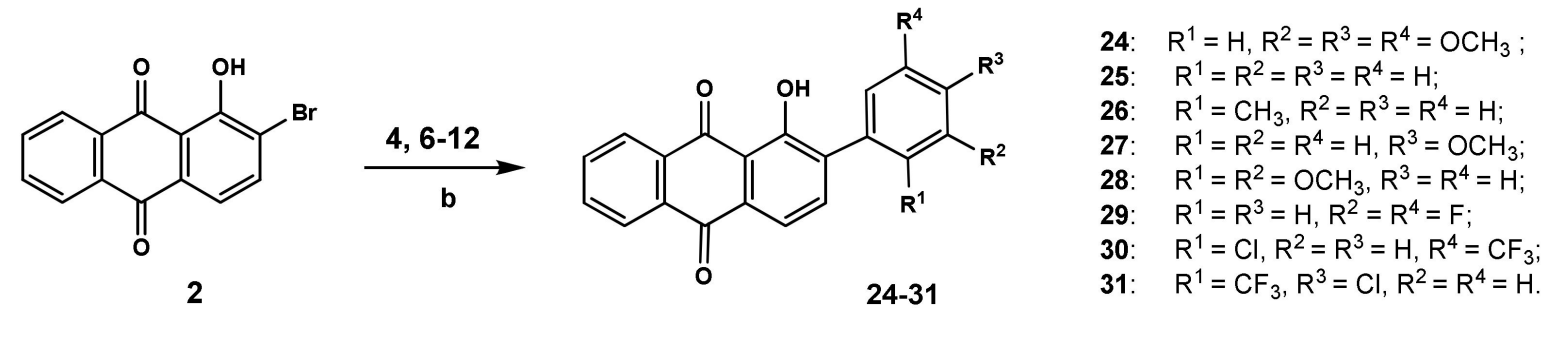
Synthesis of 4-substituted 1-hydroxyanthraquinones (**24–31**). Reagents and conditions: (b) (1.2 equiv. **4**, **6–12**), Pd(PPh_3_)_4_, K_2_CO_3_, Bu_4_NBr, PhMe-H_2_O (5:1), 100 °C, 3–6 h.

2-Bromo-9,10-anthraquinone **2** shown high activity in the Suzuki cross-coupling reaction with arylboronic acids **4**, **6–12** affording the subsequent 2-aryl-1-hydroxy-9,10-anthraquinones **24–31** in the isolated yield 52%–93% (Scheme 2).

**Scheme 3:**
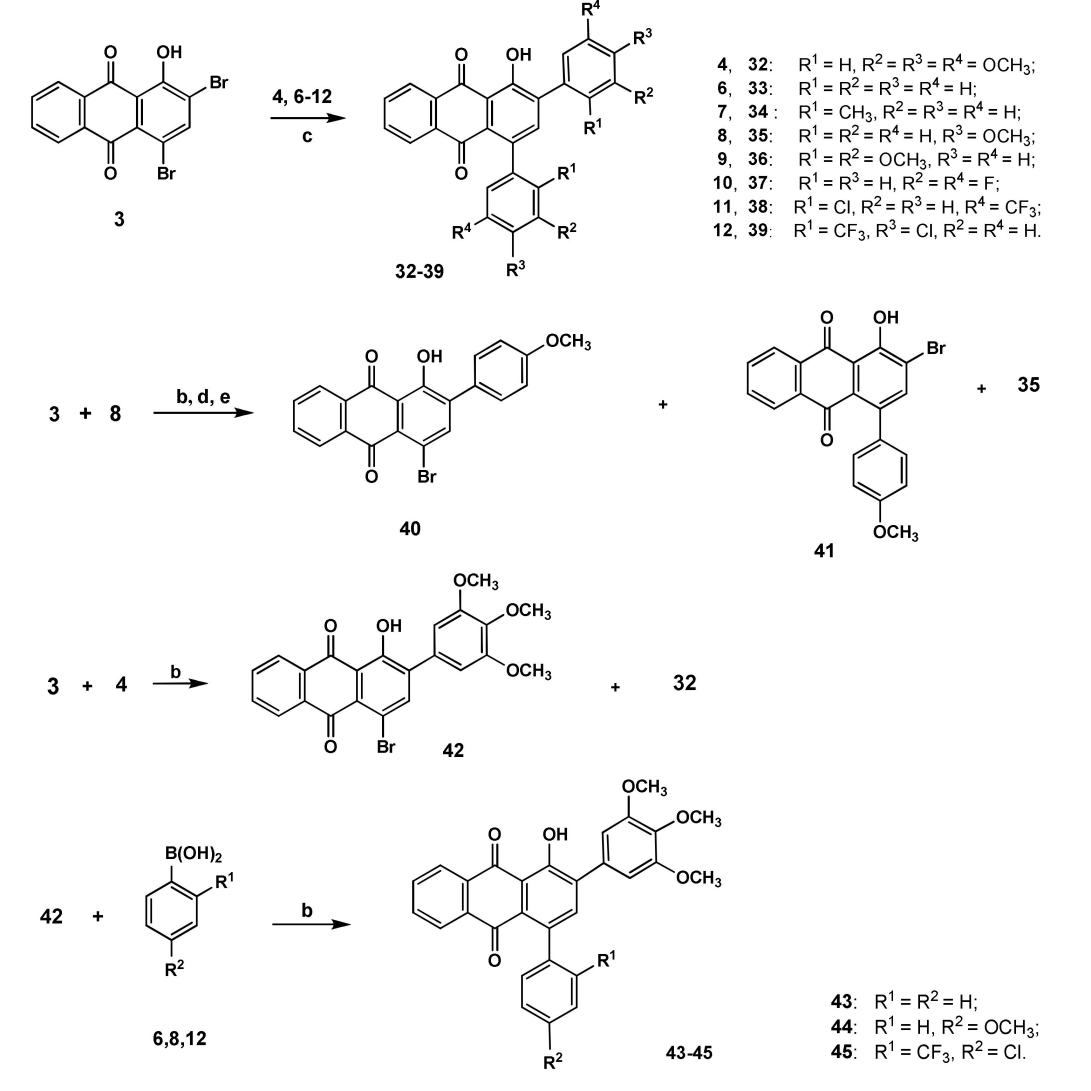
Synthesis of 2,4-disubstituted 1-hydroxyanthraquinones (**32–45**). Reagents and conditions: (b) 1.2 equiv. ArB(OH)_2_, Pd(PPh_3_)_4_, K_2_CO_3_, Bu_4_NBr, PhMe-H_2_O (5:1), 100 °C, 3–6 h; (c) (2.2 equiv. equiv. **4**, **6**–**12**), Pd(PPh_3_)_4_, K_2_CO_3_, Bu_4_NBr, PhMe-H_2_O (5:1), 100 °C, 4–6 h; (d) 1.2 equiv. **8**, Pd(PPh_3_)_4_, K_2_CO_3_, Bu_4_NBr, PhMe-H_2_O (5:1), 90 °C, 6 h; (e) 1.2 equiv. **8**, Pd(PPh_3_)_4_, K_2_CO_3_, Bu_4_NBr, PhMe-H_2_O (5:1), 80 °C, 6 h.

The reaction of 2,4-dibromo-9,10-anthraquinone **3** with arylboronic acids **4**,**6**–**12** (2.2 equiv, condition c) proceeds with the formation of 2,4-diaryl-1-hydroxyanthraquinones **32–39** (47%–93% isolated yield) (Scheme 3). Close scrutiny of the data obtained reveals that cross-coupling reaction of 2,4-dibromo-9,10-anthraquinone **3** opens the access to functionalized arylanthraquinones with different (diverse) substituent in the aromatic ring. The interaction of dibromo compound **3** with 4-methoxyphenylboronic acid **8** (1.2 equiv.) (condition b) afforded a mixture of 4-bromo-1-hydroxy-2-(4-methoxyphenyl)-9,10-anthraquinone **40** (39%), 2-bromo-1-hydroxy-4-(4-methoxyphenyl)-9,10-anthraquinone **41** (8%) and 1-hydroxy-2,4-di-(4-methoxyphenyl)- 9,10-anthraquinone **35** (40%). By decreasing the reaction temperature to 90 °C mono aryl substituted bromoanthraquinones **40**, **41** were obtained in the yield 30 and 29%; additionally, compounds **35** (27%) and **3** (8%) were isolate. By conducting the reaction at the temperature 80 °C for 6 h compounds **40** (35%), **41** (26%), **35** (22%) and **3** (17%) were isolated. The interaction of 2,4-dibromo-9,10-anthraquinone **3** with 3,4,5-trimethoxyphenylboronic acid (**4**) (1.2 equiv.) (conditions b) afforded a mixture of 4-bromo-1-hydroxy-2-(3,4,5-trimetoxyphenyl)-9,10-anthraquinone **42** (24%), 2,4-diaryl derivative **32** (45%) and the starting compound **3** (12%). The isolated 2-aryl-4-bromo-1-hydroxyanthraquinone **42** was involved in the cross-coupling reaction with aryl boronic acids **6**, **8**, **12**. The 2,4-diaryl-1-hydroxy-9,10-anthraquinones **43–45** were isolated in the yield 57%–89%. The cross-coupling reaction provide a convenient approach to diverse aryl substituted 1-hydroxy-9,10-anthraquinone derivatives.

The composition and structure of the synthesized compounds were confirmed by ^1^H, ^13^C NMR, IR and UV spectroscopy and mass-spectrometry data. The ^1^H and ^13^C NMR spectra of all synthesized compounds agree with their structure and contain one set of characteristic signals of 1-hydroxy-9,10-anthraquinone skeleton and the corresponding substituent. The distinctive feature of ^1^H NMR spectra for 4-aryl-1-hydroxyanthraquinones **5**, **13**, **15**, **17** and the corresponding 2-aryl substituted isomers **24**, **25**, **27**, **29** was the chemical shift of the protons H-1′ and H-6′ in the substituent to the high magnetic field (Δδ ~ 0.4 ppm). Like that, the 1-hydroxy substituent in compounds **24–31** appears its effect on the substituent in C-2 position. This substituent also displayed the effect on the down magnetic field chemical shift of carbon atoms in the 1′-position in the ^13^C NMR spectra of compounds **5**, **13–19**, compared with C-1′ shift of the substituent in **24**–**31**. In some cases, especially for compound **39** with two bulky substituents, aromatic signals broadening in the ^1^H NMR spectra was observed; for obtaining a satisfactory spin system the spectra of this compound were recorded in CD_3_CN.

### 2.2. Cytotoxicity Studies

One of the necessary steps in the study of the biological activity of potential pharmacological agents synthesized as oncolytics is the study of their cytotoxic profile in tumor cell cultures. This allows to evaluate the feasibility of their further research at the next stages of screening.

The cytotoxicity of the synthesized series of 4-substituted **5**, **13–17**, **22**, **23**, 2-substituted **25–30** and 2,4-disubstituted 1-hydroxy-9,10-anthraquinones **33–38, 40–45** was evaluated against a panel of seven different human cancer cell lines (glioblastoma cancer cells, human prostate cancer cells, T-cellular human leucosis, breast cancer cells) and also a normal cell line of hTERT-immortalized lung fibroblasts, using conventional MTT assay [55]. Doxorubicin (DOX) is clinically used to treat cancer as drug in world and have a very wide antitumor spectrum. That we use them as positive control compounds. The cytotoxicity was determined by measuring the concentration inhibiting human tumor cell viability by 50% (GI_50_). The results are presented in Table 1. The SAR revealed that the substituent at C-2, C-4 and C-2,4 position of 1-hydroxyanthraquinones have a great influence on the cytotoxicity. The synthesized 4-aryl substituted **5**, **15**, **16**, **17**, 2-aryl substituted **25**, **27**, **28** and 2,4-diaryl substituted **35**, **37**, **38**, **40** 1-hydroxyanthraquinones possess cytotoxicity towards glioblastoma cancer cells SNB-19, T98G and U-87MG (especially compounds **17**, **37** and **40**) with selectivity towards SNB-19 cells. The 2,4-(dimethoxyphenyl)-9,10-anthraquinone **35** and 2-(methoxyphenyl)-4-bromo-9,10-anthraquinone **40** shown a slightly more potent cytotoxicity towards all three type of glioblastoma cells than the subsequent 2- or 4-(dimethoxy)phenyl-9,10-anthraquinones **16**, **28**. 4-Bromo-1-hydroxyanthraquinone with a 4-(methoxyphenyl) substituent in the 2 position **40** was more effective than the subsequent 4-(3,4,5-trimethoxy) derivatives **42**. The 1,2,4-trisubstituted compound **38** demonstrated increase of potency against glioblastoma T98G cells against of the subsequent 1,2-disubstituted compound **30**.

1,4-Diaryl substituted 1-hydroxyanthraquinones **37** and **45** shown selective cytotoxicity towards prostate cancer cells LNCAP (GI_50_ 6.2–7.2 μM). The OMe- and CF_3_-groups in the 4-aryl substituent provided the selectivity for prostate cancer activity.

A remarkable increase in activity and selectivity towards prostate cancer cell line DU-145 was observed for 4-aryl substituted compounds **13**, **15**, **16**, **17** and 2,4-diaryl substituted derivatives **35**, **37**, **38**, **40**; all these compounds demonstrated inhibition against this prostate cancer cells in the micromolar concentration which is comparable or higher than that of the drug Doxorubicin. Characteristically, that the 4-phenylsubstituted anthraquinone **13** possess the best activity in DU-145 cell lines (GI_50_ 1.1 μM).

All compounds shown less activity against cells of T-cellular human leucosis MT-4.

Derivatives containing (2-methoxyphenyl)- or (3,5-difluorophenyl)- substituent in 4 position (**15**, **17**) and also 2,4 positions (**35**, **37**, **40**) were found to be active against breast cancer cells MDA-MB-231; the effect of the new anthraquinones **15**, **35** and **37** in this cell line was comparable to that of Doxorubicin.

Studying the comparable effect of the compounds on viability of human cancer lines revealed that compounds with an aryl substituent in the 4 position demonstrate the increase of potency compared with compounds having a furyl substituent in this position (**22**, **23**). Additionally, both C-2 aryl and C-4 aryl series were less cytotoxic towards the normal cell line than the 2,4-diarylated 1-hydroxyanthraquinone derivatives **34–38**, **45** and also the bromo-aryl substituted compounds **40–42**. Characteristically, the methoxy substitution is more favorable than the fluoro- or CF_3_-substitution in the aromatic rings in the C-4 and C-2 arylsubstituted series; these compound we less toxic towards the normal cell line model.

For further study, we selected 4-phenyl-**13** (GI_50_ = 1.1 μM on DU-145 cells), and 2-phenyl- **25** (GI_50_ = 6.8 μM, on SNB-19 cells), 4-(4-methoxyphenyl)- **15** (GI_50_ = 9.6 μM on SNB-19, 6.5 μM on DU-145, 6.8 μM on MDA-MB-231) and 2-(4-methoxyphenyl)- **27** (GI_50_ = 8.5 μM on SNB-19), 4-(2,3-dimethoxyphenyl)- **16** (GI_50_ = 9.7 μM on SNB19, 5.4 μM on DU-145) and 2-(2,3-dimethoxyphenyl)- **28** (GI_50_ = 5.77 μM on SNB-19) substituted 1-hydroxyanthraquinones. All these compounds were relatively safe towards non-cancer cells and demonstrated selectivity on subtype of cancer cells. The mode of action of natural 4-arylanthraquinones has not been established. These compounds triggered both apoptosis and necrosis and also induced DNA damage in cancer cells [7]. We further studied the action of the synthesized arylanthraquinones on the beta isoform of human topoisomerase II in silico.

### 2.3. Molecular Docking of Compounds 13, 15, 16, 23, 25, 27, 28, 35, 40, 44 with the topoisomerase IIβ-DNA

**Figure 1:**
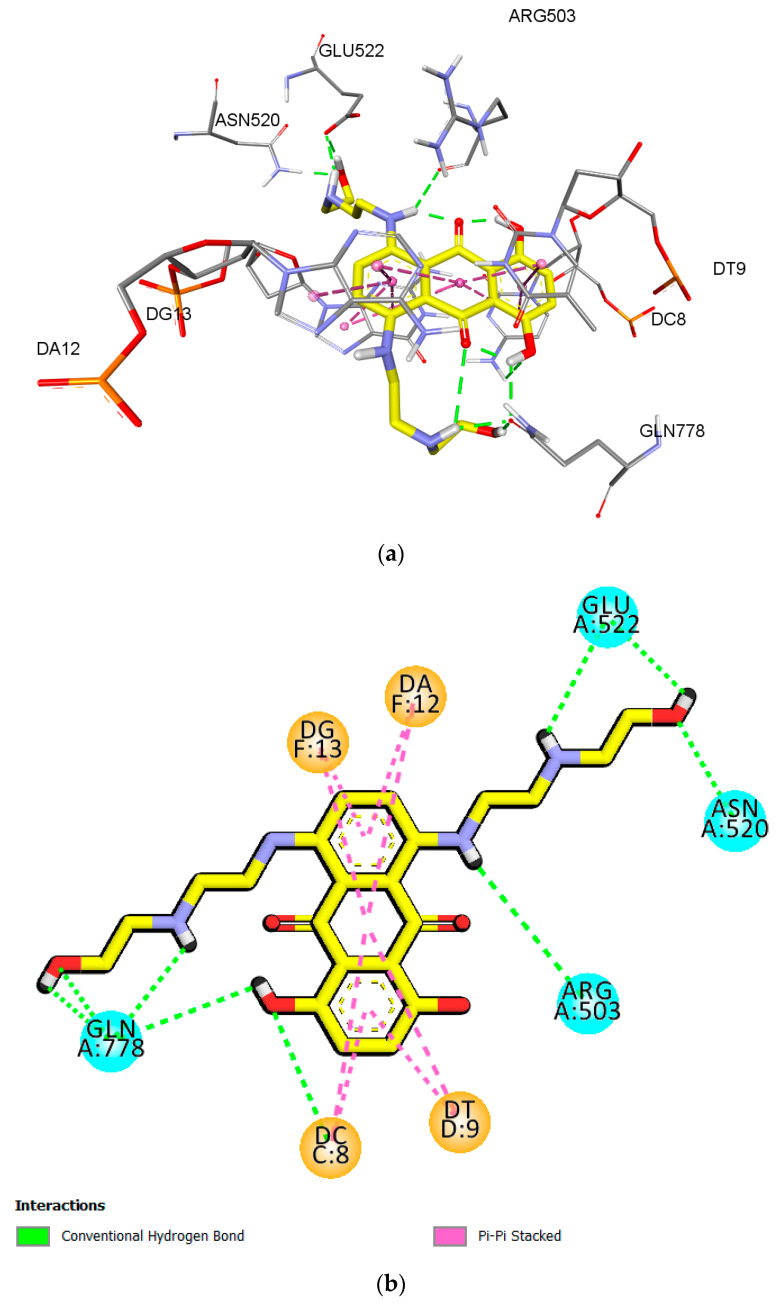
Docking of known inhibitors in topoisomerase IIβ-DNA complex binding site. Mitoxantrone: (**a**) spatial conformation, (**b**) interaction diagram. Noncovalent interactions of molecule are shown by dotted lines: green—hydrogen bonds, purple—stacking interactions. Nucleotides are indicated by orange spheres, amino acid residues of topoisomerase are indicated by blue spheres.

To better understand the possible binding patterns and to guide further SAR studies, molecular docking studies of compounds **13**, **15**, **16**, **23**, **25**, **27**, **28**, **35**, **40**, **44** and mitoxantrone with the topoisomerase IIβ-DNA complex was performed. It is well known that substituted anthraquinones enable the interaction with DNA-metabolizing enzymes and the perturbation of the replication and transcription of genetic information process using enzymes topoisomerase I and II (topo I and II) as inhibitors [49,56]. The polar groups of the mentioned topoisomerase inhibitors can take part in the formation of hydrogen bonds with the amino acid residues of the enzyme, further stabilizing its non-working conformation. This is especially characteristic of the mitoxantrone molecule, whose 2-(2-hydroxyethylamino)ethylamino symmetrical substituents contain polar groups that can form hydrogen bonds with GLN778, GLU522, ARG503 and ASN520 amino acid residues of topoisomerase (Figure 1).

**Figure 2:**
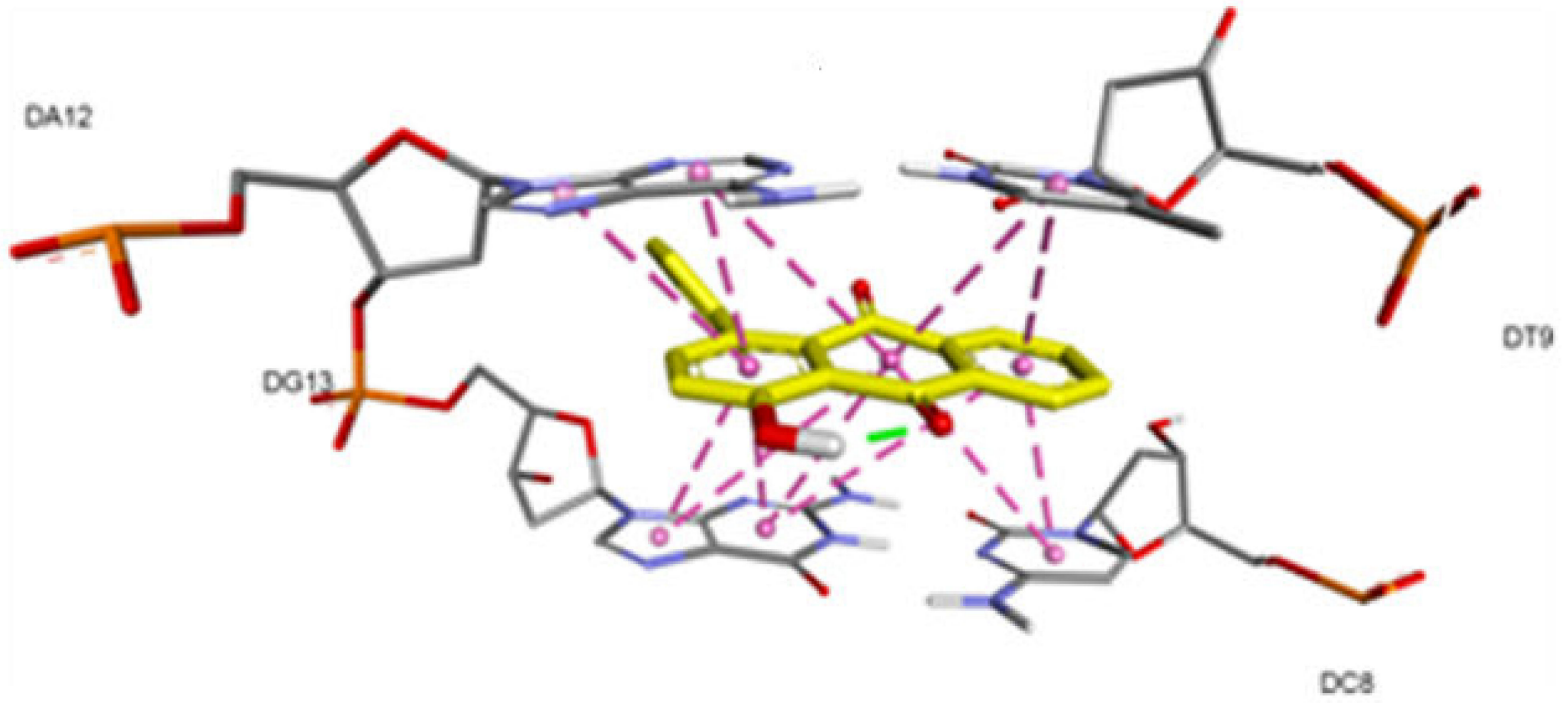
Docking of compound **13** in topoisomerase IIβ-DNA complex binding site. Non-covalent interactions of molecules are shown by dotted lines: green—hydrogen bonds, orange—electrostatic interactions, yellow—sulfur interactions, purple—stacking interactions.

We carried out molecular modeling of the possible interaction of new compounds **13**, **15**, **16**, **23**, **25**, **27**, **35**, **40**, **44** and topoisomerase IIβ-DNA complex. The results of docking studies are listed in Table 2, Figure 2 and Figure S2 Supplementary Materials. The anthraquinone motif of new compounds can be successfully inserted between pairs of nitrogenous bases, forming many stacking interactions with purine and pyrimidine π-systems. The aryl substituent in C-4 position of compounds **13**, **15**, **44** provides the formation of an intramolecular hydrogen bond of the hydroxyl group at C-1 with the carbonyl group at C-9 atom (Figure 2, Figure S2B,J). 1-Hydroxy groups of compounds **15, 25, 28** and **40** are capable of forming the hydrogen bond with GLN778 and ARG503 amino acid residues of topoisomerase, respectively (Figure S2B,E,G,I). Apparently, the presence of methoxyphenyl substituents have little effect on the formation of stacking interactions of the anthraquinone center of molecules **15**, **27**, **35** and **44**. The methoxy groups of these substituents in all cases do not participate in the formation of hydrogen bonds. However, for compound **35**, the π-system of the 4-methoxyphenyl substituent is involved in the formation of additional stacking interactions with purine nitrogen base A12 (Figure S2, Supplementary Materials); and for compounds **16** and **44**, the aromatic rings were involved in the interaction with MET782 (Figure S2, Supplementary Materials).

The binding affinity of the synthesized ligands **13**, **15**, **16**, **23**, **25**, **27**, **35**, **40**, **44** and **45** was evaluated with energy scope and compared with a minimum binding energy of Mitoxantrone on the results of re-docking. The high negative dock score was observed only for mitoxantrone. The binding affinity of the synthesized aryl substituted 1-hydroxyanthraquinones was comparable.

### 2.4. Electrophoretic Mobility

**Figure 3:**
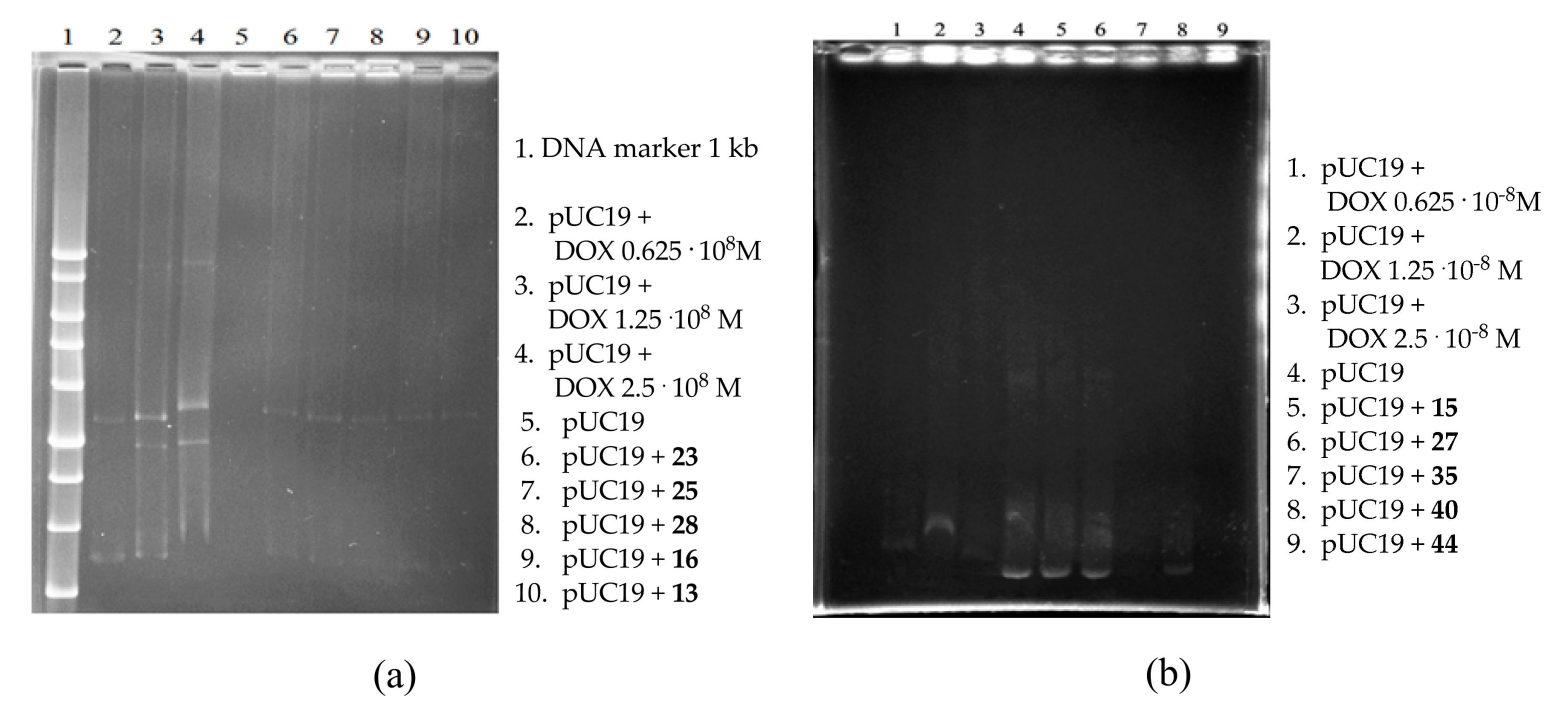
Gel retardation assay of tested aryl(furyl) substituted 1-hydroxyanthraquinones **13**, **16**, **23**, **25**, **28** (**a**) and (4-methoxyphenyl) substituted compounds **15**,**27**,**35**,**40**,**44** (**b**) with pUC19 DNA plasmid.

The interaction of compounds **13**, **15, 16**, **23**, **25 27**, **28, 35, 40** and **44** with DNA was experimentally confirmed by study of the electrophoretic mobility. For this the gel retardation assay was performed. Doxorubicin in different concentration was used as a positive control. In order to avoid destruction of the possible complex of DNA with compounds, the electrophoresis was performed at low voltage. The results are presented at Figure 3. It was shown that 4-aryl and 4-(furan-3yl) substituted compounds **13**, **15**, **16**, **23**, 2-(4-methoxyphenyl)- **27** and also 2,4-(4-dimethoxyphenyl)- **35** substituted anthraquinones cause a retardation of plasmid DNA which could indicate the formation of complexes.

The results taken into suggest that DNA interaction is a necessary component for mediating aryl substituted 1-hydroxy-9,10-anthraquinone-induced cell death but can not account for the differences in their cytotoxic potential entirely.

### 2.5. Cell Cycle and DNA Synthesis Analysis

**Figure 4:**
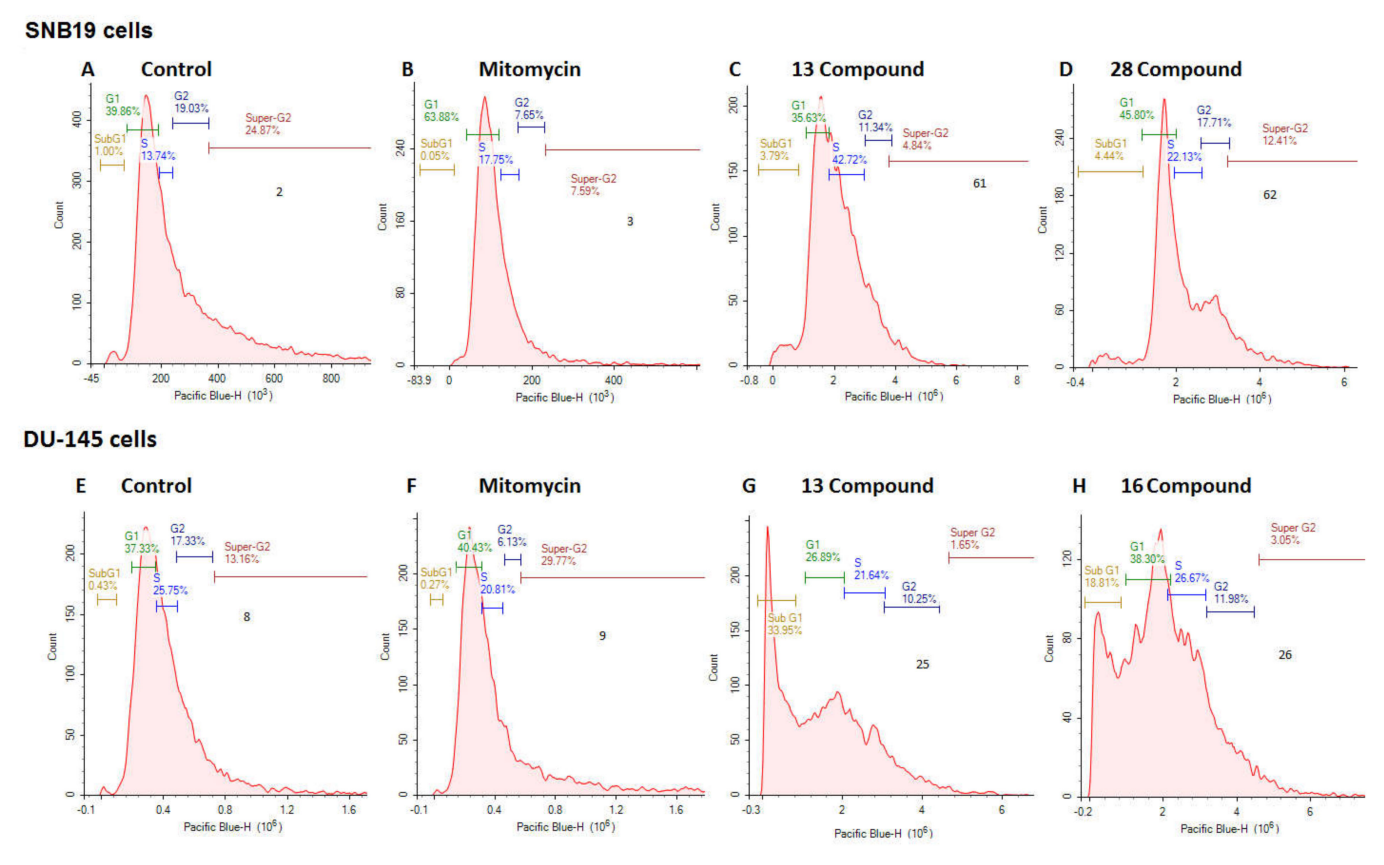
Induction of apoptosis by Mitomycin C (**B**), compounds **13** (**C**) and **28** (**D**) in SNB19 cells and Mitomycin C (**F**), 4-aryl-1-hydroxy-9,10-anthraquinones **13** (**G**), **16** (**H**) in DU-145 cells. Control (non-treated cells) (**A**,**E**). Data were obtained by flow cytometry (excitation 405 nm, emission 445 nm), in each sample 10,000 events were counted.

Due to the fact that the implementation of the antitumor potential of the currently existing antitumor agents is carried out by acting on various biological targets, at the next stage we performed the cell cycle analysis and DNA synthesis. Cell cycle analysis of 1-hydroxy-4-phenyl-9,10-anthraquinone **13**, 1-hydroxy-4-(2,3-dimethoxyphenyl)-9,10- anthraquinone **16**, 1-hydroxy-4-(furan-3-yl)-9,10-anthraquinone **23** and 1-hydroxy- 2-(2,3-dimethoxyphenyl)-9,10-anthraquinone **28** against SNB-19 cells for 24 h produced interesting results. After examining the data for compounds **13** and **16** (Table 3, Figure 4C,D), good number of cells are distributed in S phase, i.e., initiated DNA replication mechanism and also in G0/G1 phase, i.e., initial phase of cell cycle. G1 and G2 are the growth phases in cell cycle analysis, whereas S phase is a synthetic phase wherein DNA replication and DNA synthesis take place. The 2-aryl substituted compound **28** initiated the G0/G1 phase in analogy with doxorubicin and mitomycin. Direct attack on cell regulatory protein is suggested. Further, compounds play a vital role in controlling the regulation of Sub-G1 and G2/M phases. Some difference in the mechanism of operation of 4-aryl substituted **13**,**16** and 2-aryl substituted 9,10-anthraquinones **28** has been observed during the progression of cell cycle like: S phase was highly disturbed by **13**; G0/G1 phase was highly disturbed by **28**. Interestingly, 4-arylsubstituted 1-hydroxyanthraquinones **13**,**16** enhancement of DNA synthesis on SNB-19 cells (Table 3). This observation can be explained by the influence on the cell repair systems that respond to the intercalation of compounds in DNA. It is well established that the anthraquinone drug mitoxantrone arrests G1 and G2 phases at cell cycle progression and ultimately inhibits the cell growth [57]. Further, mitoxantrone promotes the arrest of S phase of cell cycle. Hence, cell goes apoptosis upon treatment with mitoxantrone due to inhibition at cell growth phases G1 and G2 along with inhibiting the DNA replication/duplication process (S phase).

Characteristically, the induction of apoptosis by the cell cycle arrest at G1 phase in MDA-MB-231 breast cancer cells was also established for the 1-arylanthraquinone containing fraction of the medicinal plant *Bulbine frutescens* [58].

Compound **13** in accordance to cytotoxic studies on DU-145 cell lines shows better effect over Doxorubicin, i.e., 1.1 and 2.0 μM, respectively (Table 1). We have carried out flow cytometric study of compounds **13**, **16** and the isomeric compound **25** (Table 4, Figure 4G,H). Concentrations of **16** and **25** were taken as 5.4 and 14.5 μM, respectively, as per cytotoxic studies (Table 1). After examining the data for **13** (Figure 4G, Table 4), a good number of cells are arrested in Sub-G1, G0/G1 and S phase. Biosynthetic activity is very high during Sub-G1 phase; this is probably achieved by enhancing of apoptosis. The isomeric molecule **25** arrest G0/G1 and S phases, i.e., affecting the DNA synthesis/replication mechanism. Similarly, for **25** a different pattern is observed, i.e., variations at S and G2/M phases were observed, in comparison to the control. Hence, both the isomeric molecules **13** and **25** deregulate the cell cycle which is the primary condition for any drug candidate to be cytotoxic. Some difference in the mechanism of operation of **13** and **25** has been observed during the progression of cell cycle like: Sub-G1 phase was highly disturbed by **13** and S and G0/G1 phases were highly disturbed by **25**.

## 3. Conclusions

A straightforward methodology has been developed for the introduction of an aryl substituent at C-2, C-4 or C-2,4 positions in the anthraquinone core in a two-step procedure starting from 1-hydroxyanthraquinone by Suzuki–Miyaura cross coupling reaction of the subsequent halogen substituted anthraquinones with aryl (hetaryl) boronic acids. The cytotoxicity of twenty-six novel compounds was tested against a panel of seven human tumor cell lines and also towards hTERT-immortalized lung fibroblast cells in the MTT assay. Cytotoxicity studies revealed that six of the aryl substituted compounds among the entire series **3**, **15**, **16**, **25**, **27**, **28** are more potent than the commercially available reference drug doxorubicin against human glioblastoma SNB-19, prostate cancer DU-145 or breast cancer MDA-MB-231 cells and relatively safe towards hTERT-immortalized lung fibroblasts cells. The structure-cytotoxicity investigations implied that the phenyl, 4-methoxyphenyl, 2,3-dimethoxyphenyl or 3,5-difluorophenyl substituted 1-hydroxyanthraquinones exhibited the higher cytotoxicity in glioblastoma cancer cell lines. Another observed effect is the enhancement of DNA synthesis in SNB-19 cells for 4-aryl 1-hydroxyanthraquinones **13**, **16** compared with doxorubicin and especially mitomycin C can be explained by the strengthening of cell repair systems that respond to the intercalation of compounds in DNA.

## 4. Experimental Section

### 4.1. General Information

IR spectra were recorded by means of the KBr pellet technique on a Bruker Vector-22 spectrometer. UV spectra were obtained on an HP 8453 UV–Vis spectrometer (Hewlett-Packard, Waldbronn, Germany) in EtOH. ^1^H and ^13^C NMR spectra were acquired on Bruker Avance 300 (^1^H: 300.15 MHz, ^13^C: 75.47 MHz), 400 (^1^H: 400.13 MHz, ^13^C: 100.78 MHz), DRX-500 (^1^H: 500.13 MHz, ^13^C: 125.76 MHz) and Avance 600 (^1^H: 600.30 MHz, ^13^C: 150.95 MHz) spectrometers (Bruker BioSpin GmbH, Rheinstetten, Germany), using tetramethylsilane (TMS) as an internal standard. NMR signal assignments were carried out with the aid of a combination of 1D and 2D NMR techniques that included Heteronuclear Single Quantum Correlation (HSQC) and Heteronuclear Multiple Bond Correlation (HMBC). Chemical shifts are reported in parts per million (ppm) and coupling constants are expressed in Hz. HRMS spectra were recorded on a Thermo Scientific DFS mass spectrometer (evaporator temperature 200–230 °C, EI ionization at 70 eV). Melting points were determined using termosystem Mettler Toledo FP900 (USA). The analytical and spectroscopic investigations were carried out at the Collective Use Center for Chemical Services of the Siberian Branch of the Russian Academy of Sciences.

The reaction progress was monitored by TLC on Silufol UV-254 plates (Kavalier, Czech Republic), CHCl_3_-EtOH, 100:1; detection under UV light. Column chromatography was performed by using silica gel (0.070–0.230 mm, Acros-Organics). Purity of all compounds was checked by TLC.

The chemicals used: arylboronic acids **4**, **6–10**, 2- and 3-furylboronic acids **20**, **21**, Pd(PPh_3_)_4_, Bu_4_NBr were purchased from Sigma-Aldrich (St. Louis, MO, USA) or Alfa Aesar (GmbH, Karlsruhe, Germany). 1-Hydroxy-4-iodoanthraquinone (**1**) [45], 1-hydroxy-2-bromoantrhraquinone (**2**) or 1-hydroxy-2,4-dibromoanthraquinone (**3**) [46] were synthesized according to published procedures. Solvents (dioxane, PhMe, CH_2_Cl_2_, CHCl_3_, MeOH) and Et_3_N were purified by standard methods and distilled under a stream of argon just before use.

### 4.2. Syntheses and Spectral Data

#### 4.2.1. Procedures for Suzuki–Miyaura Reactions

(a) A mixture of 4-iodo-1-hydroxyanthraquinone **1** (1 mmol) and 3,4,5-trimethoxyphenylboronic acid (**4**) (1.2 mmol) was stirred in dry dioxane (100 mL) in the presence of a catalytic amount of Pd(PPh_3_)_4_ (10 mol %), Bu_4_NBr (1 mmol) and K_2_CO_3_ (4 mmol) at 100 °C for 11 h in an argon flow. Afterward, water was added and the product was extracted with chloroform. The combined organic layers were dried (MgSO_4_), filtered and the filtrate was concentrated in vacuo. The residue was purified by column chromatography (benzene) to afford the corresponding compound **5** in 85 % yield.

(b) A mixture of 4-iodo-1-hydroxyanthraquinone **1** or 2-bromo-1-hydroxyanthraquinone **2** (1 mmol) and subsequent aryl boronic or furyl boronic acids (**4**, **6–12**, **20**, **21**) (1.2 mmol), Pd(PPh_3_)_4_ (10 mol %), Bu_4_NBr (1 mmol) and K_2_CO_3_ (4 mmol) was stirred in toluene–water mixture (100 and 20 mL) at 100 °C for 3–4 h (TLC) in an argon flow. After cooling the mixture was diluted with benzene (100 mL) and washed with water. Organic layer was dried over MgSO_4_, filtered and concentrated under reduced pressure. The residue was purified by column chromatography (benzene or CCl_4_/benzene, 1:1) to afford the corresponding compounds (**13–19**, **22**–**31**).

The reaction of 2,4-dibromo-1-hydroxyanthraquinone **3** (1 mmol) with 3,4,5-trimethoxyphenylboronic acid (**4**) (1.2 mmol) in condition (b) afforded a mixture of 4-bromo-1-hydroxy-2-(3,4,5-trimethoxyphenyl)-9,10-anthraquinone **42** (34%), 1-hydroxy-2,4-(3,4,5-trimethoxyphenyl)-9,10-anthraquinone **5** (40%) and the starting compound **3** (12%).

By reaction of compound **42** with aryl boronic acids **6**, **8** or **12** in condition (b) the subsequent diaryl substituted 1-hydroxyanthraquinones (**43**–**45**) were obtained in the yield 57%–89%.

(c) A mixture of 2,4-dibromo-1-hydroxyanthraquinone **3** (1 mmol) and subsequent aryl boronic acid (**4**, **6–12**) (2.2 mmol), Pd(PPh_3_)_4_ (10 mol %), Bu_4_NBr (1 mmol) and K_2_CO_3_ (4 mmol) was stirred in toluene–water mixture (100 and 20 mL) at 100 °C for 4–6 h (TLC) in an argon flow. After cooling to 25 °C, the mixture was diluted with benzene (200 mL) and washed with water. Organic layer was dried over MgSO_4_, filtered and concentrated under reduced pressure. The residue was purified by column chromatography (benzene or CHCl_3_) to afford the corresponding compounds (**32–39**) in 47%–93%.

(d) A mixture of 2,4-dibromo-1-hydroxyanthraquinone **3** (1 mmol) and 4-methoxyphenylboronic acid (**8**) (1.2 mmol), Pd(PPh_3_)_4_ (5 mol %), Bu_4_NBr (1 mmol) and K_2_CO_3_ (4 mmol) was stirred in toluene–water mixture (100 and 20 mL) at 90 °C for 6 h in an argon flow. After cooling to 25 °C, the mixture was diluted with benzene (100 mL) and washed with water. Organic layer was dried over MgSO_4_, filtered and concentrated under reduced pressure. The residue was purified by column chromatography (benzene, CCl_4_/benzene, 1:1) to afford the corresponding monosubstituted bromoanthraquinones 4-bromo-1-hydroxy-2-(4-methoxyphenyl)-9,10- anthraquinone **40** (30%), 2-bromo-1-hydroxy-4-(4-methoxyphenyl)-9,10-anthraquinone **41** (29%), 1-hydroxy-2,4-di-(4-methoxyphenyl)-9,10-anthraquinone **35** (27%). The reaction in conditions (b, 100 °C, 3 h) led to the isolation of **40** (39%), **41** (8%) and **35** (40%).

(e) The above reaction at 80 °C for 6 h gave compounds **35** (22%), **40** (35%), **41** (26%) and compound **3** (17%).

All reported compounds were obtained in purities of 95% and above.

#### 4.2.2. Characteristics of Compounds (**5, 13–19, 22–45**)

1-Hydroxy-4-(3,4,5-trimethoxyphenyl)anthracene-9,10-dione (**5**).

Yield 85% (a), 95% (b). M.p.: 230.5 °C (decomp.); ^1^H NMR (500 MHz, CDCl_3_, δ, ppm): δ 3.86 (s, 6H, 3′,5′-OCH_3_), 3.95 (s, 3H, 4′-OCH_3_), 6.48 (s, 2H, H-2′,6′), 7.29 (m, 1H, H-2), 7.51 (d, *J* = 8.67 Hz, 1H, H-3), 7.78 (m, 2H, H-6,7), 8.13 (m, 1H, H-5), 8.31 (m, 1H, H-8), 13.18 (s, 1H, OH); ^13^C NMR (125 MHz, CDCl_3_, δ, ppm): δ 56.0 (3′,5′-OCH_3_), 60.8 (4′-OCH_3_), 105.4 (C-2′,6′), 116.3 (C-9a), 123.4 (C-2), 126.4 (C-8), 127.3 (C-5), 130.1 (C-4), 132.4 (C-10a), 133.6 (C-6), 134.5 (C-8a), 134.7 (C-7), 136.5 (C-4a), 137.2 (C-1′), 137.4 (C-4′), 140.6 (C-3), 153.0 (C-3′,5′), 162.6 (C-1), 182.5 (C-10), 189.0 (C-9); IR (KBr, ν, cm^−1^): 3442 (OH), 1680 (C=O), 1637, 1585 (C=C); UV/Vis: λ_max_, nm (lgε): 250 (4.16), 409 (3.34); HR-MS (ESI), calcd. C_23_H_18_O_6_, [M]^+^ *m*/*z*: 390.1098. found: 390.1101.

1-Hydroxy-4-phenylanthracene-9,10-dione (**13**).

Yield 90% (b). M.p.: 207.3 °C (decomp.); ^1^H NMR (400 MHz, CDCl_3_, δ, ppm): δ 7.22–7.26 (m, 2H, H-3′,5′), 7.29 (d, *J* = 8.70 Hz, 1H, H-2), 7.38–7.45 (m, 3H, H-2′,4′,6′), 7.47 (d, *J* = 8.70 Hz, 1H, H-3), 7.74 (m, 2H, H-6,7), 8.08 (m, 1H, H-5), 8.28 (m, 1H, H-8), 13.19 (s, 1H, OH); ^13^C NMR (100 MHz, CDCl_3_, δ, ppm): 116.3 (C-9a), 123.5 (C-2), 126.4 (C-8), 127.0 (C-4′), 127.4 (C-5), 127.9 (2CH), 128.1 (2CH), 130.0 (C-4), 132.4 (C-10a), 133.6 (C-6), 134.4 (C-8a), 134.7 (C-7), 136.7 (C-4a), 140.6 (C-3), 141.8 (C-1′), 162.6 (C-1), 182.7 (C-10), 189.1 (C-9); IR (KBr, ν, cm^-1^): 3428 (OH), 1674 (C=O), 1627, 1589 (C=C); UV/Vis: λ_max_, nm (lgε): 224 (4.38), 253 (4.51), 409 (3.72); HR-MS (ESI), calcd. C_20_H_12_O_3_, *m*/*z* [M]^+^ 299.0781. found 299.0776.

1-Hydroxy-4-(*o*-tolyl)anthracene-9,10-dione (**14**).

Yield 90% (b). M.p.: 195.1–195.2 °C; ^1^H NMR (400 MHz, CDCl_3_, δ, ppm): δ 2.02 (s, 3H, CH_3_), 7.03 (d, *J* = 7.50 Hz, 1H, H-3′), 7.24-7.35 (m, 4H, H-2,4′,5′,6′), 7.40 (d, *J* = 8.70 Hz, 1H, H-3), 7.74 (m, 2H, H-6,7), 8.08 (m, 1H, H-5), 8.29 (m, 1H, H-8), 13.21 (s, 1H, OH); ^13^C NMR (100 MHz, CDCl_3_, δ, ppm): 19.9 (CH_3_), 116.3 (C-9a), 123.9 (C-2), 125.8 (CH), 126.4 (C-8), 127.2 (CH), 127.4 (C-5,CH), 129.6 (CH), 130.0 (C-4), 132.5 (C-10a), 133.7 (C-6), 134.1 (C-8a), 134.6 (C-7), 134.9 (C-2′), 135.8 (C-4a), 140.3 (C-3), 141.6 (C-1′), 162.5 (C-1), 182.5 (C-10), 189.2 (C-9); IR (KBr, ν, cm^−1^): 3435 (OH), 1674 (C=O), 1637, 1589 (C=C); UV/Vis: λ_max_, nm (lgε): 254 (4.53), 325 (3.52), 407 (3.79); HR-MS (ESI), calcd. C_21_H_14_O_3_, *m*/*z* [M]^+^ 314.0938. found 314.0943.

1-Hydroxy-4-(4-methoxyphenyl)anthracene-9,10-dione (**15**).

Yield 92% (b). M.p.: 209.2–209.4 °C; ^1^H NMR (300 MHz, CDCl_3_, δ, ppm): δ 3.86 (s, 3H, OCH_3_), 6.96 (m, 2H, H-3′,5′), 7.17 (m, 2H, H-2′,6′), 7.27 (d, *J* = 8.74 Hz, 1H, H-2), 7.47 (d, *J* = 8.74 Hz, 1H, H-3), 7.74 (m, 2H, H-6,7), 8.10 (m, 1H, H-5), 8.27 (m, 1H, H-8), 13.20 (s, 1H, OH); ^13^C NMR (125 MHz, CDCl_3_, δ, ppm): 55.1 (CH_3_), 113.6 (C-3′,5′), 116.4 (C-9a), 123.5 (C-2), 126.4 (C-8), 127.4 (C-5), 129.2 (C-2′,6′), 129.9 (C-4), 132.4 (C-10a), 133.6 (C-7), 133.9 (C-1′), 134.5 (C-8a), 134.6 (C-6), 136.5 (C-4a), 141.1 (C-3), 158.6 (C-4′), 162.5 (C-1), 182.9 (C-10), 189.1 (C-9); IR (KBr, ν, cm^−1^): 3420 (OH), 1674 (C=O), 1637, 1591 (C=C); UV/Vis: λ_max_, nm (lgε): 225 (4.39), 254 (4.56), 321 (3.65), 409 (3.69); HR-MS (ESI), calcd. C_21_H_14_O_4_,_._ *m/z* [M]^+^ 330.0887. found 330.0871.

1-(2,3-Dimethoxyphenyl)-4-hydroxyanthracene-9,10-dione (**16**).

Yield 66% (b). M.p.: 213.6 °C (decomp.); ^1^H NMR (500 MHz, CDCl_3_, δ, ppm): δ 3.51 (s, 3H, 3′-OCH_3_), 3.91(s, 3H, 2′-OCH_3_), 6.73 (d, *J* = 8.00, 1.50 Hz, 1H, H-4′), 6.97 (d, *J* = 8.05 Hz, 1H, H-6′), 7.12 (t, *J* = 8.00 Hz, 1H, H-5′), 7.29 (d, *J* = 8.65 Hz, 1H, H-2), 7.48 (d, *J* = 8.65 Hz, 1H, H-3), 7.73 (m, 2H, H-6,7), 8.10 (m, 1H, H-5), 8.28 (m, 1H, H-8), 13.15 (s, 1H, OH); ^13^C NMR (125 MHz, CDCl_3_, δ, ppm): 55.7 (3′-OCH_3_), 60.3 (2′-OCH_3_), 111.6 (C-4′), 116.4 (C-9a), 121.0 (C-5′), 123.3 (C-2), 124.0 (C-6′), 126.4 (C-8), 127.4 (C-5), 130.9 (C-4), 132.2 (C-1′), 132.6 (C-10a), 133.5 (C-6), 134.4 (C-8a), 134.6 (C-7), 136.3 (C-4a), 140.6 (C-3), 145.5 (C-3′), 152.6 (C-2′), 162.6 (C-1), 182.7 (C-10), 189.2 (C-9); IR (KBr, ν, cm^-1^): 3431 (OH), 1672 (C=O), 1635, 1593 (C=C); UV/Vis: λ_max_, nm (lgε): 225 (4.08), 253 (4.88), 408 (4.10); HR-MS (ESI), calcd. C_22_H_16_O_5_,_._*m*/*z* [M]^+^ 360.0992. found 360.0989.

1-(3,5-Difluorophenyl)-4-hydroxyanthracene-9,10-dione (**17**).

Yield 84% (b). M.p.: 214.2–214.3 °C; ^1^H NMR (400 MHz, CDCl_3_, δ, ppm): δ 6.74 (m, 2H, H-2′,6′), 6.82 (ddd, *J* = 8.6, 8.9, 2.45, 2.25 Hz, 1H, H-4′), 7.28 (d, *J* = 8.75 Hz, 1H, H-2), 7.42 (d, *J* = 8.75 Hz, 1H, H-3), 7.77 (m, 2H, H-6,7), 8.08 (m, 1H, H-5), 8.28 (m, 1H, H-8), 13.15 (s, 1H, OH); ^13^C NMR (100 MHz, CDCl_3_, δ, ppm): 102.5 (C-4′, *J*_C-F_ = 25.2 Hz), 111.1 (C-6′, *J*_C-F_ = 8.0 Hz), 111.3 (C-2′, *J*_C-F_ = 8.0 Hz), 116.4 (C-9a), 123.7 (C-2), 126.5 (C-8), 127.4 (C5), 130.2 (C), 132.3 (C), 133.9 (C6), 133.9 (C-4, *J* = 2.4 Hz), 134.1 (C), 134.8 (C-7), 139.7 (C-3), 145.0 (C-1′, *J*_C-F_ = 10.0 Hz), 162.6 (C-3′, *J*_C-F_ = 248 Hz), 162.8 (C-5′, *J*_C-F_ = 248 Hz), 163.0 (C-1), 182.4 (C-10), 189.9 (C-9); IR (KBr, ν, cm^−1^): 3440 (OH), 1672 (C=O), 1621, 1593 (C=C); UV/Vis: λ_max_, nm (lgε): 222 (4.42), 254 (4.53), 328 (3.47), 405 (3.77); HR-MS (ESI), calcd. C_20_H_10_F_2_O_3_, *m*/*z* [M]^+^ 336.0593. found 336.0572.

1-(2-Chloro-5-(trifluoromethyl)phenyl)-4-hydroxyanthracene-9,10-dione (**18**).

Yield 84% (b). M.p.: 184.3 °C (decomp.); ^1^H NMR (300 MHz, CDCl_3_, δ, ppm): δ = 7.36 (d, *J* = 8,8 Hz, 1H, H-2), 7.40 (d, *J* = 8.8 Hz, 1H, H-3), 7.46 (br.s, 1H, H-6′), 7.59 (m, 2H, H-3′,4′), 7.77 (m, 2H, H-6,7), 8.09 (m, 1H, H-5), 8.30 (m, 1H, H-8), 13.16 (s, 1H, OH); ^13^C NMR (75 MHz, CDCl_3_, δ, ppm): 116.3 (C-9a), 123.6 (CF_3_, *J* = 272 Hz), 124.1 (C-2), 125.2 (C-4′, *J*_C-F_ = 3.7 Hz), 126.2 (C-6′, *J*_C-F_ = 3.7 Hz), 126.6 (C-8), 127.4 (C-5), 129.3 (C-5′, *J*_C-F_ = 33.0 Hz), 129.6 (C-3′), 130.7 (C), 131.1 (C), 132.4 (C-10a), 133.7 (C), 134.0 (C-6), 134.8 (C-7), 136.5 (C-4a), 139.6 (C-3), 141.5 (C), 163.2 (C-1), 182.3 (C-10), 189.0 (C-9); IR (KBr, ν, cm^−1^): 3433 (OH), 1674 (C=O), 1629, 1593 (C=C); UV/Vis: λ_max_, nm (lgε): 255 (4.54), 328 (3.46), 404 (3.77); HR-MS (ESI), calcd. C_21_H_10_ClF_3_O_3_, *m*/*z* [M]^+^ 402.0265. found 402.0259.

1-(4-Chloro-2-(trifluoromethyl)phenyl)-4-hydroxyanthracene-9,10-dione (**19**).

Yield 52% (b). M.p.: 186.0 °C (decomp.); ^1^H NMR (500 MHz, CDCl_3_, δ, ppm): δ 7.14 (d, *J* = 8.30 Hz, 1H, H-6′), 7.31 (d, *J* = 8.80 Hz, 1H, H-2), 7.38 (d, *J* = 8.80 Hz, 1H, H-3), 7.56 (dd, *J* = 8.30, 2.1 Hz, 2H, H-5′), 7.70–7.827 (m, 3H, H-6,7,3′), 8.04 (m, 1H, H-5), 8.30 (m, 1H, H-8), 13.16 (s, 1H, OH) ppm; ^13^C NMR (125 MHz, CDCl_3_, δ, ppm): 116.3 (C-9a), 123.2 (CF_3_, *J* = 272.0 Hz), 123.3 (C-2), 126.4 (C-3′, *J*_C-F_ = 5.44 Hz), 126.6 (C-8), 127.4 (C-5), 129.0 (C-2′, *J*_C-F_ = 33.0 Hz), 130.7 (C), 131.1 (C), 131.4 (C-6′), 131.7 (C-5′), 132.5 (C-10a), 133.3 (C), 133.8 (C), 134.0 (C-6), 134.8 (C-7), 139.2 (C-1′, *J*_C-F_ = 1.75 Hz,), 139.6 (C-3), 163.2 (C-1), 182.3 (C-10), 189.0 (C-9); IR (KBr, ν, cm^−1^): 3441 (OH), 1676 (C=O), 1635, 1591 (C=C); UV/Vis: λ_max_, nm (lgε): 254 (4.50), 329 (3.43), 403 (3.74); HR-MS (ESI), calcd. C_21_H_10_ClF_3_O_3_, *m*/*z* [M]^+^ 402.0265. found 402.0261.

1-(Furan-2-yl)-4-hydroxyanthracene-9,10-dione (**22**).

Yield 84% (b). M.p.: 141.8 °C (decomp.); ^1^H NMR (300 MHz, CDCl_3_, δ, ppm): δ 6.56 (m, 2H, H-3′,4′), 7.28 (d, *J* = 8.77 Hz, 1H, H-2), 7.52 (m, 1H, H-5′), 7.70 (d, *J* = 8.77 Hz, 1H, H-3), 7.76 (m, 2H, H-6,7), 8.16 (m, 1H, H-5), 8.26 (m, 1H, H-8), 13.19 (s, 1H, OH); ^13^C NMR (75 MHz, CDCl_3_, δ, ppm): 108.3 (C-3′), 111.2 (C-4′), 116.5 (C-9a), 123.6 (C-2), 124.3 (C-4), 126.4 (C-8), 127.4 (C-5), 131.0 (C-4a), 132.2 (C-10a), 133.7 (C-6), 134.5 (C-8a), 134.8 (C-7), 140.1 (C-3), 142.3 (C-5′), 152.7 (C-2′), 163.2 (C-1), 182.2 (C-10), 189.0 (C-9); IR (KBr, ν, cm^-1^): 3435 (OH), 1676 (C=O), 1635, 1591 (C=C); UV/Vis: λ_max_, nm (lgε): 253 (4.58), 323 (3.80), 415 (3.57), 476 (3.55); HR-MS (ESI), calcd. C_18_H_10_O_4_, *m*/*z*: [M]^+^ 290.0574. found 290.0572.

1-(Furan-3-yl)-4-hydroxyanthracene-9,10-dione (**23**).

Yield 85%. M.p.: 190.0 °C (decomp.); ^1^H NMR (400 MHz, CDCl_3_, δ, ppm): δ = 6.45 (br.d, 1H, H-4′), 7.26 (d, *J* = 8.75 Hz, 1H, H-2), 7.48–7.55 (m, 3H, H-3,2′,5′), 7.76 (m, 2H, H-6,7), 8.15 (m, 1H, H-5), 8.26 (m, 1H, H-8), 13.21 (s, 1H, OH); ^13^C NMR (100 MHz, CDCl_3_, δ, ppm): 112.3 (C-4′), 116.6 (C-9a), 123.8 (C-2), 125.9 (C), 126.5 (C-8), 127.1 (C), 127.3 (C-5), 132.3 (C-10a), 130.4 (C-4a), 133.6 (C-6), 134.5 (C-8a), 134.8 (C-7), 139.7 (C-2′), 141.3 (C-3), 142.3 (C-5′), 162.8 (C-1), 182.8 (C-10), 189.0 (C-9); IR (KBr, ν, cm^-1^): 3423 (OH), 1668 (C=O), 1639, 1591 (C=C); UV/Vis: λ_max_, nm (lgε): 250 (4.47), 359 (3.53), 403 (3.69), 616 (3.26); HR-MS (ESI), calcd. C_18_H_10_O_4_, *m/z*: [M]^+^ 290.0574. found 290.0571.

1-Hydroxy-2-(3,4,5-trimethoxyphenyl)anthracene-9,10-dione (**24**).

Yield 86% (a), 93% (b). M.p.: 190.1 °C (decomp.); ^1^H NMR (400 MHz, CDCl_3_, δ, ppm): δ 3.90 (c, 3H, 4′-OCH_3_), 3.91 (c, 6H, 3′,5′-OCH_3_), 6.88 (s, 2H, H-2′,6′), 7.74 (d, *J* = 7.85 Hz, 1H, H-3), 7.82 (m, 2H, H-6,7), 7.89 (d, *J* = 7.85 Hz, 1H, H-4), 8.32 (m, 2H, H-5,8), 13.38 (s, 1H, OH); ^13^C NMR (125 MHz, CDCl_3_, δ, ppm): 56.1 (3′,5′-OCH_3_), 60.8 (4′-OCH_3_), 106.5 (C-2′,6′), 116.2 (C-9a), 119.5 (C-4), 127.0 (C-8), 127.3 (C-5), 131.2 (C-2), 132.1 (C-10a), 133.2 (C-1′), 133.6 (C-8a), 134.1 (C-7), 134.7 (C-6), 136.8 (C-4a), 137.0 (C-3), 138.1 (C-4′), 153.0 (C3′,5′), 160.0 (C-1), 182.1 (C-10), 189.1 (C-9); IR (KBr, ν, cm^−1^): 3430 (OH), 1670 (C=O), 1630, 1589 (C=C); UV/Vis: λ_max_, nm (lgε): 222 (4.46), 260 (4.50), 329 (3.43), 435 (3.87); HR-MS (ESI), calcd. C_23_H_18_O_6_, [M]^+^ *m*/*z*: 390.1098. found: 390.1095.

1-Hydroxy-2-phenylanthracene-9,10-dione (**25**).

Yield 74% (b). M.p.: 174.1 °C (decomp.); ^1^H NMR (500 MHz, CDCl_3_, δ, ppm): δ 7.41 (m, 1H, H-4′), 7.47 (m, 2H, H-3′,5′), 7.65 (m, 2H, H-2′,6′), 7.73 (d, *J* = 7.80 Hz, 1H, H-3), 7.80 (m, 2H, H-6,7), 7.89 (d, *J* = 7.80 Hz, 1H, H-4), 8.30 (m, 2H, H-5,8), 13.32 (s, 1H, OH); ^13^C NMR (125 MHz, CDCl_3_, δ, ppm): 116.1 (C-9a), 119.5 (C-4), 127.0 (C-8), 127.3 (C-5), 128.3 (C-2′,6′), 128.3 (C-4′), 129.1 (C3′,C5′), 132.1 (C-10a), 133.1 (C-2), 133.5 (C-8a), 134.1 (C-7), 134.6 (C-6), 135.8 (C-1′), 137.0 (C-4a), 137.2 (C-3), 160.1 (C-1), 182.1 (C-10), 189.0 (C-9); IR (KBr, ν, cm^−1^): 3435 (OH), 1672 (C=O), 1632, 1589 (C=C); UV/Vis: λ_max_, nm (lgε): 255 (4.62), 330 (3.49), 422 (3.92); HR-MS (ESI), calcd. C_20_H_12_O_3_, *m*/*z* [M]^+^ 299.0781. found 300.0701.

1-Hydroxy-2-(*o*-tolyl)anthracene-9,10-dione (**26**).

Yield 65% (b). M.p.: 148.5 °C (decomp.); ^1^H NMR (400 MHz, CDCl_3_, δ, ppm): δ 2.22 (s, 3H, CH_3_), 7.36–7.20 (m, 4H, H-3′,4′, 5′.6′), 7.59 (d, *J* = 7.70 Hz, 1H, H-3), 7.82 (m, 2H, H-6,7), 7.90 (d, *J* = 7.70 Hz, 1H, H-4), 8.32 (m, 2H, H-5,8), 13.03 (s, 1H, OH); ^13^C NMR (125 MHz, CDCl_3_, δ, ppm): 19.7 (CH_3_), 115.8 (C-9a), 119.1 (C-4), 125.7 (CH), 126.9 (C-8), 127.3 (C-5), 128.4 (CH), 129.5 (CH), 130.0 (CH), 132.5 (C-10a), 133.2 (C-2), 133.6 (C-8a), 134.1 (C-6), 134.6 (C-7), 135.8 (C-1′), 136.5 (C-4a), 137.8 (C-3), 137.9 (C-2′), 159.9 (C-1), 182.2 (C-10), 188.9 (C-9); IR (KBr, ν, cm^−1^): 3440 (OH), 1666 (C=O), 1628, 1591 (C=C); UV/Vis: λ_max_, nm (lgε): 253 (4.55), 331 (3.48), 413 (3.89); HR-MS (ESI), calcd. C_21_H_14_O_3_, *m*/*z* [M]^+^ 314.0938. found 314.0937.

1-Hydroxy-2-(4-methoxyphenyl)anthracene-9,10-dione (**27**).

Yield 68% (b). M.p.: 209.5 °C (decomp.); ^1^H NMR (500 MHz, CDCl_3_, δ, ppm): δ 3.85 (s, 3H, OCH_3_), 7.00 (d, *J* = 8.75 Hz, 2H, H-3′,5′), 7.62 (d, *J* = 8.70 Hz, 2H, H-2′,6′), 7.71 (d, *J* = 7.85 Hz, 1H, H-3), 7.80 (m, 2H, H-6,7), 7.88 (d, *J* = 7.85 Hz, 1H, H-4), 8.31 (m, 2H, H-5,8), 13.36 (s, 1H, OH); ^13^C NMR (125 MHz, CDCl_3_, δ, ppm): 55.2 (OCH_3_), 113.7 (C-3′,5′), 116.1 (C-9a), 119.6 (C-4), 126.9 (C-8), 127.2 (C-5), 128.1 (C-1′), 130.5 (C-2′,6′), 131.7 (C-2), 133.2 (2C-8a,10a), 133.6 (C-4a), 134.0 (C-6), 134.7 (C-7), 136.7 (C-3), 159.7 (C-4′), 160.1 (C-1), 182.1 (C-10), 189.0 (C-9); IR (KBr, ν, cm^−1^): 3430 (OH), 1664 (C=O), 1641, 1606 (C=C); UV/Vis: λ_max_, nm (lgε): 261 (4.59), 297 (4.06), 440 (3.96); HR-MS (ESI), calcd. C_21_H_14_O_4_, *m*/*z* [M]^+^ 330.0887. found 330.0889.

2-(2,3-Dimethoxyphenyl)-1-hydroxyanthracene-9,10-dione (**28**).

Yield 78% (b). M.p.: 176.3 °C (decomp.); ^1^H NMR (400 MHz, CDCl_3_, δ, ppm): δ = 3.70 (s, 3H, 3′-OCH_3_), 3.90 (s, 3H, 2′-OCH_3_), 6.93 (dd, *J* = 7.85, 1.20 Hz, 1H, H-4′), 6.99 (dd, *J* = 7.85, 1.20 Hz, 1H, H-6′), 7.14 (t, *J* = 7.85 Hz, 1H, H-5′), 7.68 (d, *J* = 7.72 Hz, 1H, H-3), 7.80 (m, 2H, H-6,7), 7.88 (d, *J* = 7.75 Hz, 1H, H-4), 8.31 (m, 2H, H-5,8), 13.08 (s, 1H, OH); ^13^C NMR (100 MHz, CDCl_3_, δ, ppm): 55.8 (3′-OCH_3_), 60.7 (2′-OCH_3_), 112.7 (C-4′), 115.9 (C-9a), 118.9 (C-4), 122.5 (C-5′), 123.7 (C-6′), 126.9 (C-8), 127.3 (C-5), 130.1 (C), 132.5 (C-10a), 133.3 (C), 133.6 (C), 134.0 (C-6), 134.5 (C-4a), 134.5 (C-7), 138.3 (C-3), 146.8 (C-3′), 152.8 (C-2′), 160.4 (C-1), 182.2 (C-10), 188.8 (C-9); IR (KBr, ν, cm^−1^): 3464 (OH), 1672 (C=O), 1633, 1591 (C=C); UV/Vis: λ_max_, nm (lgε): 222 (4.86), 253 (4.95), 416 (4.28); HR-MS (ESI), calcd. C_22_H_16_O_5_, *m*/*z* [M]^+^ 360.0992. found 360.0995.

2-(3,5-Difluorophenyl)-1-hydroxyanthracene-9,10-dione (**29**).

Yield 56% (b). M.p.: 264.3 °C (decomp.); ^1^H NMR (500 MHz, CDCl_3_, δ, ppm): δ = 6.86 (t t, *J* = 8.90, 2.3 Hz, 1H, H-4‘), 7.18–7.26 (m, 2H, 2H, H-2′,6′), 7.73 (d, *J* = 7.90 Hz, 1H, H-3), 7.83 (m, 2H, H-6,7), 7.91 (d, *J* = 7.90 Hz, 1H, H-4), 8.33 (m, 2H, H-5,8), 13.37 (s, 1H, OH) ppm; ^13^C NMR (125 MHz, CDCl_3_, δ, ppm): 103.6 (t, C-4′, *J*_C-F_ = 25.2 Hz), 112.2 (d, C-2′, *J*_C-F_ = 6.4 Hz), 112.4 (d, C-6′, *J*_C-F_ = 6.4 Hz), 116.4 (C-9a), 119.4 (C-4), 127.0 (C-8), 127.4 (C-5), 133.1 (C-10a), 133.1 (C-8a), 133.5 (C-4a), 134.2 (t, C-2, *J*_C-F_ = 2.3 Hz), 134.2 (C-6), 134.8 (C-7), 136.9 (C-3), 138.8 (C-1′, *J*_C-F_ = 10.1 Hz), 159.9 (C-1), 162.7 (C-3′, *J*_C-F_ 249.9 Hz), 162.8 (C-5′, *J*_C-F ‘_, *J* = 249.9 Hz), 181.9 (C-10), 189.0 (C-9); IR (KBr, ν, cm^−1^): 3444 (OH), 1670 (C=O), 1643, 1593 (C=C); UV/Vis: λ_max_, nm (lgε): 255 (4.65), 333 (3.53), 416 (3.92); HR-MS (ESI), calcd. C_20_H_10_F_2_O_3_, *m/z* [M]^+^ 336.0593. found 336.0583.

2-(2-Chloro-5-(trifluoromethyl)phenyl)-1-hydroxyanthracene-9,10-dione (**30**).

Yield 52% (b). M.p.: 188.5 °C (decomp.); ^1^H NMR (500 MHz, CDCl_3_, δ, ppm): δ = 7.58–7.68 (m, 4H, H-3, 3′,4′,6′), 7.83 (m, 2H, H-6,7), 7.92 (d, *J* = 7.76 Hz, 1H, H-4), 8.32 (m, 2H, H-5,8), 13.03 (s, 1H, OH); ^13^C NMR (125 MHz, CDCl_3_, δ, ppm): 116.2 (C-9a), 118.8 (C-4), 123.4 (CF_3_, *J* = 272.0 Hz), 126.2 (CH, *J* = 3.83 Hz), 126.9 (C-8), 127.3 (C-5), 128.2 (CH, *J* = 3.81 Hz), 129.1 (C-5′, *J*_C-F_ = 33.0 Hz), 130.1 (C-3′), 133.0 (C), 133.2 (C), 133.4 (C), 133.4 (C), 134.1 (C-6), 134.7 (C-7), 135.7 (C), 137.3 (C-1′, *J*_C-F_ = 1.65 Hz), 137.7 (C-3), 159.8 (C-1), 181.9 (C-10), 188.7 (C-9); IR (KBr, ν, cm^−1^): 3421 (OH), 1666 (C=O), 1632, 1589 (C=C); UV/Vis: λ_max_, nm (lgε): 253 (4.78), 333 (3.64), 407 (4.05); HR-MS (ESI), calcd. C_21_H_10_ClF_3_O_3_, *m/z* [M]^+^ 402.0265. found 402.0267.

2-(4-Chloro-2-(trifluoromethyl)phenyl)-1-hydroxyanthracene-9,10-dione (**31**).

Yield 71% (b). M.p.: 189–191 °C; ^1^H NMR (400 MHz, CDCl_3_, δ, ppm): δ 7.32 (d, *J* = 8.30 Hz, 1H, H-6′), 7.56 (d, *J* = 7.80 Hz, 1H, H-3), 7.60 (dd, *J* = 8.30, 1.95 Hz, 1H, H-5′), 7.78 (d, *J* = 1.95 Hz, 1H, H-3′), 7.82 (m, 2H, H-6,7), 7.87 (d, *J* = 7.80 Hz, 1H, H-4), 8.31 (m, 2H, H-5,8), 12.93 (s, 1H, OH) ppm; ^13^C NMR (100 MHz, CDCl_3_): 115.9 (C-9a), 118.5 (C-4), 123.0 (CF_3_, *J* = 274.0 Hz), 126.7 (C-3′), 127.0 (C-8), 127.4 (C-5), 130.7 (C2′, *J*_C-F_ = 31.0 Hz), 131.6 (C-6′), 133.0 (C), 133.1 (C-5′), 133.3 (C), 133.5 (C), 133.7 (C), 134.2 (C-6), 134.6 (C), 134.8 (C-7), 137.4 (C-3), 160.0 (C-1), 182.1 (C-10), 188.8 (C-9); IR (KBr, ν, cm^−1^): 3442 (OH), 1670 (C=O), 1632, 1589 (C=C); UV/Vis: λ_max_, nm (lgε): 225 (4.40), 253 (4.56), 333 (3.48), 406 (3.86); HRMS, calcd. C_21_H_10_ClF_3_O_3_, *m/z* [M]^+^ 402.0265. found 402.0268.

1-Hydroxy-2,4-di-(3,4,5-trimethoxyphenyl)anthracene-9,10-dione (**32**).

Yield 67% (a), 45% (b); 90% (c). M.p.: 214.9 °C (decomp.); ^1^H NMR (400 MHz, CDCl_3_, δ, ppm): δ 3.84 (s, 6H, 3′′,5′′-OCH_3_), 3.89 (s, 3H, 4′-OCH_3_), 3.90 (s, 6H, 3′,5′-OCH_3_), 3.93 (s, 3H, 4′′-OCH_3_), 6.49 (s, 2H, H-2′′,6′′), 6.90 (s, 2H, H-2′,6′), 7.58 (s, 1H, H-3), 7.79 (m, 2H, H-6,7), 8.13 (m, 1H, H-5), 8.33 (m, 1H, H-8), 13.99 (s, 1H, OH); ^13^C NMR (100 MHz, CDCl_3_, δ, ppm): 56.0, 56.2 (4C, 3′,5′,3′′,5′′-OCH_3_), 60.8, 60.9 (2C, 4′,4′′-OCH_3_), 105.2 (C-2′′,6′′), 106.6 (C-2′,6′), 116.6 (C-9a), 126.6 (C-8), 127.3 (C-5), 128.9 (C-4), 130.8 (C-2), 132.5 (C-10a), 133.7 (C-7), 134.5 (C-8a), 134.9 (C-6), 135.9 (C-1′), 136.4 (C-4a), 137.1 (C-4′), 137.4 (C-1′′), 138.4 (C4′′), 140.6 (C3), 153.0 (C3′,5′), 153.1 (C3′′,5′′), 160.2 (C-1), 182.3 (C-10), 189.6 (C-9); IR (KBr, ν, cm^−1^): 3435 (OH), 1671 (C=O), 1629, 1582 (C=C); UV/Vis: λ_max_, nm (lgε): 258 (4.15), 338 (3.14), 432 (3.47); HR-MS (ESI), calcd. C_32_H_28_O_9_, *m/z* [M]^+^ 556.1728. found 556.1723.

1-Hydroxy-2,4-diphenylanthracene-9,10-dione (**33**).

Yield 67% (a); 90% (c). M.p.: 225-228 °C; ^1^H NMR (500 MHz, CDCl_3_, δ, ppm): δ 7.29 (d, *J* = 7.04 Hz, 2H, H-2′,6′), 7.37–7.51 (m, 6H, H-3′,4′,5′,3′′,4′′,5′′), 7.58 (s, 1H, H-3), 7.68 (d, *J* = 7.27 Hz, 2H, H-2′′,6′′), 7.76 (m, 2H, H-6,7), 8.11 (m, 1H, H-5), 8.32 (m, 1H, H-8), 13.94 (s, 1H, OH); ^13^C NMR (125 MHz, CDCl_3_, δ, ppm): 116.5 (C-9a) 126.5 (C-8), 127.0 (CH), 127.3 (C-5), 128.0 (2CH), 128.1 (2CH), 128.3 (2CH), 128.4 (CH), 128.8 (C-4), 129.2 (2CH), 132.6 (C-10a), 133.6 (C-6), 134.5 (C-8a), 134.8 (C-7), 135.4 (C-1′), 136.1 (C), 136.7 (C-4a), 141.1 (C-3), 141.9 (C-1′′), 160.3 (C-1), 182.5 (C-10), 189.6 (C-9); IR (KBr, ν, cm^−1^): 3435 (OH), 1672 (C=O), 1630, 1589 (C=C); UV/Vis: λ_max_, nm (lgε): 259 (4.62),429 (3.65); HRMS, calcd. C_26_H_16_O_3_, *m/z* [M]^+^ 376.1094. found 376.1068.

1-Hydroxy-2,4-di-*o*-tolylanthracene-9,10-dione (**34**).

Yield 60% (c). M.p.: 119.8 °C (decomp.); ^1^H NMR (500 MHz, CDCl_3_, δ, ppm): 2.09 (s, 3H, 2′′-CH_3_), 2.27 (s, 3H, 2′-CH_3_), 7.09 (d, *J* = 7.30 Hz, 1H, H-3′′), 7.25-7.33 (m, 7H, H-3′,4′,5′,6′,4′′,5′′,6′′), 7.35 (s, 1H, H-3), 7.76 (m, 2H, H-6,7), 8.12 (m, 1H, H-5), 8.32 (m, 1H, H-8), 13.65 (s, 1H, OH); ^13^C NMR (125 MHz, CDCl_3_, δ, ppm): 19.8 (CH_3_), 19.9 (CH_3_), 116.1 (C-9a), 125.6 (C), 125.7 (C), 126.4 (C-8), 127.1 (C), 127.3 (C-5), 127.4 (C), 128.3 (C), 129.1 (C-4), 129.5 (C), 129.5 (C), 129.9 (C), 132.6 (C-10a), 133.6 (C-6), 134.2 (C-8a), 134.6 (C7), 134.8 (C), 135.4 (C-1′), 136.3 (C-4a), 137.4 (C), 141.3 (C-3), 141.4 (C-1′′), 160.1 (C-1), 182.4 (C-10), 189.5 (C-9); IR (KBr, ν, cm^−1^): 3439 (OH), 1670 (C=O), 1628, 1591 (C=C); UV/Vis: λ_max_, nm (lgε): 255 (4.55), 325 (3.52), 416 (3.83); HRMS, calcd. C_28_H_20_O_3_, *m/z* [M]^+^ 404.1407. found 404.1408.

1-Hydroxy-2,4-di-(4-methoxyphenyl)anthracene-9,10-dione (**35**).

Yield: 40% (b), 93% (c), 27% (d), 22% (e). M.p.: 226.4 °C (decomp.); ^1^H NMR (400 MHz, CDCl_3_, δ, ppm): δ 3.85 (s, 3H, 4′-OCH_3_), 3.86 (s, 3H, 4′′-OCH_3_), 6.95-7.00 (m, 4H, H-3′,5′,3′′,5′′), 7.22 (d, *J* = 8.88 Hz, 2H, H-2′,6′), 7.54 (s, 1H, H-3), 7.65 (d, *J* = 8.88 Hz, 2H, H-2′′,6′′), 7.75 (m, 2H, H-6,7), 8.12 (m, 1H, H-5), 8.31 (m, 1H, H-8), 14.00 (s, 1H, OH); ^13^C NMR (125 MHz, CDCl_3_, δ, ppm): 55.1 (4′′-OCH_3_), 55.3 (4′-OCH_3_), 113.6 (C-3′′,5′′), 113.7 (C3′,5′), 116.5 (C-9a), 126.5 (C-8), 127.3 (C-5), 127.8 (C), 128.3 (C), 129.3 (C-2′′,6′′), 130.5 (C-2′,6′), 132.6 (C-10a), 133.5 (C-6), 134.1 (C), 134.6 (C-8a), 134.7 (C-7), 135.8 (C), 136.5 (C-4a), 141.0 (C-3), 158.6 (C-4′′), 159.8 (C-4′), 160.3 (C-1), 182.6 (C-10), 189.6 (C-9); IR (KBr, ν, cm^-1^): 3442 (OH), 1668 (C=O), 1630, 1606 (C=C); UV/Vis: λ_max_, nm (lgε): 258 (4.61), 317 (3.06), 440 (3.92); HRMS, calcd. C_28_H_20_O_5_, *m/z* [M]^+^ 436.1305. found 436.1229.

2,4-Di-(2,3-dimethoxyphenyl)-1-hydroxyanthracene-9,10-dione (**36**).

Yield 78% (c). M.p.: 192.9 °C (decomp.); ^1^H NMR (400 MHz, CDCl_3_, δ, ppm): δ 3.55 (s, 3H, 3′′-OCH_3_), 3.71 (s, 3H, 3′-OCH_3_), 3.89 (s, 3H, 2′′-OCH_3_), 3.90 (s, 3H, 2′-OCH_3_), 6.79 (dd, *J* = 7.75, 1.50 Hz, 1H, H-4′′), 6.93-7.00 (m, 3H, H-4′,6′,6′′), 7.08-7.15 (m, 2H, H-5′,5′′), 7.52 (s, 1H, H-3), 7.74 (m, 2H, H-6,7), 8.12 (m, 1H, H-5), 8.30 (m, 1H, H-8), 13.65 (s, 1H, OH); ^13^C NMR (100 MHz, CDCl_3_, δ, ppm): δ = 55.6 (3′′-OCH_3_), 55.8 (3′-OCH_3_), 60.4 (2′′-OCH_3_), 60.8 (2′-OCH_3_), 111.5 (C-4′′), 112.7 (C-4′), 116.2 (C-9a), 121.0 (C-5′′), 122.6 (C-5′), 123.7 (C-6′), 124.0 (C-6′′), 126.4 (C-8), 127.4 (C-5), 129.9 (C), 130.0 (C), 131.4 (C), 132.7 (C-10a), 133.3 (C), 133.5 (C-6), 134.4 (C-8a), 134.5 (C-7), 136.3 (C-4a), 142.1 (C-3), 145.5 (C-3′), 146.8 (C-3′′), 152.4 (C-2′), 152.8 (C-2′′), 160.5 (C-1), 182.5 (C-10), 189.5 (C-9); IR (KBr, ν, cm^−1^): 3442 (OH), 1672 (C=O), 1633, 1579 (C=C); UV/Vis: λ_max_, nm (lgε): 255 (4.53), 316 (3.12), 423 (3.77); HRMS, calcd. C_30_H_24_O_7_, *m/z* [M]^+^ 496.1517. found 496.1514.

2,4-Di-(3,5-difluorophenyl)-1-hydroxyanthracene-9,10-dione (**37**).

Yield 49% (c). M.p.: 263.3 °C (decomp.); ^1^H NMR (500 MHz, CDCl_3_, δ, ppm): δ 6.79 (m, 2H, H-2′′,6′′), 6.82-6.90 (m, 2H, H-4′,4′′), 7.22 (m, 2H, H-2‘,6‘), 7.50 (s, 1H, H-3), 7.80 (m, 2H, H-6,7), 8.11 (m, 1H, H-5), 8.33 (m, 1H, H-8), 13.93 (s, 1H, OH); ^13^C NMR (125 MHz, CDCl_3_, δ, ppm): 102.7 (C4′′, *J*_C-F_ = 25.2 Hz), 103.9 (C4′, *J*_C-F_ = 25.2 Hz), 111.2 (C2′′, *J*_C-F_ = 6.42 Hz), 111.4 (C6′′, *J*_C-F_ = 6.42 Hz), 112.3 (C2′, *J*_C-F_ = 6.42 Hz), 112.4 (C6′, *J*_C-F_ = 6.42 Hz), 116.9 (C-9a), 126.8 (C-8), 127.4 (C-5), 129.9 (C), 132.4 (C), 133.6 (C), 133.8 (C), 134.1 (C), 134.1 (C-6), 135.1 (C-7), 138.1 (C-1′, *J*_C-F_ = 10.0 Hz), 139.7 (C-3), 144.6 (C-1′′, *J*_C-F_ = 10.0 Hz), 160.4 (C-1), 162.7 (C3′,5′, *J*_C-F_ = 248 Hz), 162.8 (C-3′′,5′′, *J*_C-F_ = 248 Hz), 182.0 (C-10), 189.4 (C-9); IR (KBr, ν, cm^−1^): 3444 (OH), 1666 (C=O), 1624, 1593 (C=C); UV/Vis: λ_max_, nm (lgε): 257 (5.06), 417 (4.30); HRMS, calcd. C_26_H_12_F_4_O_3_, *m/z* [M]^+^ 448.0717. found 448.0711.

2,4-Di-(2-chloro-5-(trifluoromethyl)phenyl)-1-hydroxyanthracene-9,10-dione (**38**).

Yield 93% (c).M.p.: 154-157 °C; ^1^H NMR (500 MHz, CDCl_3_, δ, ppm): δ 7.41 (s, 1H, H-6′), 7.65-7.58 (m, 4H, H-3′,4′,3′′,4′′), 7.70 (s, 1H, H-6′′), 7.80 (m, 2H, H-6,7), 8.12 (m, 1H, H-5), 8.34 (m, 1H, H-8), 13.62 (s, 1H, OH); ^13^C NMR (125 MHz, CDCl_3_, δ, ppm): 116.5 (C-9a), 123.4 (CF_3_, *J* = 272.0 Hz), 123.6 (CF_3_, *J* = 272.0 Hz), 125.3 (C-6′′), 126.2 (C-6′), 126.4 (C-4′′), 126.7 (C-8), 127.4 (C-5), 128.2 (C-4′), 129.2, 129.3 (C5′, C5′′, *J* = 33.0 Hz), 129.6 (C-3′′), 130.2 (C-3′), 130.5, 130.8, 132.4, 133.0, 133.7 (al C), 134.1 (C-6), 134.9 (C-7), 135.0 (C), 136.5 (C-4a), 137.3 (C-1′), 140.5 (C-3), 141.0 (C-1′′), 160.5 (C-1), 181.9 (C-10), 189.1 (C-9); IR (KBr, ν, cm^−1^): 3435 (OH), 3442 (O-H), 1674 (C=O), 1639, 1593 (C=C); UV/Vis: λ_max_, nm (lgε): 256 (4.56), 406 (3.81); HRMS, calcd. C_28_H_12_Cl_2_F_6_O_3_, *m/z* [M]^+^ 580.0062. found 580.0069.

2,4-Di-(4-Chloro-2-(trifluoromethyl)phenyl)-1-hydroxyanthracene-9,10-dione (**39**).

Yield 47% (b). M.p.: 150-152 °C; ^1^H NMR (500 MHz, CD_3_CN, δ, ppm): δ 7.26 (d, *J* = 8.20 Hz, 1H, H-6′), 7.41 (d, *J* = 1.95 Hz, 1H, H-3′′), 7.48 (d, *J* = 8.35 Hz, 1H, H-6′′), 7.66 (dt, *J* = 8.35, 1.92 Hz, 1H, H-5′′), 7.71 (dd, *J* = 8.20, 2.1 Hz, 2H, H-5′),7.79-7.91 (m, 4H, H-3,6,7,3′), 8.01 (m, 1H, H-5), 8.30 (m, 1H, H-8), 13.40 (s, 1H, OH); ^13^C NMR (125 MHz, CDCl_3_, δ, ppm): 116.1 (C-9a), 121.8 (CF_3_, *J* = 270.0 Hz), 124.0 (CF_3_, *J* = 273.0 Hz), 126.3 (C-3′′, *J*_C-F_ = 5.44 Hz), 126.7 (C-3′, *J*_C-F_ = 5.12 Hz), 126.7 (C-8), 127.4 (C-5), 128.8 (C-2′′, *J*_C-F_ = 32.0 Hz), 129.3 (C-2′, *J*_C-F_ = 30.4 Hz), 131.3 (C-6′′), 131.6 (C-6′), 131.8 (C), 132.3 (C-5′′), 132.5 (C-10a), 132.7 (C), 133.0 (C-5′), 134.3 (C-1′, *J*_C-F_ = 1.65 Hz), 133.3 (C), 133.7 (C), 134.6 (C), 134.1 (C-6), 134.8 (C), 135.1 (C-7), 138.6 (C-1′′, *J*_C-F_ = 2.15 Hz,), 140.8 (C-3), 161.1 (C-1), 182.8 (C-10), 189.5 (C-9); IR (KBr, ν, cm^−1^): 3434 (OH), 1673 (C=O), 1633, 1592 (C=C); UV/Vis: λ_max_, nm (lgε): 229 (4.28), 260 (4.63), 412 (3.92); HRMS, calcd. C_28_H_12_Cl_2_F_6_O_3_, *m/z* [M]^+^ 580.0062. found 580.0065.

4-Bromo-1-hydroxy-2-(4-methoxyphenyl)anthracene-9,10-dione (**40**).

Yield 39% (b), 30% (d), 35% (e). M.p.: 187.1 °C (decomp.); ^1^H NMR (600 MHz, CDCl_3_, δ, ppm): δ = 3.85 (s, 3H, 4′-OCH_3_), 7.00 (d, *J* = 8.80 Hz, 2H, H-3′,5′), 7.62 (d, *J* = 8.80 Hz, 2H, H-2′,6′), 7.75-7.86 (m, 2H, H-6,7), 7.94 (s, 1H, H-3), 8.30 (m, 2H, H-5,8), 14.09 (s, 1H, OH); ^13^C NMR (150 MHz, CDCl_3_, δ, ppm): δ 55.3 (OCH_3_), 113.4 (C-4), 113.9 (C-3′,5′), 117.6 (C-9a), 126.6 (C-8), 126.7 (C-4a), 127.6 (C-5), 128.1 (C-2), 130.6 (C-2′,6′), 132.2 (C-8a), 133.8 (C-6), 134.1 (C-10a), 135.0 (C-7), 137.6 (C-1′), 143.2 (C-3), 160.2 (C-4′), 160.8 (C-1), 180.9 (C-10), 188.5 (C-9); IR (KBr, ν, cm^−1^): 3431 (OH), 1670 (C=O), 1620, 1592 (C=C); UV/Vis: λ_max_, nm (lgε): 262 (4.88), 443 (3.75); HRMS, calcd. C_21_H_13_BrO_4_, *m/z* [M]^+^ 407.9992. found 407.9995.

2-Bromo-1-hydroxy-4-(4-methoxyphenyl)anthracene-9,10-dione (**41**).

Yield 8% (b), 29% (d), 26% (e). M.p.: 241.3 °C (decomp.); ^1^H NMR (500 MHz, CDCl_3_, δ, ppm): δ 3.86 (s, 3H, OCH_3_), 6.96 (d, *J* = 8.60 Hz, 2H, H-3′,5′), 7.17 (d, *J* = 8.60 Hz, 2H, H-2′,6′), 7.74-7.82 (m, 2H, H-3,6,7), 8.10 (m, 1H, H-5), 8.29 (m, 1H, H-8), 13.93 (s, 1H, OH); ^13^C NMR (150 MHz, CDCl_3_, δ, ppm): δ 55.2 (OCH_3_), 113.7 (C-3′,C5′), 116.8 (C-2), 118.1 (C-9a), 126.7 (C-8), 127.5 (C-5), 129.1 (C-4), 129.2 (C-2′,6′), 132.0 (C-10a), 132.8 (C-4a), 133.8 (C-6), 134.5 (C-8a), 135.1 (C-7), 137.1 (C-1′), 143.8 (C-3), 159.0 (C-4′), 159.0 (C-1), 182.3 (C-10), 189.1 (C-9); IR (KBr, ν, cm^−1^): 3441 (OH), 1674 (C=O), 1637, 1600 (C=C); UV/Vis: λ_max_, nm (lgε): 228 (4.28), 256 (4.49), 321 (3.66), 415 (3.66); HRMS, calcd. C_21_H_13_BrO_4_, *m/z* [M]^+^ 407.9992. found 407.9927.

4-Bromo-1-hydroxy-2-(3,4,5-trimethoxyphenyl)anthracene-9,10-dione (**42**).

Yield 24% (b). M.p.: 217.4 °C (decomp.); ^1^H NMR (400 MHz, CDCl_3_, δ, ppm): δ 3.90 (s, 3H, 4′-OCH_3_), 3.91 (s, 6H, 3′,5′-OCH_3_), 6.86 (s, 2H, H-2′,6′), 7.82 (m, 2H, H-6,7), 7.96 (s, 1H, H-3), 8.31 (d, *J* = 7.71 Hz, 2H, H-5,8), 14.09 (s, 1H, OH); ^13^C NMR (100 MHz, CDCl_3_, δ, ppm): δ = 56.2 (3′,5′-OCH_3_), 60.9 (4′-OCH_3_), 106.5 (C-2′,6′), 113.2 (C-4), 117.7 (C-9a), 126.6 (C-8), 127.6 (C-5), 128.5 (C-2), 129.7 (C-4a), 132.0 (C-8a), 133.9 (C-6), 134.0 (C-10a), 135.1 (C-1′), 137.7 (C-7), 138.6 (C-4′), 143.5 (C-3), 153.1 (C-3′,5′), 160.6 (C-1), 180.8 (C-10), 188.5 (C-9); IR (KBr, ν, cm^−1^): 3439 (OH), 1673 (C=O), 1625, 1588 (C=C); UV/Vis: λ_max_, nm (lgε): 261 (4.08), 442 (3.56); HRMS, calcd. C_23_H_17_BrO_6_, *m/z* [M]^+^ 468.0203. found 468.0198.

1-Hydroxy-4-phenyl-2-(3,4,5-trimethoxyphenyl)anthracene-9,10-dione (**43**).

Yield 68% (b). M.p.: 60.4 °C (decomp.); ^1^H NMR (500 MHz, CDCl_3_, δ, ppm): δ 3.89 (s, 9H, 3-OCH_3_), 6.89 (s, 2H, H-2′,6′), 7.30 (m, 2H, H-3′′,5′′), 7.44 (m, 3H, H-2′′,4′′,6′′), 7.57 (s, 1H, H-3), 7.77 (m, 2H, H-6,7), 8.11 (m, 1H, H-5), 8.32 (m, 1H, H-8), 14.00 (s, 1H, OH); ^13^C NMR (125 MHz, CDCl_3_, δ, ppm): δ = 56.1 (3′,5′-OCH_3_), 60.8 (4′-OCH_3_), 106.5 (C-2′,6′), 116.5 (C-9a), 126.5 (C-4′′), 127.00 (C-8), 127.3 (C-5), 128.0 (C-2′′,6′′), 128.1 (C-3′′,5′′), 128.7 (C), 130.8 (C), 132.5 (C-10a), 133.6 (C-6), 134.4 (C), 134.7 (C-7), 135.9 (C-1′), 136.5 (C-4a), 138.2 (C-4′), 140.6 (C-3), 141.8 (C), 152.9 (C-3′,5′), 160.1 (C-1), 182.3 (C-10), 189.6 (C-9); IR (KBr, ν, cm^−1^): 3435 (OH), 1670 (C=O), 1628, 1585 (C=C); UV/Vis: λ_max_, nm (lgε): 254 (4.51), 437 (3.88); HRMS, calcd. C_29_H_22_O_6_, *m/z* [M]^+^ 466.1411. found 466.1416.

1-Hydroxy-4-(4-methoxyphenyl)-2-(3,4,5-trimethoxyphenyl)anthracene-9,10-dione (**44**)

Yield 89% (b). M.p.: 182-184 °C; ^1^H NMR (500 MHz, CDCl_3_, δ, ppm): δ = 3.86 (s, 3H, 4′′-OCH_3_), 3.89 (s, 9H, 3′,4′,5′-OCH_3_), 6.89 (s, 2H, H-2′,6′), 6.98 (d, *J* = 8.65 Hz, 2H, H-3′′,5′′), 7.24 (d, *J* = 8.65 Hz, 2H, H-2′′,6′′), 7.56 (s, 1H, H-3), 7.77 (m, 2H, H-6,7), 8.13 (m, 1H, H-5), 8.32 (m, 1H, H-8), 14.01 (s, 1H, OH); ^13^C NMR (125 MHz, CDCl_3_, δ, ppm): δ 55.1 (4′′-OCH_3_), 56.2 (3′,5′-OCH_3_), 60.9 (4′-OCH_3_), 106.6 (C-2′,6′), 113.6 (C-3′′,5′′), 116.7 (C-9a), 126.5 (C-8), 127.3 (C-5), 128.7 (C-4), 129.3 (C-2′′,6′′), 130.9 (C), 132.5 (C), 133.6 (C-6), 133.9 (C), 134.6 (C), 134.8 (C-7), 136.0 (C-1′), 136.4 (C-4a), 138.3 (C-4′), 141.1 (C3), 153.0 (C3‘,C5‘), 158.7 (C-4′′), 160.1 (C-1), 182.6 (C-10), 189.6 (C-9); IR (KBr, ν, cm^−1^): 3445 (OH), 1666 (C=O), 1624, 1587 (C=C); UV/Vis: λ_max_, nm (lgε): 224 (4.12), 257 (4.16), 435 (3.45); HRMS, calcd. C_30_H_24_O_7_, *m/z* [M]^+^ 496.1517. found 496.1515.

4-(4-Chloro-2-(trifluoromethyl)phenyl)-1-hydroxy-2-(3,4,5-trimethoxyphenyl)-anthracene-9,10-dione (**45**).

Yield 57% (b). M.p.: 226.8 °C (decomp.); ^1^H NMR (400 MHz, CDCl_3_, δ, ppm): δ 3.89 (s, 9H, 3-OCH_3_), 6.87 (s, 2H, H-2′,6′), 7.21 (d, *J* = 8.00 Hz, 1H, H-6′′), 7.47 (s, 1H, H-3), 7.58 (dd, *J* = 8.00, 2.00 Hz, 1H, H-5′′), 7.78 (m, 3H, H-6,7,3′′), 8.07 (m, 1H, H-5), 8.34 (m, 1H, H-8), 13.97 (s, 1H, OH); ^13^C NMR (100 MHz, CDCl_3_, δ, ppm): 56.1 (3′,5′-OCH_3_), 60.8 (4′-OCH_3_), 106.6 (C-2′,6′), 116.4 (C-9a), 123.2 (CF_3_, *J* = 274.0 Hz), 126.5 (C-3′′), 126.7 (C-8), 127.3 (C-5), 128.9 (C-2′′, *J*_C-F_ = 30.0 Hz), 129.3 (C), 130.5 (C), 131.1 (C), 131.4 (C-6′′), 131.7 (C-5′′), 132.5 (C), 133.3 (C), 133.8 (C), 134.0 (C-6), 134.9 (C-7), 135.7 (C), 138.4 (C), 139.1 (C-1′′, *J*_C-F_ = 1.65 Hz), 139.7 (C-3), 153.0 (C-3′,5′), 160.7 (C-1), 182.1 (C-10), 189.5 (C-9); IR (KBr, ν, cm^−1^): 3434 (OH), 1670 (C=O), 1626, 1585 (C=C); UV/Vis: λ_max_, nm (lgε): 224 (4.24), 261 (4.19), 432 (3.56); HR-MS (ESI), calcd. C_30_H_20_ClF_3_O_6_, *m*/*z* [M]^+^ 568.0895. found 568.0897.

### 4.3. Molecular Docking Study

Molecular modeling was carried out in the *Schrodinger Maestro* visualization environment using applications from the Schrödinger Small Molecule Drug Discovery Suite 2016-1 package [59]. Three-dimensional structures of the derivatives were obtained empirically in the *LigPrep* application using the OPLS3 force field [60]. For the calculations, the XRD model of topoisomerase IIβ-DNA complex inhibited by mitoxantrone from Protein Data Bank was chosen (PDB ID 4G0V) [61]. To model a possible mechanism of inhibition of selected target, molecular docking of new compounds was performed at the binding site of topoisomerase IIβ-DNA complex using *Glide* [62]. The search area for docking was selected according to the size of inhibitor. Docking was performed in comparison with mitoxantrone and doxorubicin. The three-dimensional structures of inhibitors were obtained in the PubChem database and prepared in the *LigPrep* application. Non-covalent interactions of molecules in the binding site were visualized using Biovia Discovery Studio Vizualizer.

### 4.4. Biological Evaluation

#### 4.4.1. Cell Culture and Determination of Cytotoxicity

The human cancer cells of the glioblastoma (U-87MG, SNB-19, T98G), prostate cancer cell line (LNCAP, DU-145), the cells of T-cellular human leucosis (MT-4) and human breast cancer cells (MDA-MB-231) were used in this study. The cells were cultured in the RPMI-1640 medium that contained 10% embryonic calf serum, L-glutamine (2 mmol/L), gentamicin (80 mg/mL) and lincomycin (30 mkg/mL) in a CO_2_ incubator at 37 °C. The tested compounds were dissolved in DMSO and added to the cellular culture at the required concentrations. Three wells were used for each concentration. The cells which were incubated without the compounds were used as a control. Cells were placed on 96-well microliter plates and cultivated at 37 °C in 5% CO_2_/95% air for 72 h. The cell viability was assessed through an MTT [3-(4,5-dimethylthiazol-2-yl)-2,5-phenyl-2H-tetrazolium bromide] conversion assay [55]. A total of 1% MTT was added to each well. Four hours later, DMSO was added and mixed for 15 min. Optical density (*D*) of the samples was measured on a BioRad 680 multi-well spectrophotometer (USA) at the wavelength of 450 nm. The 50% cytotoxic dose GI_50_) of each compound (i.e., the compound concentration that causes the death of 50% of cells in a culture, or decreases the optical density twice as compared to the control wells) was calculated from the data obtained. Statistical processing of the results was performed using the Microsoft Excel-2007, STATISTICA 6.0 and GraphPad Prism 5.0 programs. The results are given as an average value ± a deviation from the average (mean ± standard error of the mean (SEM)). Reliability of differences (*p*) was estimated using the Student t test. The differences with *p* < 0.05 were considered as reliable. The experimental results are given as the data average values obtained from three independently conducted experiments.

#### 4.4.2. Gel Retardation Assay

To study the interaction between test compounds and DNA the electrophoretic mobility shift assay was performed. 0.5 µg of pUC19 plasmid was incubated with 0.025 µM of test compounds at room temperature for 1 h. After that the electrophoresis was performed in agarose gel (1/2%) in TBE buffer (60 V, 4 h). For visualization the gel was staining with ethidium bromide and analyzed with Bio-Rad gel documentation system.

#### 4.4.3. Cell Cycle Analysis

For cell cycle analysis cells were seeded in 6-well plates (30,000 per plate) and incubate for adhesion for 24 h. After that cells were washed with PBS twice and treated with test compounds dissolved in DMSO in GI50 concentration, than incubated for 24 h. Cells were washed with PBS twice, harvested, pelleted and resuspended in 0.5 mL of PBS. For staining the Hoechst 33342 Ready Flow Reagent (Invitrogen) was used according to the instruction. CytoFLEX flow cytometer (Beckman Coulter) was used for analysis. The results are presented as a percentage of cell population standing in different stages of the cell cycle.

#### 4.4.4. DNA Synthesis Investigation

Cell cycle analysis was performed by Flow Cytometry using BrdU and PI staining [63]. For this experiment cells were seeded in 6-well plates (30,000 per plate) and incubate for adhesion for 24 h. After that cells were washed with PBS twice and treated with test compounds dissolved in DMSO in GI_50_ concentration, then incubated for 24 h. For the next step the BrdU stock was added at a final concentration of 30 µM and cells were incubated for 30 min. Cells were washed with PBS, harvested using trypsine and resuspended at 0.3 mL of PBS. Permeabilization of cell membrane was performed as follows: 0.7 mL of ice-cold 100% EtOH was added into each sample, mixed gently and incubate at 4 °C for 1 h. Cells were pelleted and supernatant was removed completely. Then 0.5 mL of 2 N HCl/0.5% Triton X-100 was added and samples were incubated 30 min at room temperature. Cells were pelleted again, supernatant was removed. The pellet was resuspended in 0.5 mL of 0.1 M sodium tetraborate for 2 min and cells were pelleted again, washed with 150 µL of PBS/1% BSA and resuspended in 50 µM 0.5% Tween 20/1% BSA/PBS. Than BrdU antibody (Alexa Fluor 488, Abcam) was added in concentration according to instruction, incubated for 1 h at room temperature. After that the samples were resuspended in 0.5 mL of PBS containing 10 mg/mL RNAse A and 20 mg/mL PI. Samples were incubated at room temperature for 30 min and analyzed immediately or store at 4 °C. CytoFLEX flow cytometer (Beckman Coulter) was used for analysis. The results are presented as a percentage of cell population standing in DNA synthesis process.

## Figures and Tables

**Table 1:** Cytotoxicity of aryl substituted 1-hydroxy-9,10-anthraquinones **5**, **13–17**, **22**, **23**, **25–30**, **33-38, 40–45.**

Compound	Cytotoxicity (GI_50_, μM) Against Cell Lines ^[a]^							
	SNB-19	U-87MG	T98G	LNCAP	DU-145	MT-4	MDA-MB- 231	hTERT Lung Fibroblasts
**5**	6.7 ± 0.47	>100	72.5 ± 3.21	21.1 ± 0.85	14.8 ± 0.52	42 ± 4.14	28.6 ± 1.24	28.6 ± 4.54
**13**	24.3 ± 1.08	>100	14.1 ± 0.65	24 ± 1.47	1.1 ± 0.19	71 ± 0.7	31.2 ± 1.65	67.1 ± 8.06
**14**	38.1 ± 1.15	>100	>100	92 ± 3.93	41.1 ± 3.34	65 ± 5.49	>100	>100
**15**	9.6 ± 1.04	45.4 ± 1.87	11.6 ± 0.78	28.5 ± 0.96	6.5 ± 0.48	32.1 ± 1.54	6.8 ± 0.58	42.01 ± 2.04
**16**	9.7 ± 0.41	>100	25.1 ± 1.04	>100	5.4 ± 0.17	>100	16.5 ± 0.88	48.0 ± 3.87
**17**	10.2 ± 0.86	8.2 ± 1.35	9.2 ± 0.56	16.7 ± 1.34	3.5 ± 0.96	38.3 ± 3.16	9.6 ± 0.69	26.6 ± 2.44
**22**	>100	>100	>100	21.1 ± 0.85	24.2 ± 1.32	>100	>100	52.2 ± 8.01
**23**	37.0 ± 1.41	58.7 ± 2.48	34.8 ± 1.06	22.2 ± 2.12	15.4 ± 0.96	56.1 ± 1.96	18.2 ± 1.27	69.5 ± 1.52
**25**	6.8 ± 1.19	>100	21.5 ± 0.92	32.1 ± 8.55	14.5 ± 0.73	>100	16.5 ± 0.88	44.6 ± 2.46
**26**	>100	>100	>100	74 ± 3.74	70.2 ± 1.47	80 ± 5.21	>100	87.5 ± 9.11
**27**	8.5 ± 0.32	35.8 ± 2.17	21.6 ± 0.38	>100	19.1 ± 0.78	38.6 ± 1.36	23.2 ± 2.34	46.5 ± 1.21
**28**	5.77 ± 0.23	>100	32.6 ± 1.68	23.1 ± 1.24	>100	18.2 ± 0.86	42.4 ± 3.32	51.3 ± 3.18
**29**	15.3 ± 0.71	34.1 ± 2.18	29.6 ± 2.21	36.2 ± 2.05	13.8 ± 1.05	36.6 ± 1.68	35.2 ± 1.88	28.1 ± 2.12
**30**	16.8 ± 2.11	21.6 ± 1.42	39.2 ± 1.65	>100	12.5 ± 3.17	55.1 ± 3.24	39.1 ± 1.71	25.6 ± 5.24
**33**	18.3 ± 1.12	56.8 ± 2.36	24.6 ± 0.85	21.3 ± 1.16	>100	>100	28.3 ± 1.06	52.8 ± 8.05
**34**	>100	>100	>100	>100	>100	>100	>100	72.2 ± 4.18
**35**	10.1 ± 1.08	25.7 ± 3.14	9.8 ± 0.56	26.7 ± 1.86	5.9 ± 0.52	20.2 ± 1.04	7.6 ± 0.64	17.7 ± 0.96
**36**	37.0 ± 1.41	36.8 ± 1.42	15.5 ± 0.92	16.7 ± 1.86	10.6 ± 1.12	25.8 ± 0.94	22.6 ± 0.86	12.2 ± 2.08
**37**	9.1 ± 0.68	8.7 ± 0.89	10.1 ± 0.78	6.2 ± 0.43	5.8 ± 0.38	56.6 ± 1.28	8.2 ± 0.96	10.5 ± 1.98
**38**	39.4 ± 2.16	>100	7.8 ± 0.56	>100	6.5 ± 0.89	>100	32.5 ± 5.37	19.4 ± 0.97
**40**	6.1 ± 0.38	10.2 ± 1.03	8.1 ± 0.98	22.2 ± 1.26	7.1 ± 0.72	35.6 ± 3.28	10.5 ± 1.03	16.5 ± 0.46
**41**	14.1 ± 3.15	44.5 ± 1.56	29.7 ± 2.05	41.1 ± 5.15	>100	44.1 ± 3.23	15.8 ± 0.82	17.2 ± 0.62
**42**	31.6 ± 2.16	>100	11.5 ± 1.34	71.8 ± 9.76	23.9 ± 4.28	>100	21.1 ± 3.28	18.2 ± 2.19
**43**	19.2 ± 2.52	>100	16.1 ± 0.78	>100	18.6 ± 2.45	>100	17.1 ± 1.55	34.1 ± 0.92
**44**	41.8 ± 5.27	>100	16.5 ± 1.26	14.2 ± 3.12	15.2 ± 1.06	40.5 ± 6.44	19.1 ± 0.98	36.2 ± 1.31
**45**	11.2 ± 1.49	18.2 ± 1.06	28.3 ± 3.25	7.2 ± 0.74	22.6 ± 0.91	31.5 ± 1.52	14.2 ± 1.42	20.5 ± 2.68
**DOX**	7.62 ± 0.69	6.11 ± 0.15	4.5 ± 0.46	2.11 ± 0.4	6.61 ± 0.34	0.2 ± 0.04	7.9 ± 0.51	2.2 ± 0.58

^[a]^ GI_50_: concentration at which 50% growth inhibition of tumor cells is observed after 72 h incubation; The experimental results are given as the data average values obtained from three independently conducted experiments.

**Table 2:** Results of molecular docking with the topoisomerase IIβ-DNA complex.

Compound	Binding Affinity, kcal/mol	Compound	Binding Affinity, kcal/mol	Compound	Binding Affinity, kcal/mol
**Mitoxantrone**	−13.234	23	−7.530	**35**	−7.817
**13**	−8.559	25	−6.973	**40**	−8,954
**15**	−9.530	27	−8.093	**44**	−8.468
**16**	−7.683	28	−8.268	**45**	−7.056

**Table 3:** Cell cycle analysis in malignant glioblastoma cell line SNB-19.

Compound	Concentration, μM ^[b]^	Population (% Cell Distribution)					
		Sub-G1	G0/G1	S	G2/M	Super-G2	DNA Synthesis
**13**	24.3	4.0	29.0	48.5	13.9	4.6	28.63
**16**	9.7	3.0	36.6	34.3	18.6	7.6	28.67
**23**	37.0	3.1	34.6	32.5	17.3	11.8	22.71
**28**	5.7	3.6	54.5	22.4	13.7	5.8	16.83
**Doxorubicin**	2.0	1.9	49.6	23.2	19.6	5.7	11.81
**Mitomycin C**	0.15	0.0	63.6	25.2	4.1	7.0	7.44
**Control ^[a]^**	-	0.7	42.9	12.5	35.5	8.5	18.40

^[a]^ Non-treated cells; ^[b]^ The GI_50_ value.

**Table 4:** Cell cycle analysis in DU-145 cell lines.

Compound	Concentration, μM ^[b]^	Population (% Cell Distribution)					
		Sub-G1	G0/ G1	S	G2/M	Super-G2	DNA Synthesis
**13**	1.1	34.6	28.7	28.7	6.3	1.6	6.83
**16**	5.4	15.7	44.3	30.7	7.3	2.0	27.22
**25**	14.5	25.9	32.3	32.4	7.4	2.0	20.27
**Doxorubicin**	2.0	0.3	48.8	28.1	15.9	6.8	10.76
**Mitomycin C**	0.15	0.1	50.3	13.2	16.3	20.2	5.74
**Control ^[a]^**	-	0.4	50.6	25.8	14.5	8.7	11.81

^[a]^ Non-treated cells; ^[b]^ The GI_50_ value.

## Supplementary Materials

Supplementary data (copies of ^1^H and ^13^C NMR spectra)can be found in the online version.
