# Supplementary material for: 1-Hydroxyanthraquinones Containing Aryl Substituents as Potent and Selective Anticancer Agents
Source: Molecules. 2020 May 29;25(11):2547. doi: 10.3390/molecules25112547 (PMC7321108; doi:10.3390/molecules25112547)
Supplement: Supplementary file 1 [file molecules-25-02547-s001.pdf]

Molecules

Supporting Information

1-Hydroxyanthraquinones containing aryl substituents as potent and selective anticancer agents

Nafisa S. Sirazhetdinova [1], Victor A. Savelyev [1], Tatyana S. Frolova [2,3], Dmitry S. Baev [1], Olga S. Oleshko [3], Teresa Sarojan [3], Lyubov S. Klimenko [4], Ivan V. Chernikov [5], Andrey G. Pokrovskii [3], Elvira E. Shults [1*]

[1]*Laboratory of Medicinal Chemistry, N.N. Vorozhtsov Novosibirsk Institute of Organic Chemistry, Siberian Branch of the Russian Academy of Sciences, Lavrentyev Ave, 9, 630090 Novosibirsk, Russian Federation*

[2]*The Federal Research Center Institute of Cytology and Genetics, Acad. Lavrentyev Ave., 10, 630090, Novosibirsk, Russia*

[3]*Novosibirsk State University, Pirogova Str. 1, 630090 Novosibirsk, Russian Federation*

[4] *Yugra State University, 628012, Khanty-Mansiysk, Russian Federation*

[5] *Institute of Chemical Biology and Fundamental Medicine Siberian Branch of the Russian Academy of Sciences, Lavrentyev Ave, 9, 630090 Novosibirsk, Russian Federation*

Content:

^{1}H and ^{13}C NMR spectra of synthesized compounds……………………..……S2-S33
HR-MS spectra of synthesized compounds………………………………….....S34-S65
IR spectra of selected compounds ………………………………….………… S65-S70
UV spectra of selected compounds…………………………………..………S71-S76
Docking of new derivatives **15**, **16**, **23**, **25**, **27**, **28**, **35, 40** and 44 in topoisomerase IIβ-DNA complex binding site……………………………….S77

✉ Elvira E. Shults

schultz@nioch.nsc.ru

[1] Laboratory of Medicinal Chemistry, Novosibirsk Institute of Organic Chemistry, Siberian Branch of the Russian Academy of Sciences, Lavrentyev Ave, 9, 630090 Novosibirsk, Russia;

<u>^{1}H and ^{13}C NMR spectra</u>

1-Hydroxy-4-(3,4,5-trimethoxyphenyl)anthracene-9,10-dione (5)

^{1}H NMR ($CDCl_3$, 500 MHz)

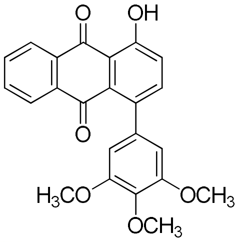

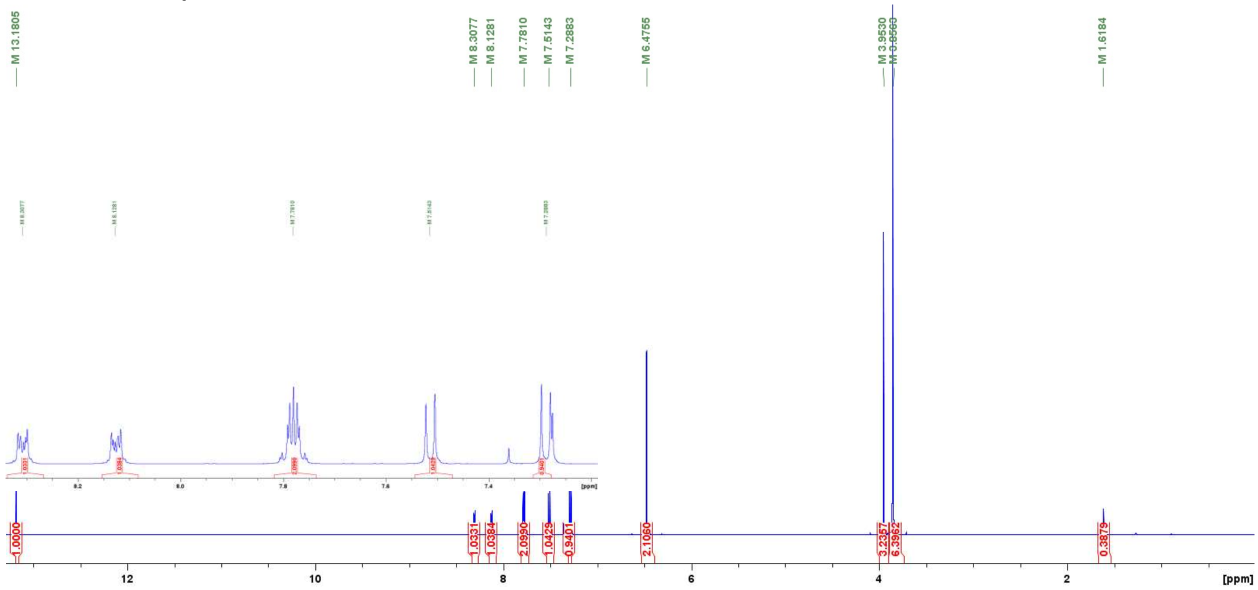

^{13}C NMR ($CDCl_3$, 125 MHz)

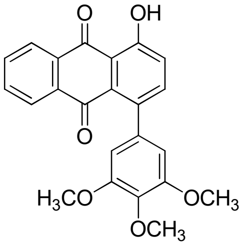

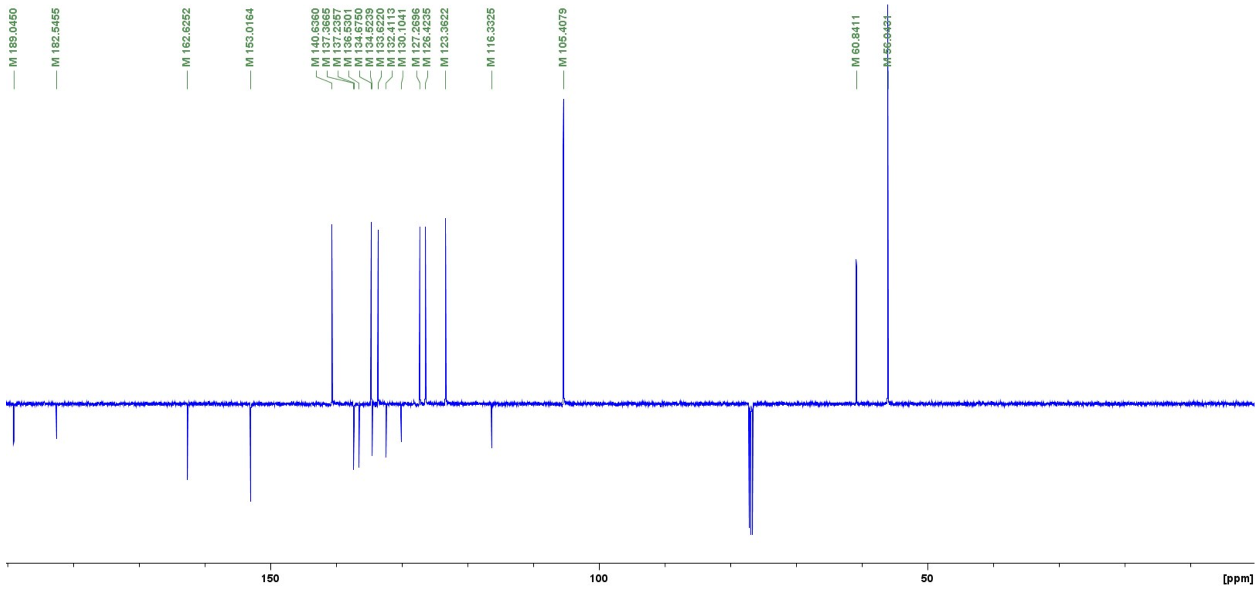

1-Hydroxy-4-phenylanthracene-9,10-dione (13)

^{1}H NMR ($CDCl_3$, 400 MHz)

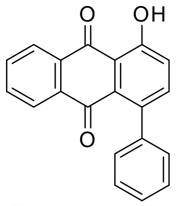

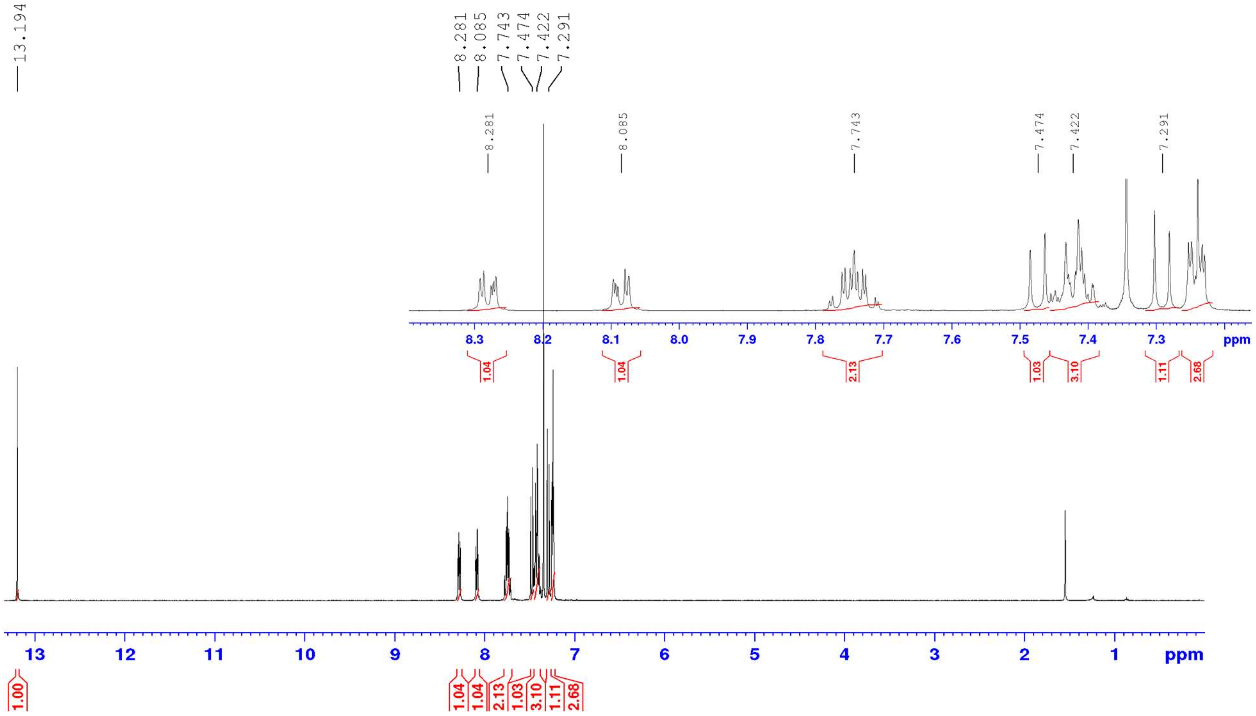

^{13}C NMR ($CDCl_3$, 125 MHz)

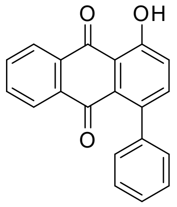

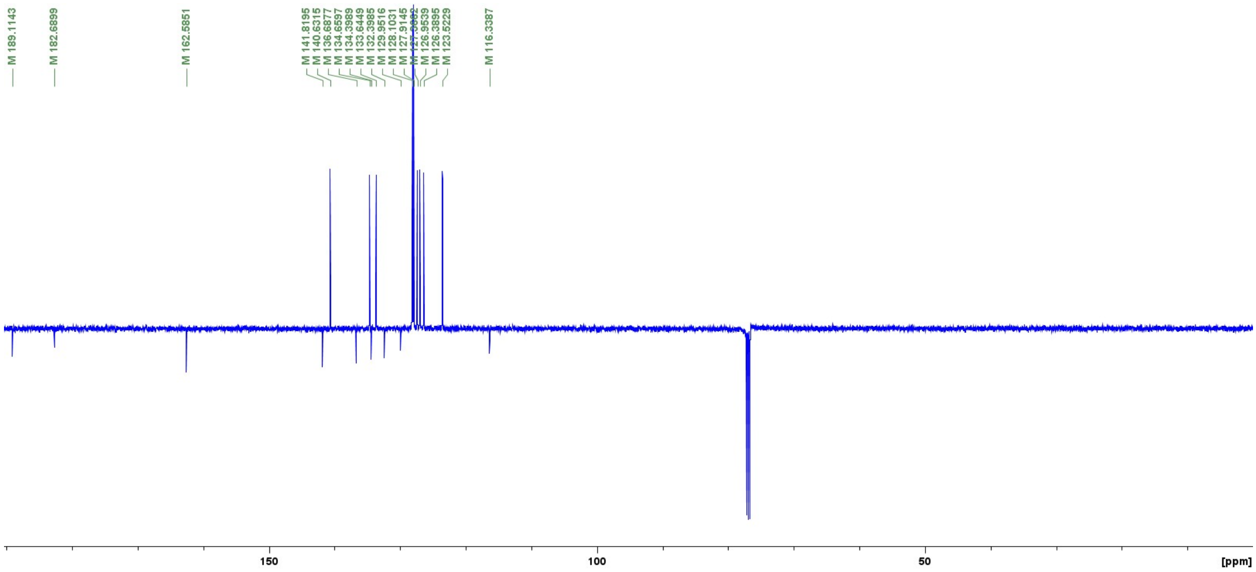

1-Hydroxy-4-(o-tolyl)anthracene-9,10-dione (14)

^{1}H NMR ($CDCl_3$, 400 MHz)

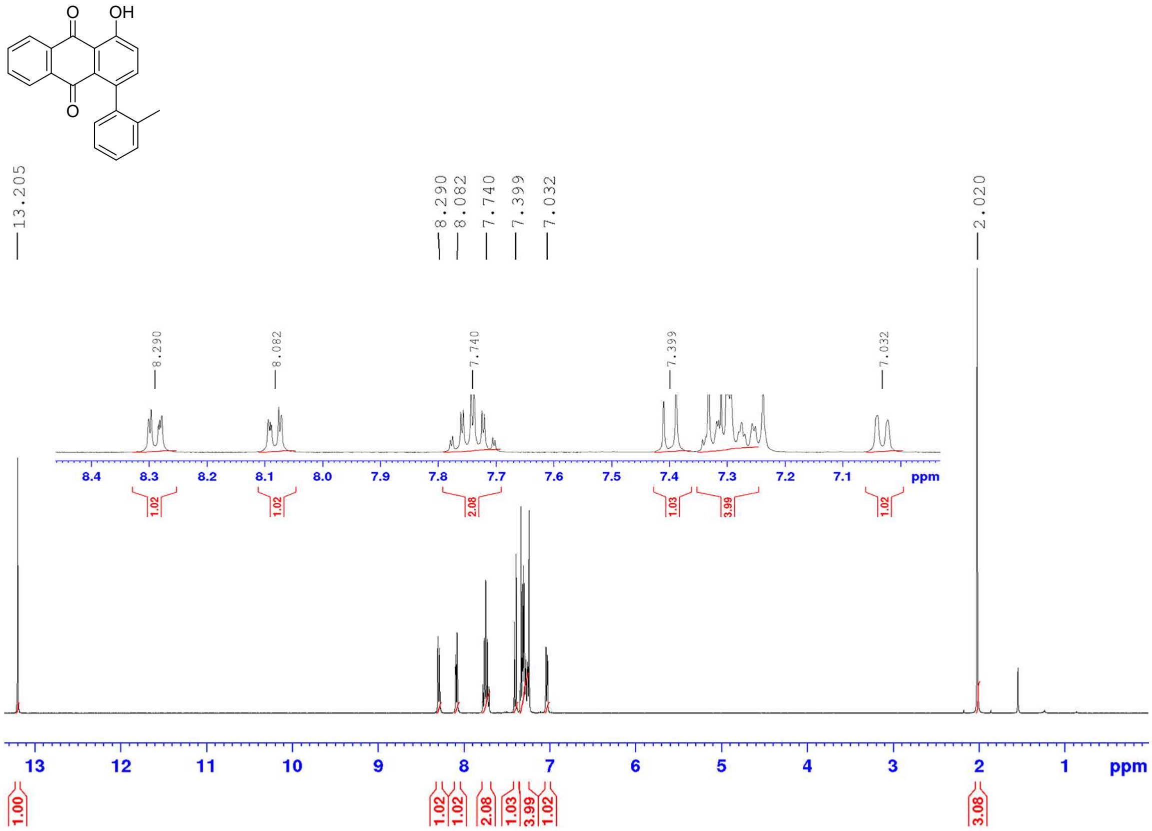

^{13}C NMR ($CDCl_3$, 75 MHz)

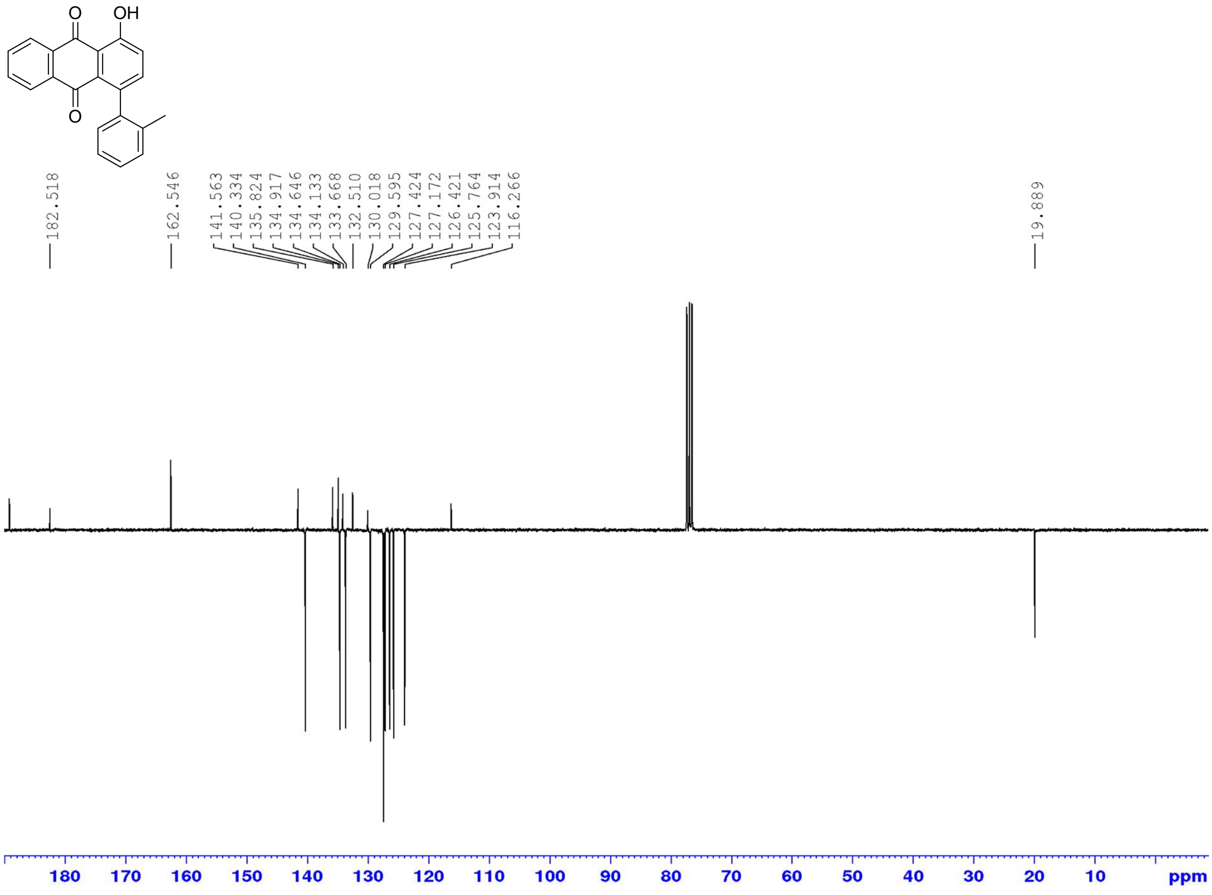

1-Hydroxy-4-(4-methoxyphenyl)anthracene-9,10-dione (15)

^{1}H NMR ($CDCl_3$, 300 MHz)

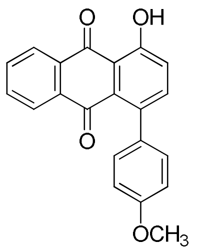

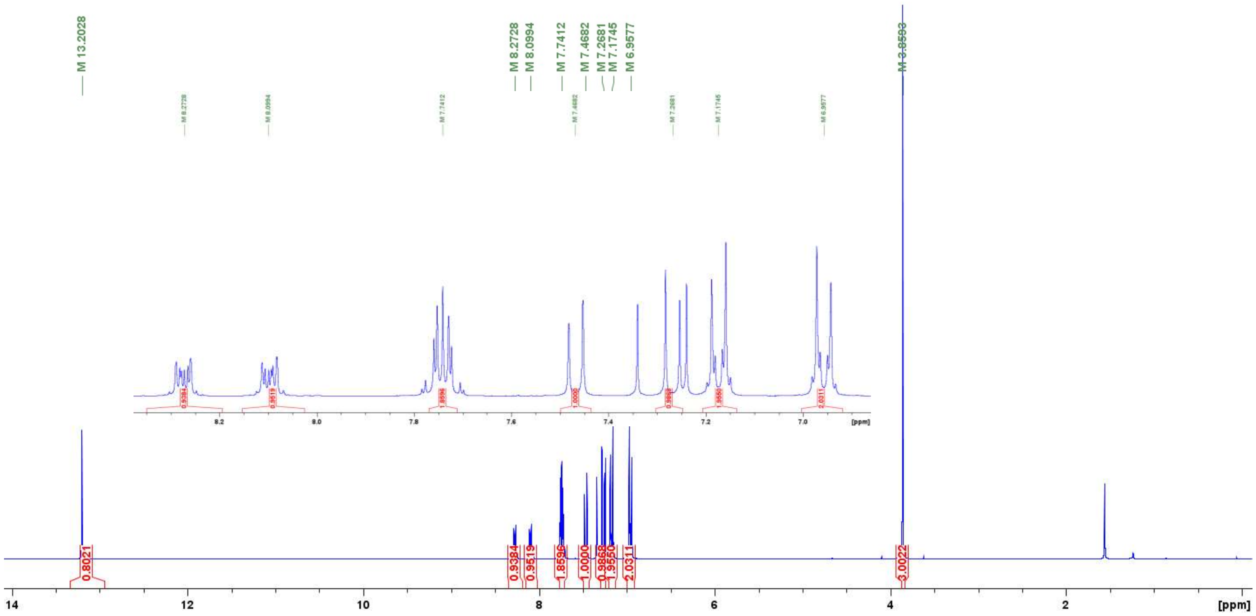

^{13}C NMR ($CDCl_3$, 125 MHz)

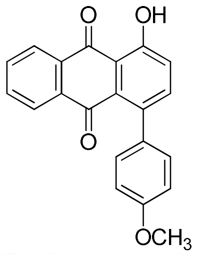

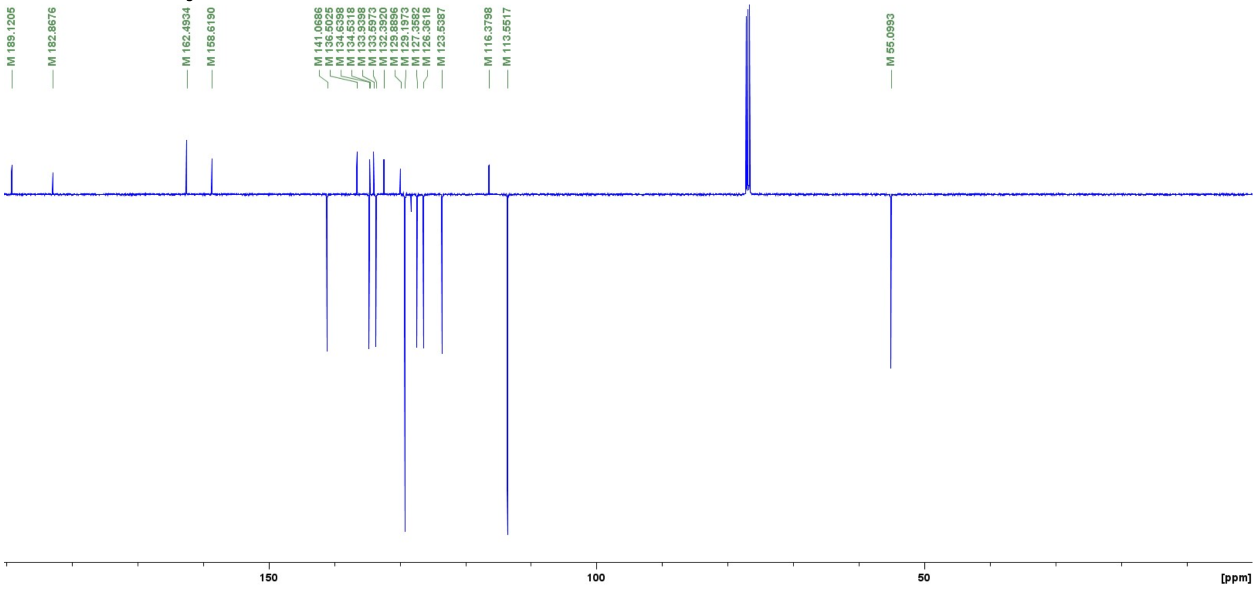

1-(2,3-Dimethoxyphenyl)-4-hydroxyanthracene-9,10-dione (16)

^{1}H NMR ($CDCl_3$, 400 MHz)

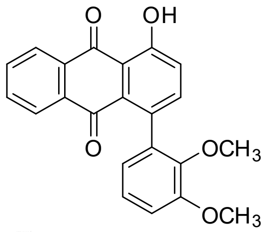

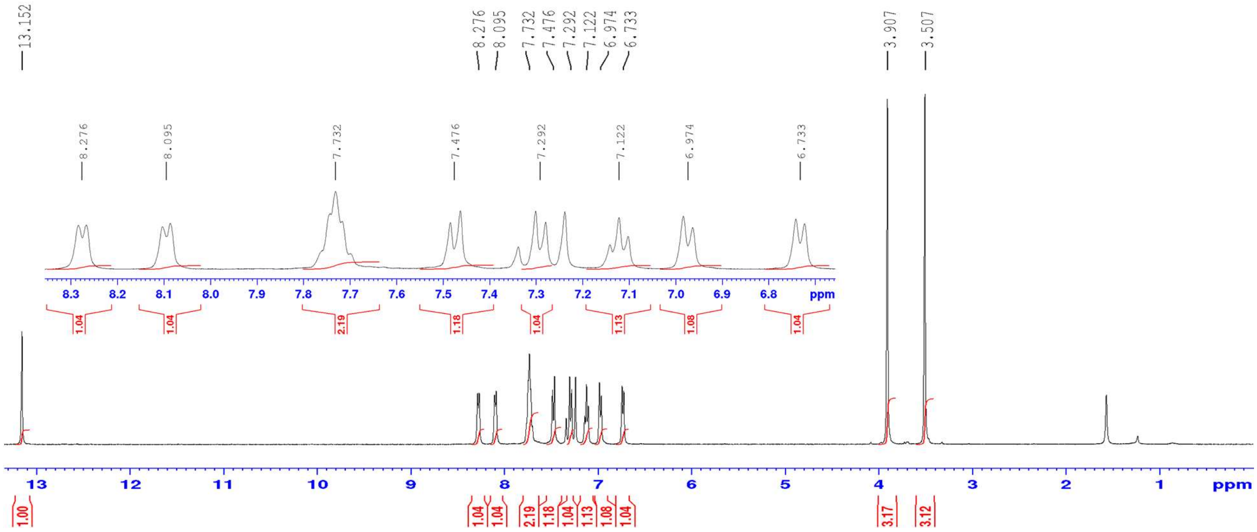

^{13}C NMR ($CDCl_3$, 75 MHz)

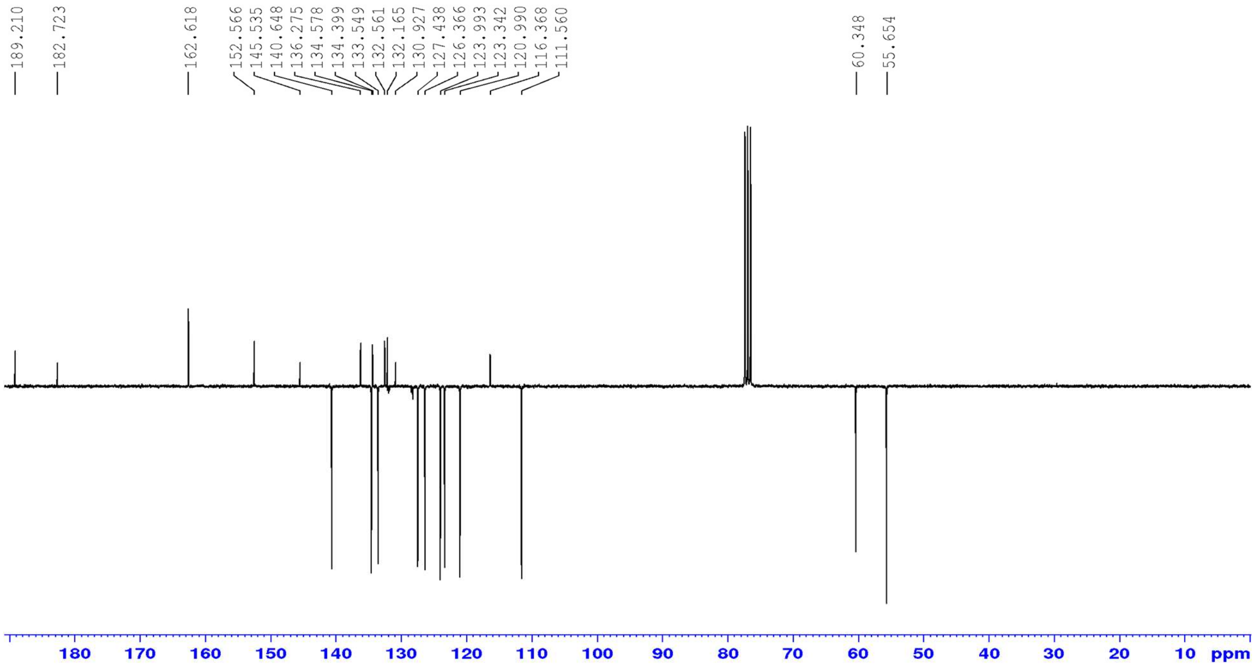

1-(3,5-Difluorophenyl)-4-hydroxyanthracene-9,10-dione (17)

^{1}H NMR (CDCl$_3$, 400 MHz)

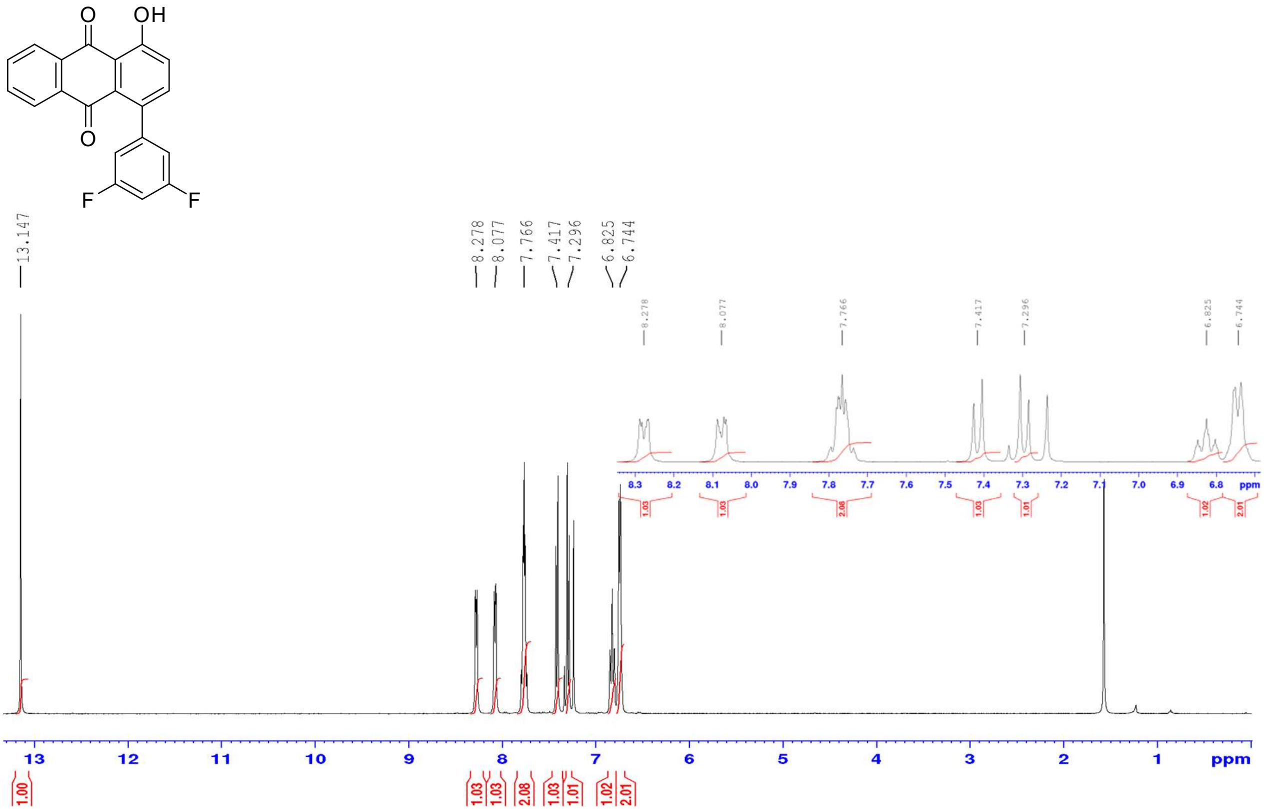

^{13}C NMR (CDCl$_3$, 75 MHz)

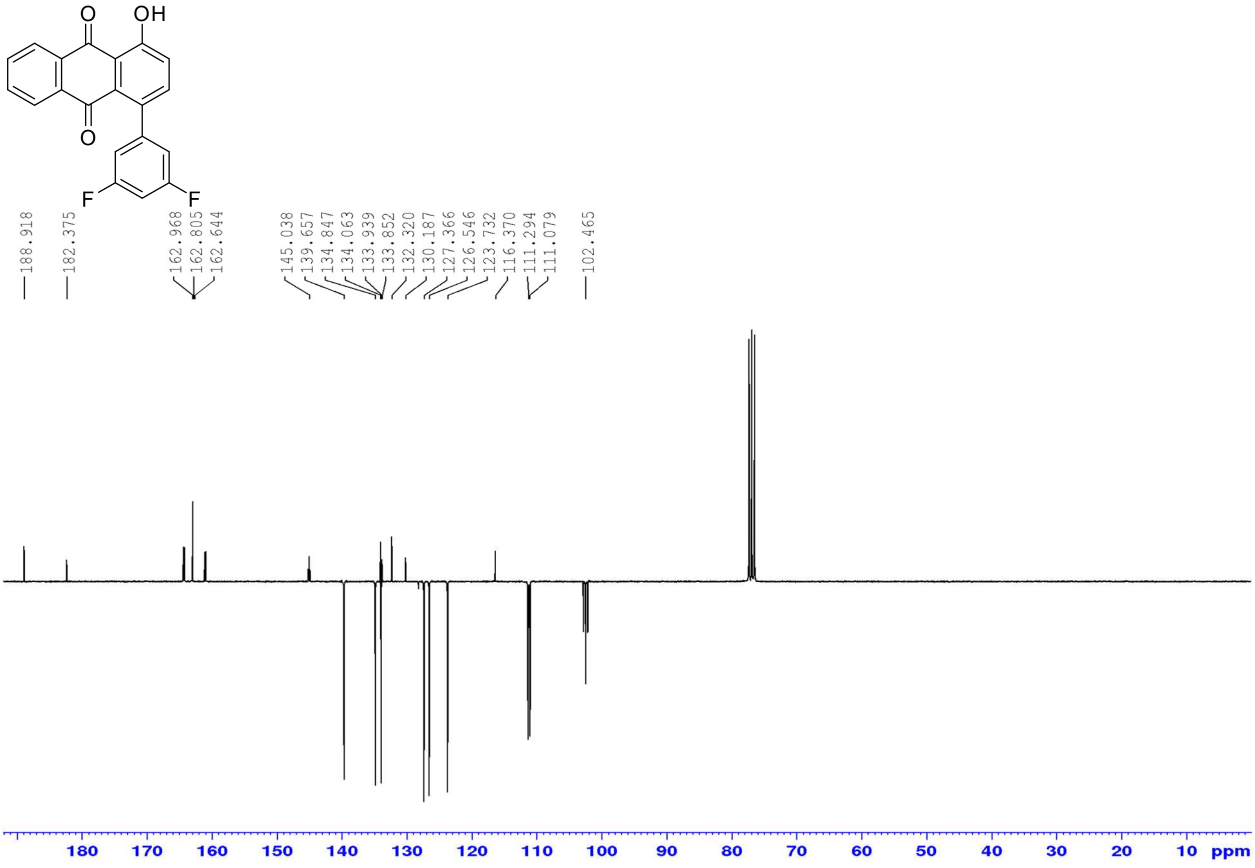

1-(2-Chloro-5-(trifluoromethyl)phenyl)-4-hydroxyanthracene-9,10-dione (18)

^{1}H NMR ($CDCl_3$, 300 MHz)

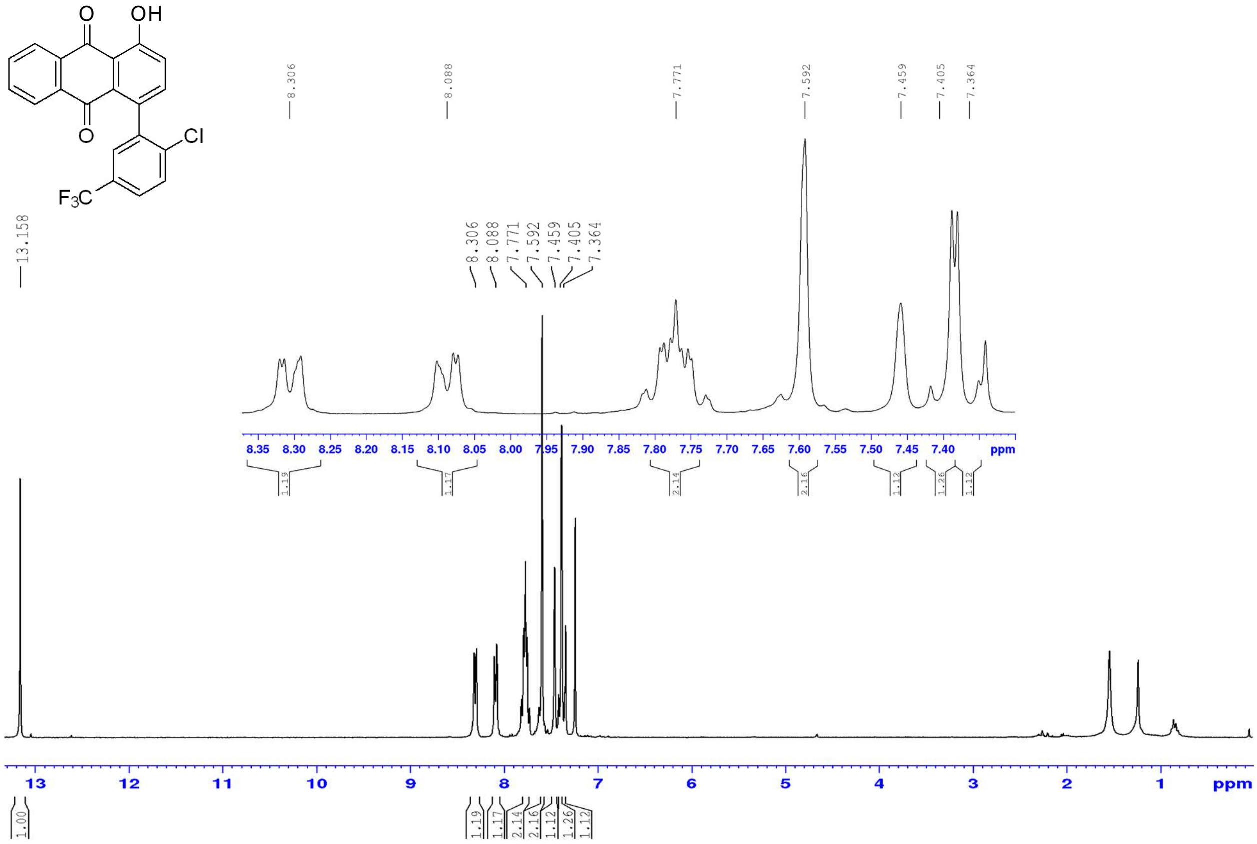

^{13}C NMR ($CDCl_3$, 100 MHz)

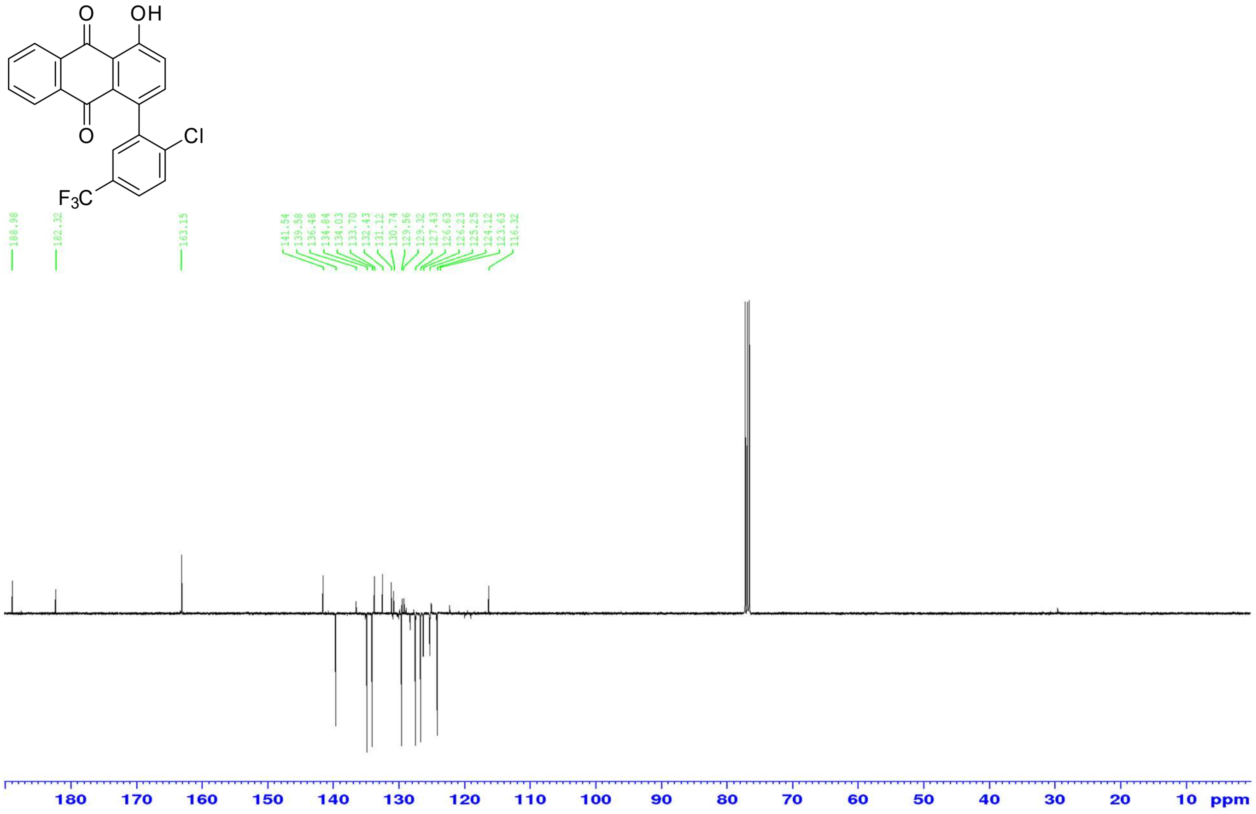

1-(4-Chloro-2-(trifluoromethyl)phenyl)-4-hydroxyanthracene-9,10-dione (19)

^{1}H NMR ($CDCl_3$, 400 MHz)

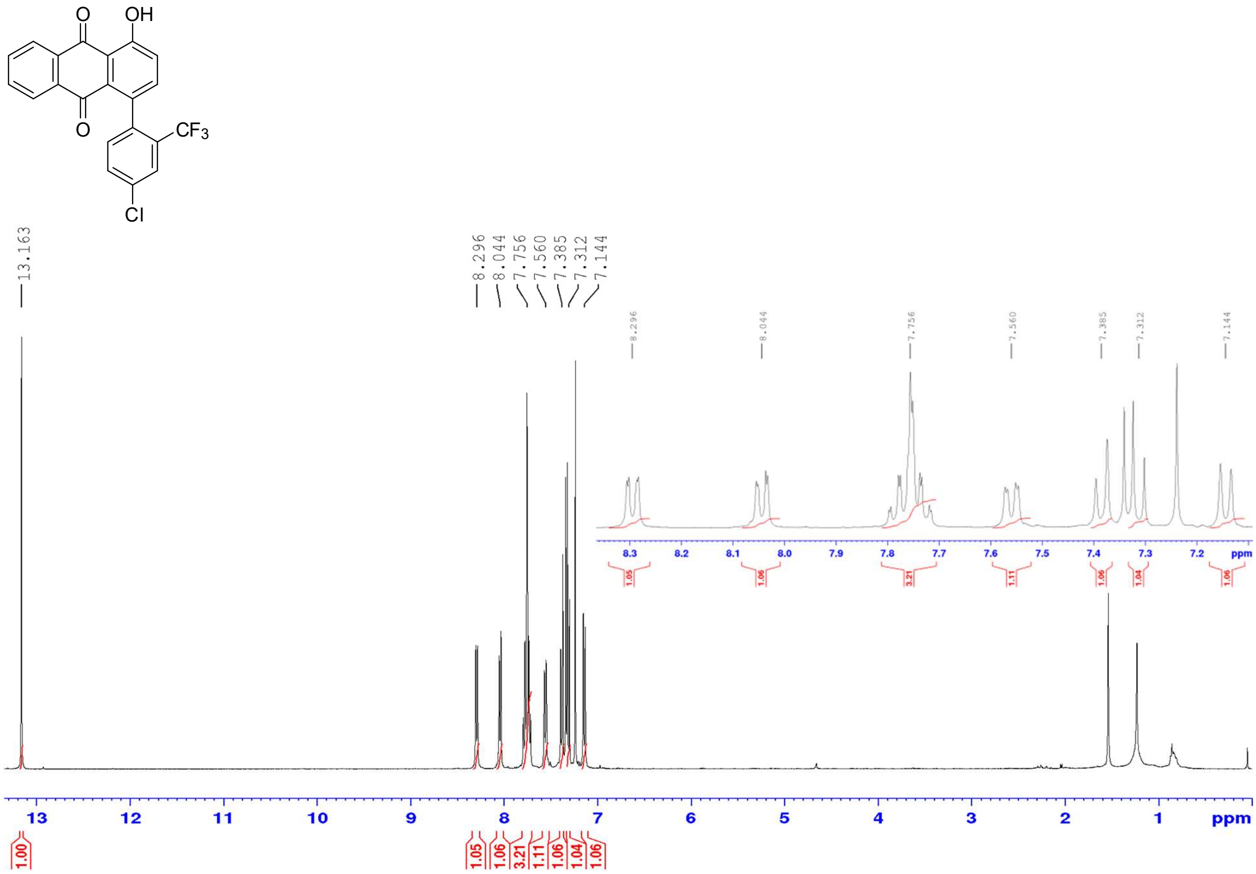

^{13}C NMR ($CDCl_3$, 125 MHz)

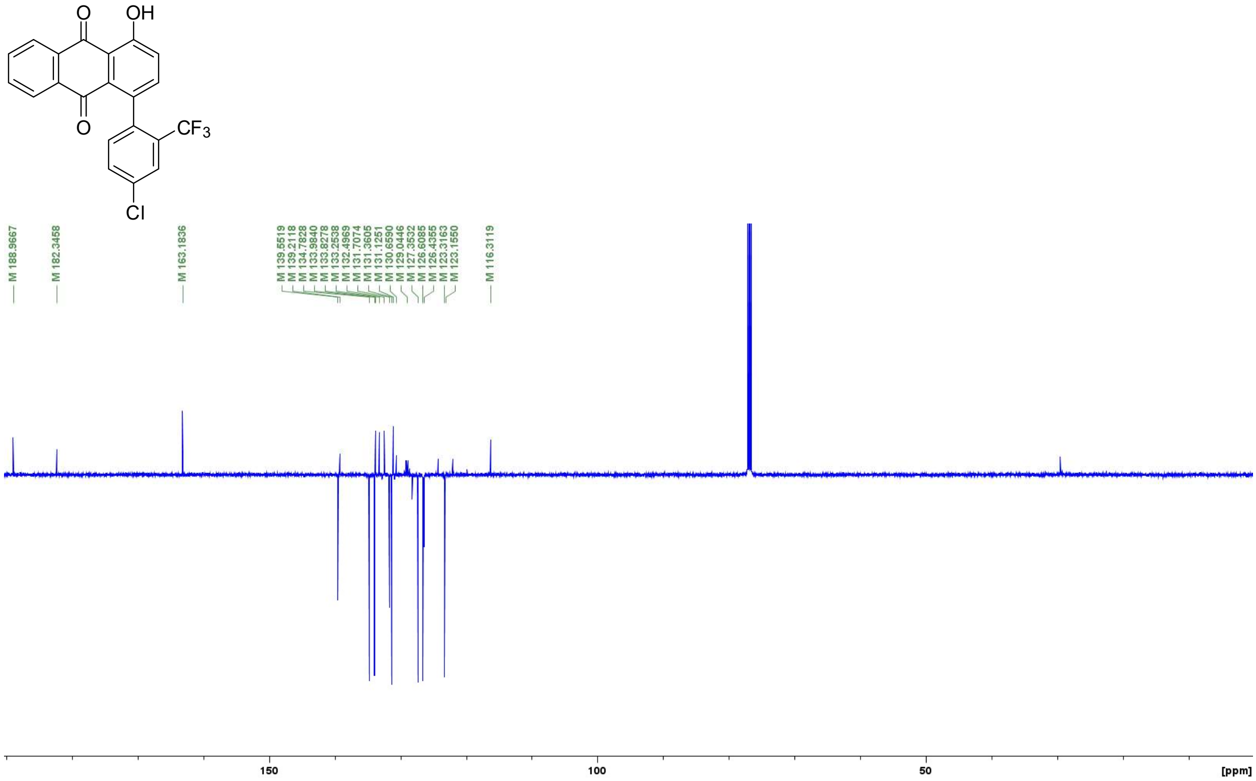

1-(Furan-2-yl)-4-hydroxyanthracene-9,10-dione (22)

^{1}H NMR ($CDCl_3$, 300 MHz)

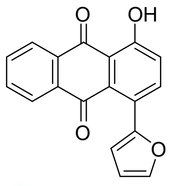

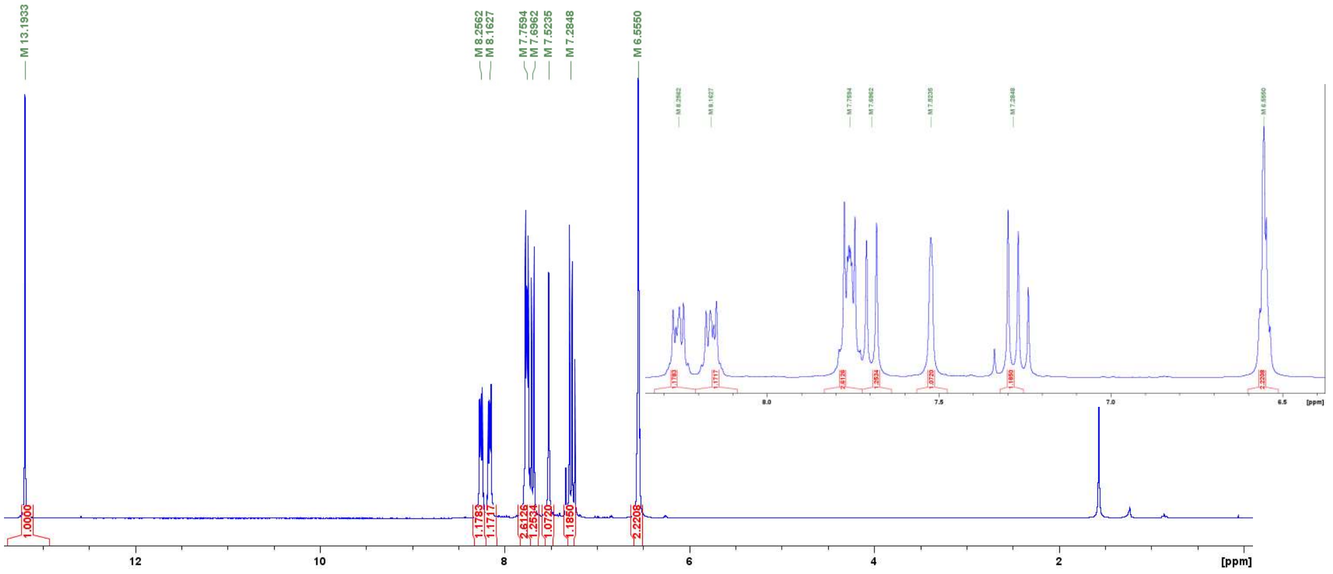

^{13}C NMR ($CDCl_3$, 75 MHz)

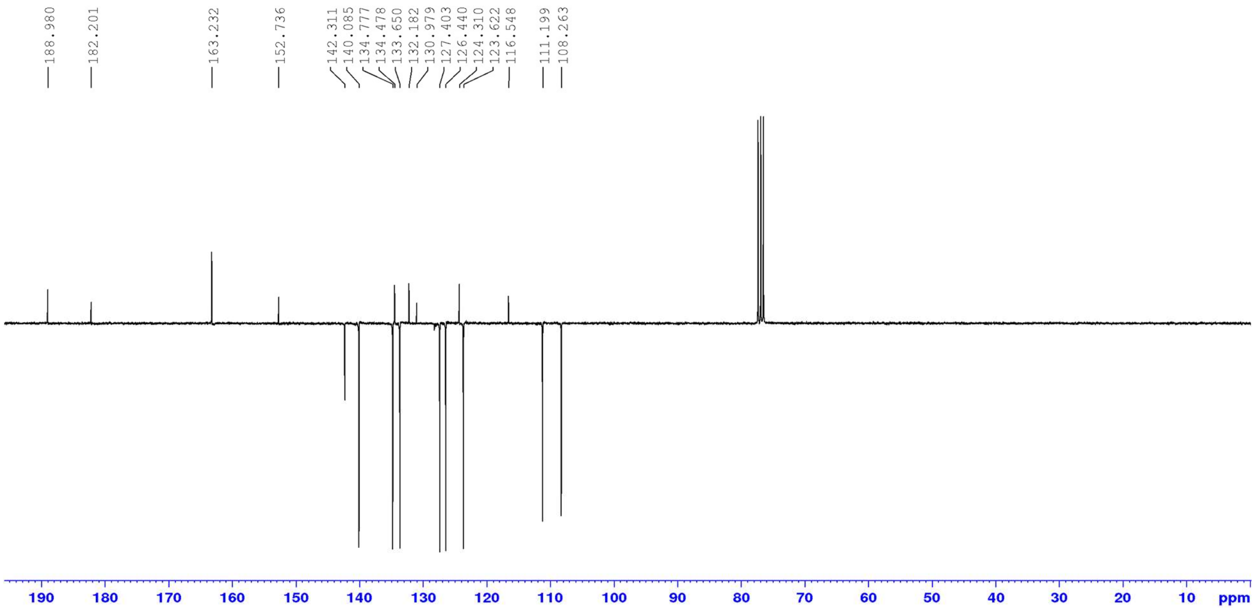

1-(Furan-3-yl)-4-hydroxyanthracene-9,10-dione (23)

^{1}H NMR ($CDCl_3$, 400 MHz)

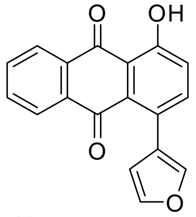

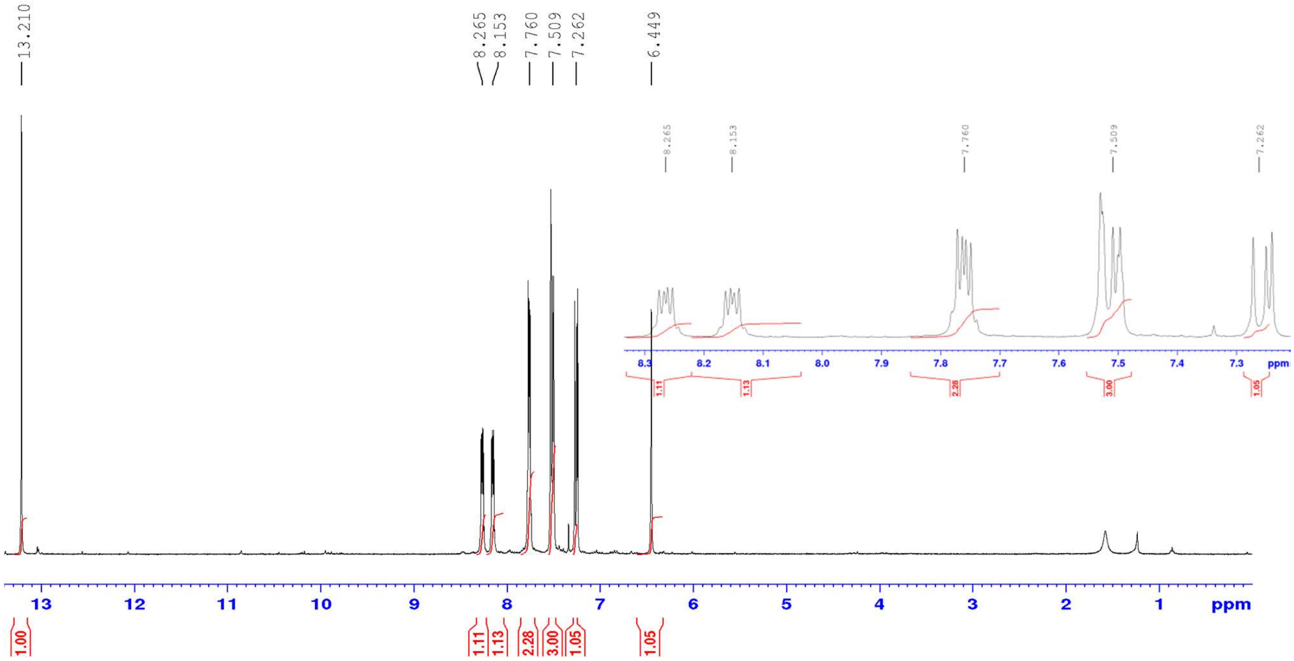

^{13}C NMR ($CDCl_3$, 100 MHz)

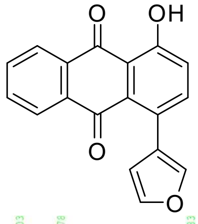

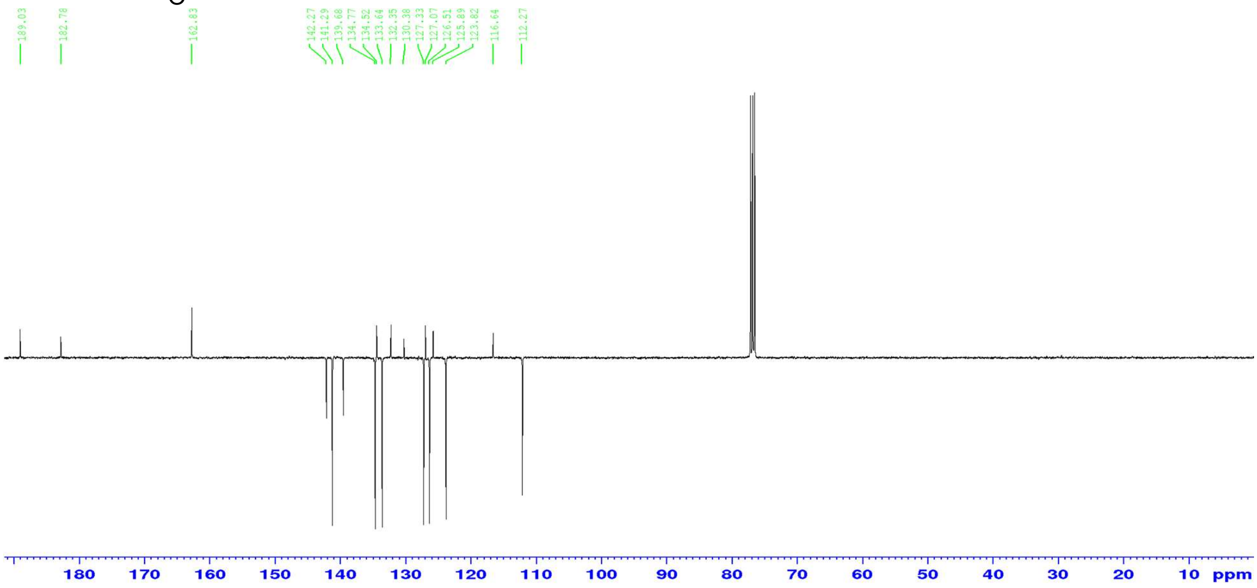

1-Hydroxy-2-(3,4,5-trimethoxyphenyl)anthracene-9,10-dione (24)

^{1}H NMR ($CDCl_3$, 400 MHz)

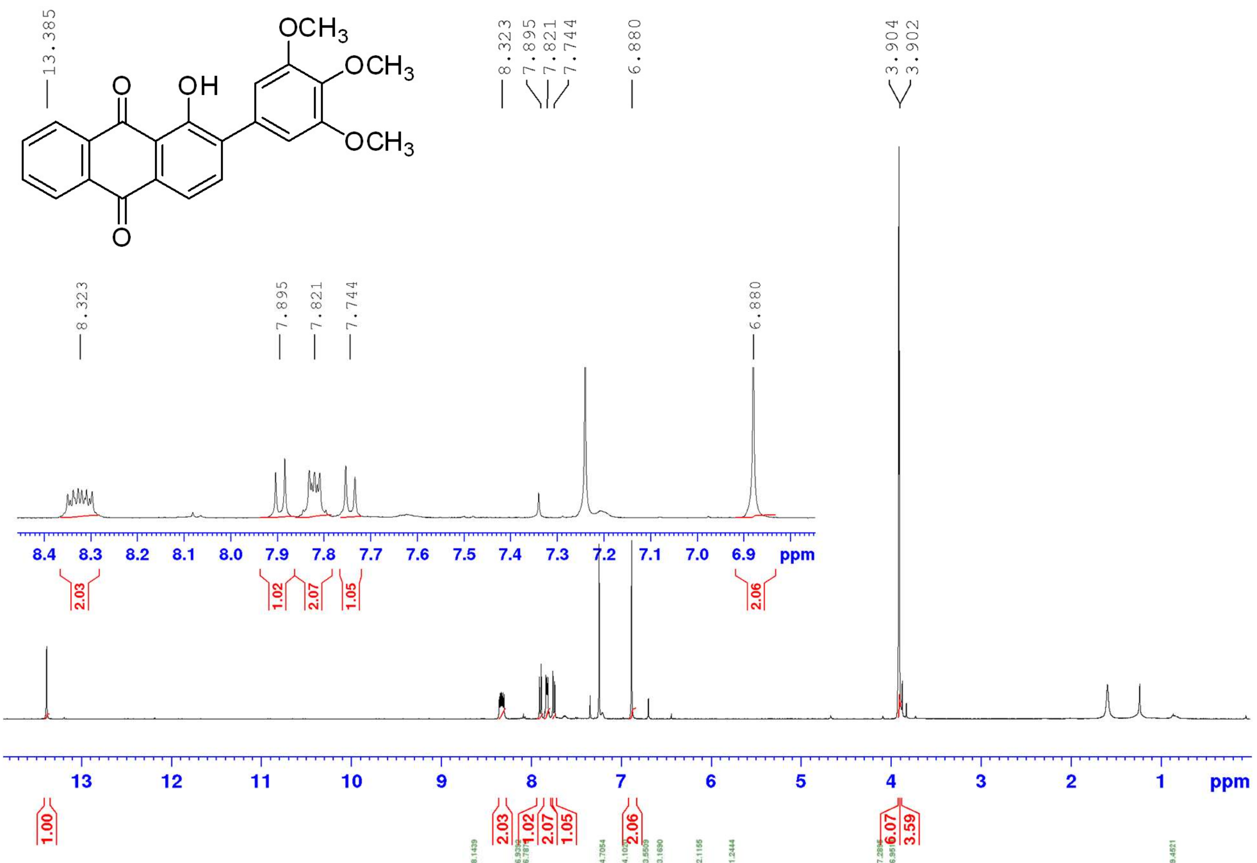

^{13}C NMR ($CDCl_3$, 125 MHz)

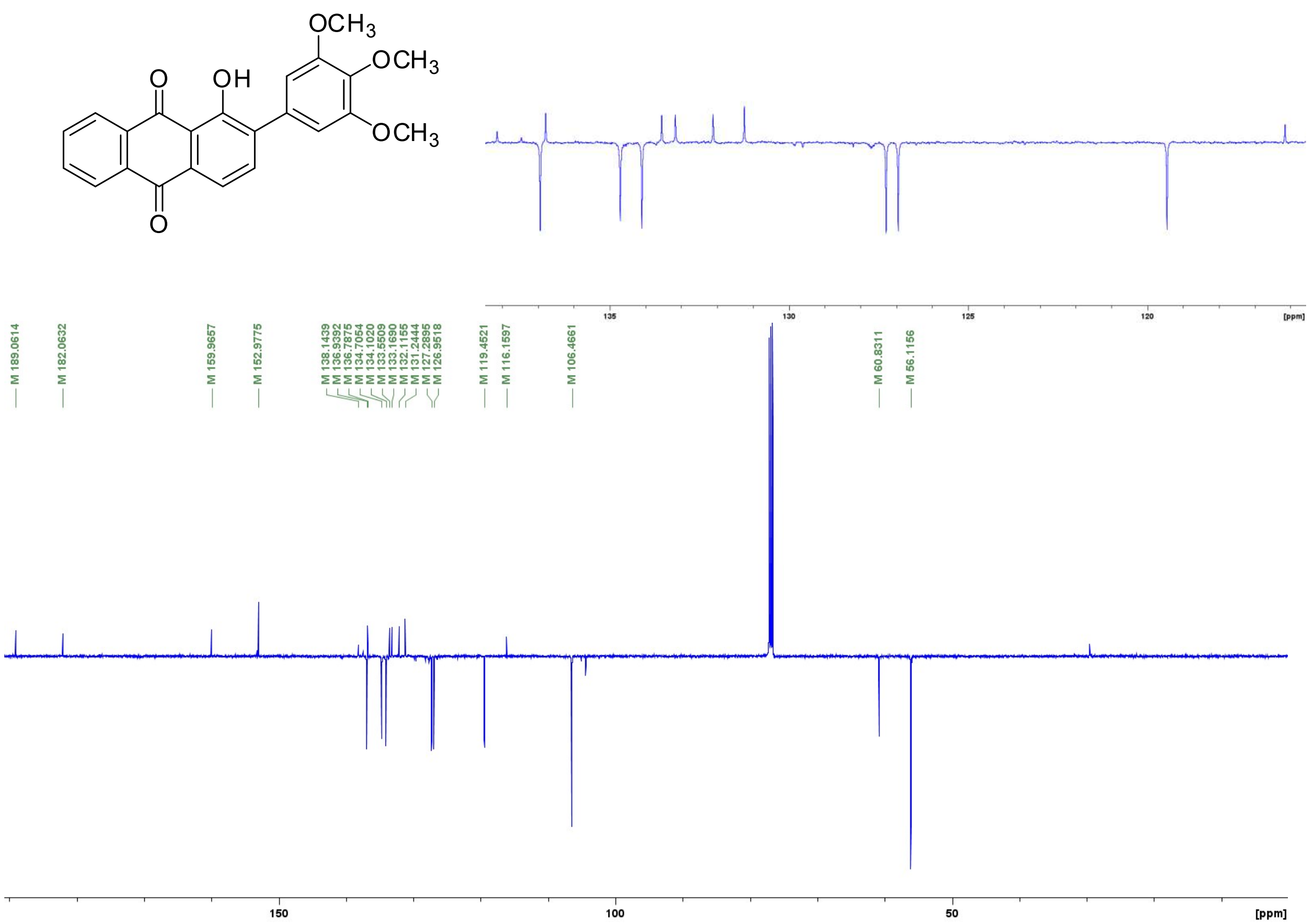

1-Hydroxy-2-phenylanthracene-9,10-dione (25)

^{1}H NMR ($CDCl_3$, 500 MHz)

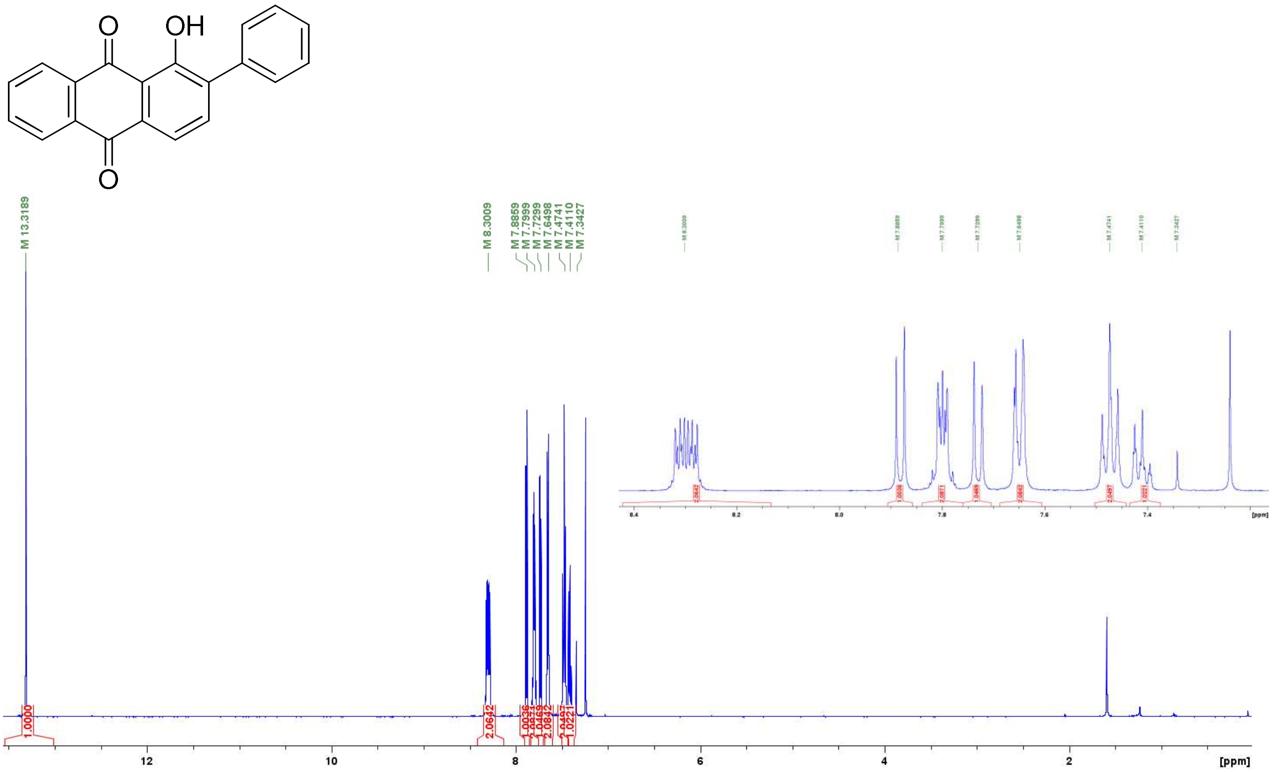

^{13}C NMR ($CDCl_3$, 125 MHz)

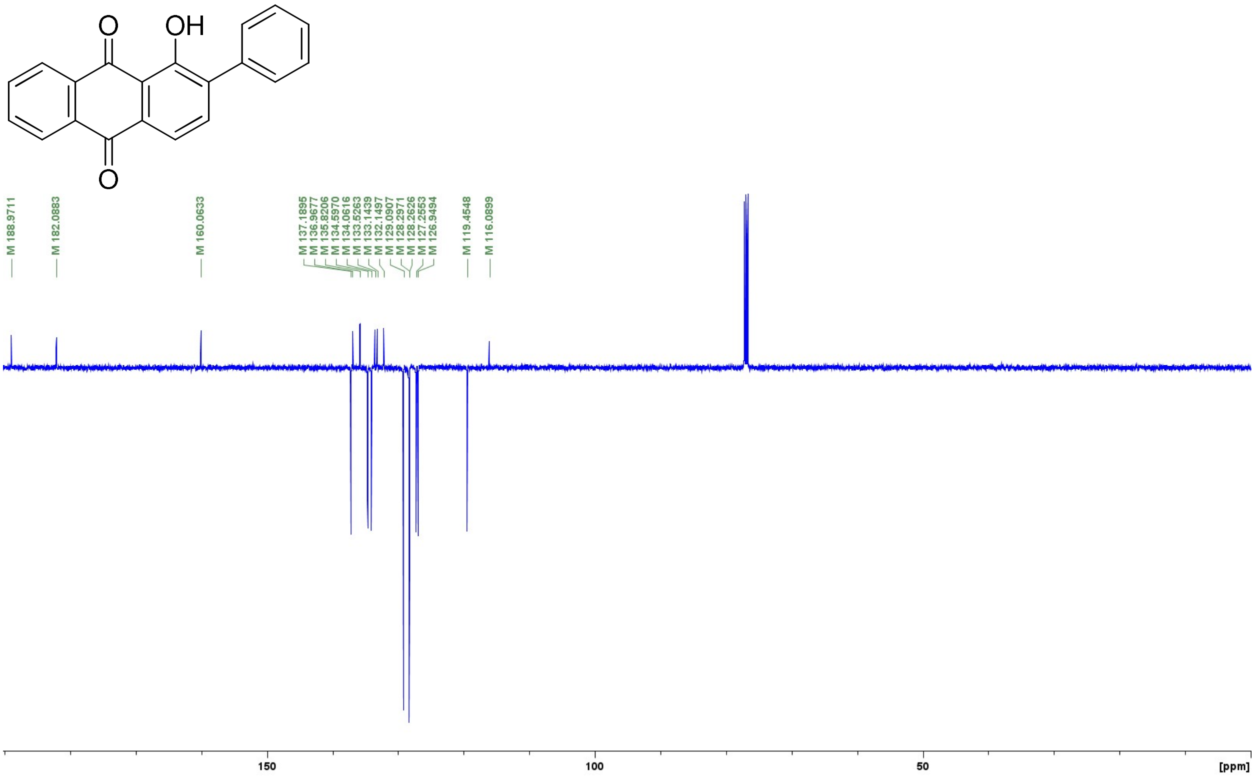

1-Hydroxy-2-(o-tolyl)anthracene-9,10-dione (26)

^{1}H NMR ($CDCl_3$, 400 MHz)

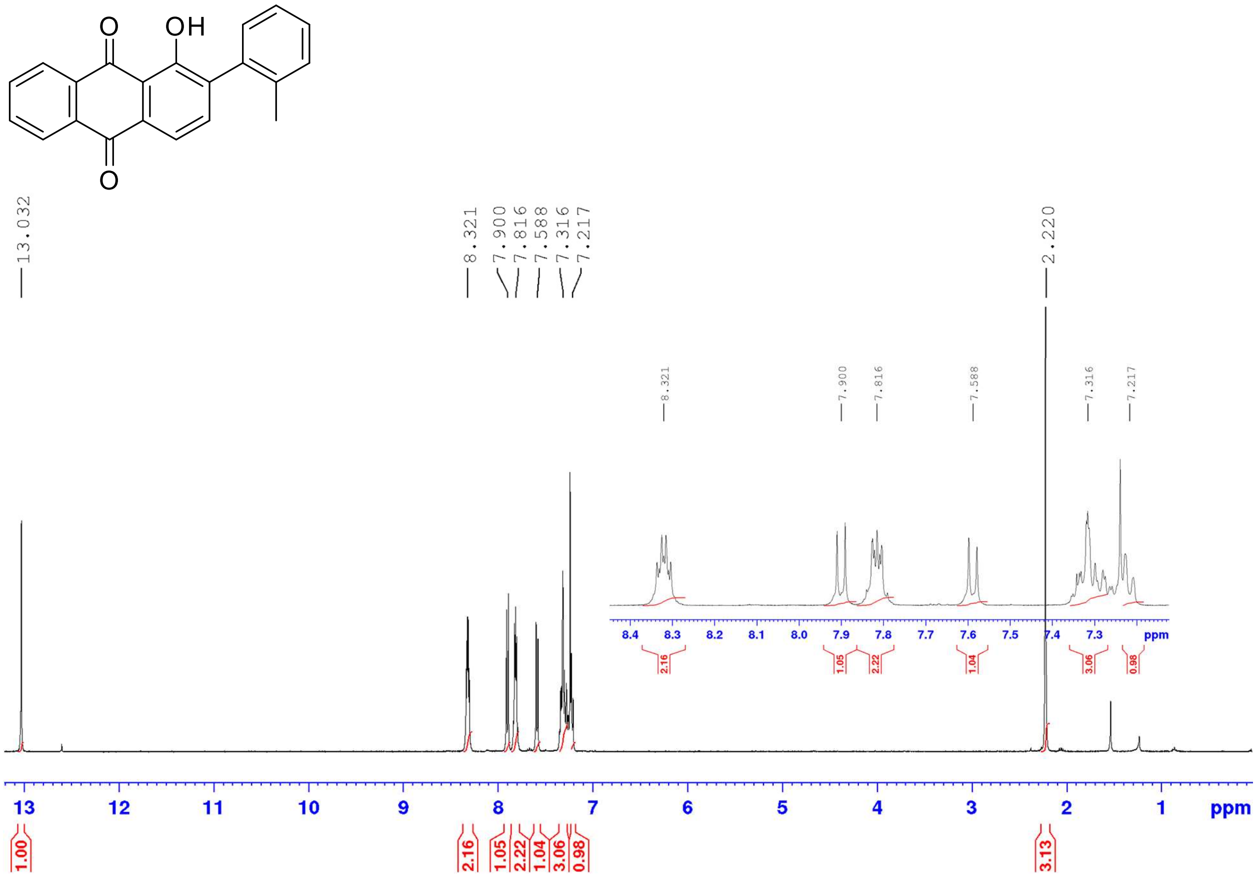

^{13}C NMR ($CDCl_3$, 125 MHz)

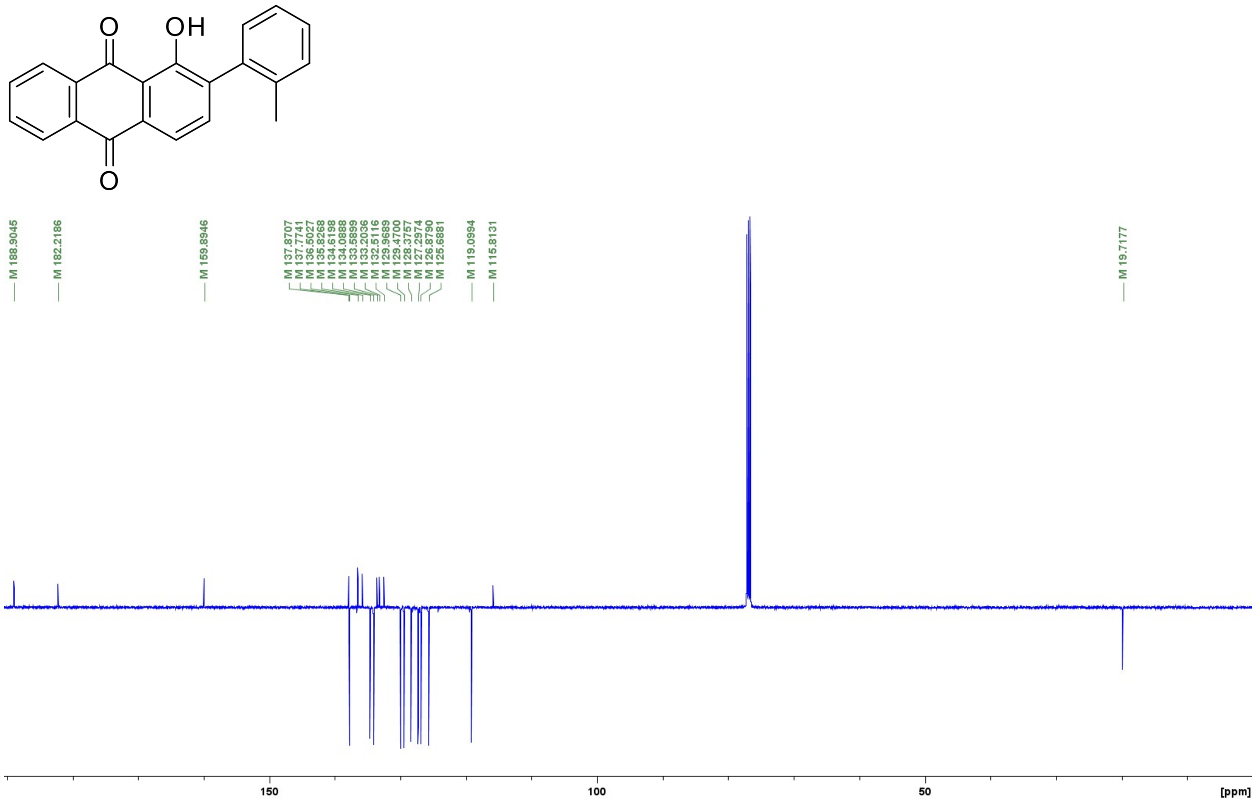

1-Hydroxy-2-(4-methoxyphenyl)anthracene-9,10-dione (27)

^{1}H NMR ($CDCl_3$, 400 MHz)

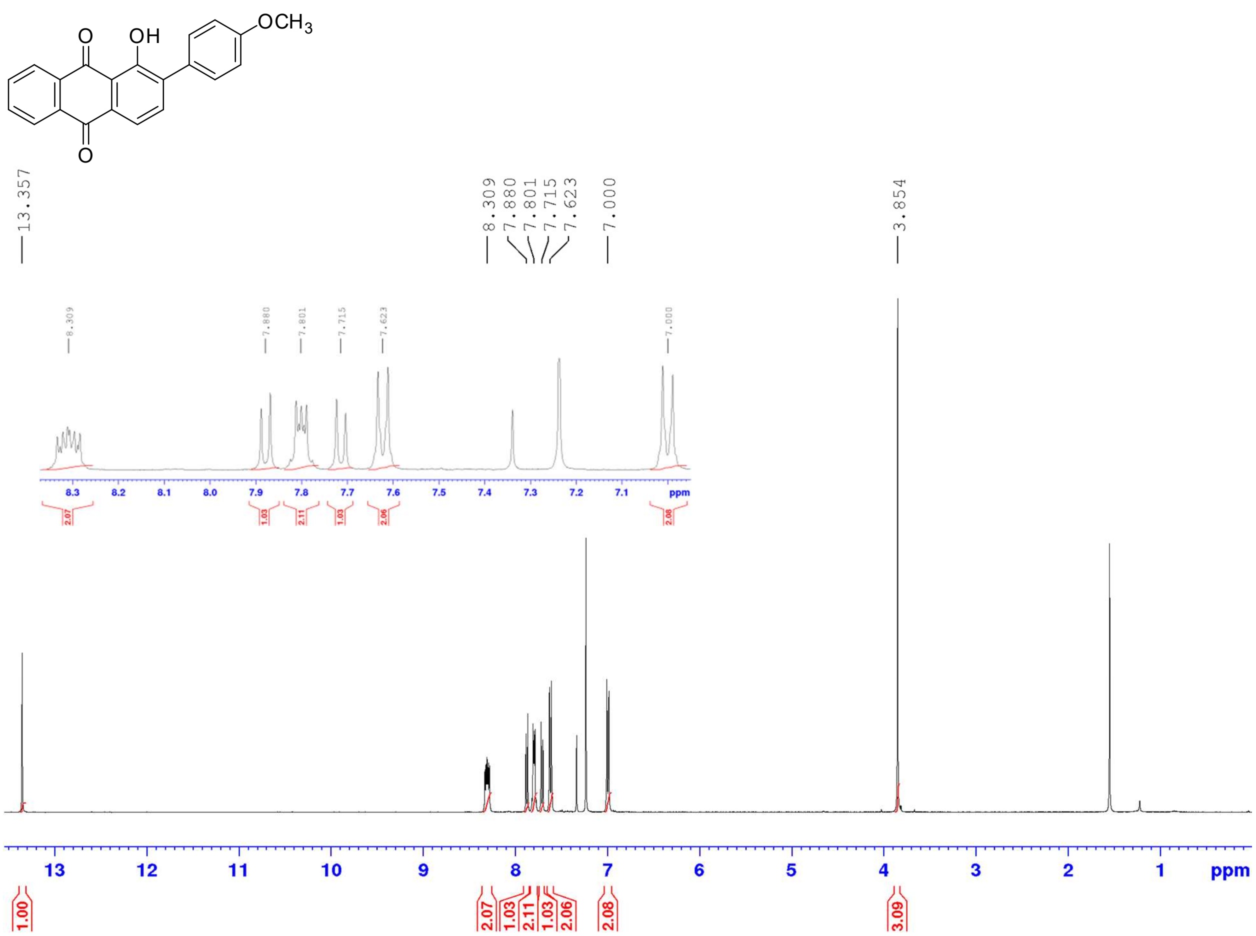

^{13}C NMR ($CDCl_3$, 125 MHz)

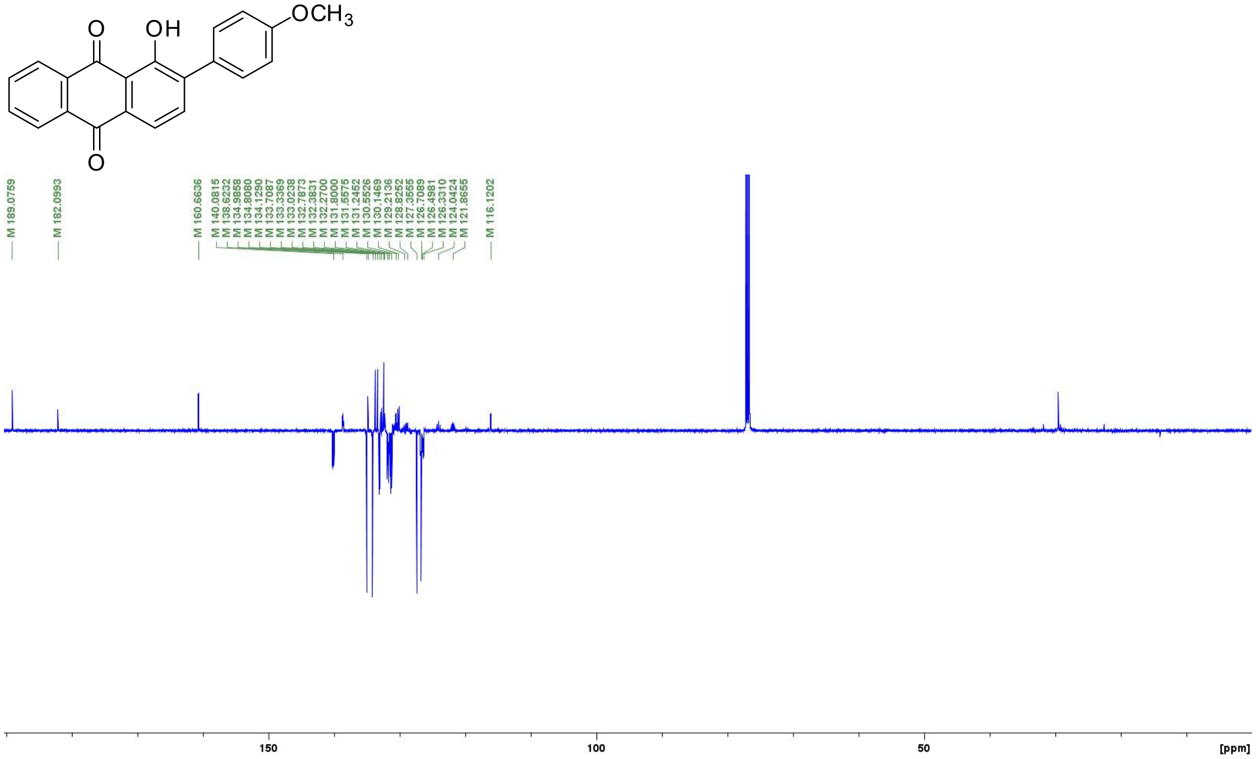

2-(2,3-Dimethoxyphenyl)-1-hydroxyanthracene-9,10-dione (28)

^{1}H NMR ($CDCl_3$, 400 MHz)

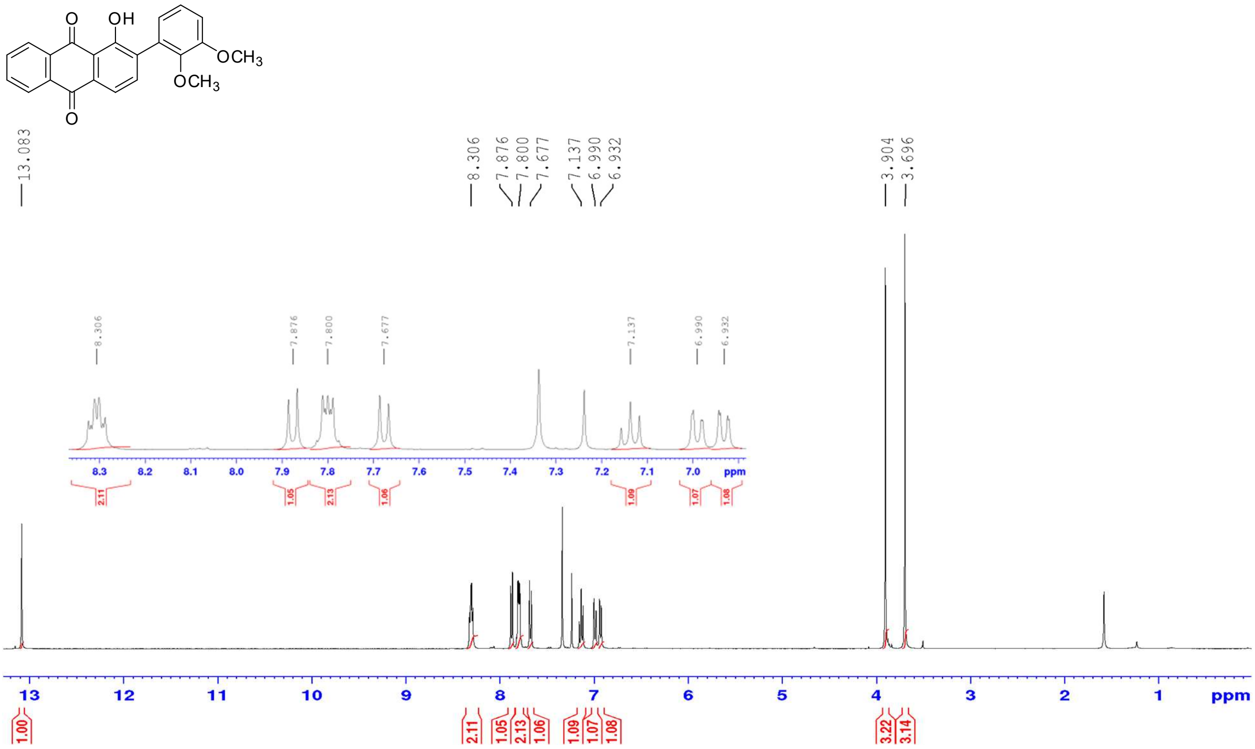

^{13}C NMR ($CDCl_3$, 75 MHz)

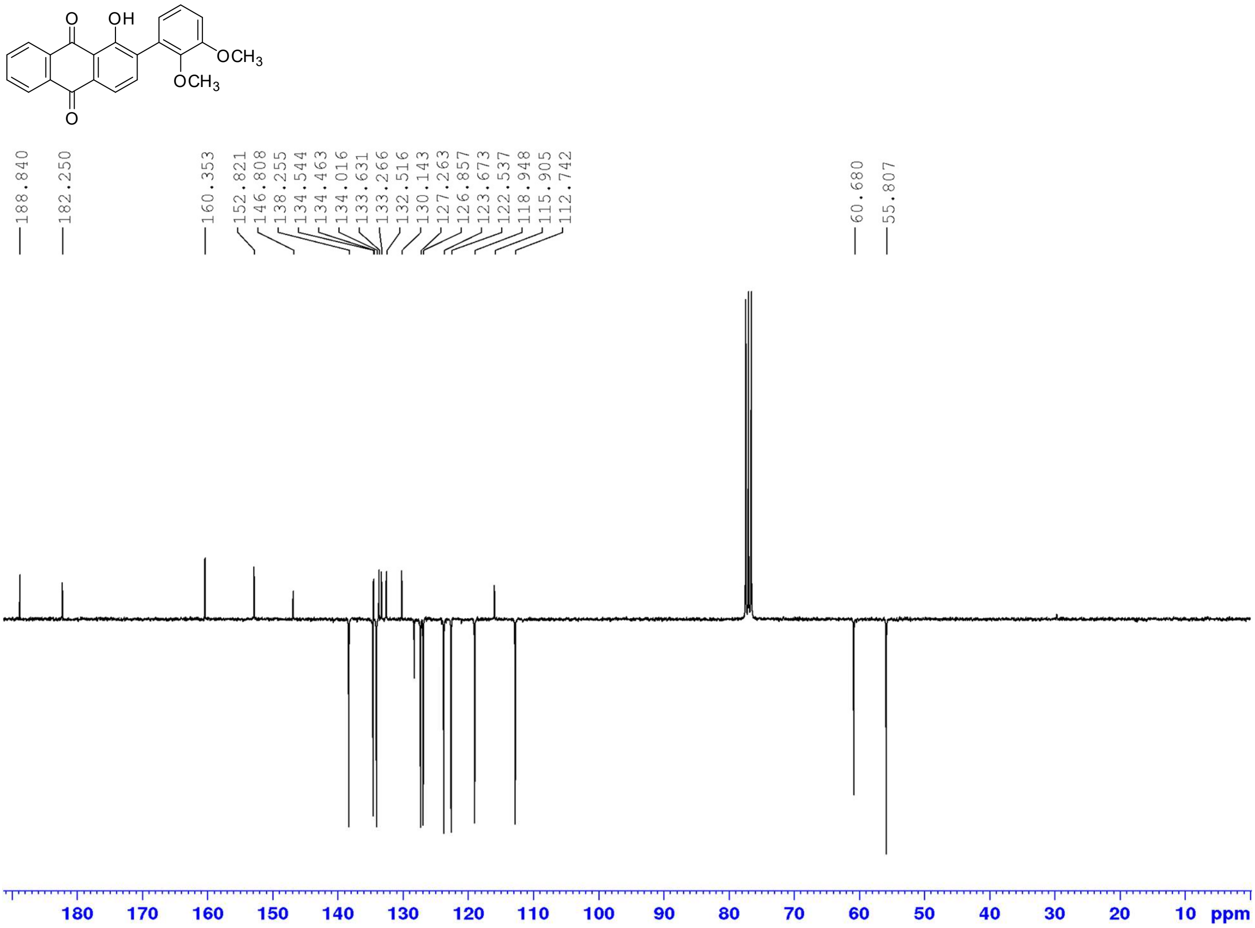

2-(3,5-Difluorophenyl)-1-hydroxyanthracene-9,10-dione (29)

^{1}H NMR ($CDCl_3$, 400 MHz)

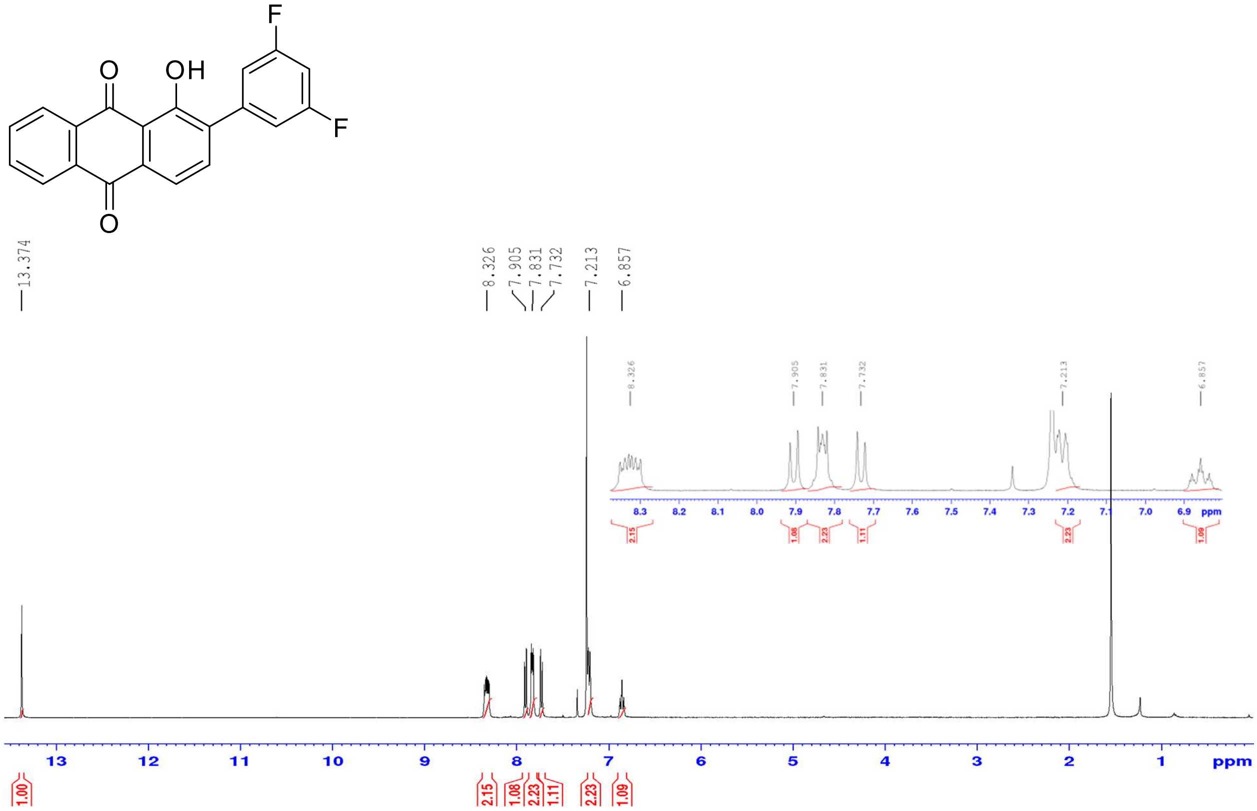

^{13}C NMR ($CDCl_3$, 125 MHz)

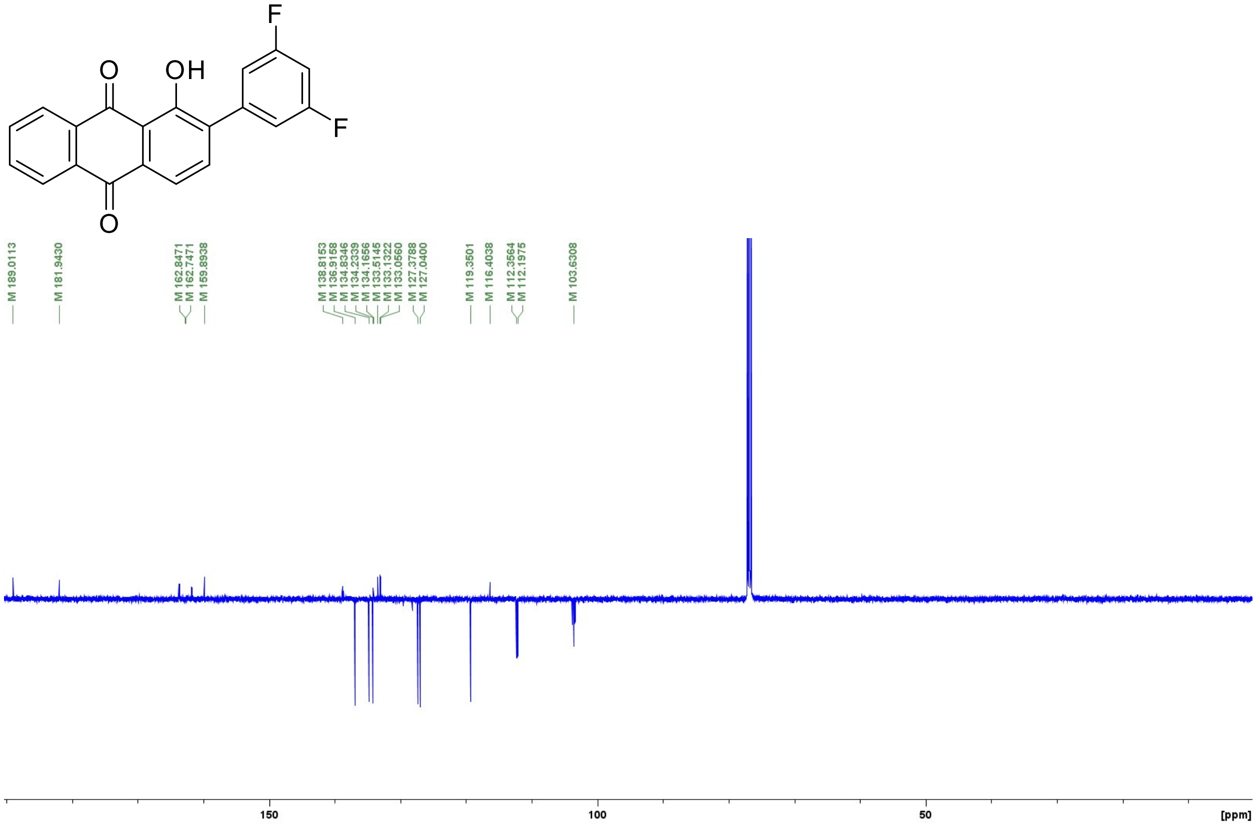

2-(2-Chloro-5-(trifluoromethyl)phenyl)-1-hydroxyanthracene-9,10-dione (30)

^{1}H NMR ($CDCl_3$, 500 MHz)

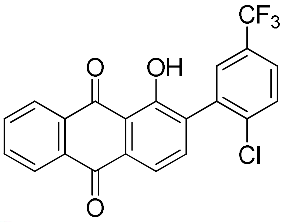

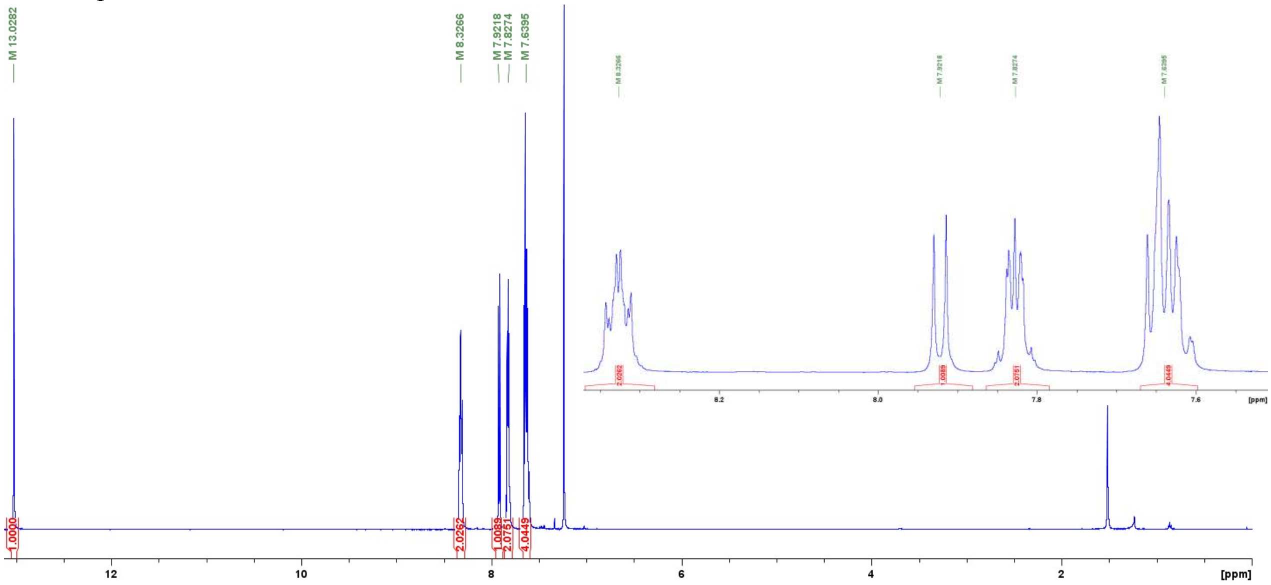

^{13}C NMR ($CDCl_3$, 125 MHz)

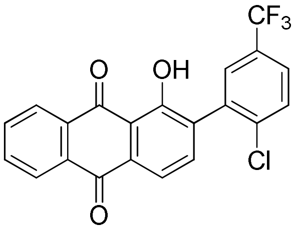

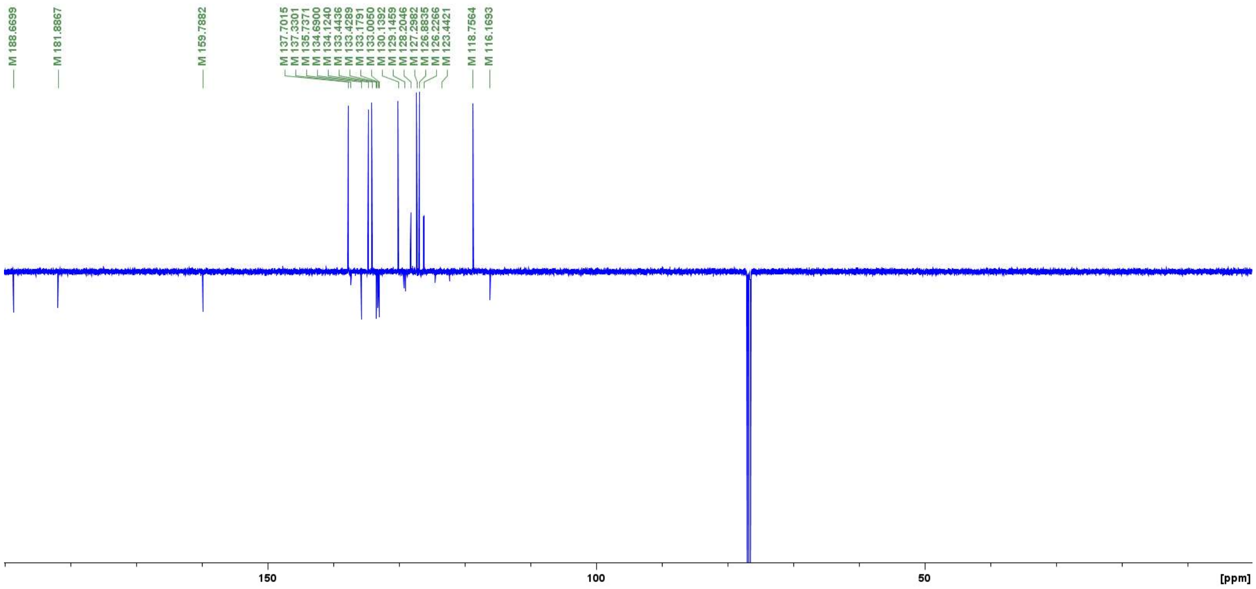

2-(4-Chloro-2-(trifluoromethyl)phenyl)-1-hydroxyanthracene-9,10-dione (31)

^{1}H NMR ($CDCl_3$, 400 MHz)

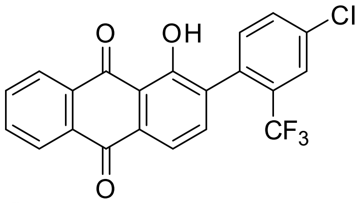

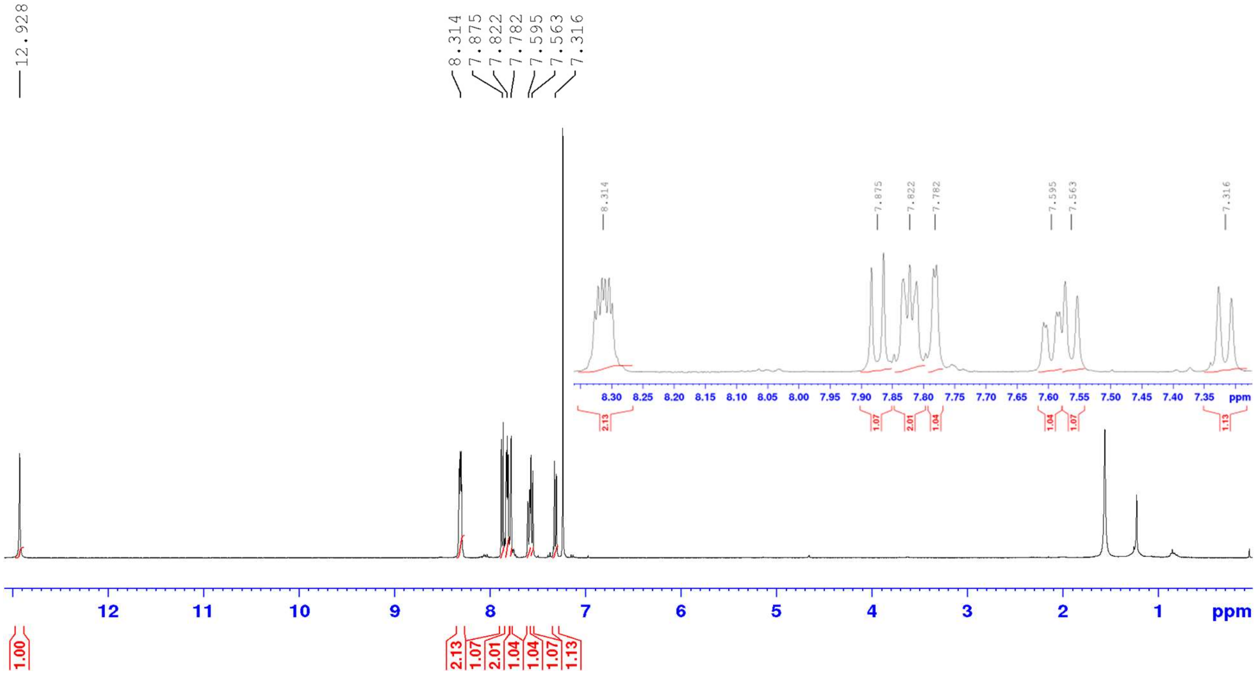

^{13}C NMR ($CDCl_3$, 125 MHz)

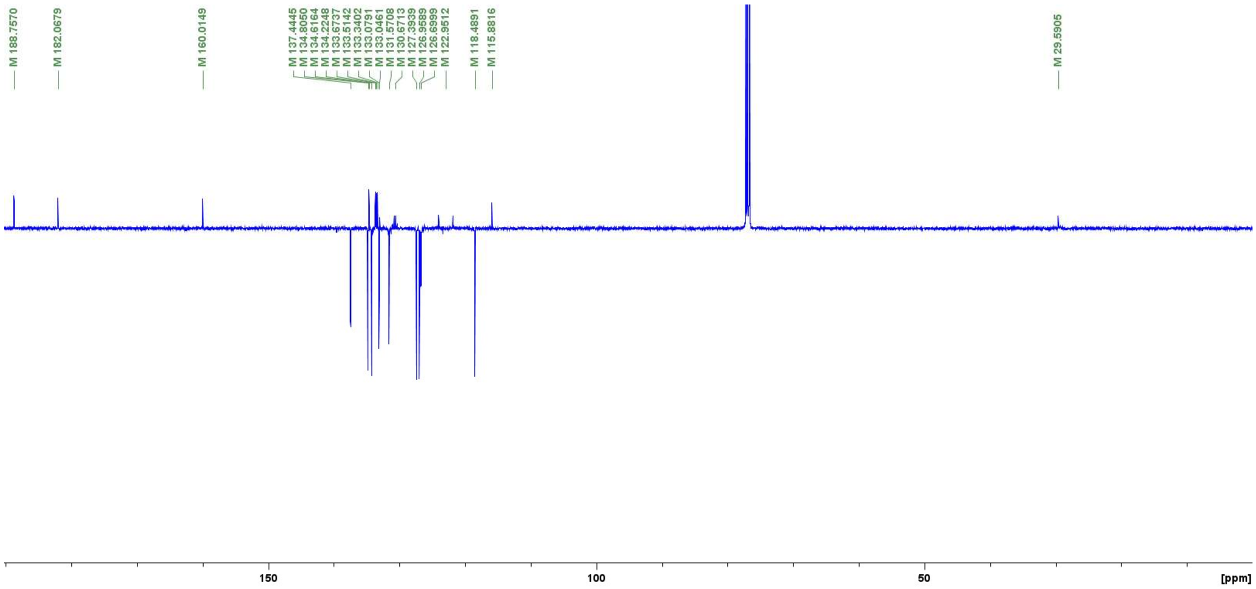

1-Hydroxy-2,4-di-(3,4,5-trimethoxyphenyl)anthracene-9,10-dione (32)

^{1}**H NMR** ($CDCl_3$, 400 MHz)

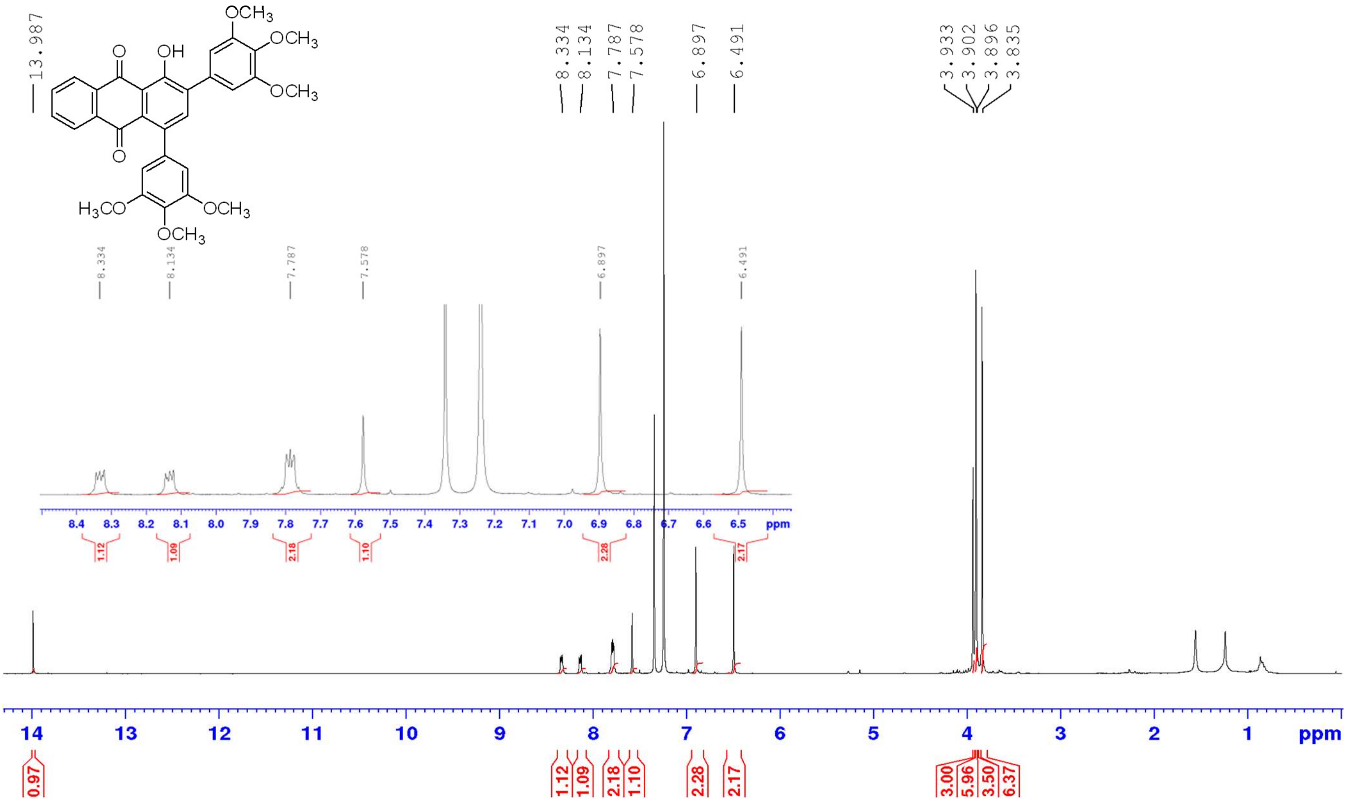

^{13}**C NMR** ($CDCl_3$, 100 MHz)

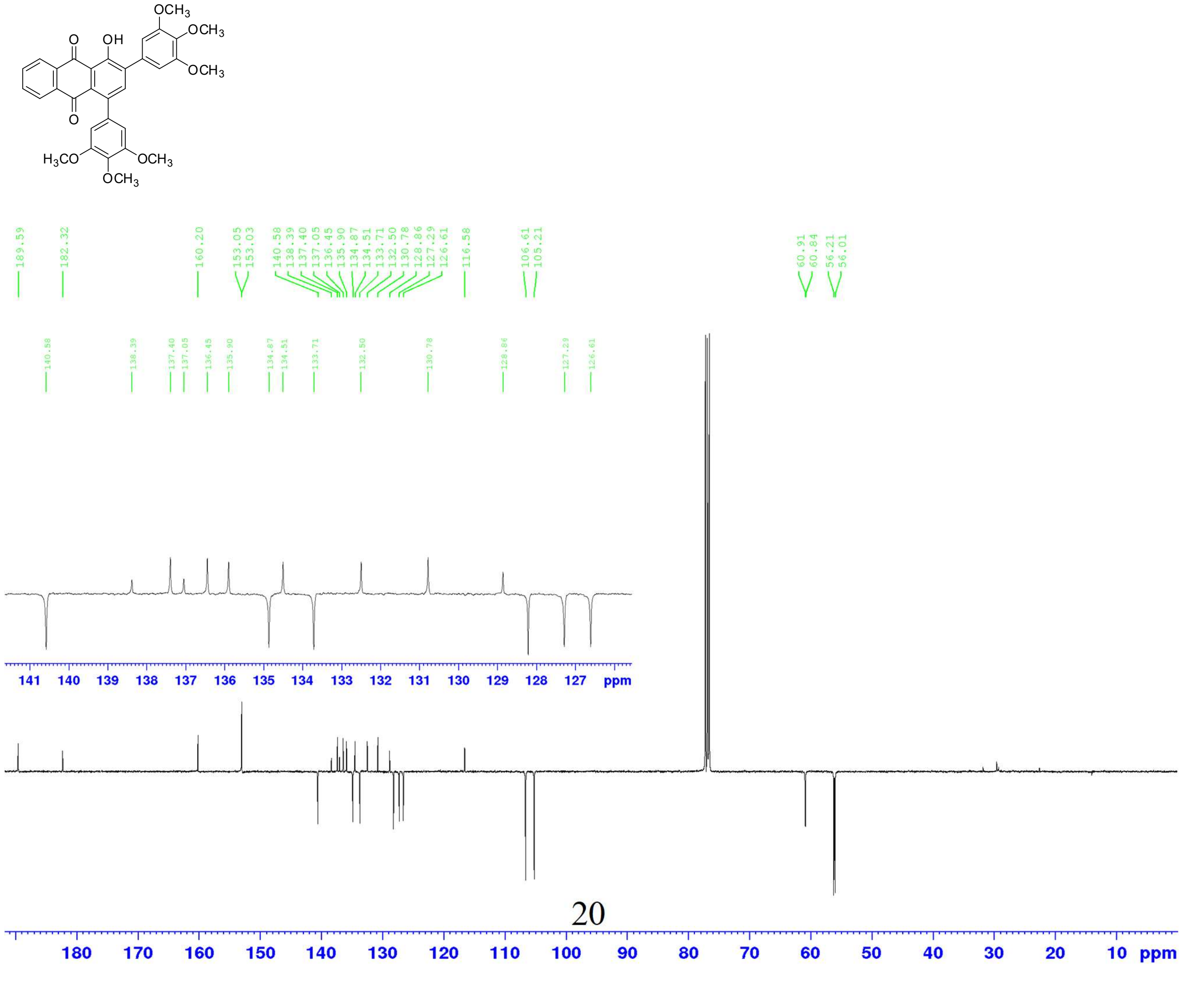

1-Hydroxy-2,4-diphenylanthracene-9,10-dione (33)

^{1}H NMR ($CDCl_3$, 400 MHz)

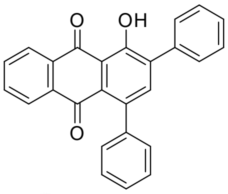

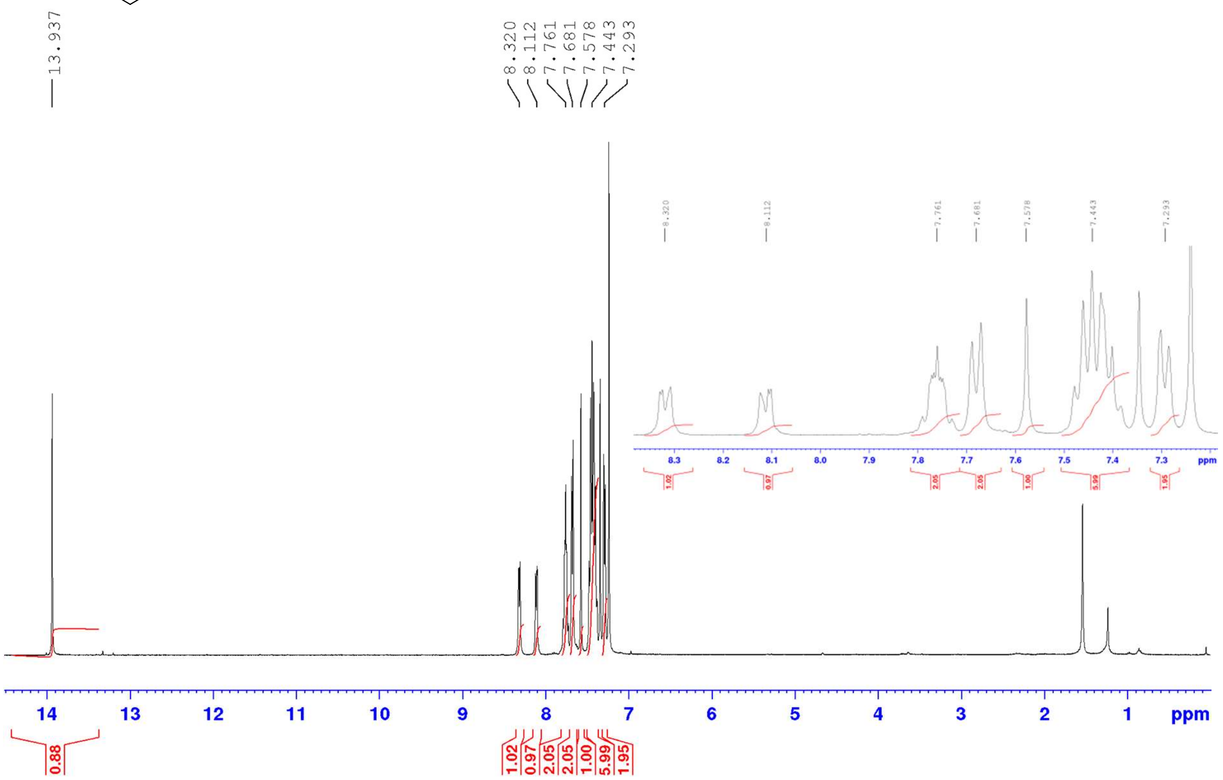

^{13}C NMR ($CDCl_3$, 125 MHz)

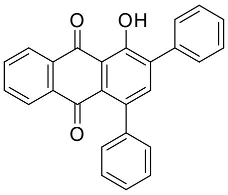

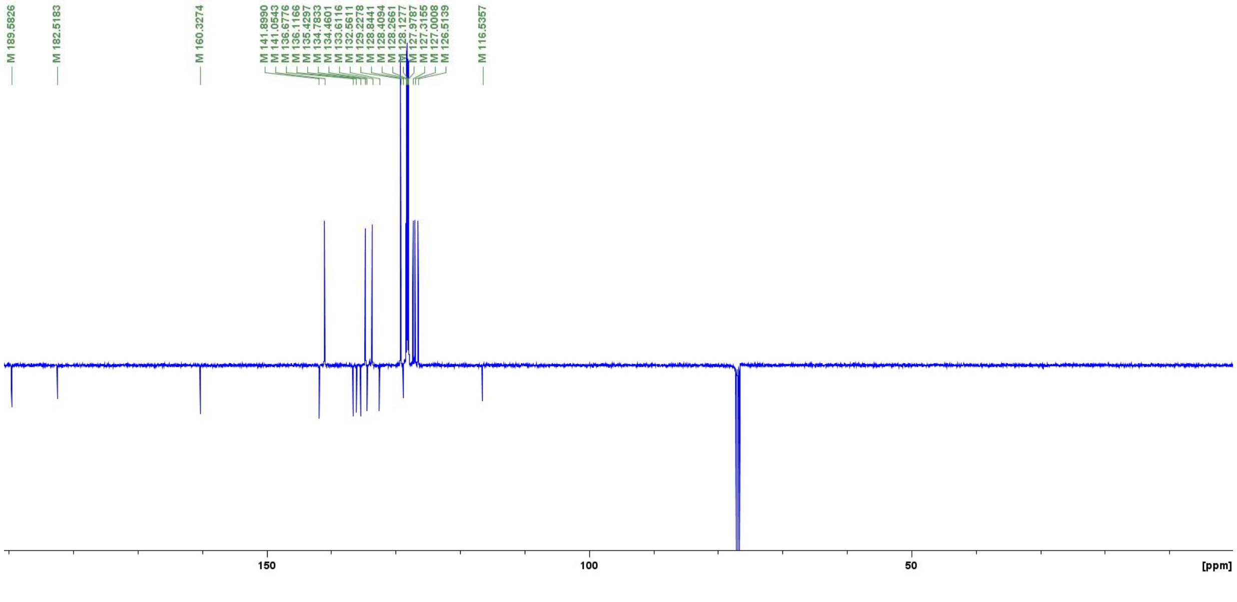

1-Hydroxy-2,4-di-(*o*-tolyl)anthracene-9,10-dione (34)

^{1}H NMR ($CDCl_3$, 400 MHz)

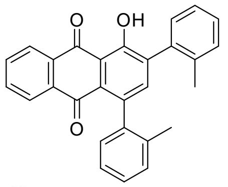

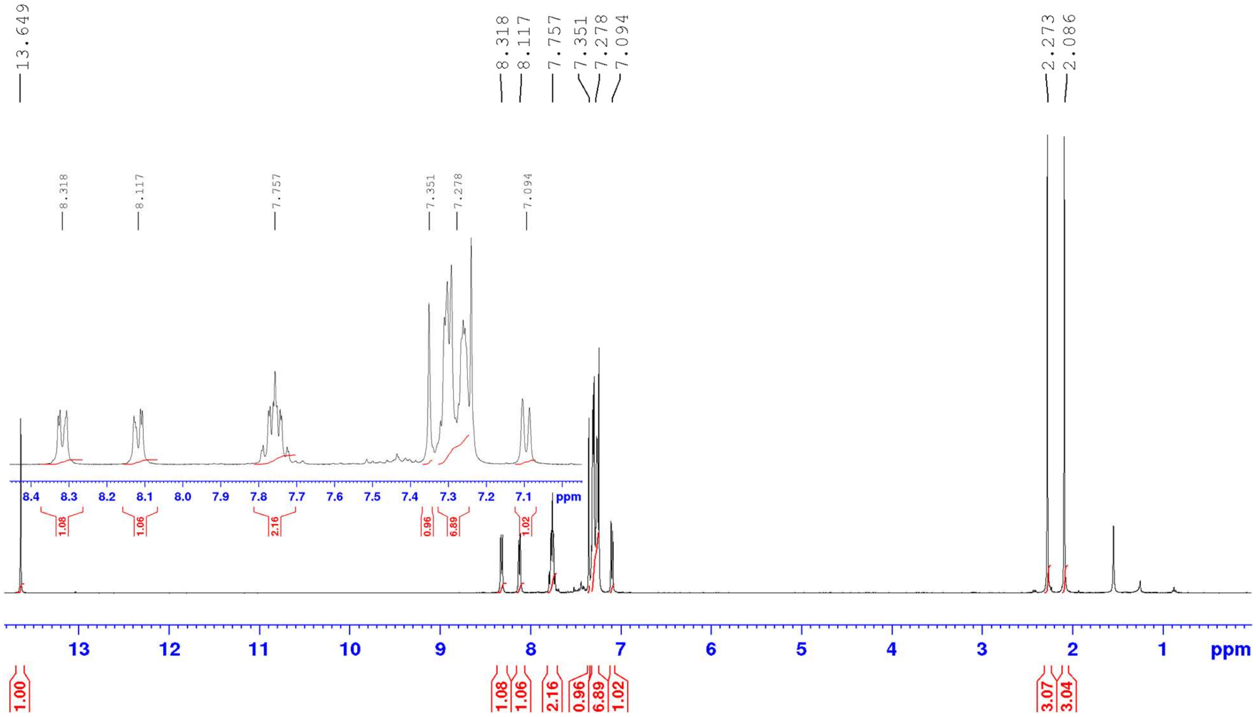

^{13}C NMR ($CDCl_3$, 125 MHz)

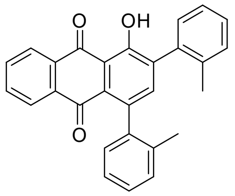

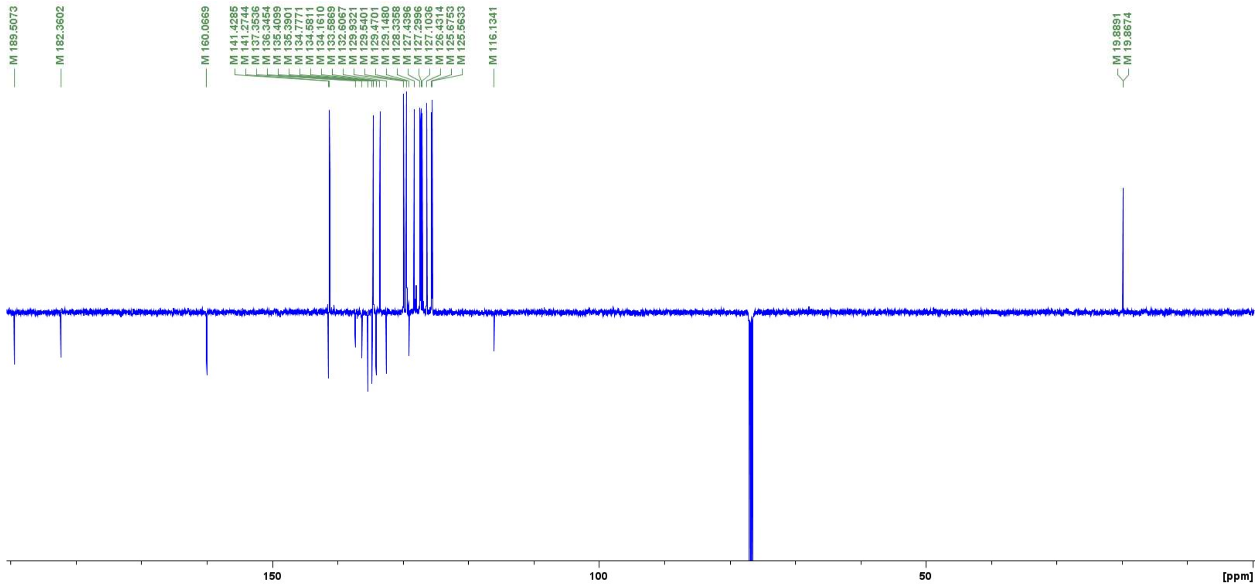

1-Hydroxy-2,4-di-(4-methoxyphenyl)anthracene-9,10-dione (35)

^{1}H NMR ($CDCl_3$, 400 MHz)

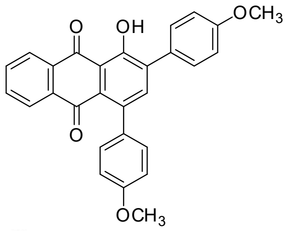

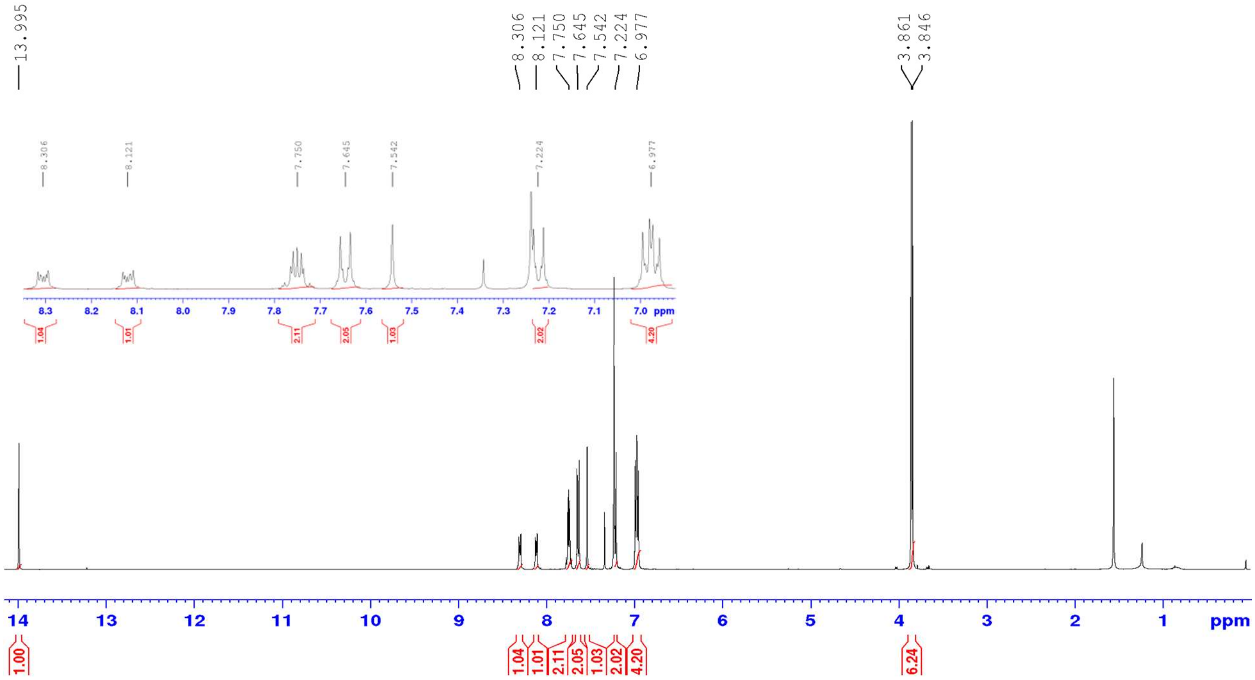

^{13}C NMR ($CDCl_3$, 125 MHz)

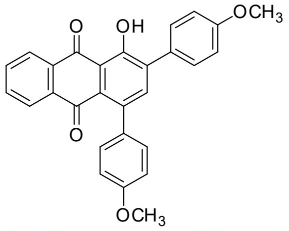

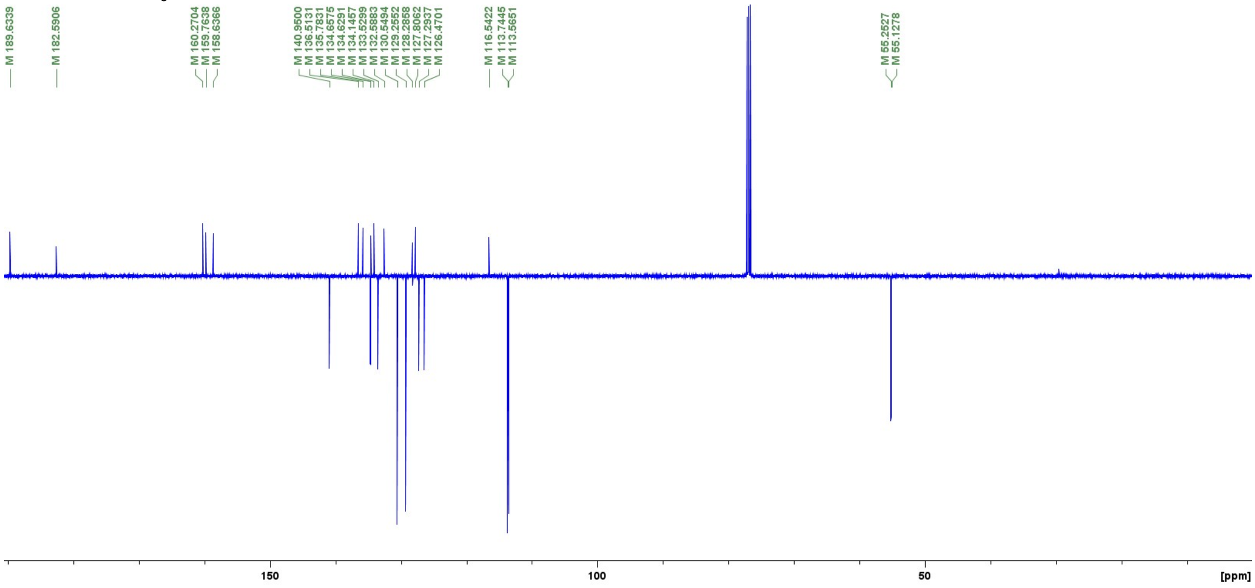

2,4-Di-(2,3-dimethoxyphenyl)-1-hydroxyanthracene-9,10-dione (36)

^{1}H NMR ($CDCl_3$, 400 MHz)

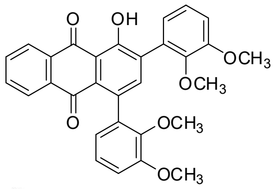

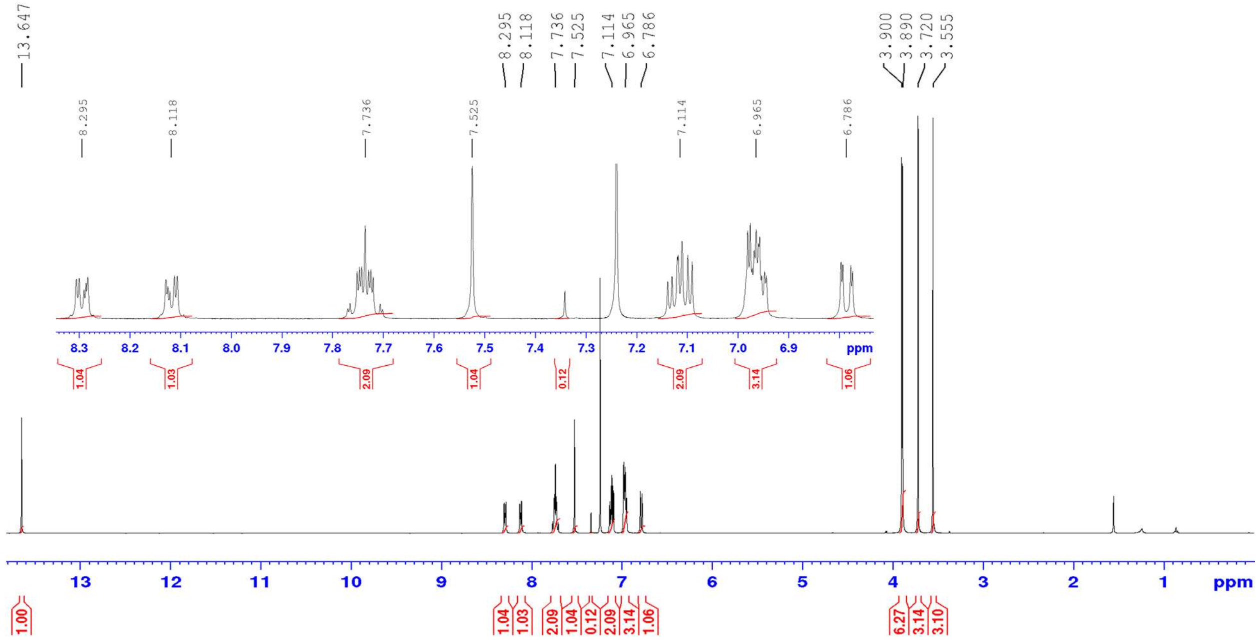

^{13}C NMR ($CDCl_3$, 100 MHz)

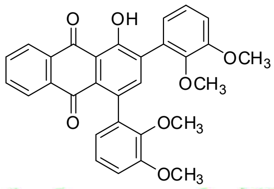

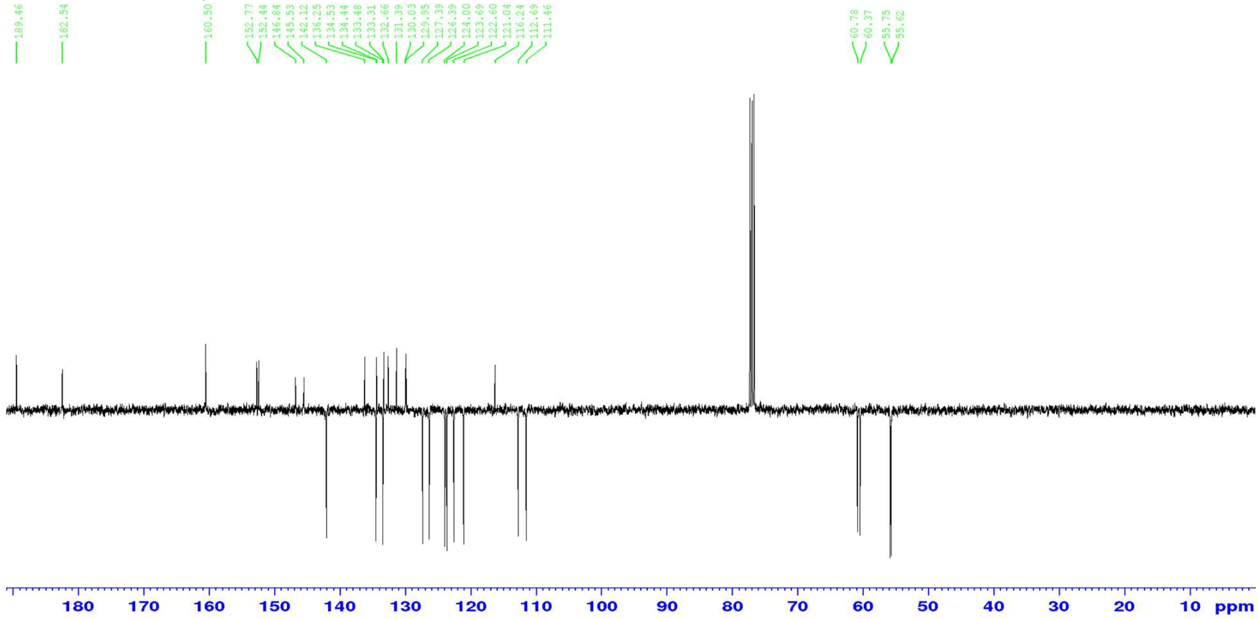

2,4-Di-(3,5-difluorophenyl)-1-hydroxyanthracene-9,10-dione (37)

^{1}H NMR ($CDCl_3$, 500 MHz)

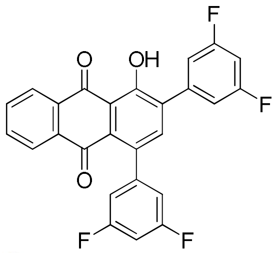

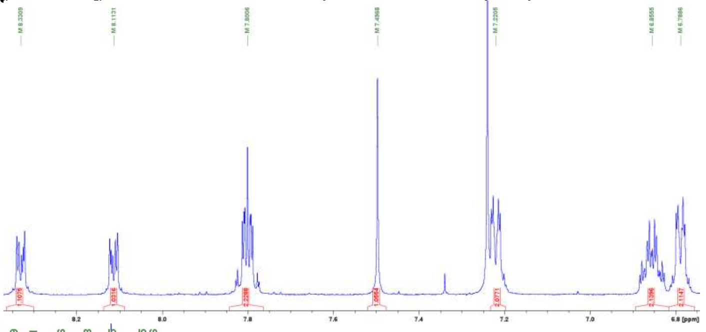

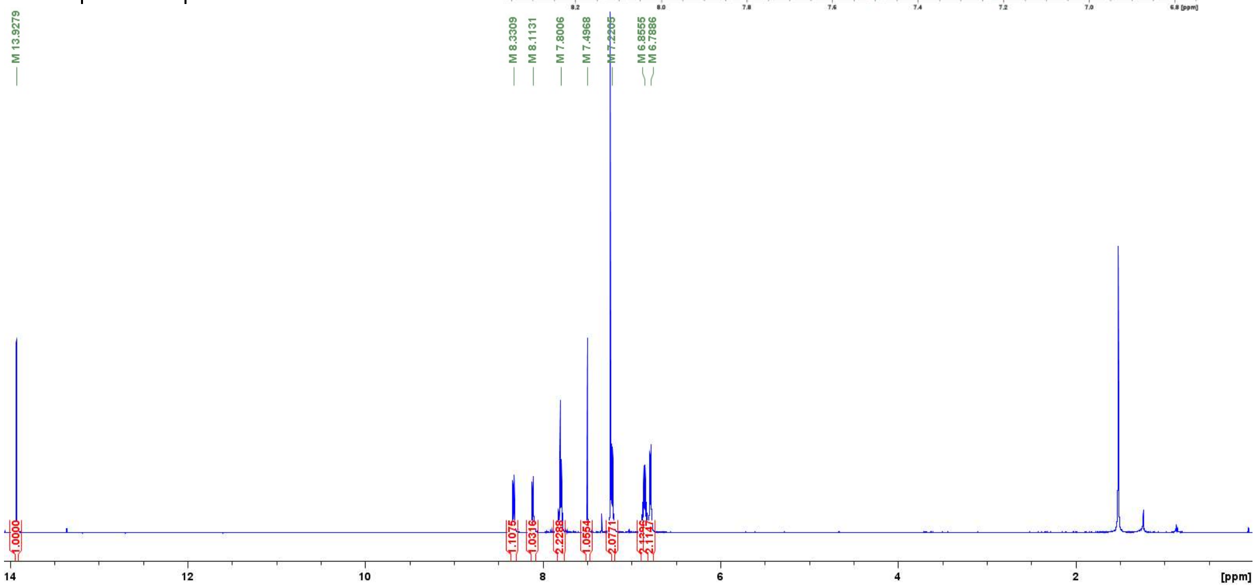

^{13}C NMR ($CDCl_3$, 125 MHz)

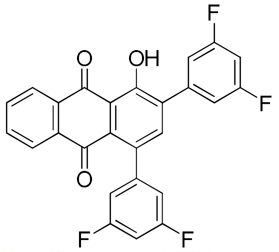

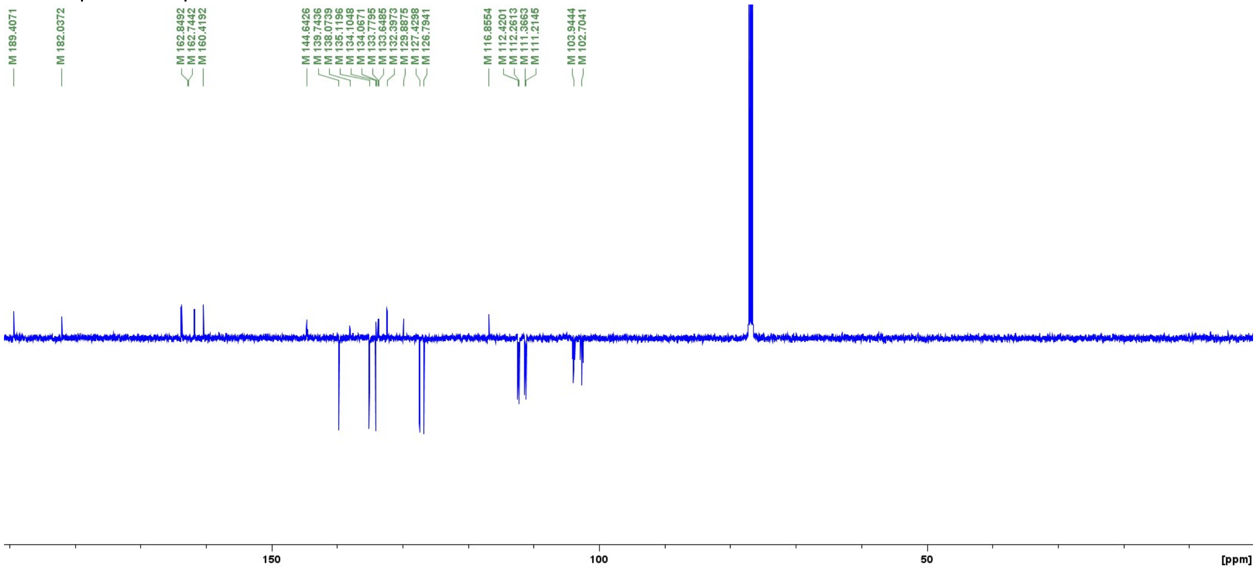

2,4-Di-(2-chloro-5-(trifluoromethyl)phenyl)-1-hydroxyanthracene-9,10-dione (38)

^{1}H NMR ($CDCl_3$, 400 MHz)

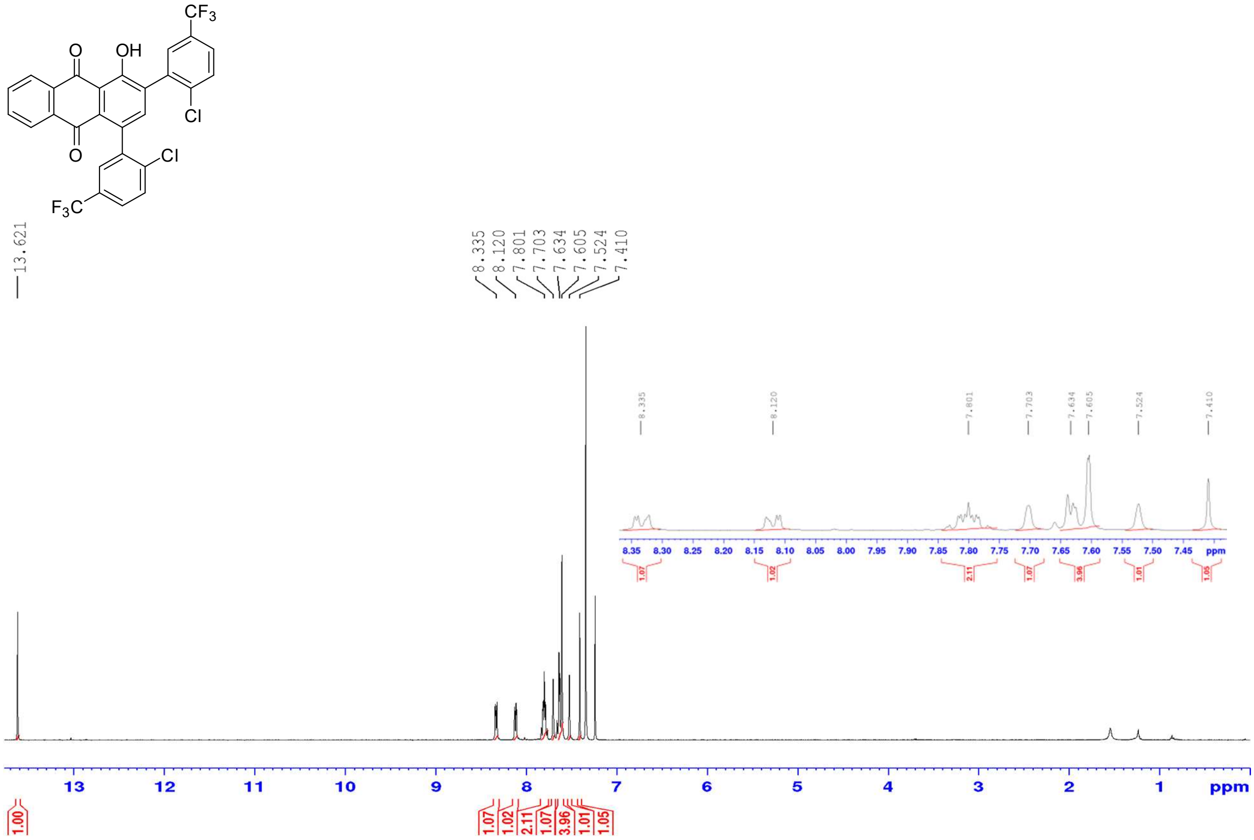

^{13}C NMR ($CDCl_3$, 125 MHz)

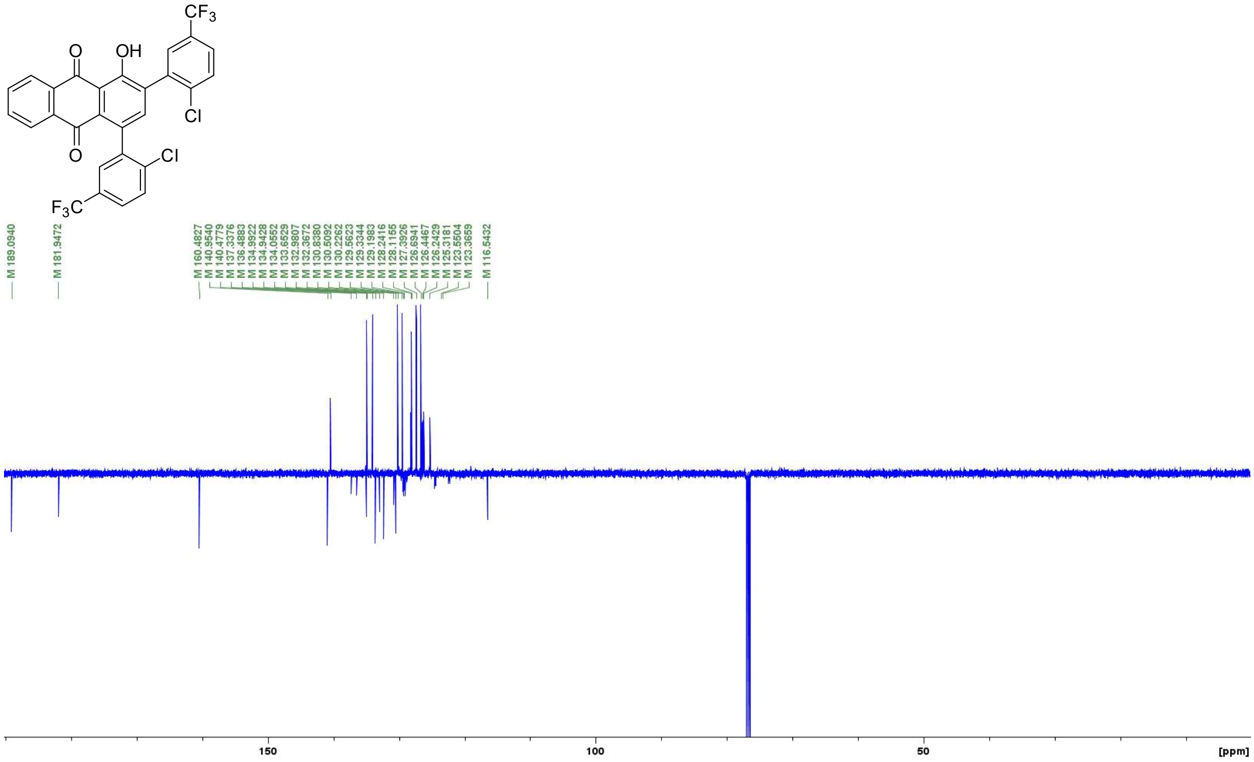

2,4-Di-(4-chloro-2-(trifluoromethyl)phenyl)-1-hydroxyanthracene-9,10-dione (39)

^{1}H NMR ($CDCl_3$, 400 MHz)

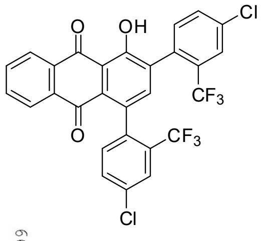

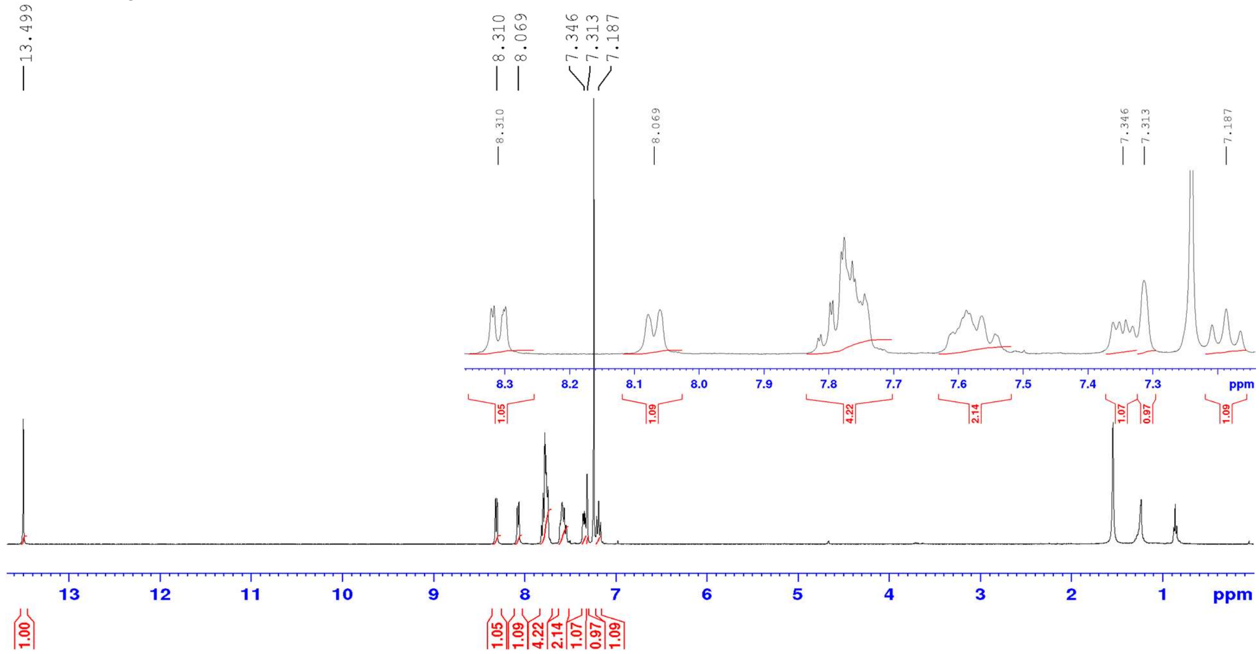

^{13}C NMR ($CDCl_3$, 125 MHz)

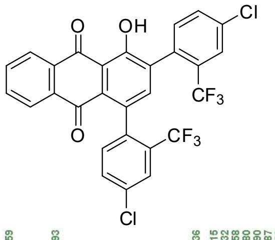

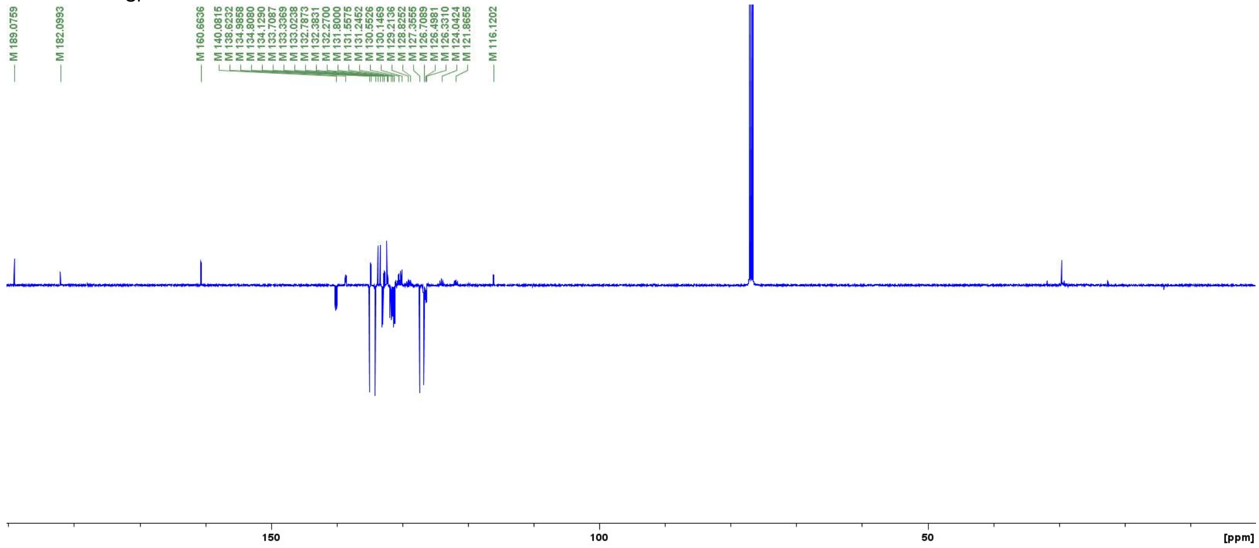

4-bromo-1-hydroxy-2-(4-methoxyphenyl)anthracene-9,10-dione (40)

^{1}H NMR ($CDCl_3$, 400 MHz)

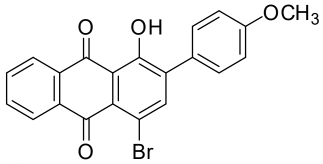

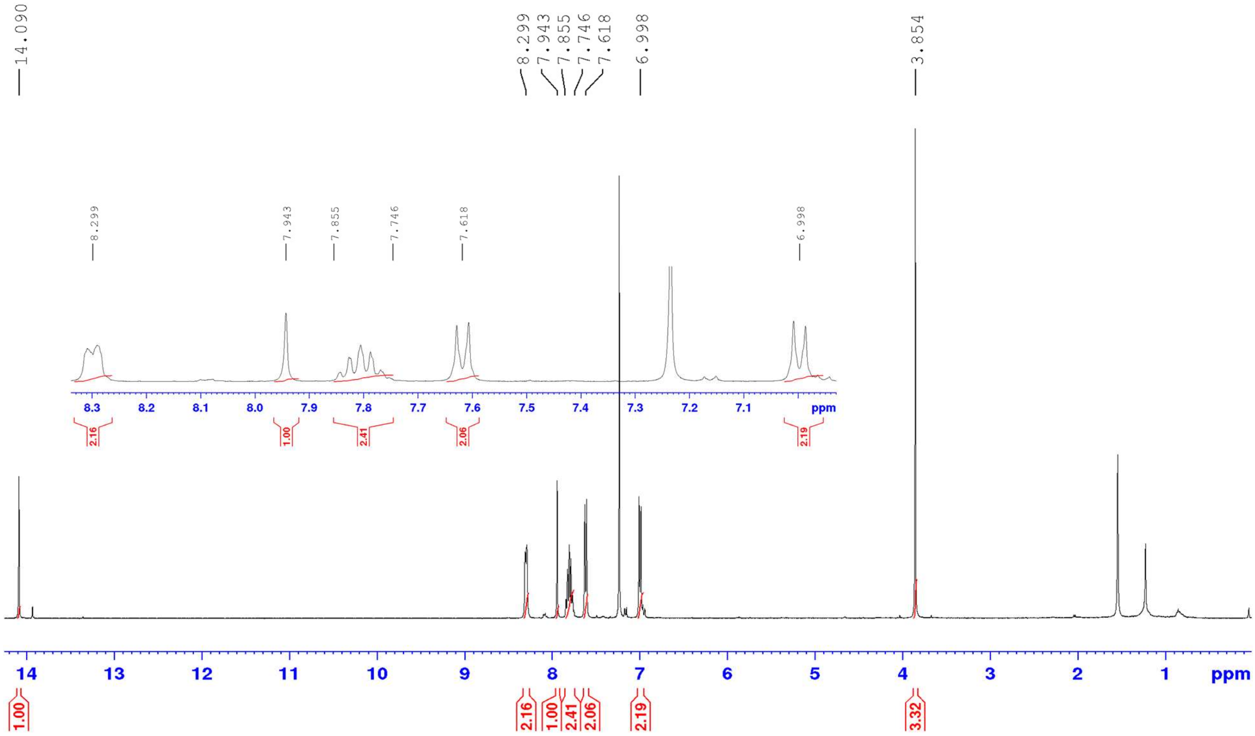

^{13}C NMR ($CDCl_3$, 150MHz)

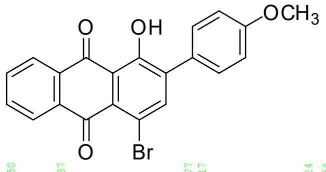

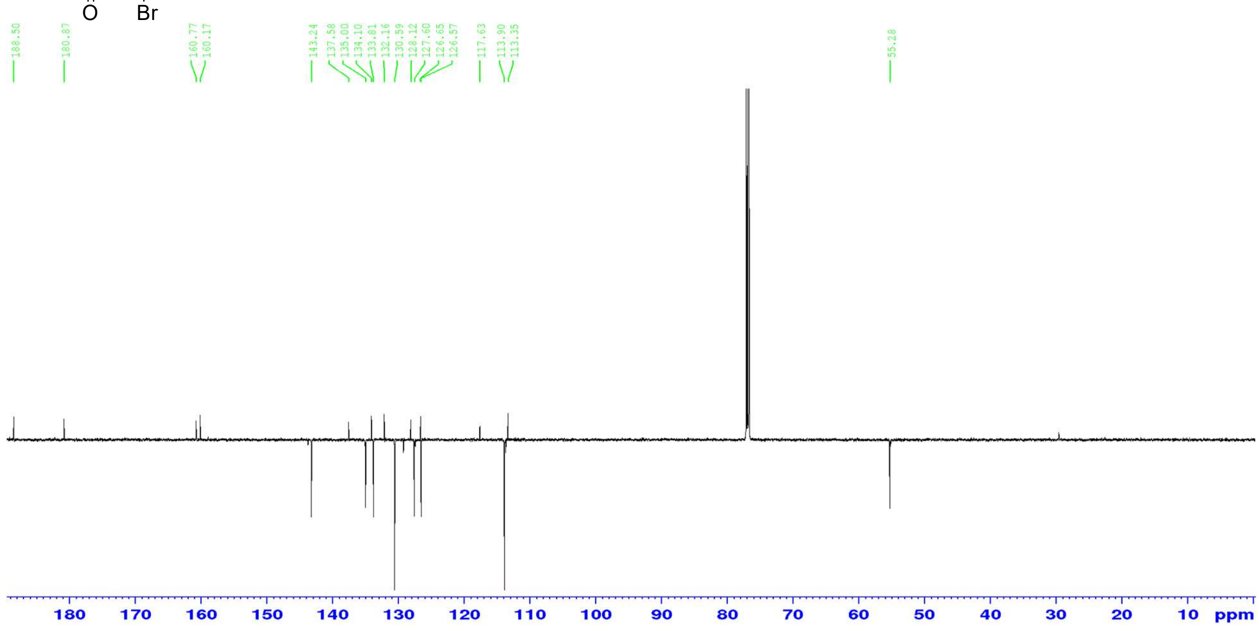

2-bromo-1-hydroxy-4-(4-methoxyphenyl)anthracene-9,10-dione (41)

^{1}H NMR ($CDCl_3$, 400 MHz)

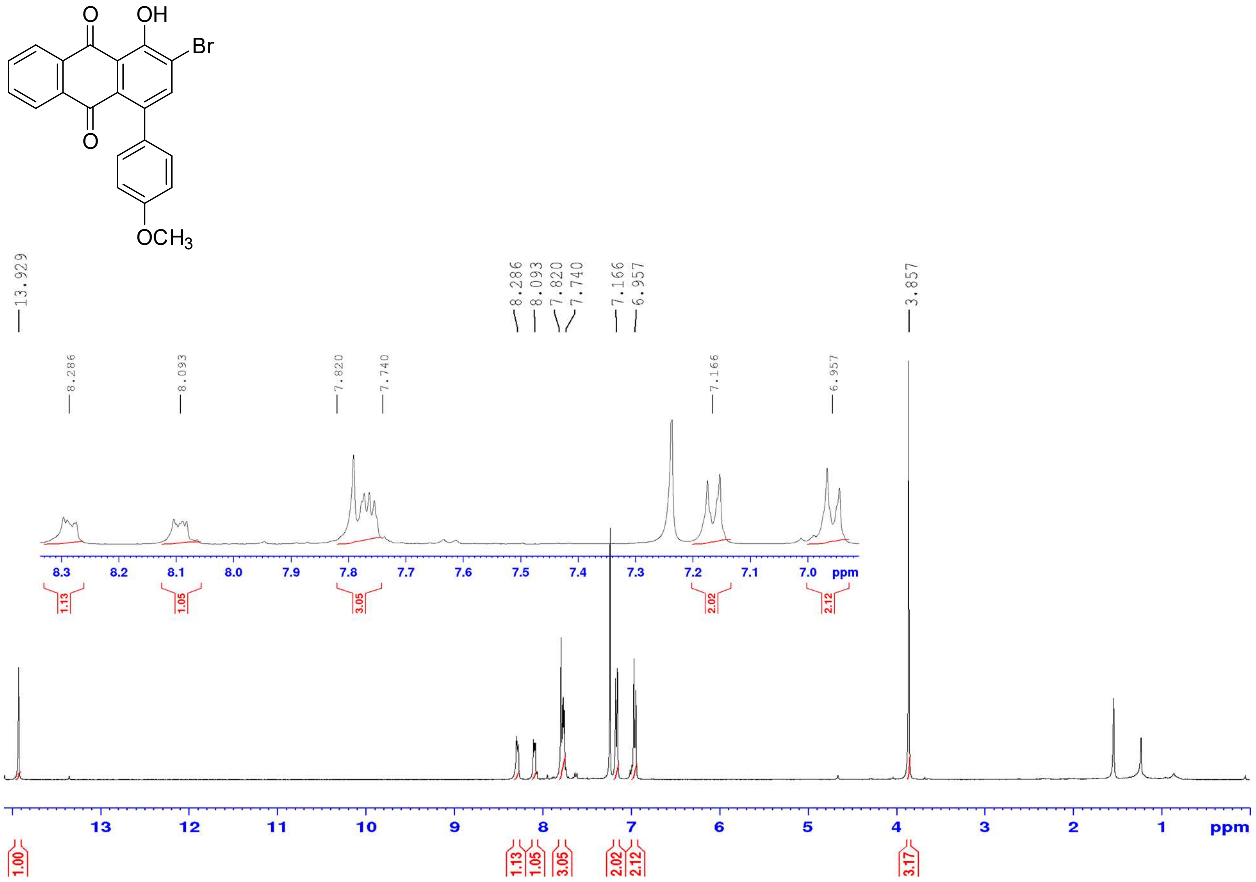

^{13}C NMR ($CDCl_3$, 150MHz)

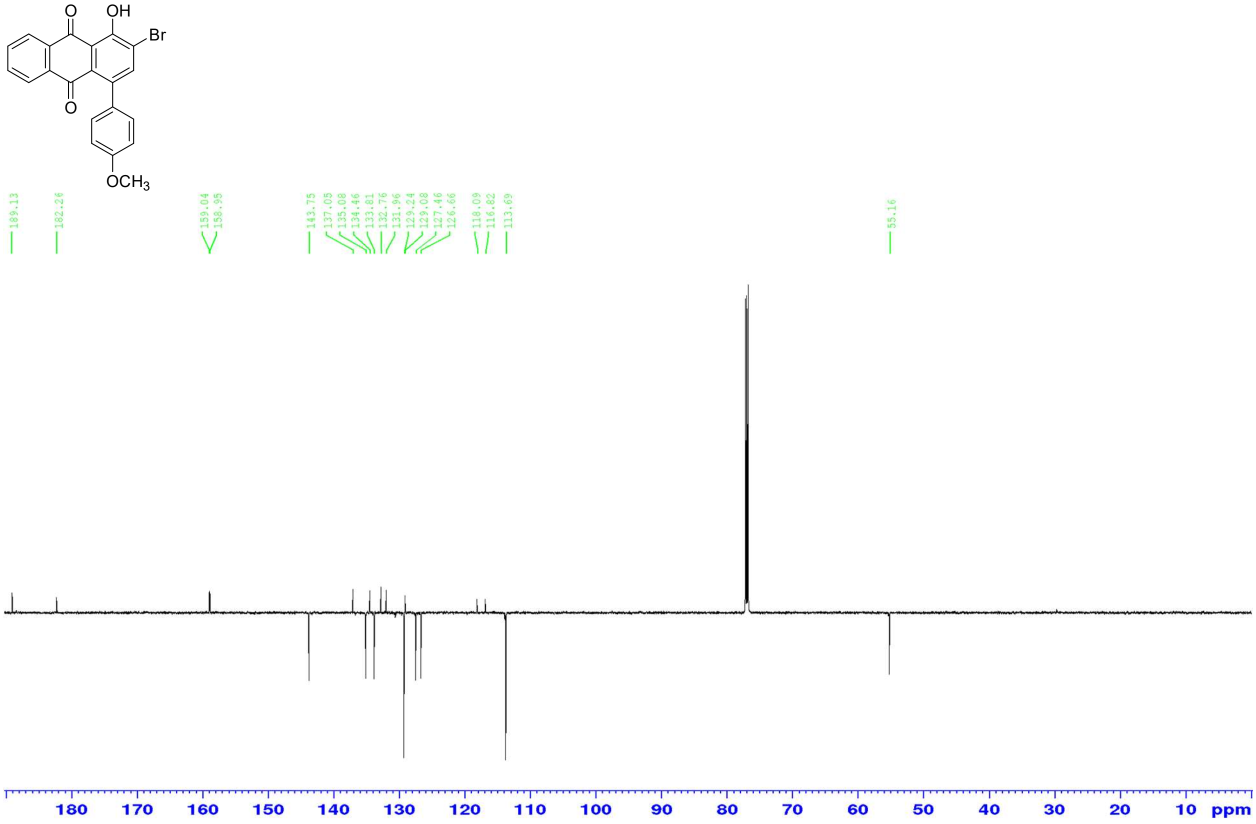

4-Bromo-1-hydroxy-2-(3,4,5-trimethoxyphenyl)anthracene-9,10-dione (42)

^{1}H NMR ($CDCl_3$, 400 MHz)

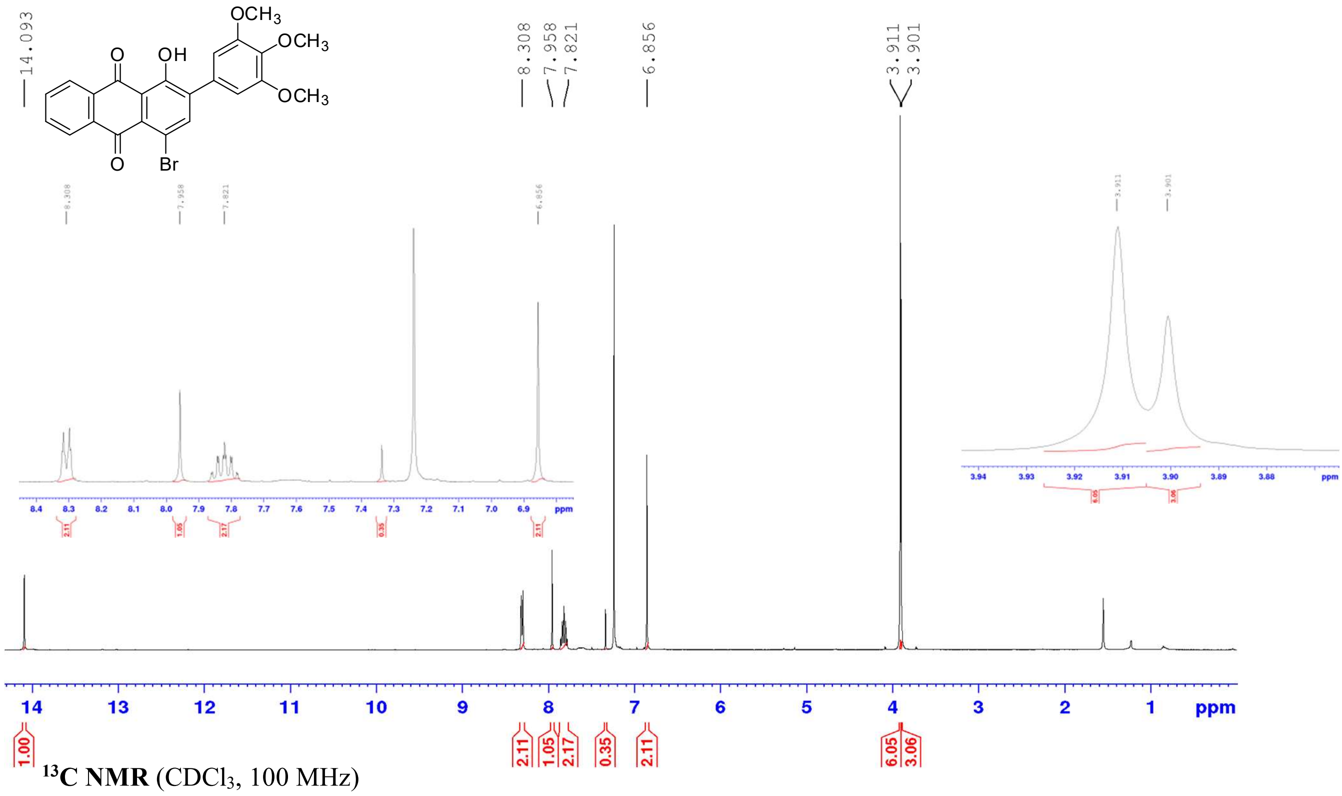

^{13}C NMR ($CDCl_3$, 100 MHz)

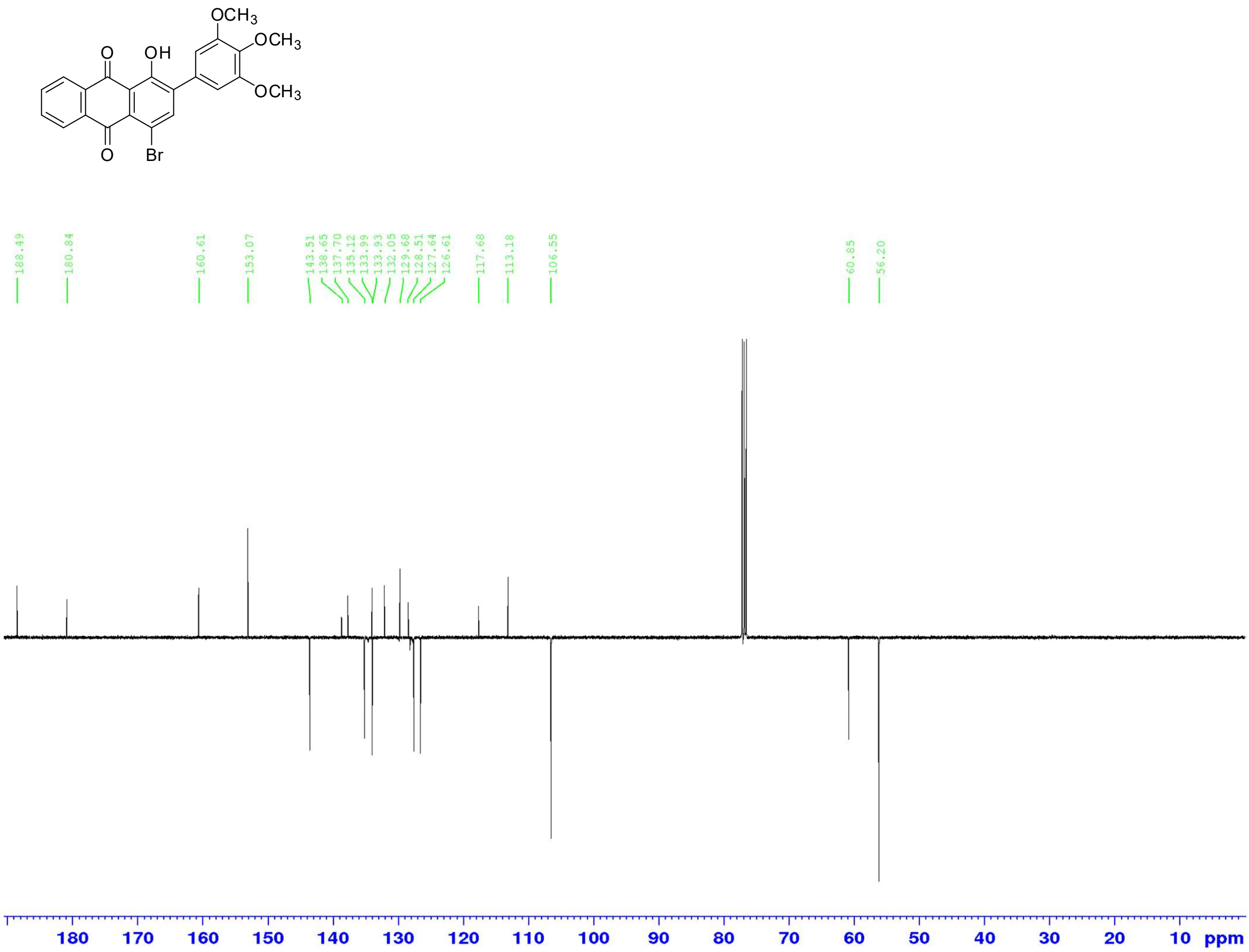

1-Hydroxy-4-phenyl-2-(3,4,5-trimethoxyphenyl)anthracene-9,10-dione (43)

^{1}H NMR ($CDCl_3$, 400 MHz)

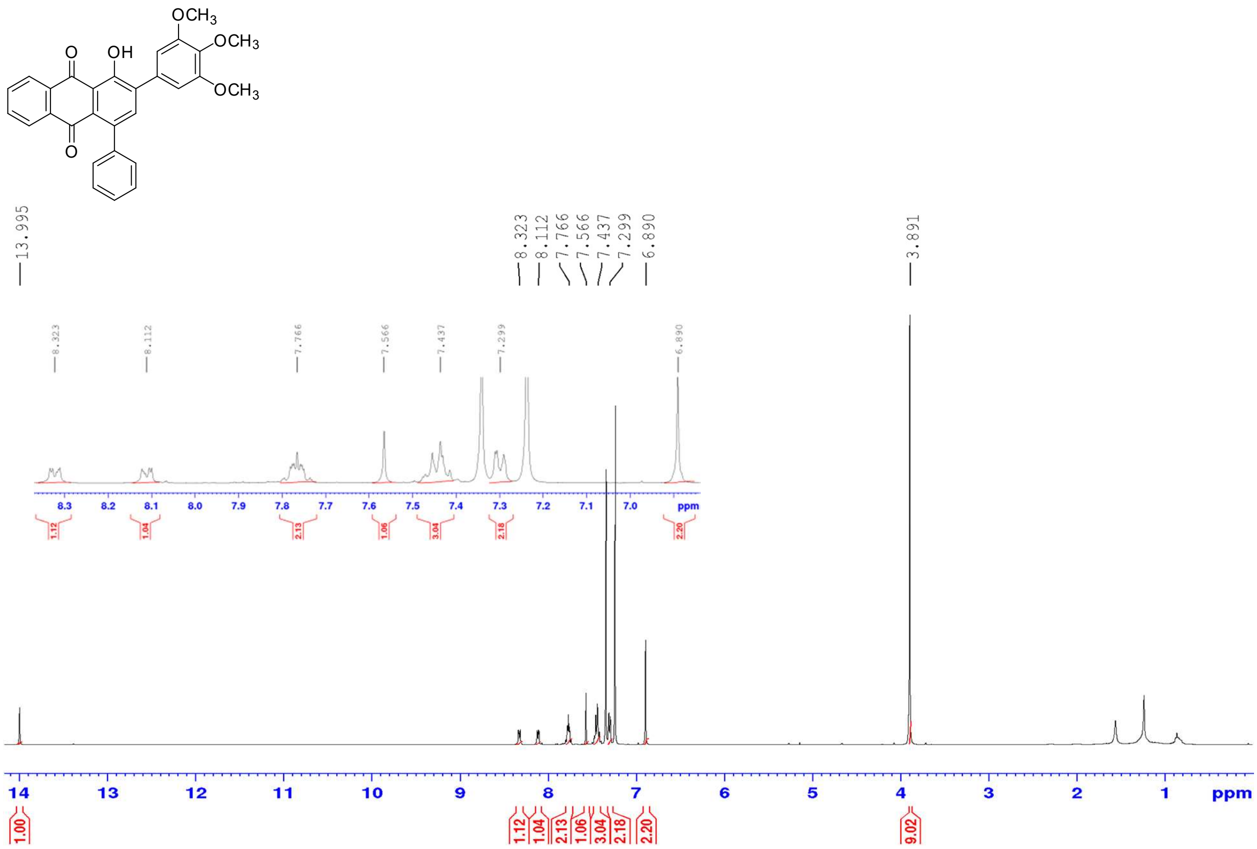

^{13}C NMR ($CDCl_3$, 125 MHz)

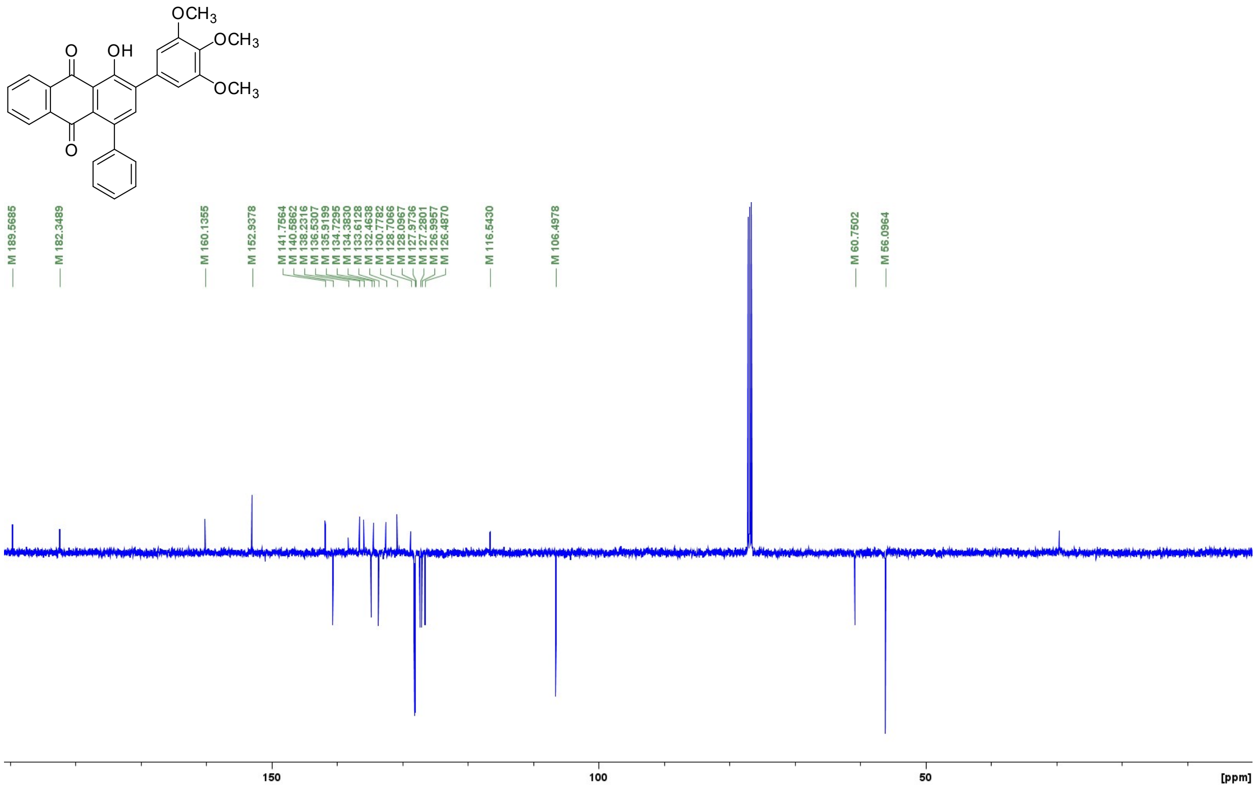

1-Hydroxy-4-(4-methoxyphenyl)-2-(3,4,5-trimethoxyphenyl)anthracene-9,10-dione (44)

^{1}H NMR ($CDCl_3$, 400 MHz)

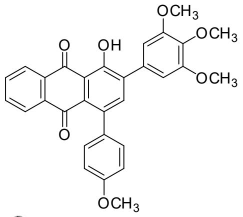

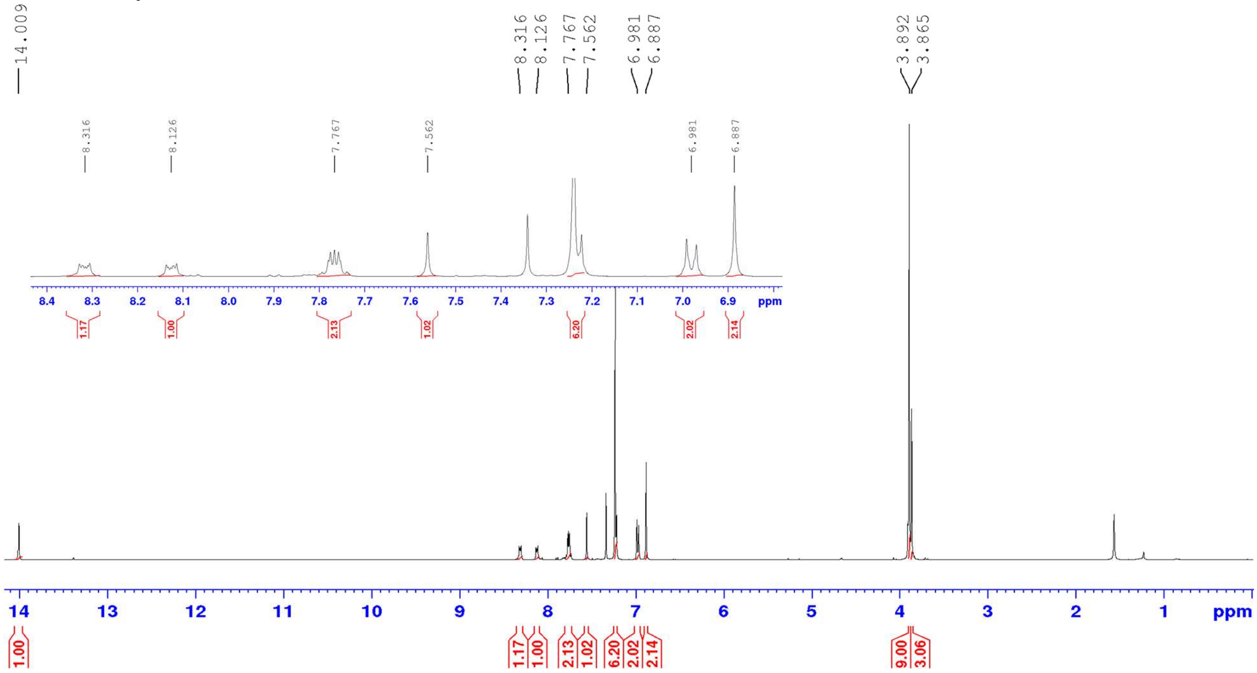

^{13}C NMR ($CDCl_3$, 125 MHz)

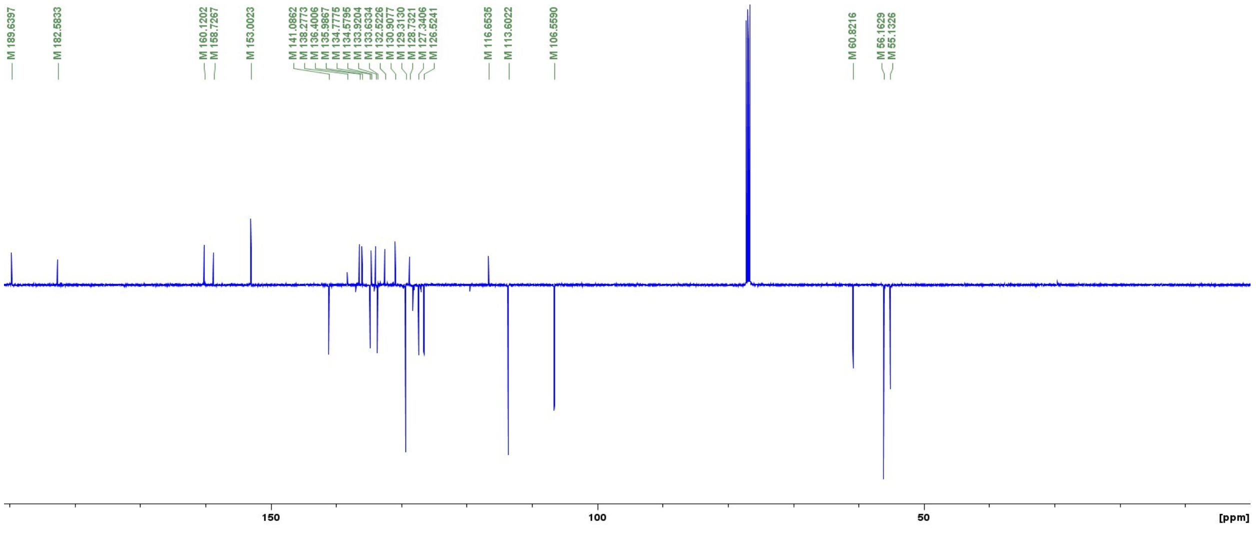

4-(4-Chloro-2-(trifluoromethyl)phenyl)-1-hydroxy-2-(3,4,5-trimethoxyphenyl)anthracene-9,10-dione (45)

^{1}H NMR ($CDCl_3$, 400 MHz)

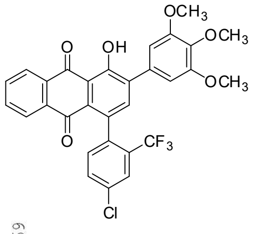

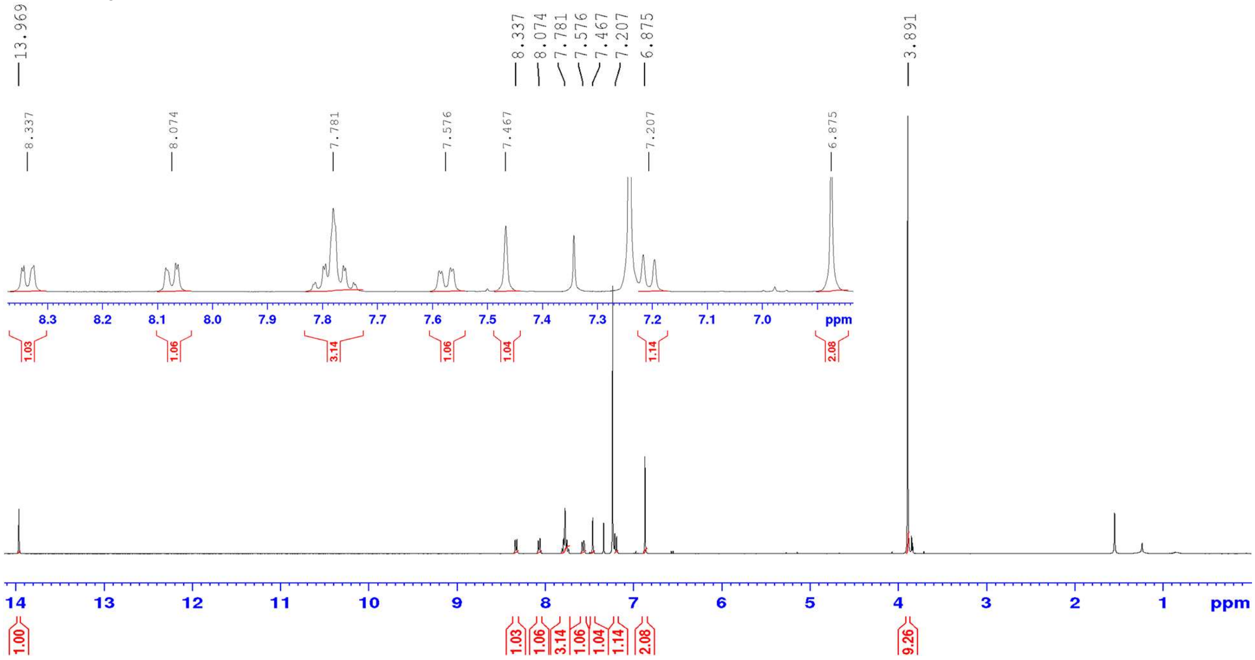

^{13}C NMR ($CDCl_3$, 100MHz)

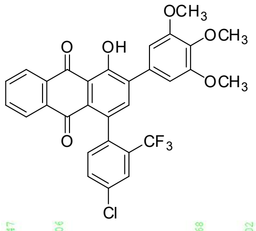

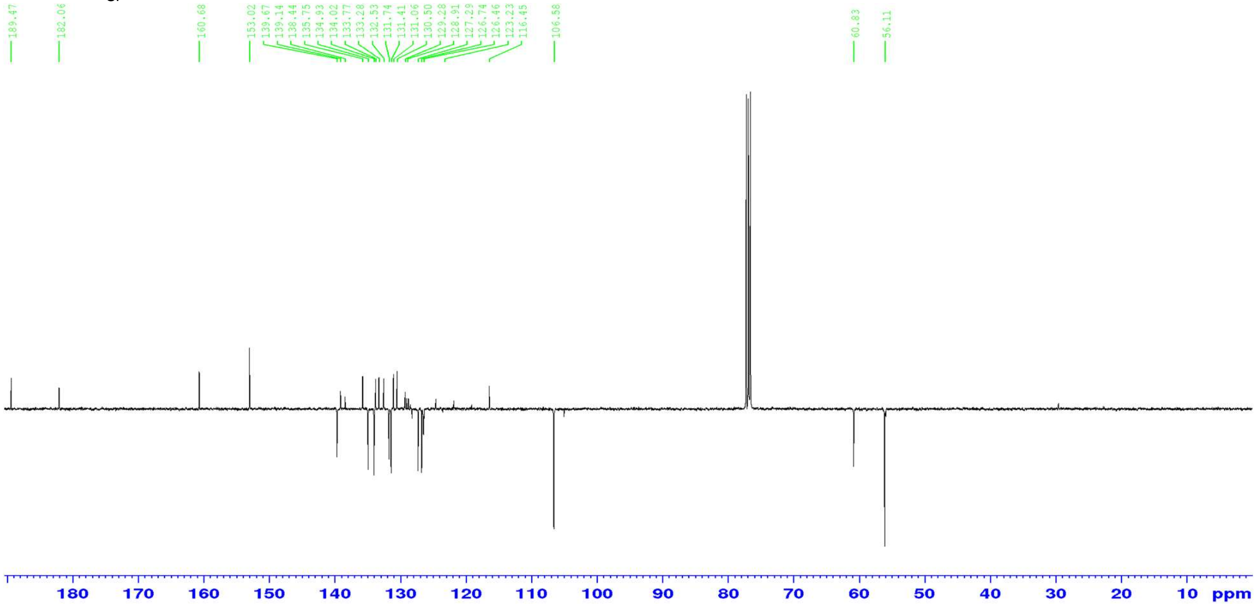

HR-MS spectra

1-Hydroxy-4-(3,4,5-trimethoxyphenyl)anthracene-9,10-dione (5)

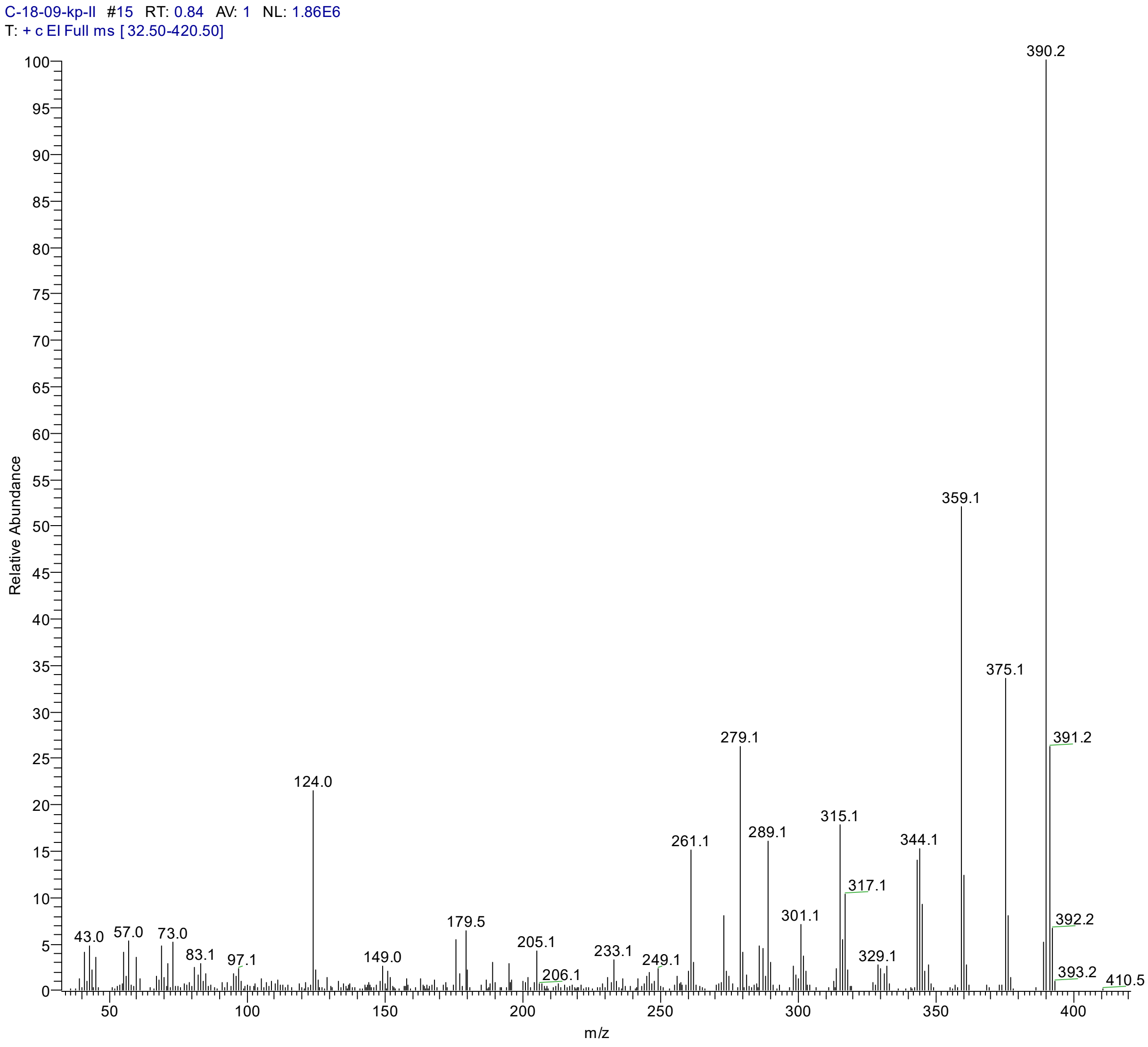

1-Hydroxy-4-phenylanthracene-9,10-dione (13)

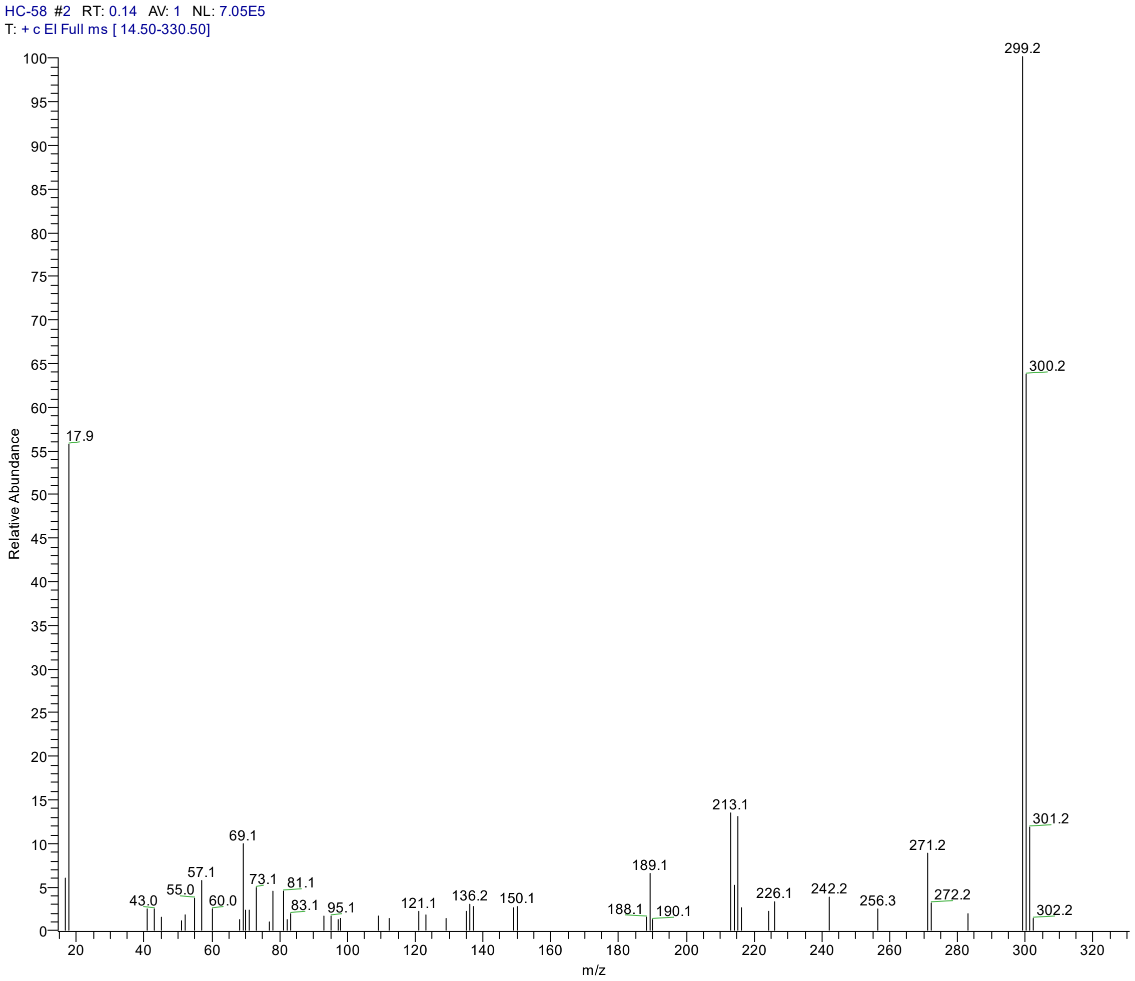

1-Hydroxy-4-(o-tolyl)anthracene-9,10-dione (14)

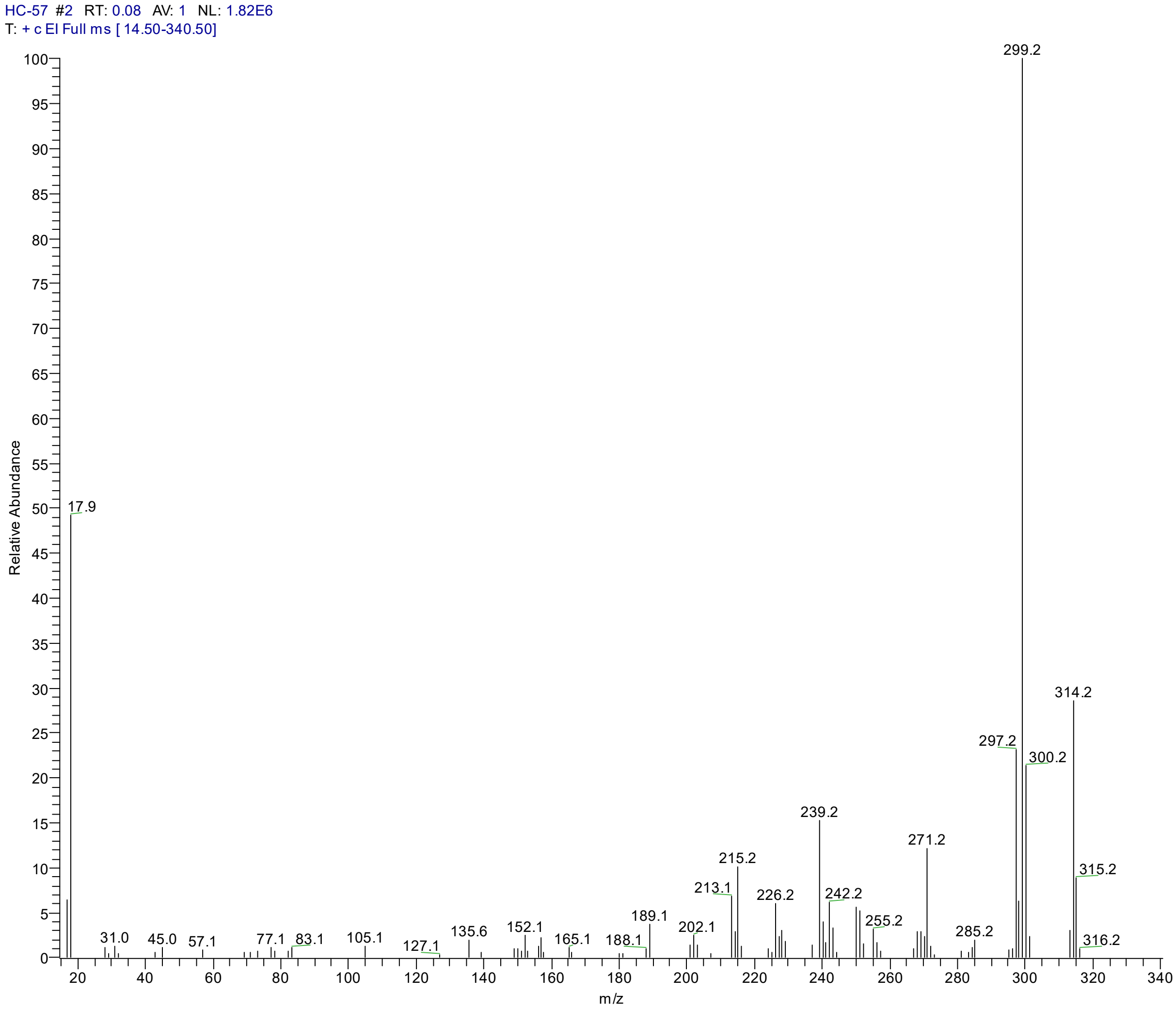

1-Hydroxy-4-(4-methoxyphenyl)anthracene-9,10-dione (15)

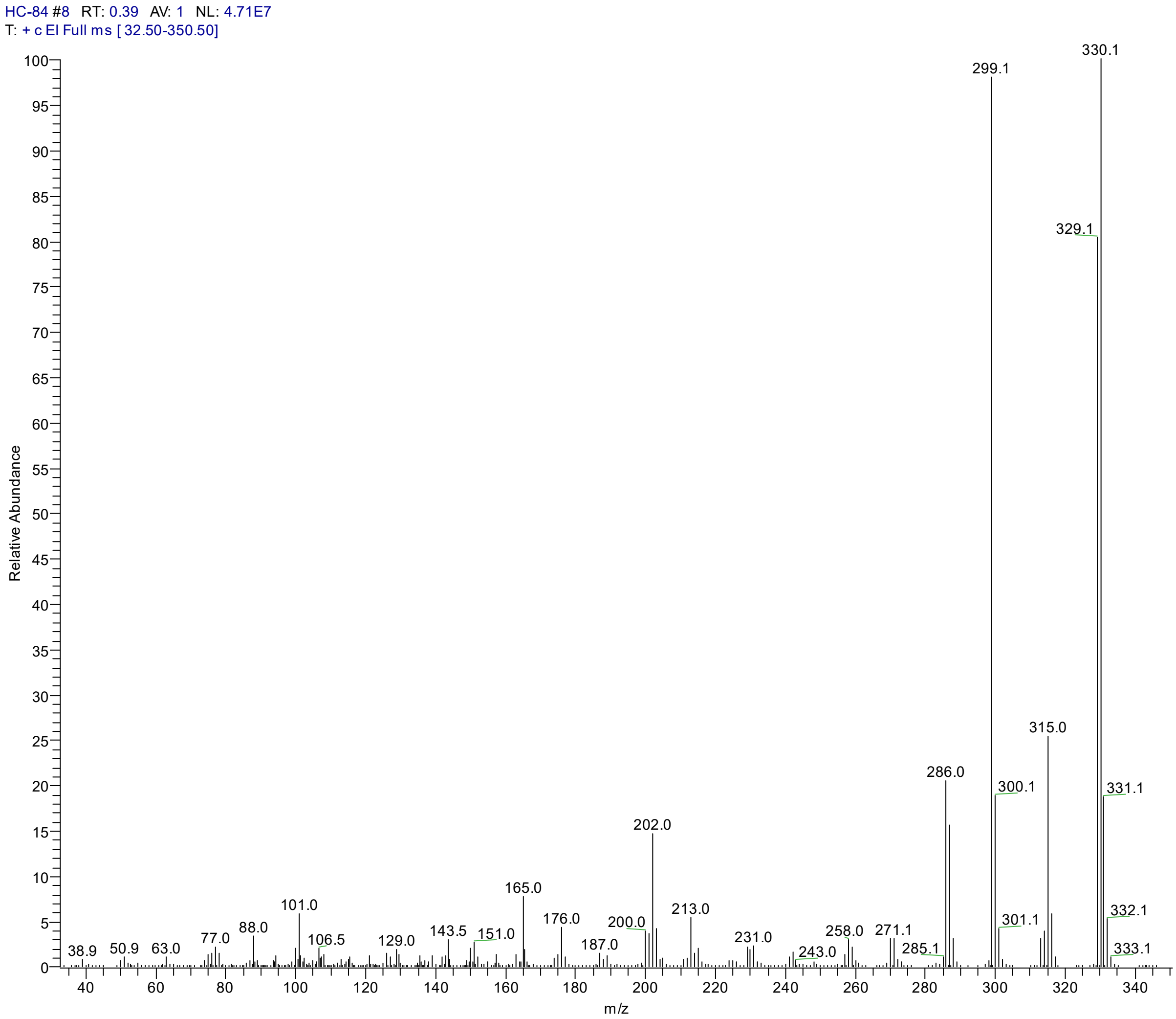

1-(2,3-Dimethoxyphenyl)-4-hydroxyanthracene-9,10-dione (16)

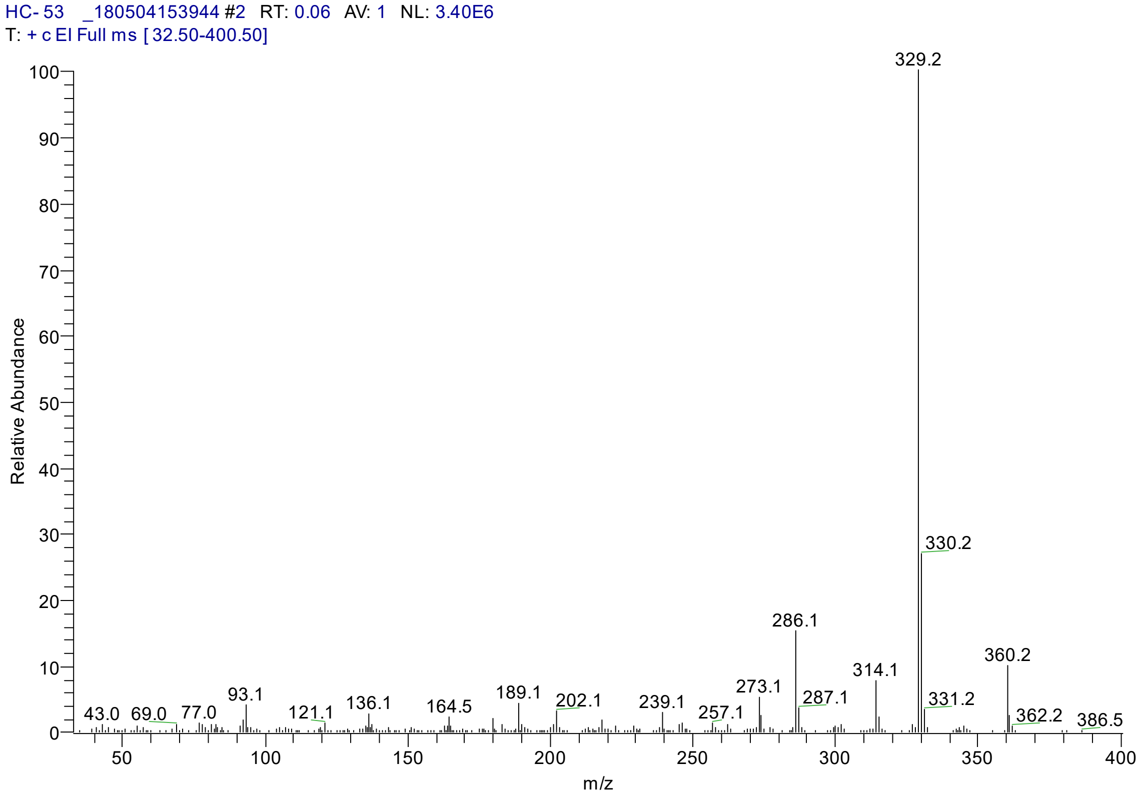

1-(3,5-Difluorophenyl)-4-hydroxyanthracene-9,10-dione (17)

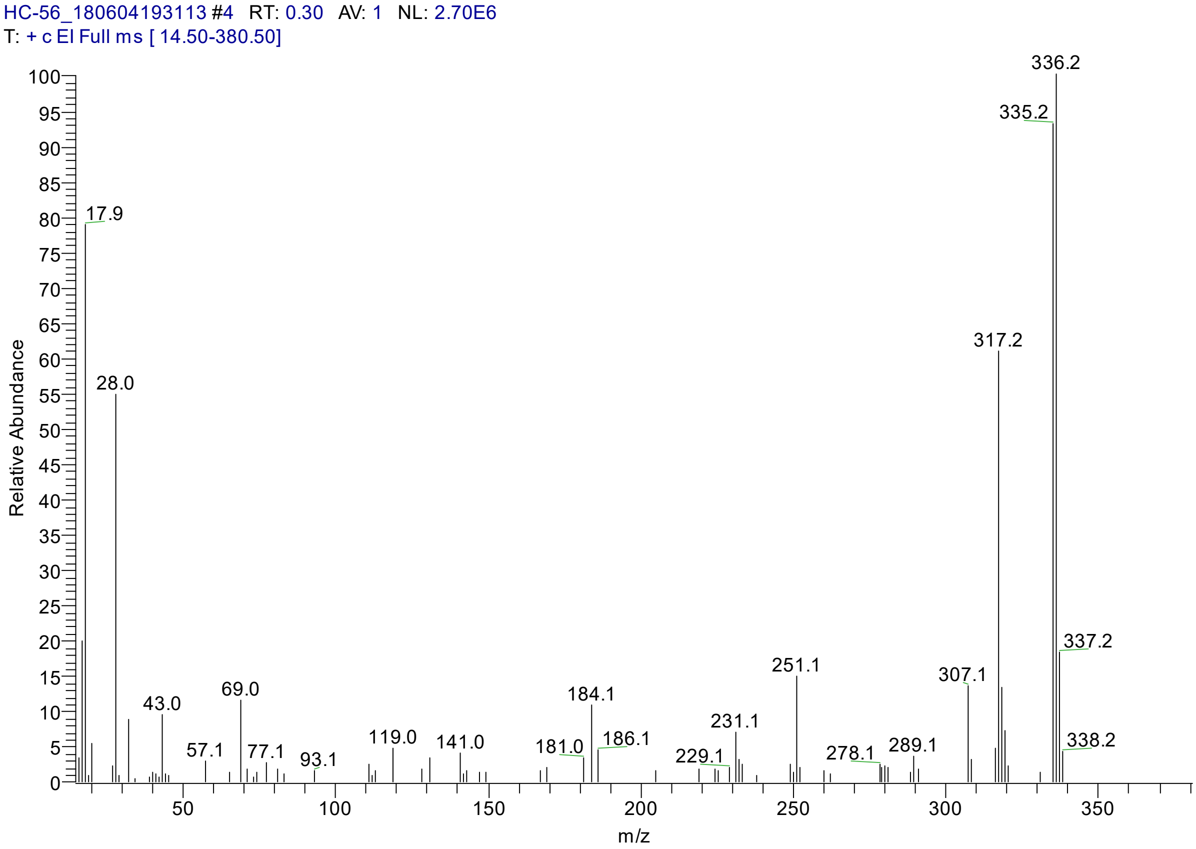

1-(2-Chloro-5-(trifluoromethyl)phenyl)-4-hydroxyanthracene-9,10-dione (18)

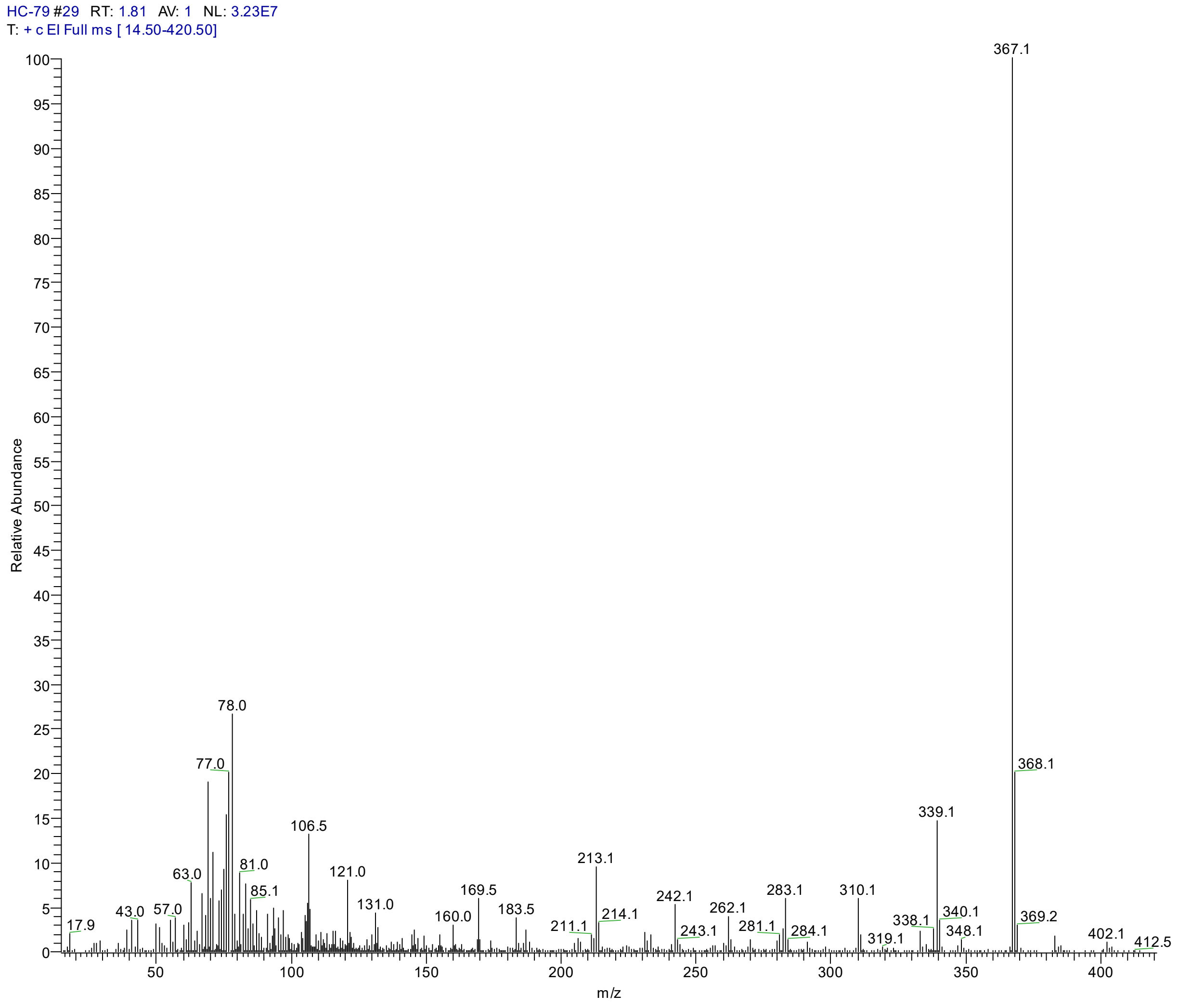

1-(4-Chloro-2-(trifluoromethyl)phenyl)-4-hydroxyanthracene-9,10-dione (19)

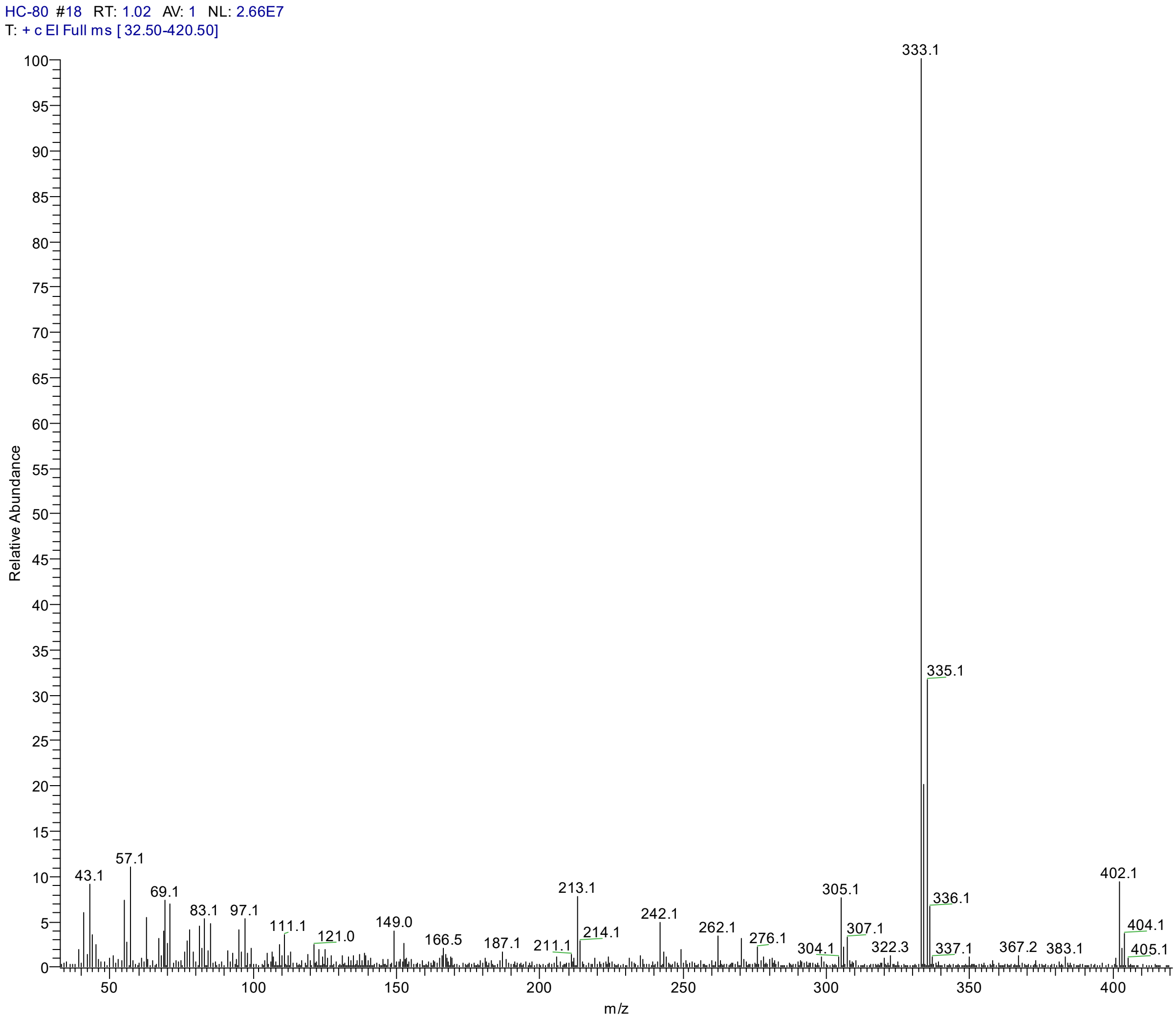

1-(Furan-2-yl)-4-hydroxyanthracene-9,10-dione (22)

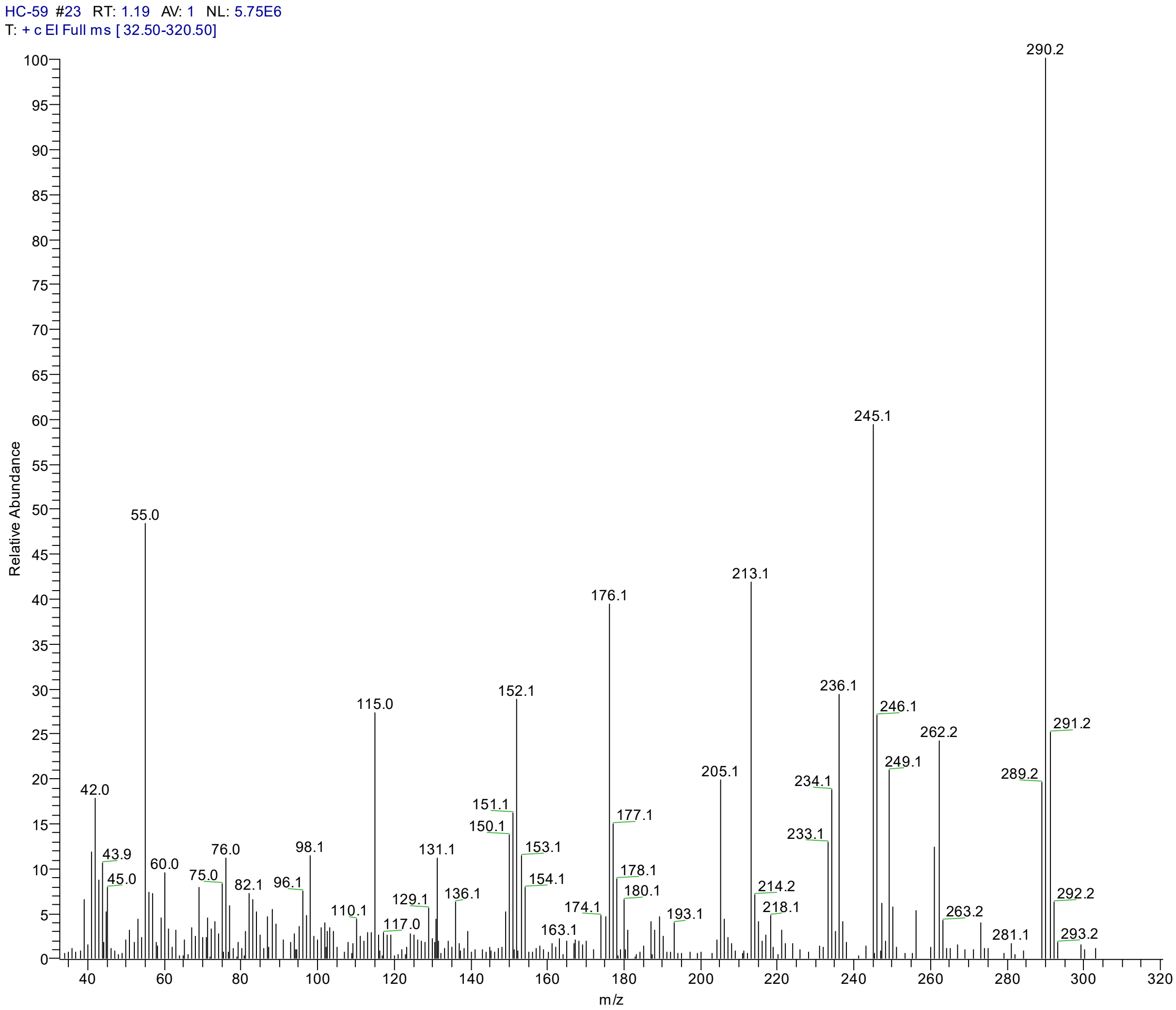

1-(Furan-3-yl)-4-hydroxyanthracene-9,10-dione (23)

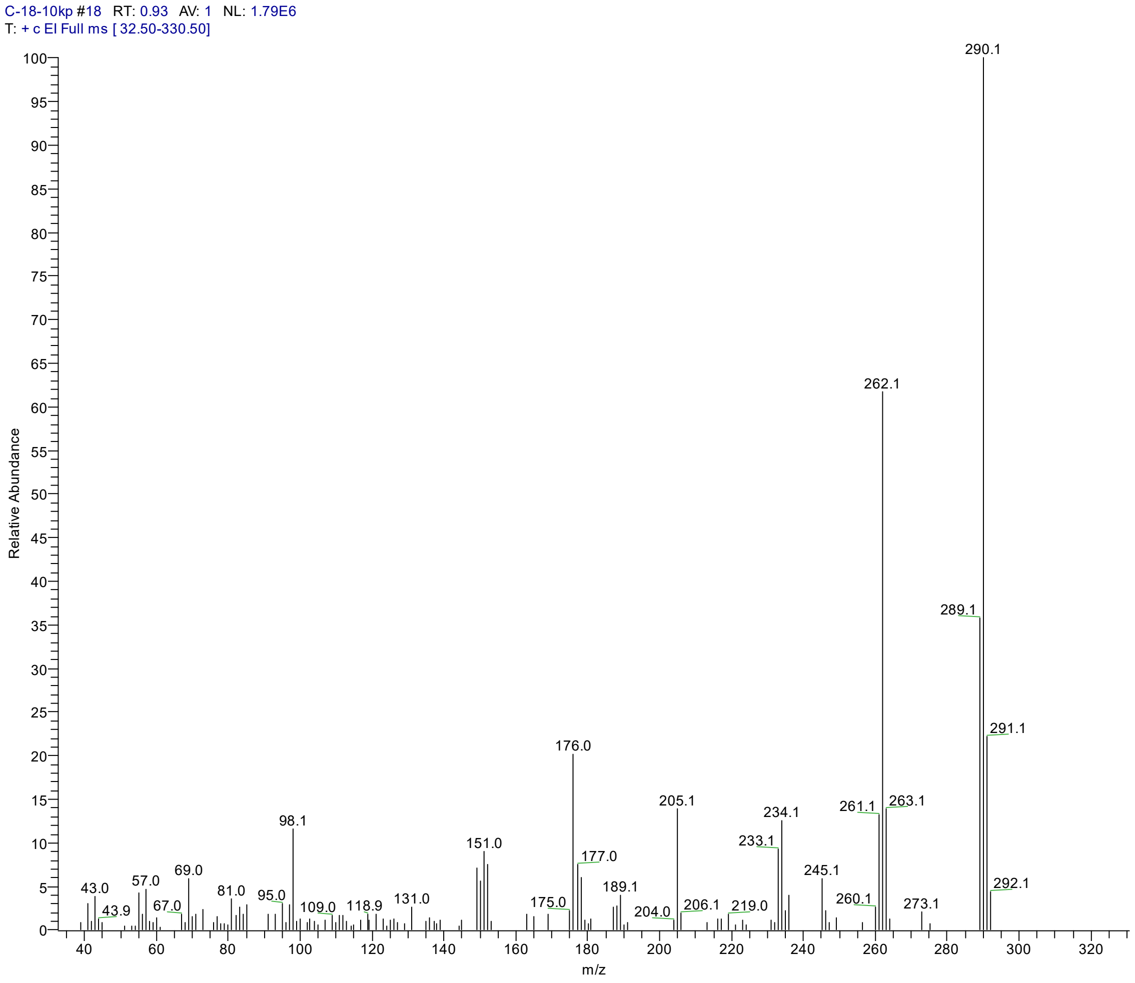

1-Hydroxy-2-(3,4,5-trimethoxyphenyl)anthracene-9,10-dione (24)

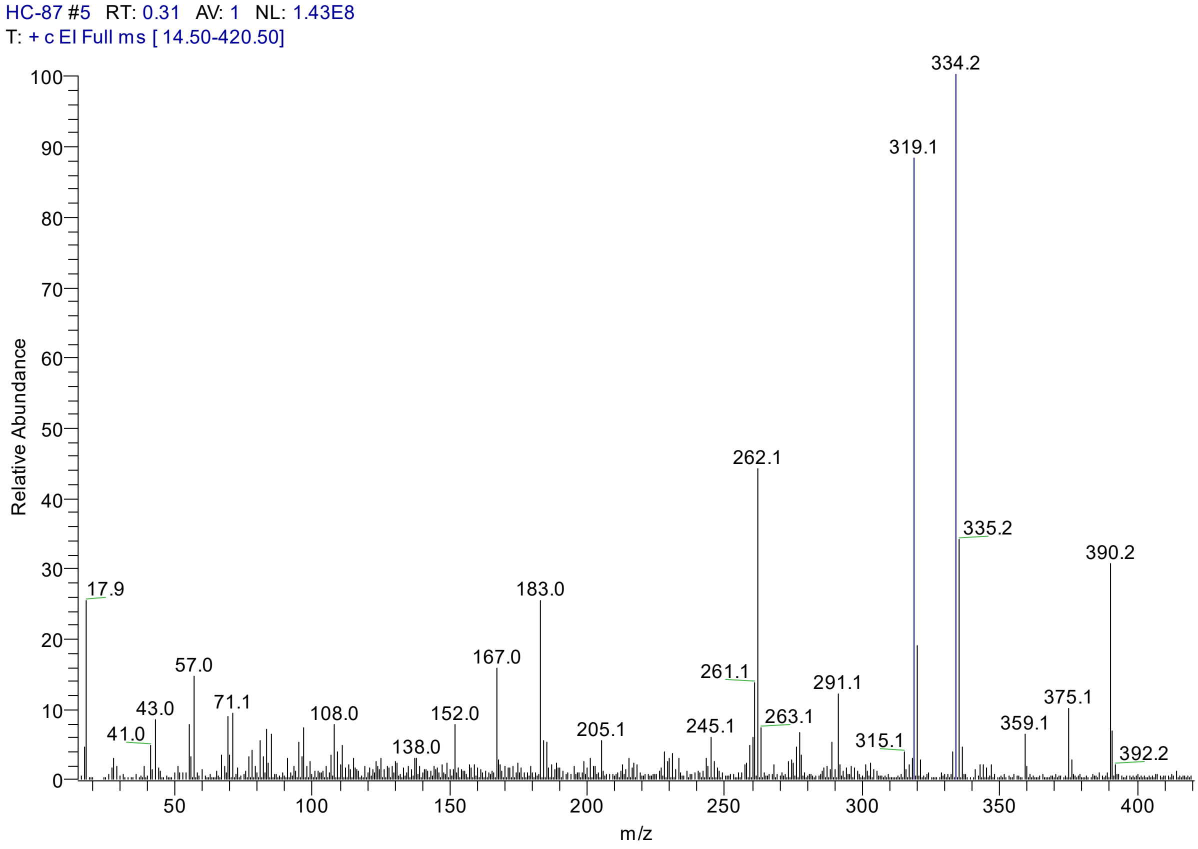

1-Hydroxy-2-phenylanthracene-9,10-dione (25)

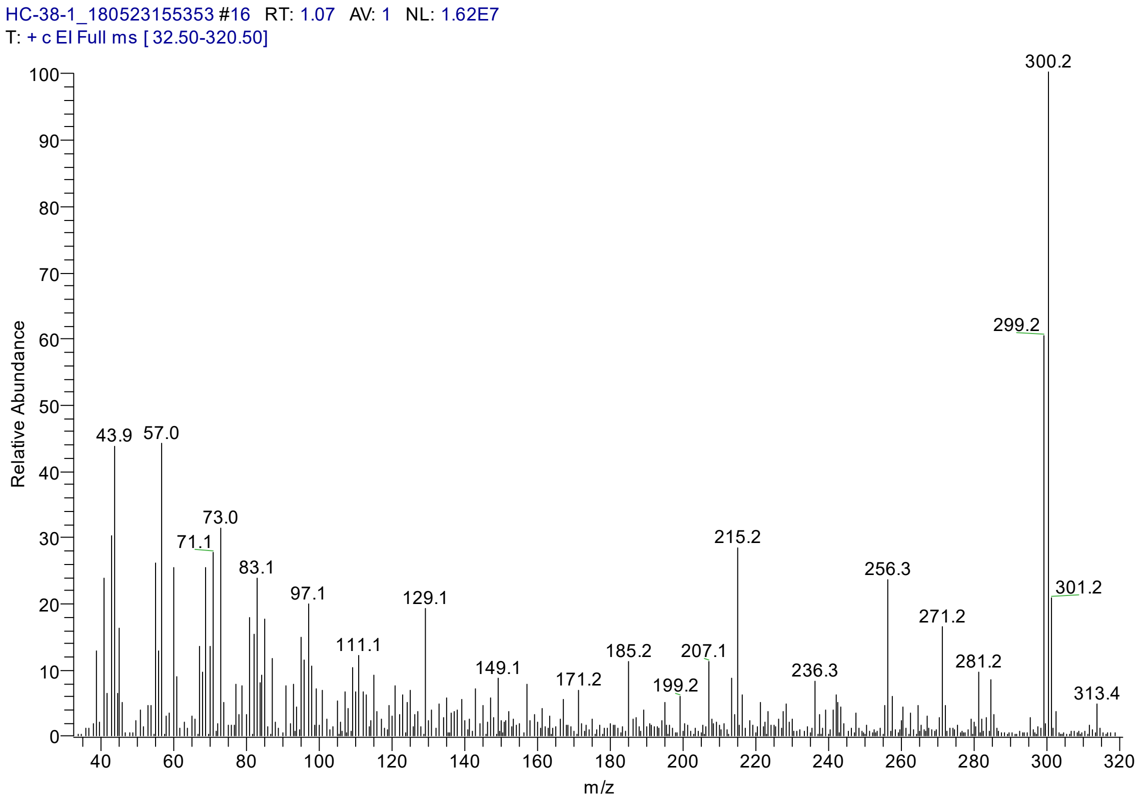

1-Hydroxy-2-(o-tolyl)anthracene-9,10-dione (26)

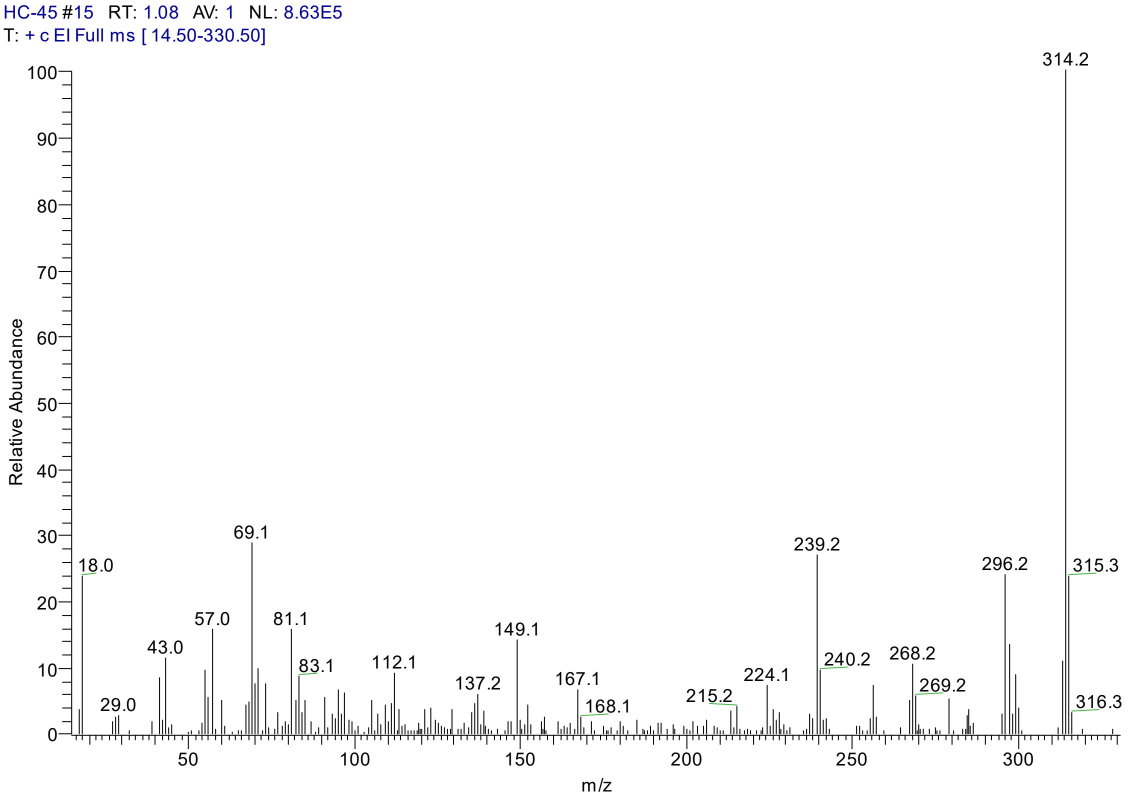

1-Hydroxy-2-(4-methoxyphenyl)anthracene-9,10-dione (27)

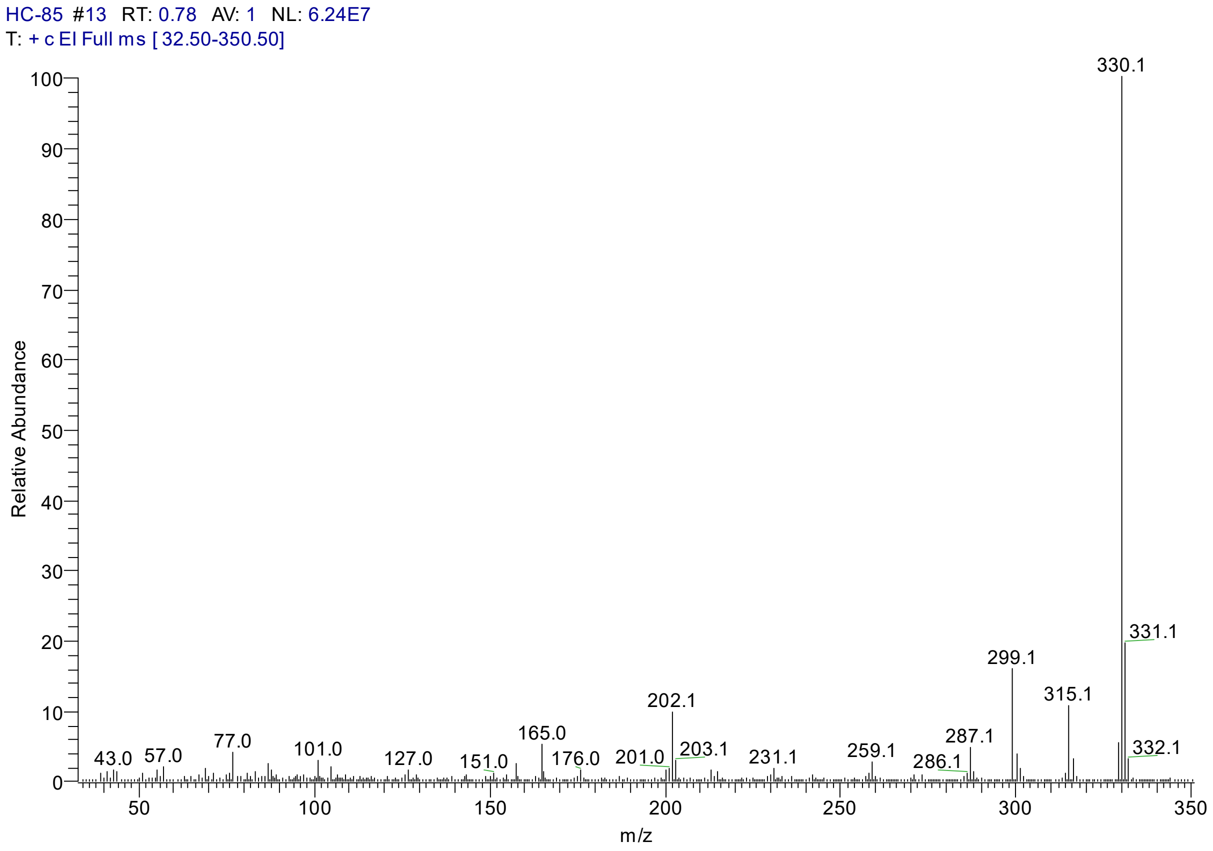

2-(2,3-Dimethoxyphenyl)-1-hydroxyanthracene-9,10-dione (28)

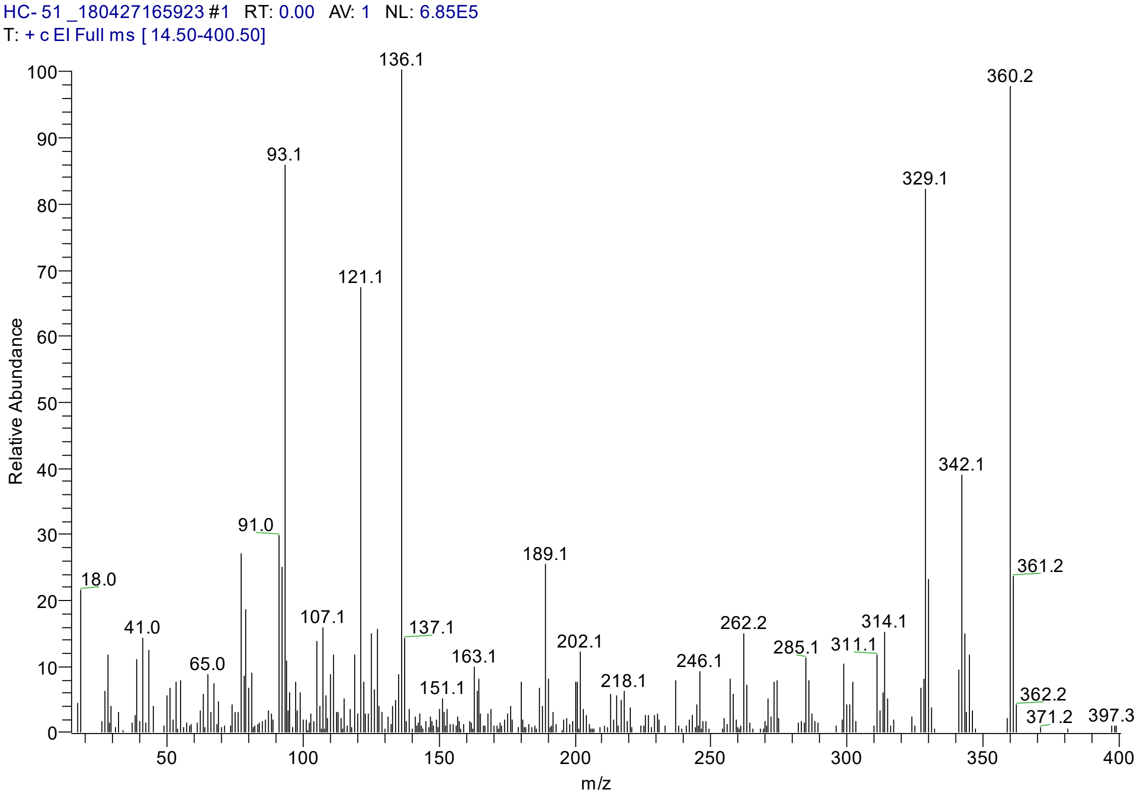

2-(3,5-Difluorophenyl)-1-hydroxyanthracene-9,10-dione (29)

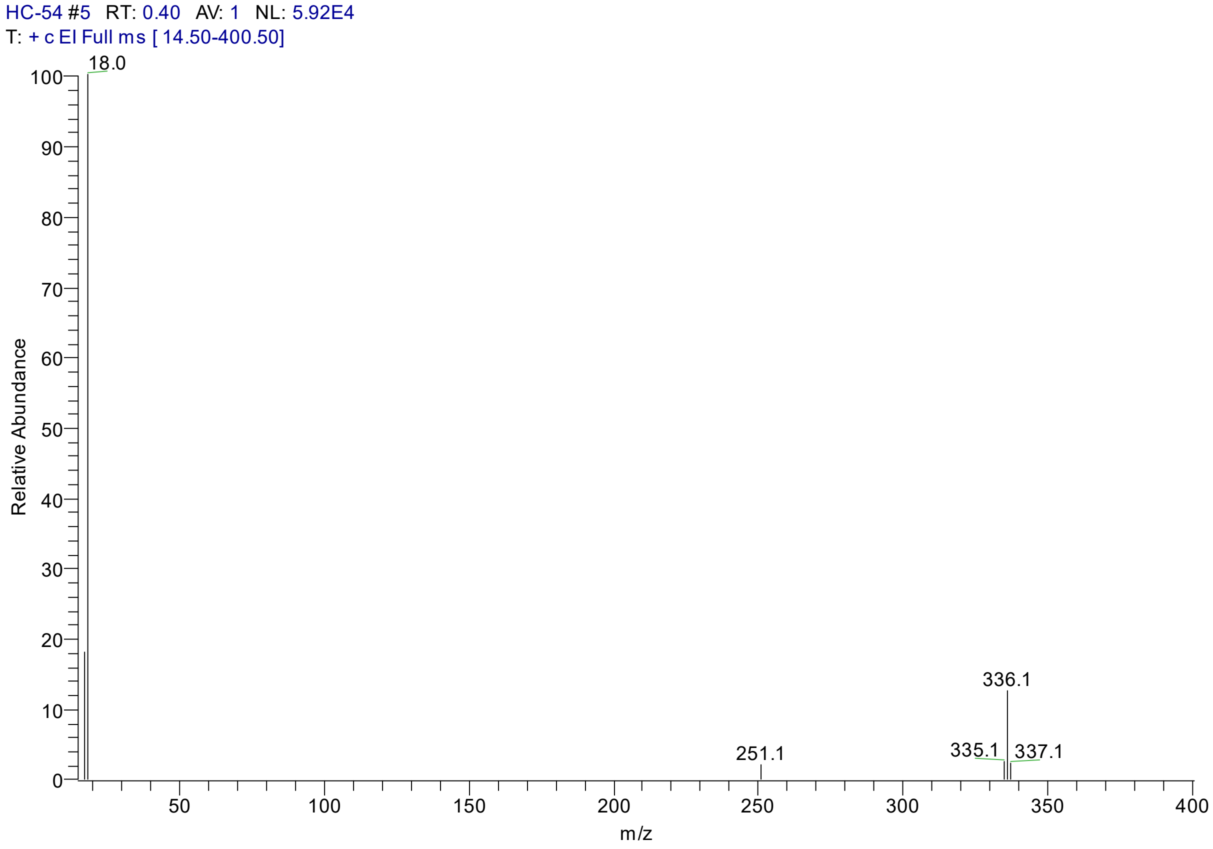

2-(2-Chloro-5-(trifluoromethyl)phenyl)-1-hydroxyanthracene-9,10-dione (30)

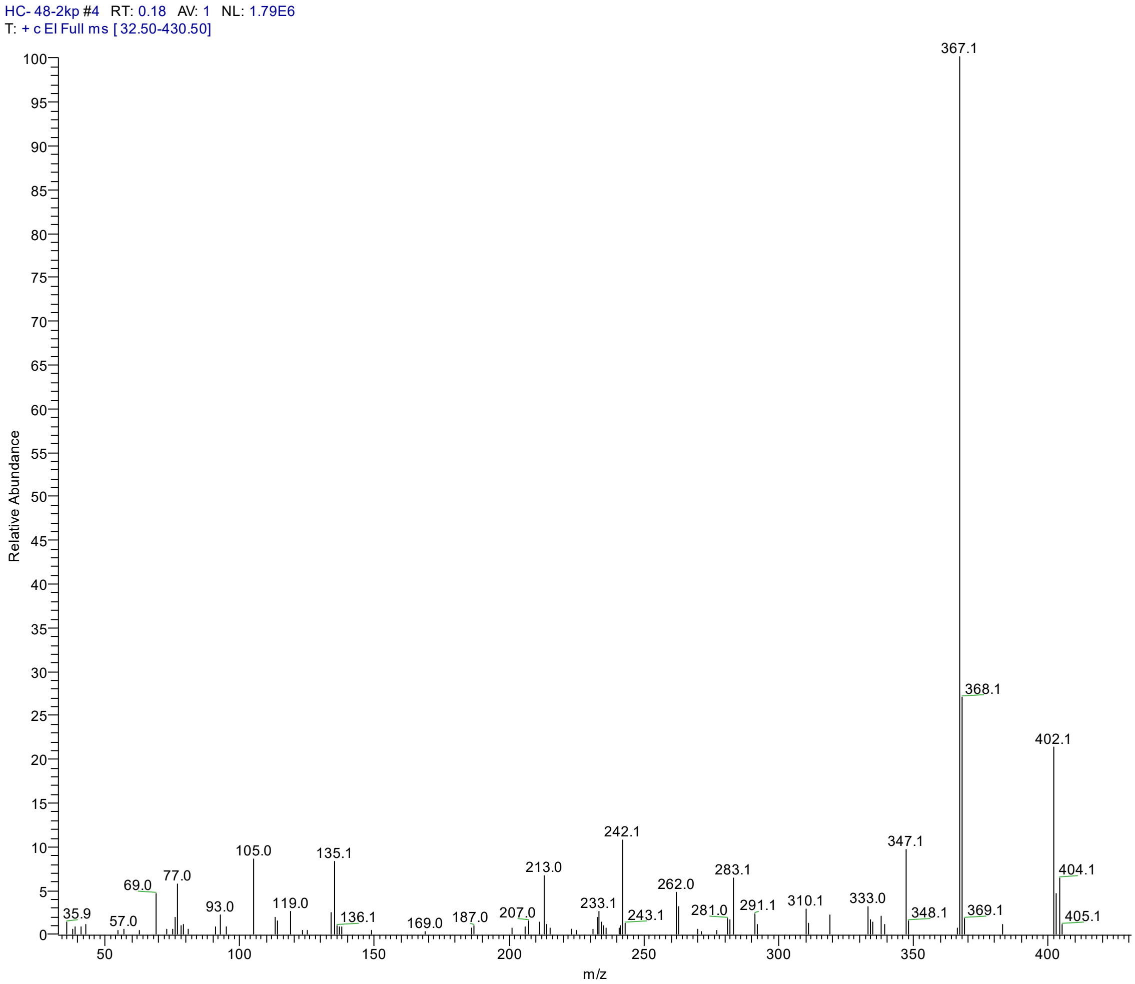

2-(4-Chloro-2-(trifluoromethyl)phenyl)-1-hydroxyanthracene-9,10-dione (31)

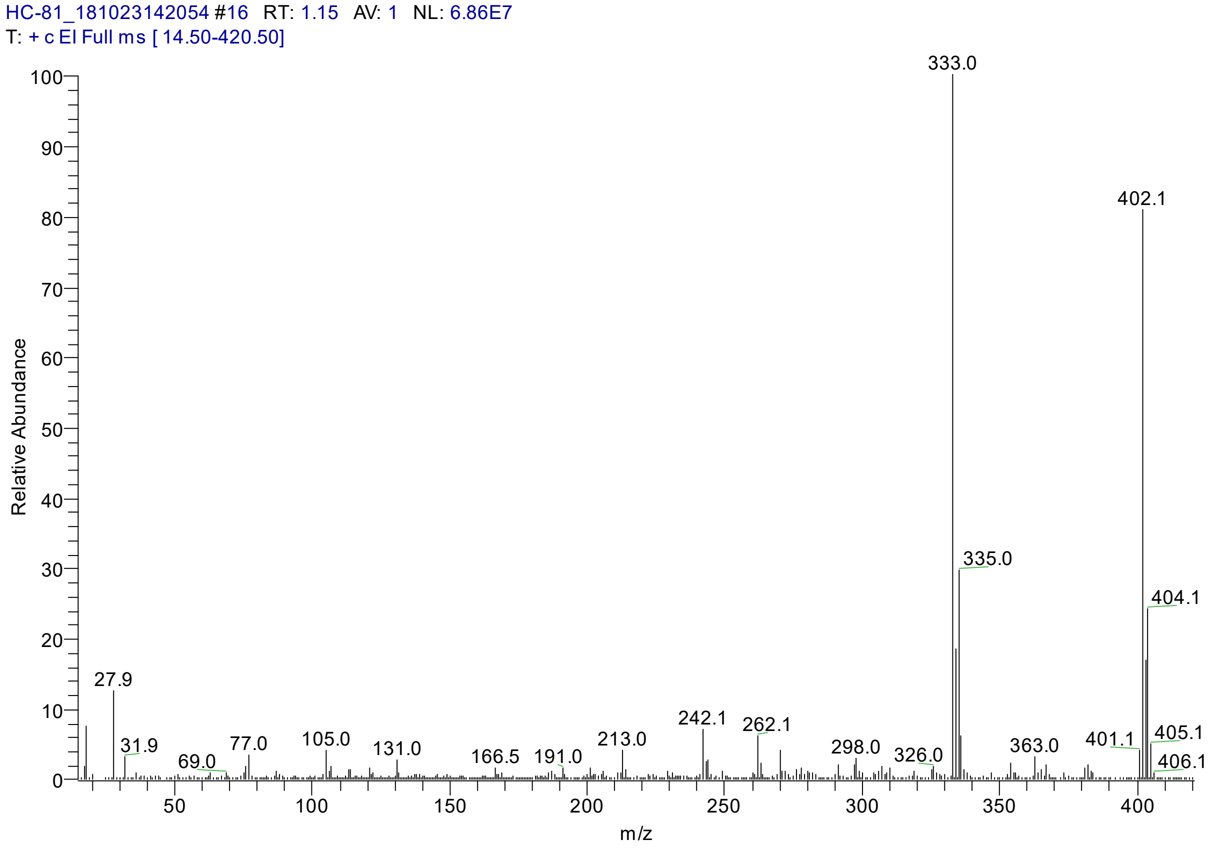

1-Hydroxy-2,4-di-(3,4,5-trimethoxyphenyl)anthracene-9,10-dione (32)

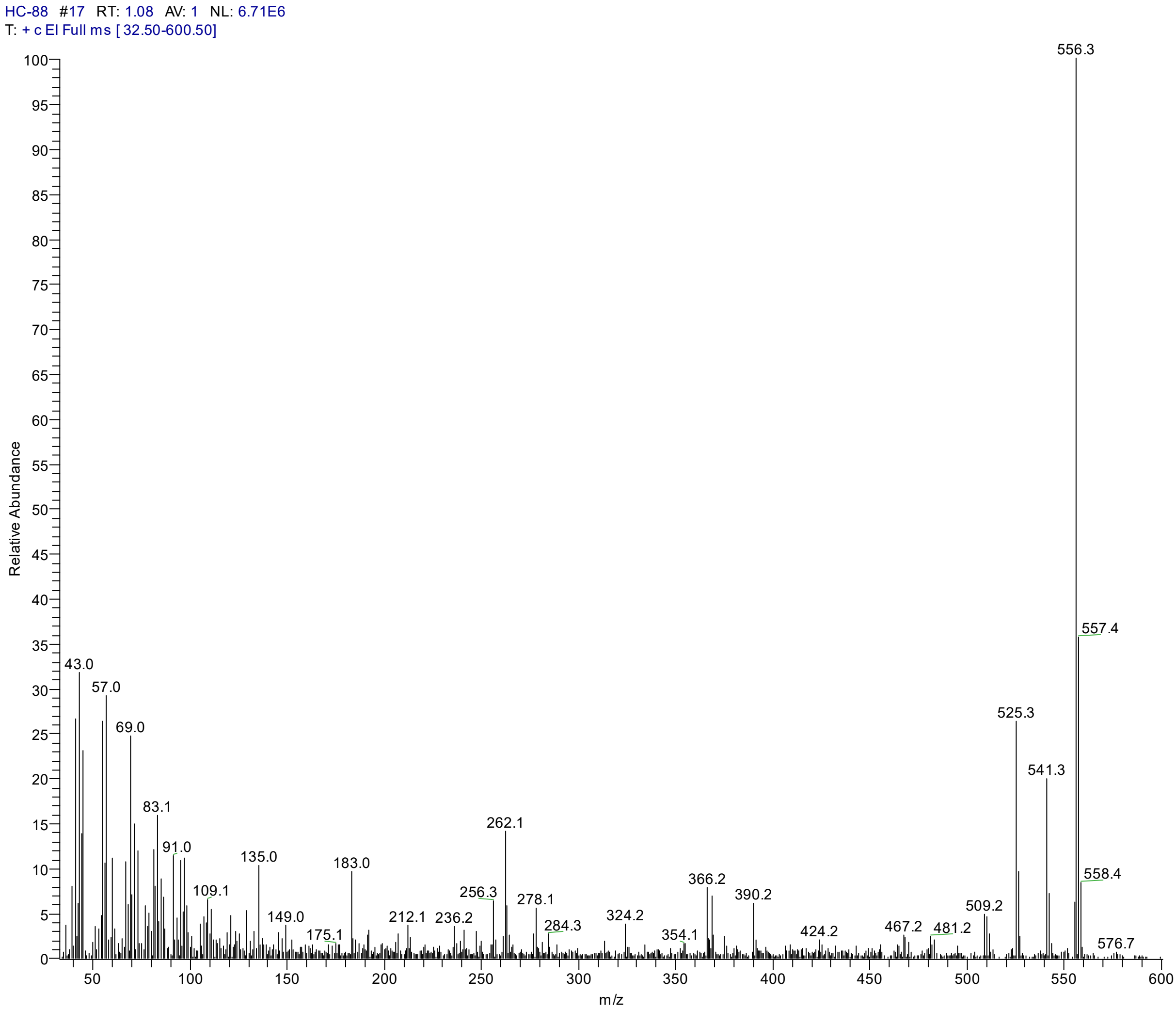

1-Hydroxy-2,4-diphenylanthracene-9,10-dione (33)

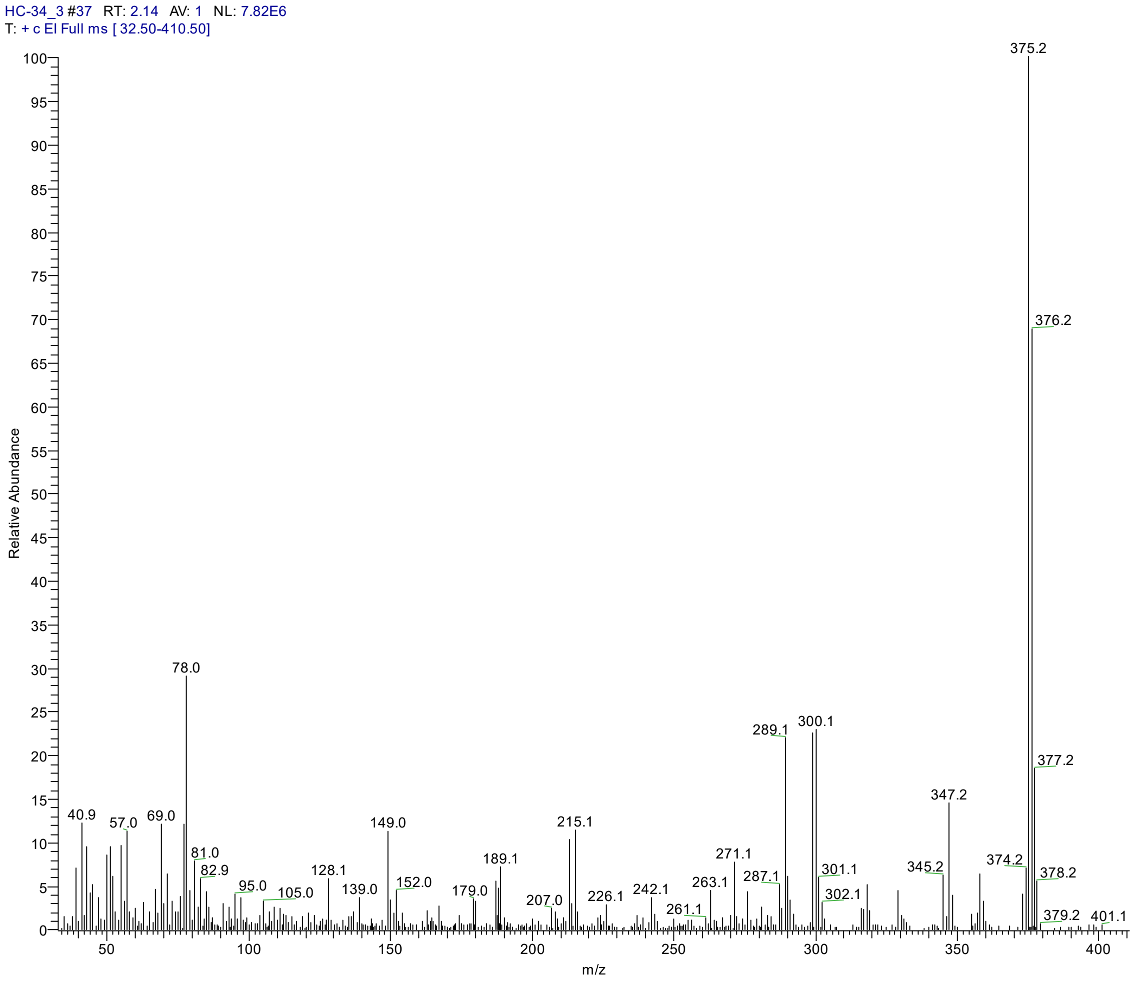

1-Hydroxy-2,4-di-(*o*-tolyl)anthracene-9,10-dione (34)

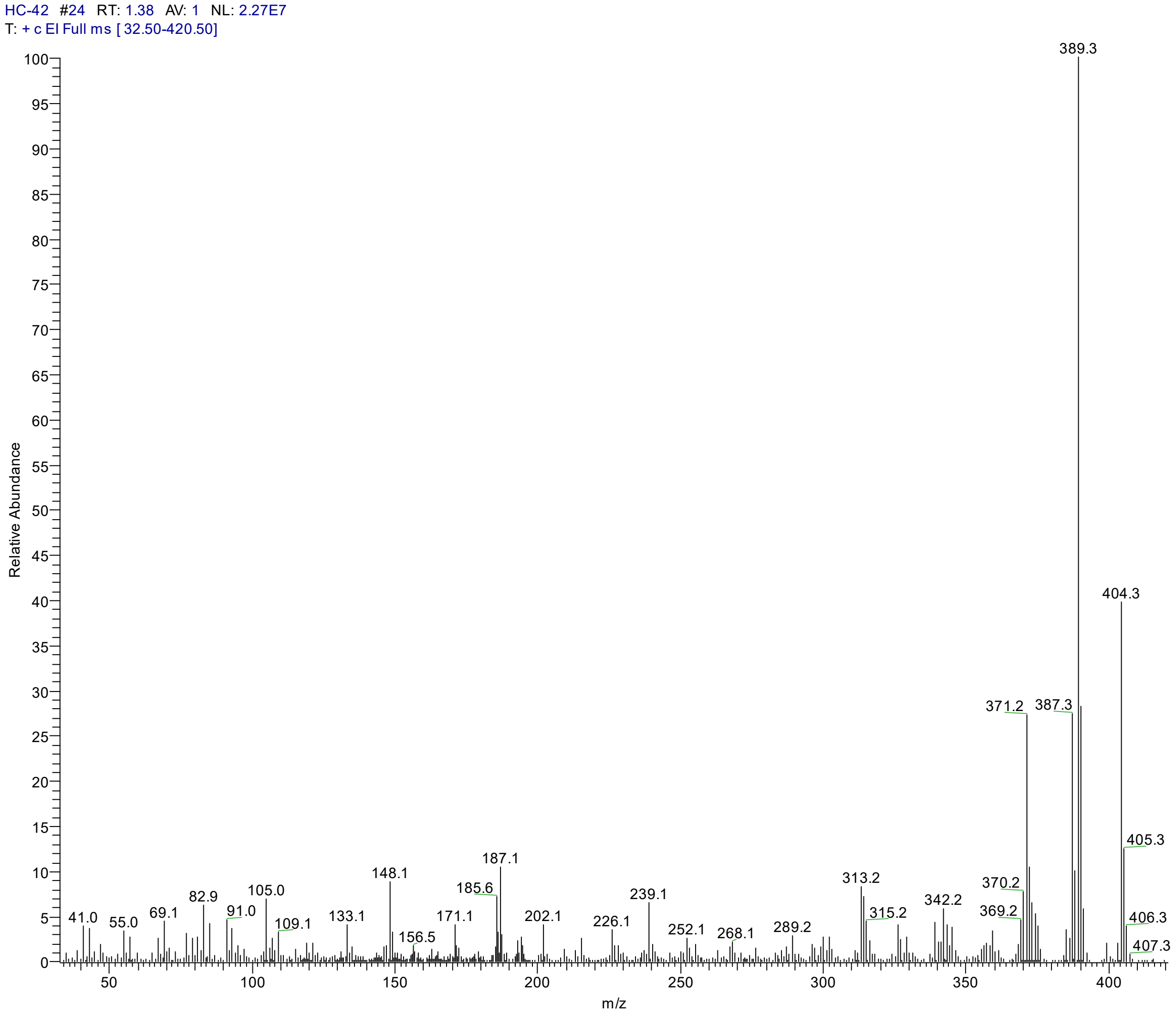

1-Hydroxy-2,4-di-(4-methoxyphenyl)anthracene-9,10-dione (35)

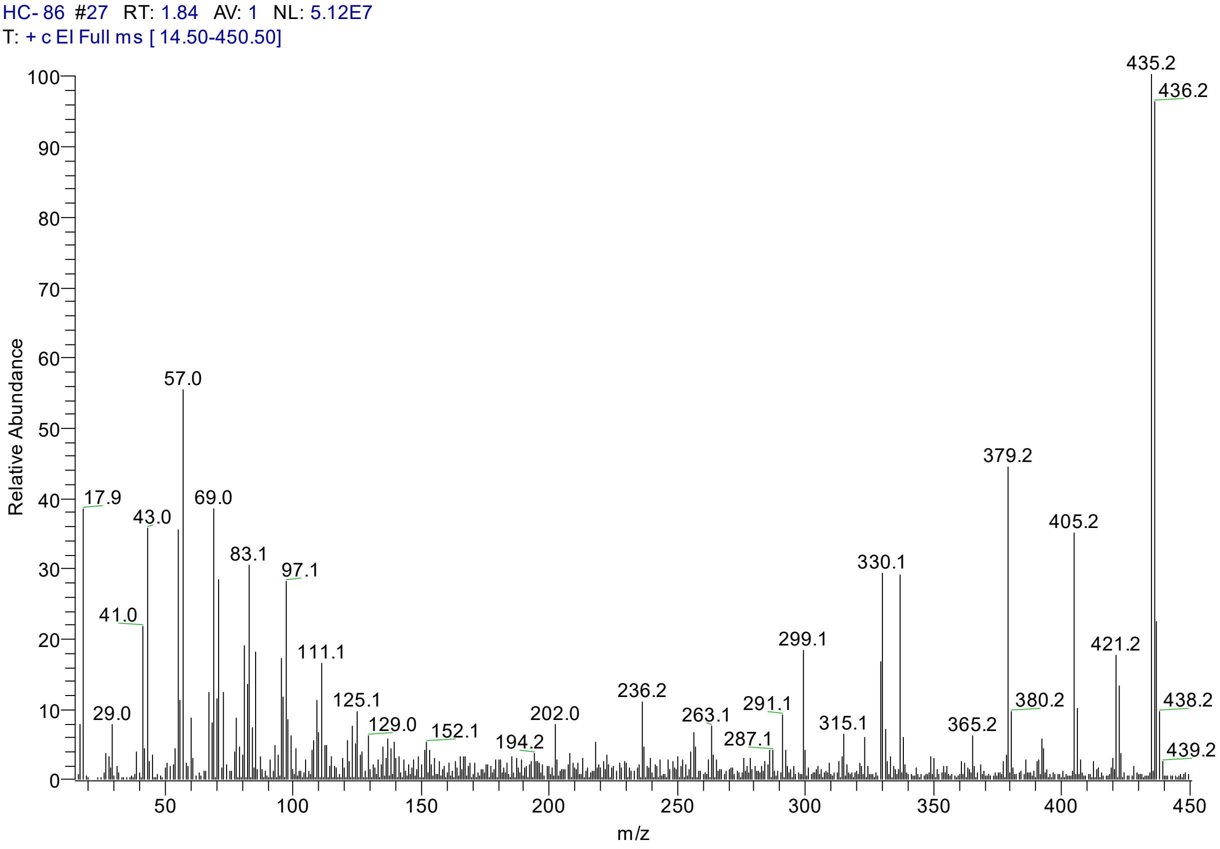

2,4-Di-(2,3-dimethoxyphenyl)-1-hydroxyanthracene-9,10-dione (36)

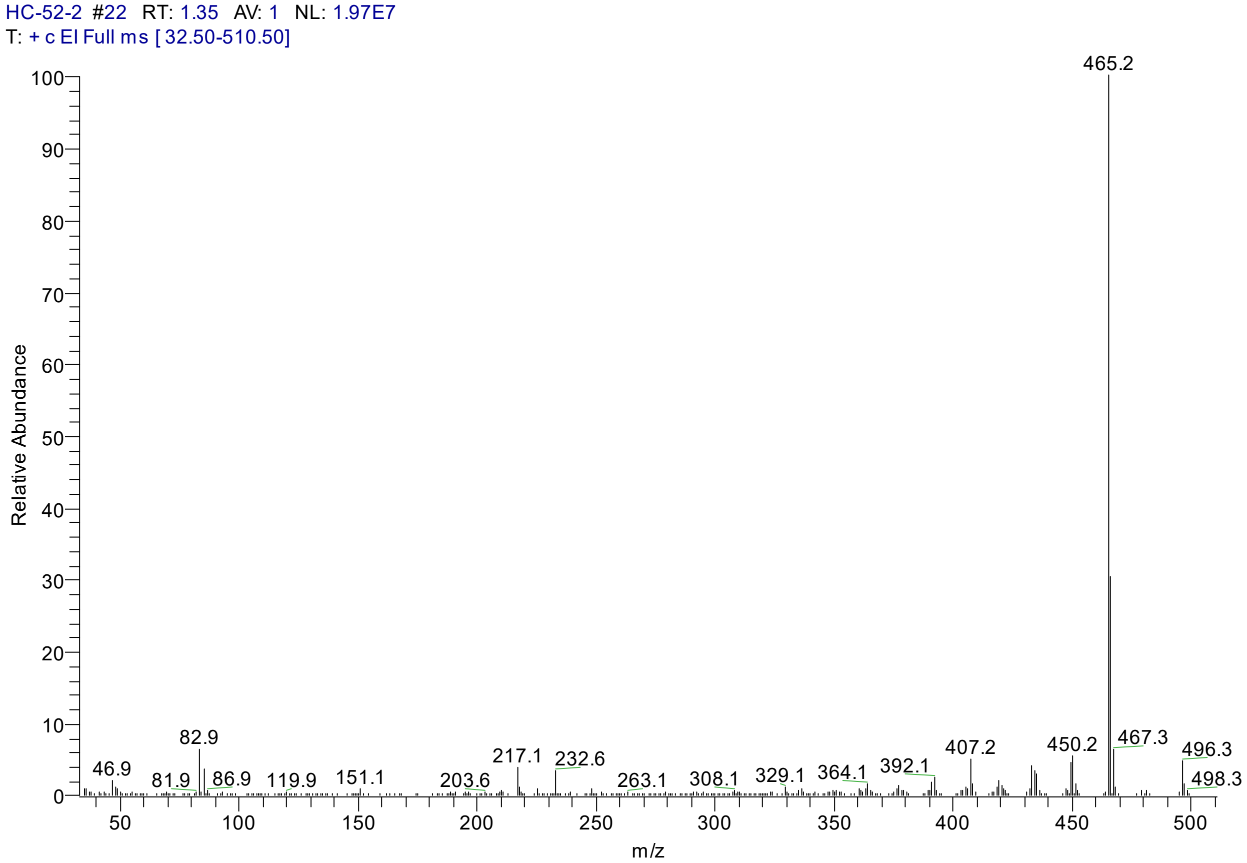

2,4-Di-(3,5-difluorophenyl)-1-hydroxyanthracene-9,10-dione (37)

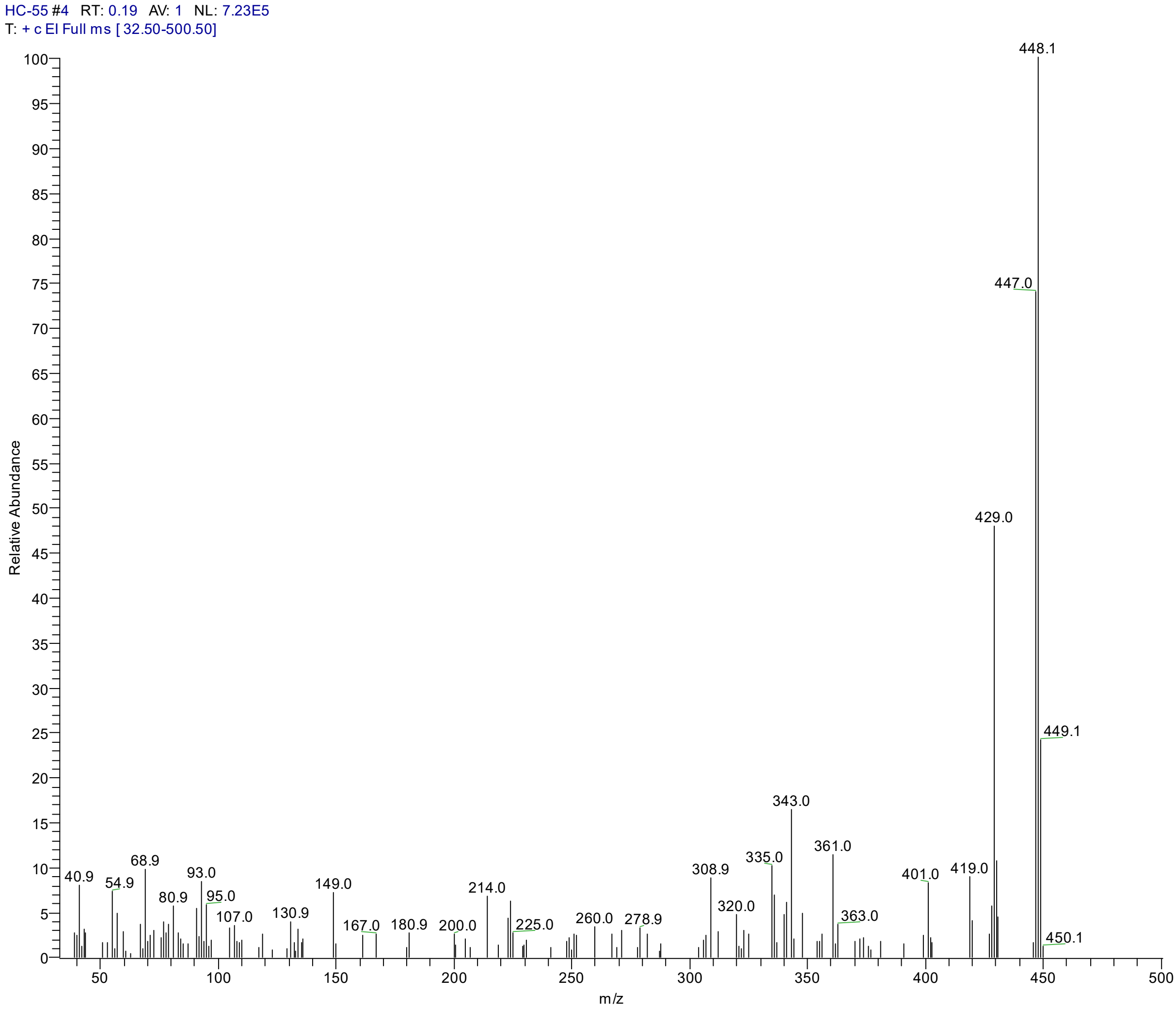

2,4-Di-(2-chloro-5-(trifluoromethyl)phenyl)-1-hydroxyanthracene-9,10-dione (38)

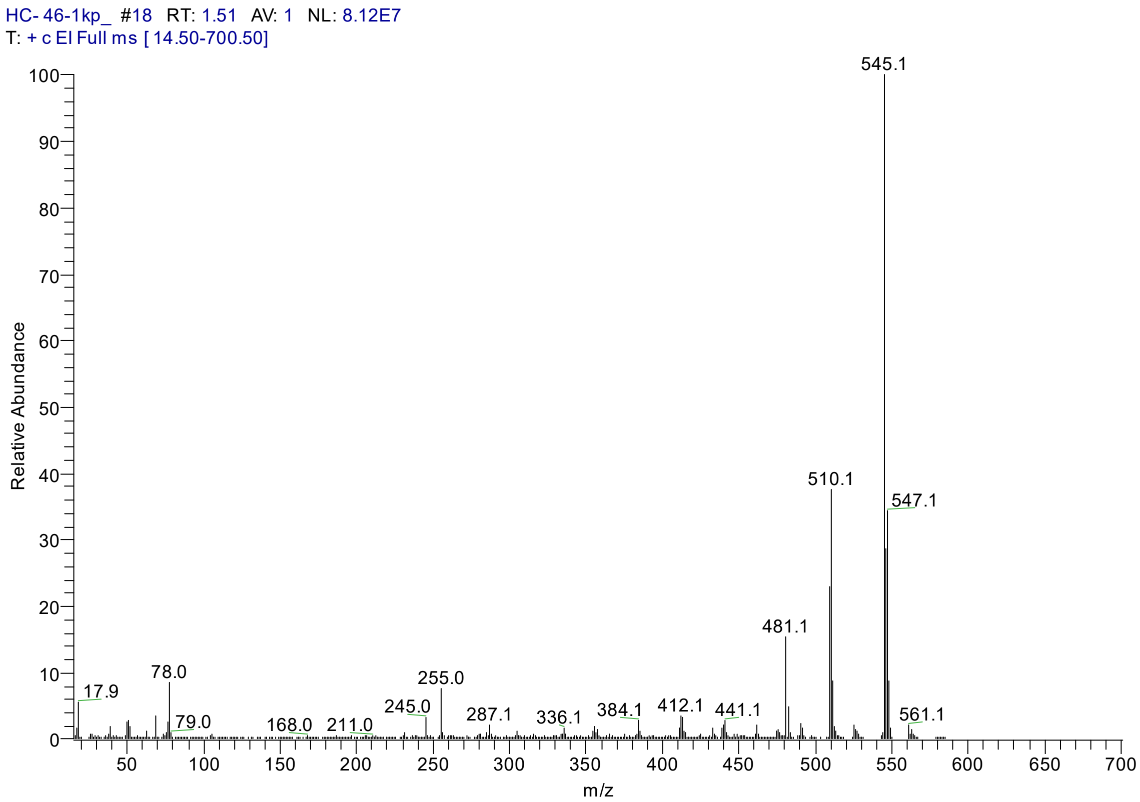

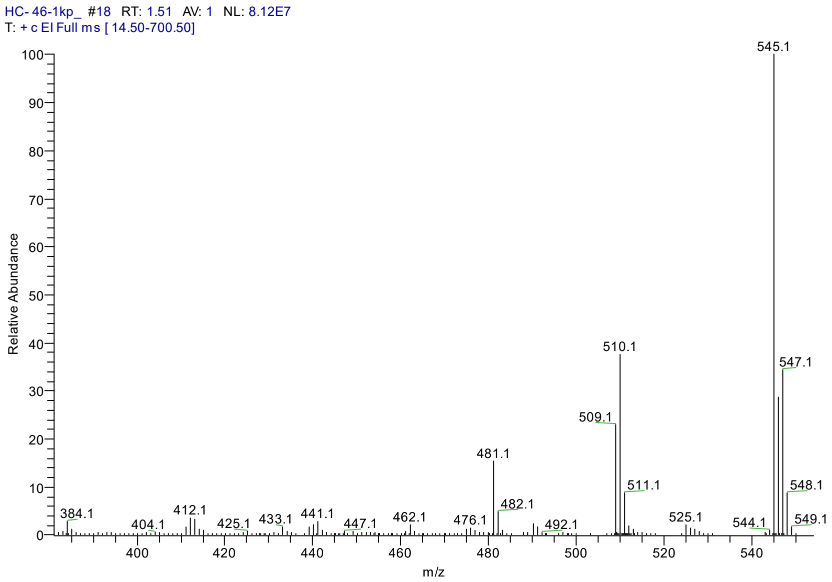

2,4-Di-(4-chloro-2-(trifluoromethyl)phenyl)-1-hydroxyanthracene-9,10-dione (39)

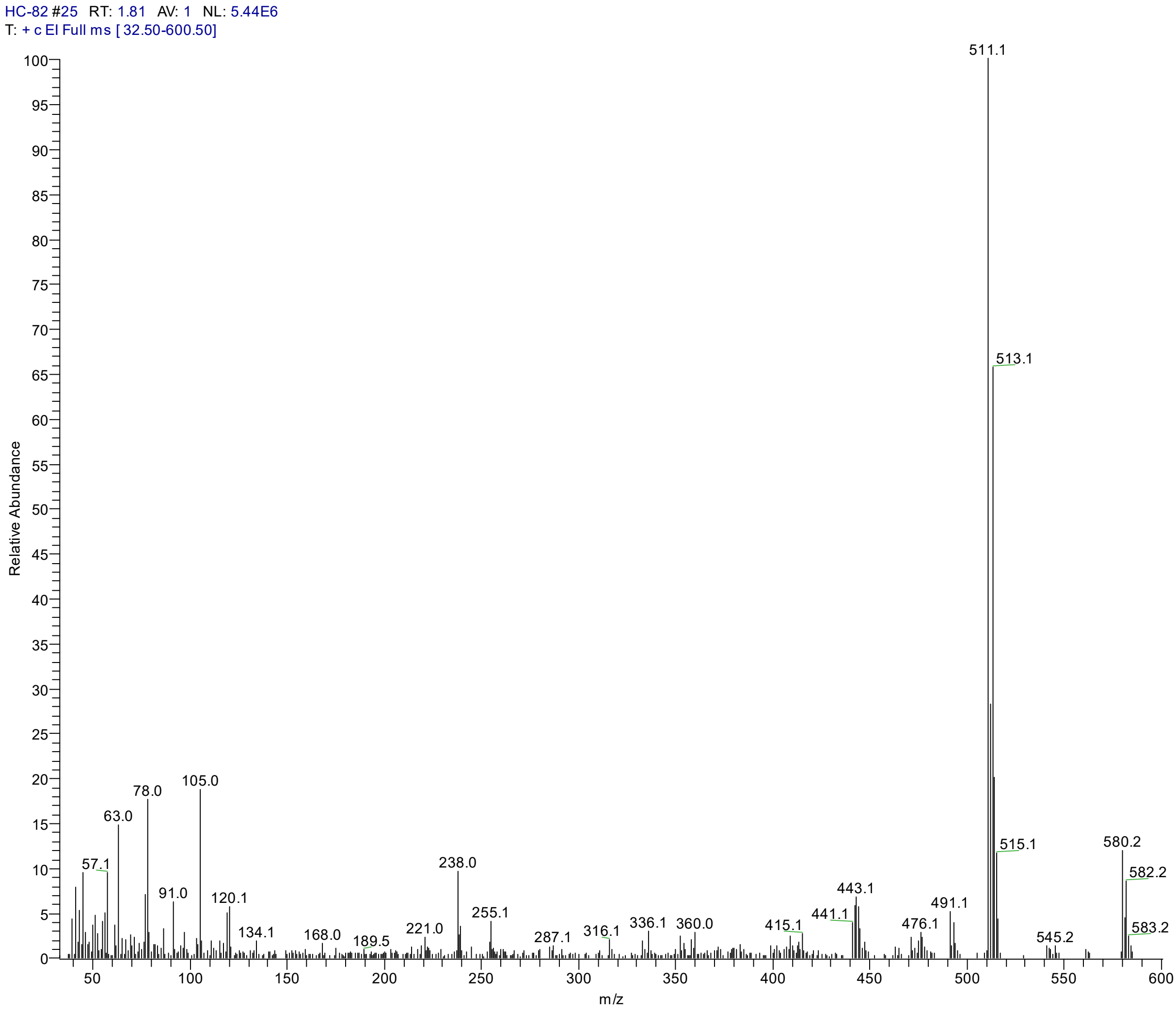

4-bromo-1-hydroxy-2-(4-methoxyphenyl)anthracene-9,10-dione (40)

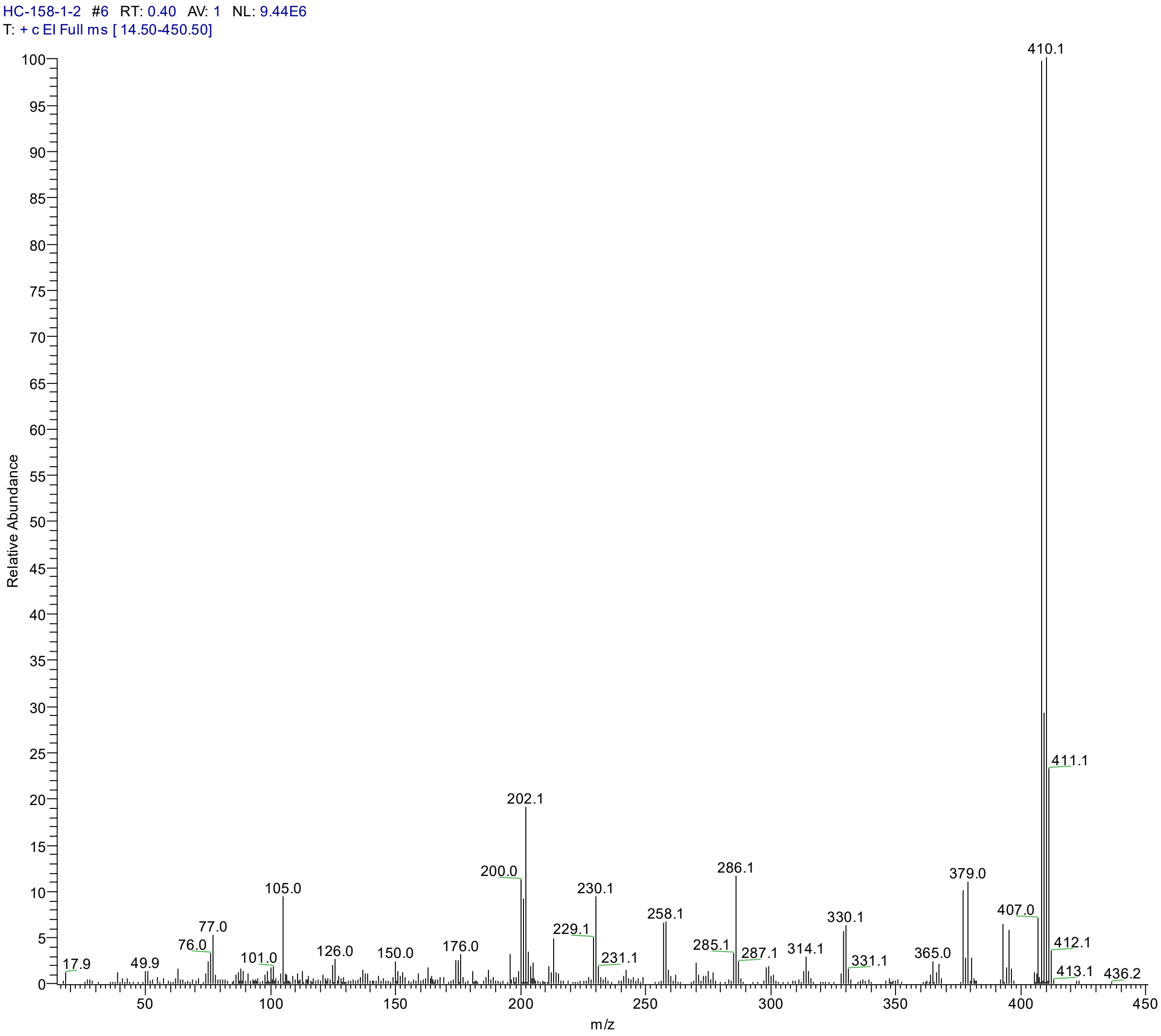

2-bromo-1-hydroxy-4-(4-methoxyphenyl)anthracene-9,10-dione (41)

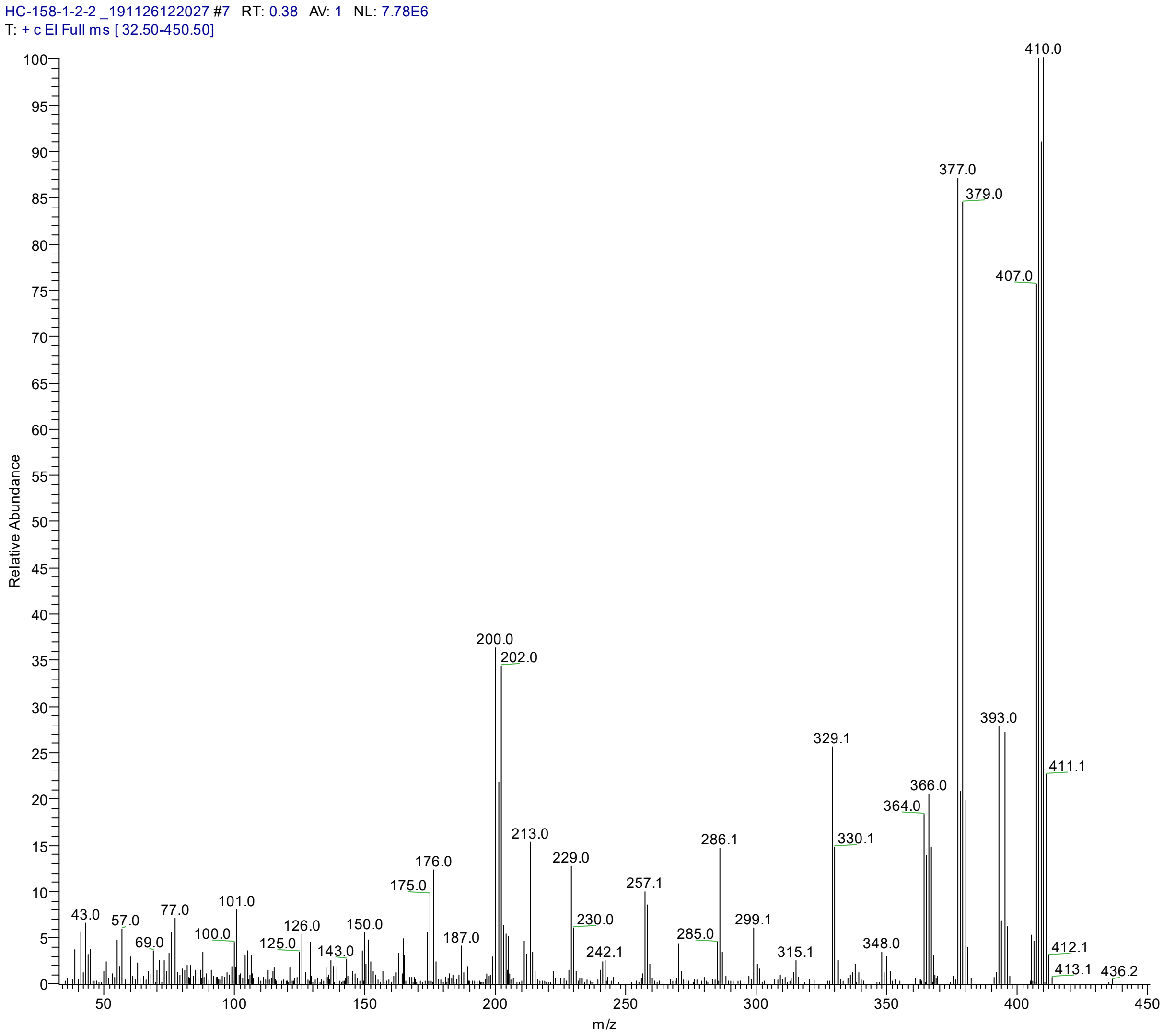

4-Bromo-1-hydroxy-2-(3,4,5-trimethoxyphenyl)anthracene-9,10-dione (42)

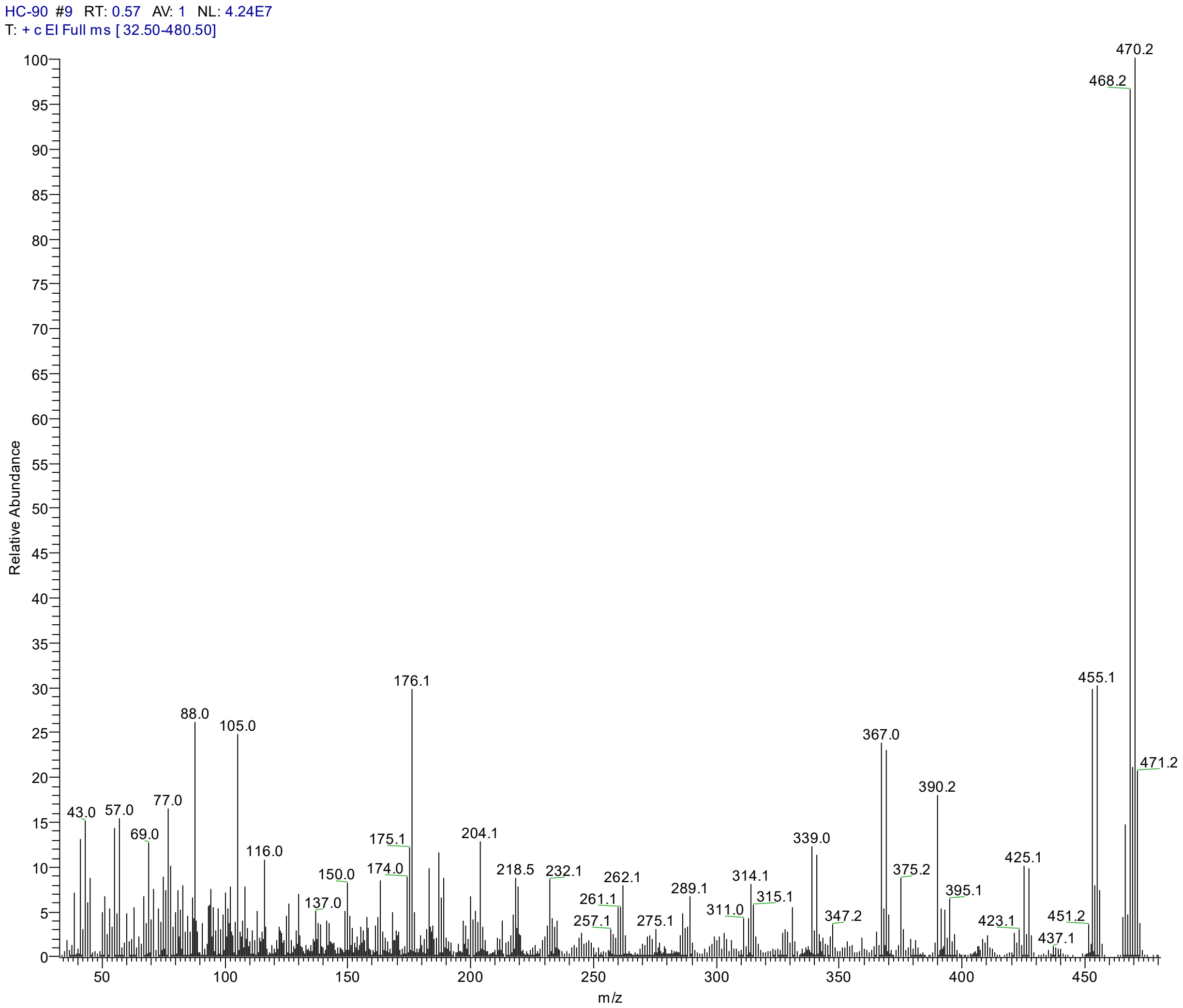

1-Hydroxy-4-phenyl-2-(3,4,5-trimethoxyphenyl)anthracene-9,10-dione (43)

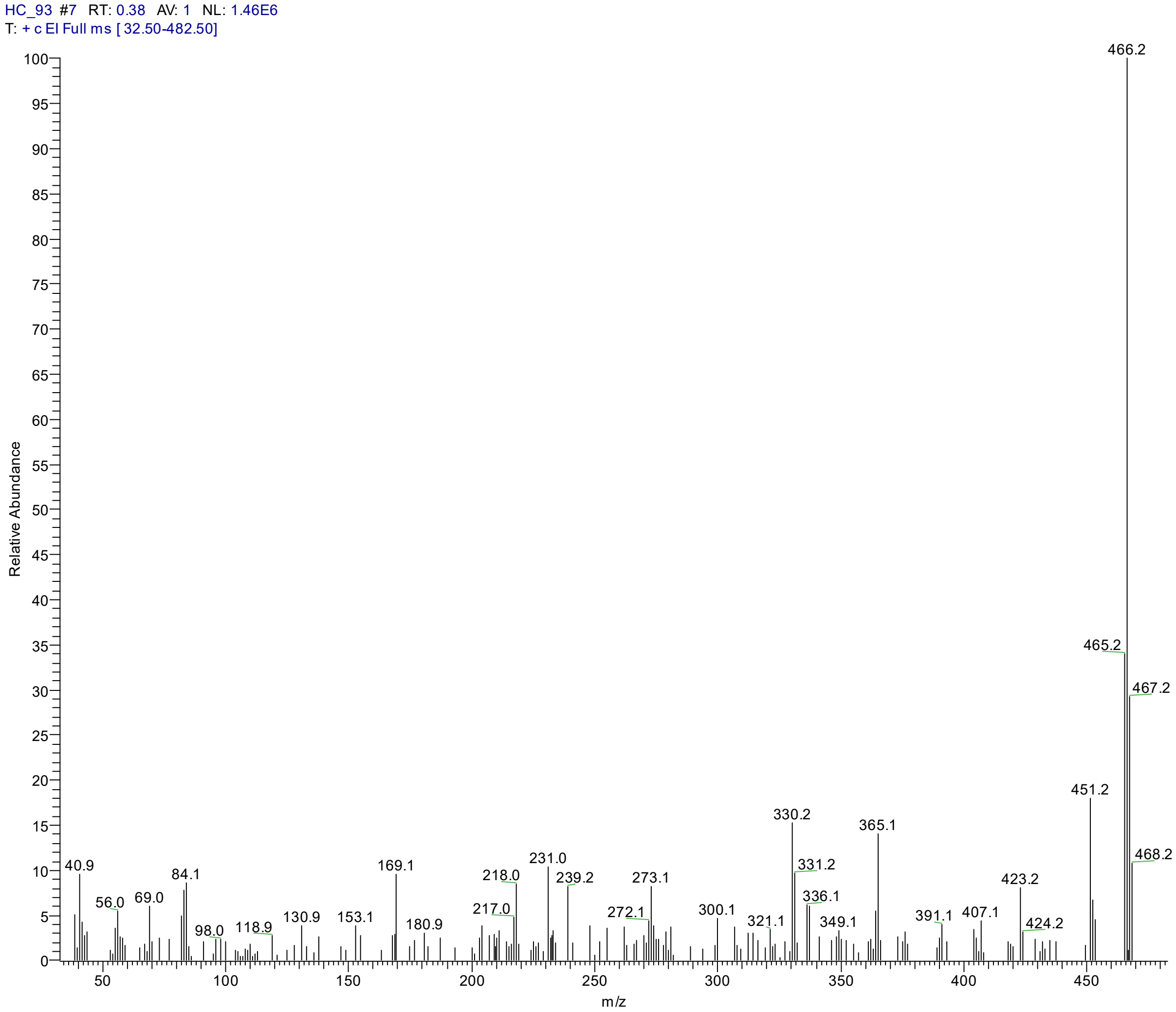

1-Hydroxy-4-(4-methoxyphenyl)-2-(3,4,5-trimethoxyphenyl)anthracene-9,10-dione (44)

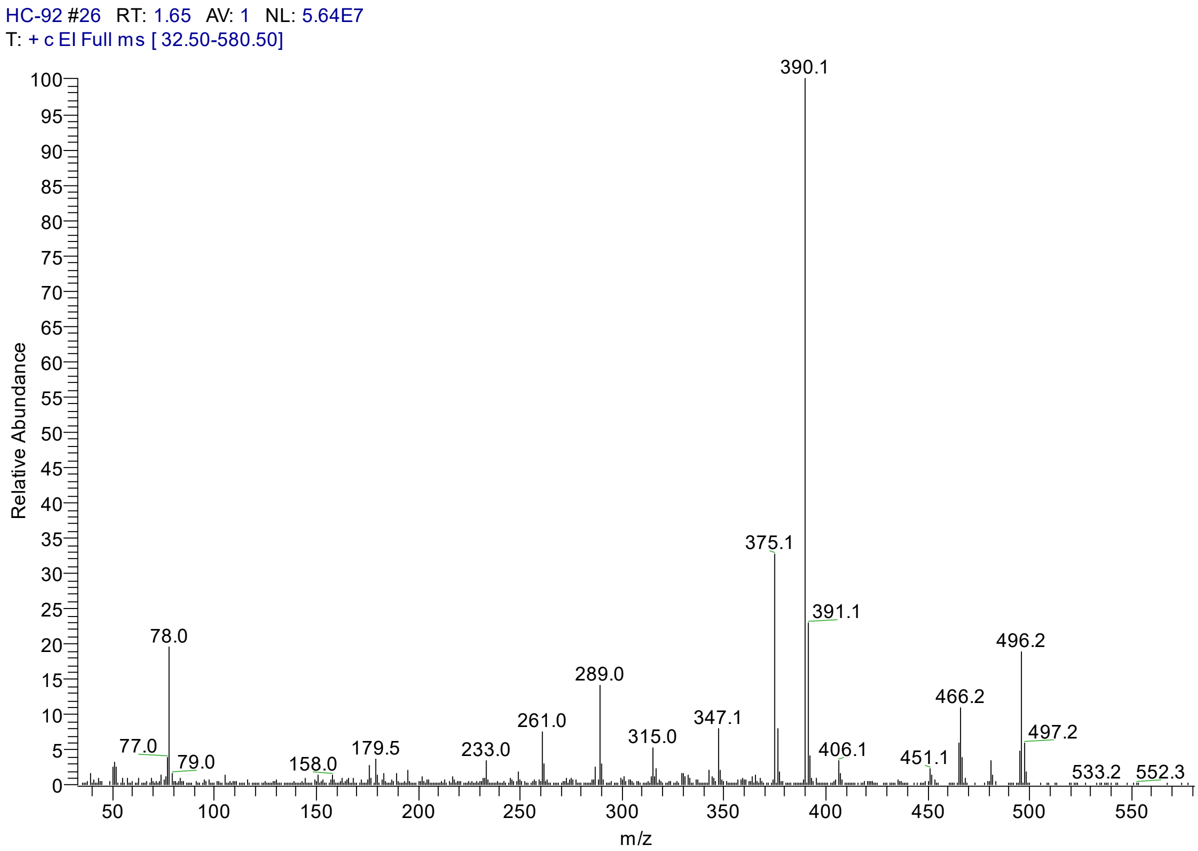

4-(4-Chloro-2-(trifluoromethyl)phenyl)-1-hydroxy-2-(3,4,5-trimethoxyphenyl)anthracene-9,10-dione (45)

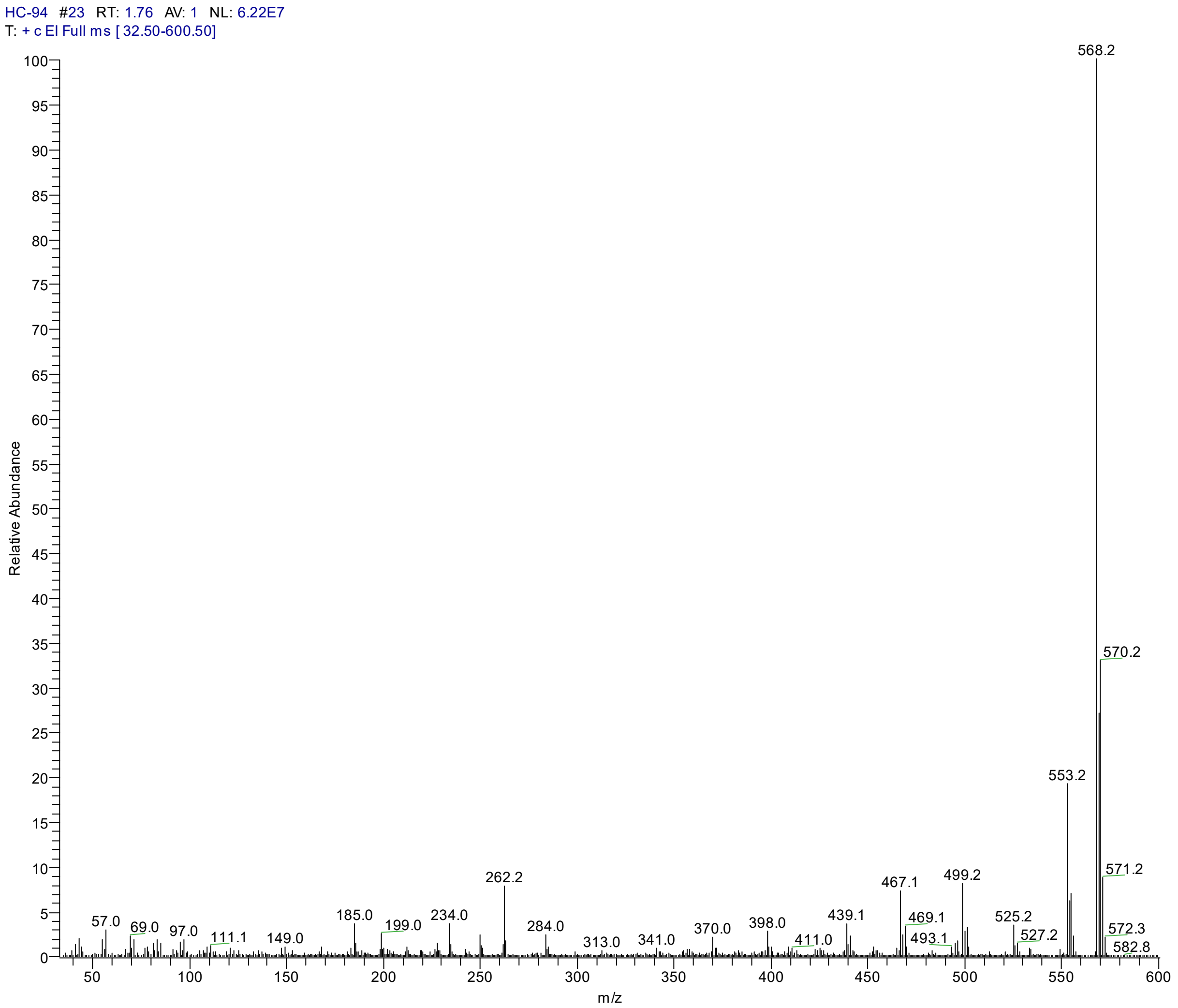

IR spectra of selected compounds

1-Hydroxy-4-(3,4,5-trimethoxyphenyl)anthracene-9,10-dione (5)

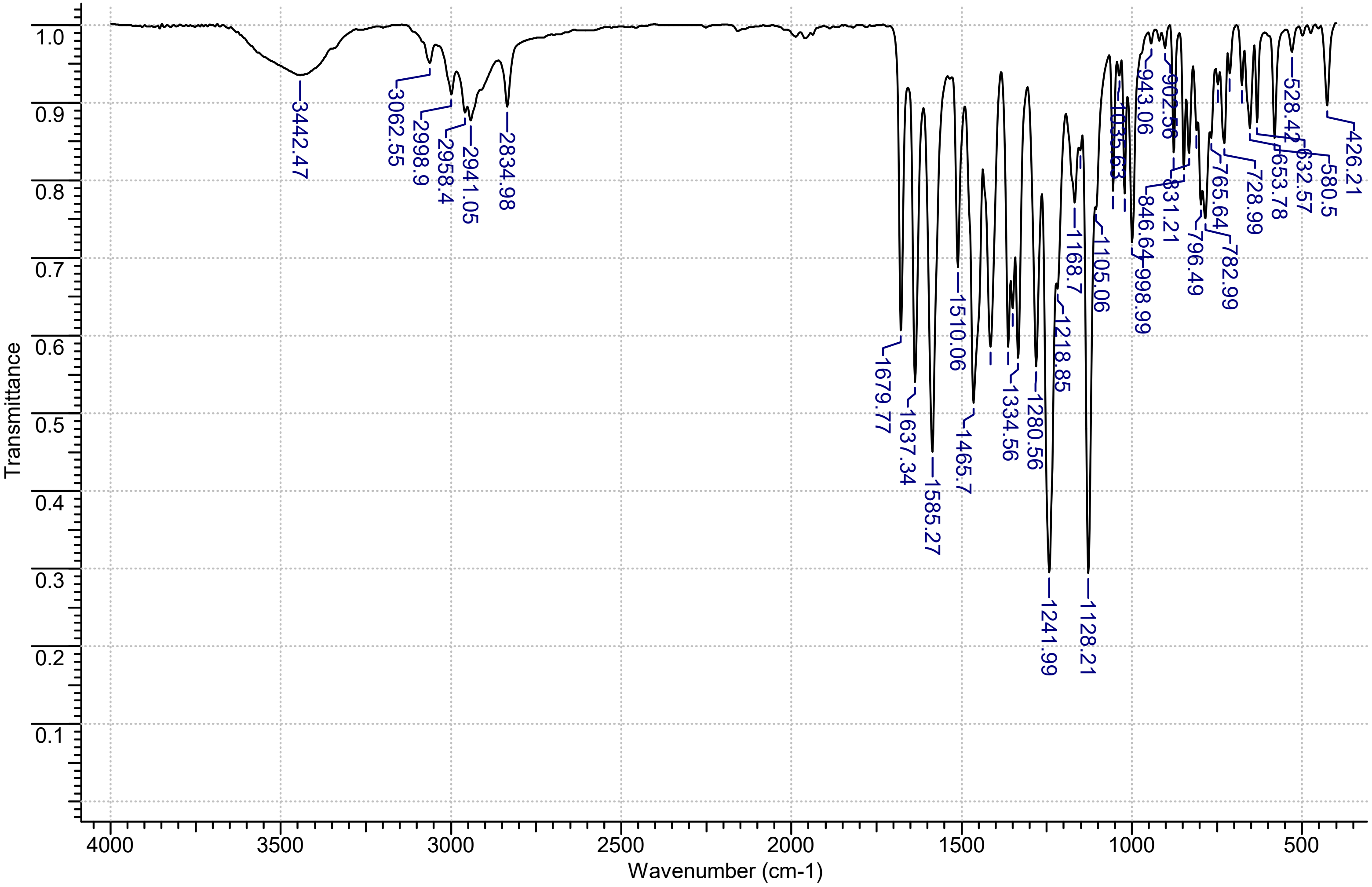

1-(2,3-Dimethoxyphenyl)-4-hydroxyanthracene-9,10-dione (16)

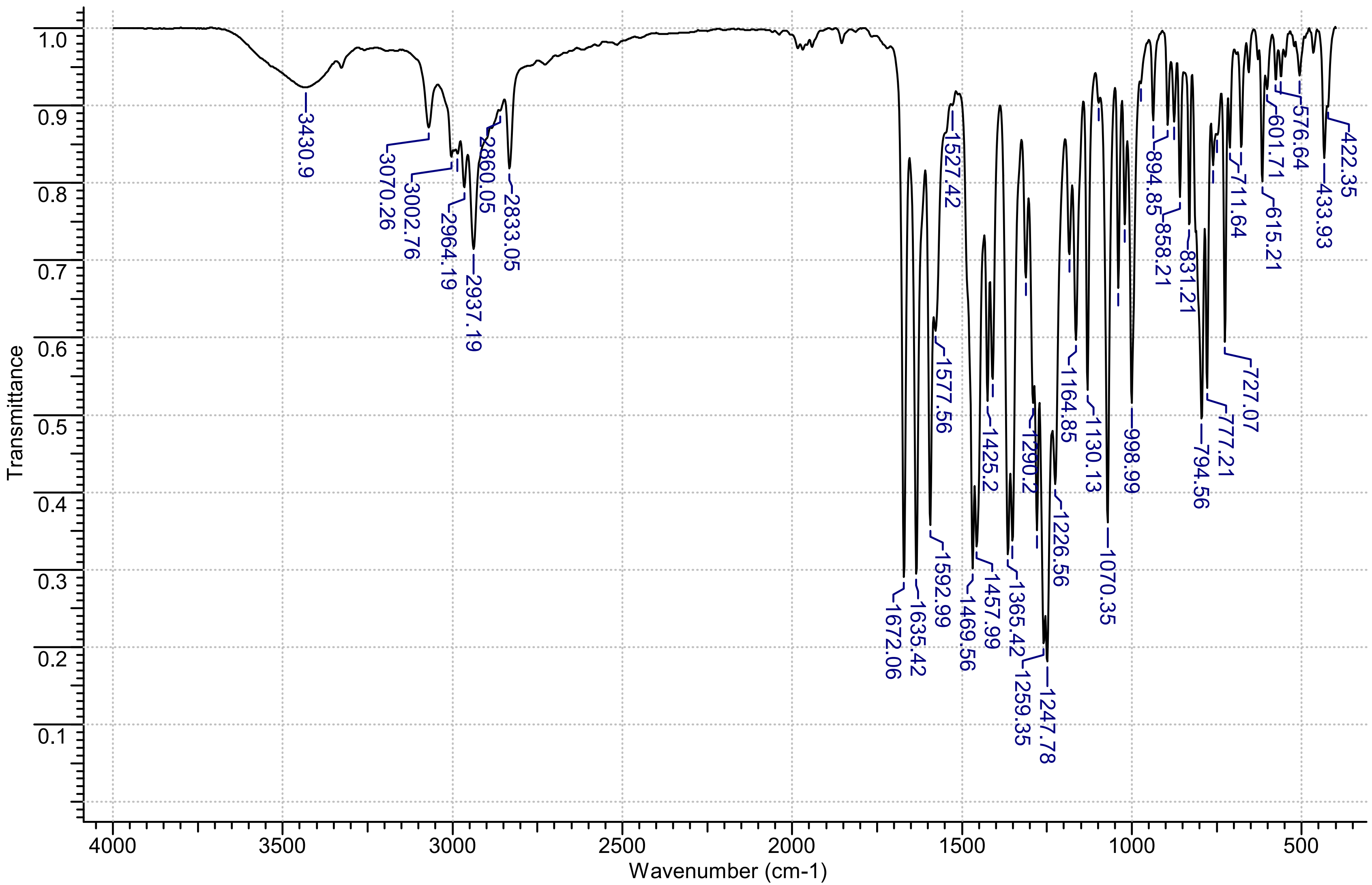

2-(3,5-Difluorophenyl)-1-hydroxyanthracene-9,10-dione (29)

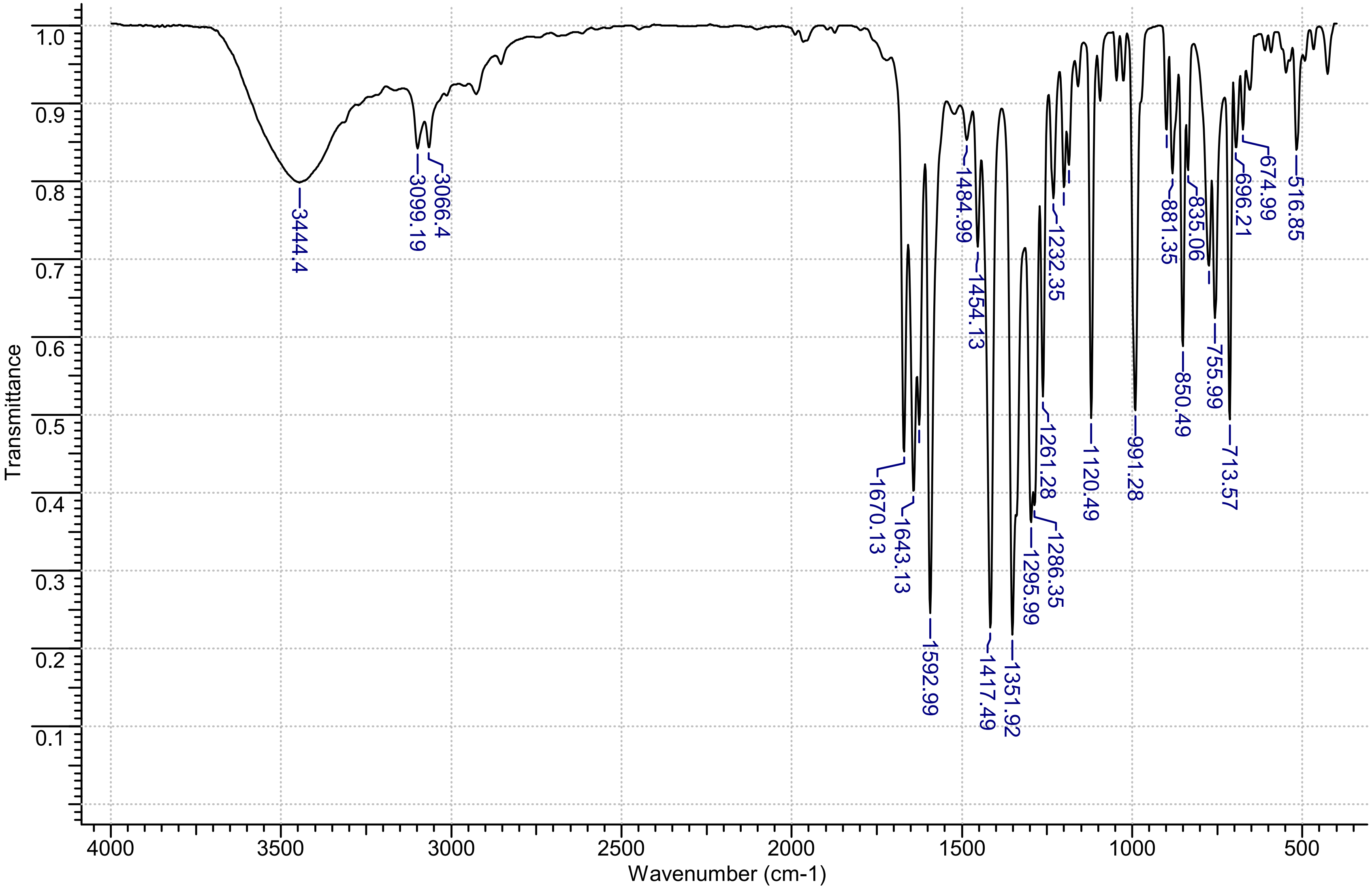

2-(2-Chloro-5-(trifluoromethyl)phenyl)-1-hydroxyanthracene-9,10-dione (30)

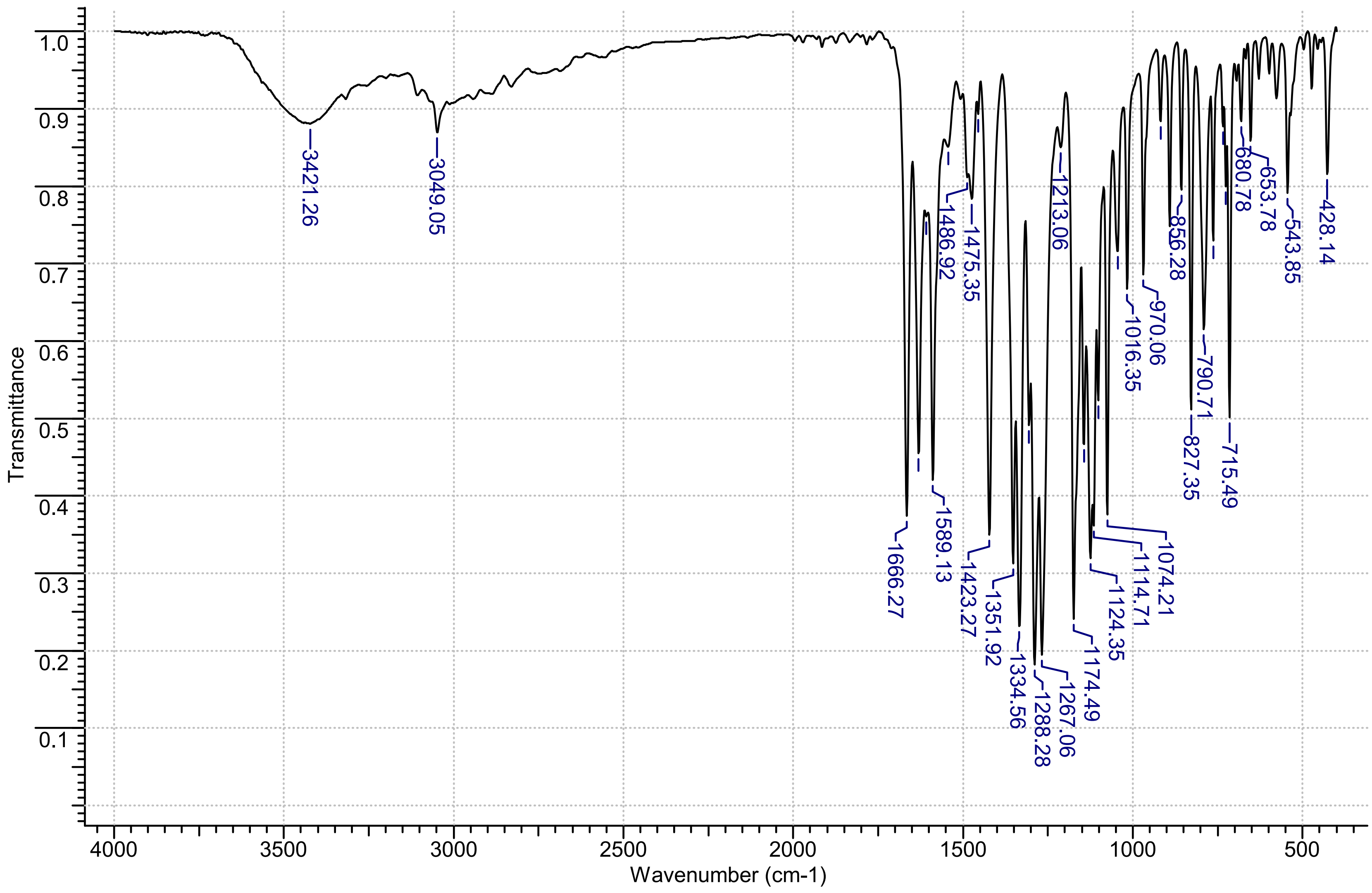

2,4-Di-(3,5-difluorophenyl)-1-hydroxyanthracene-9,10-dione (37)

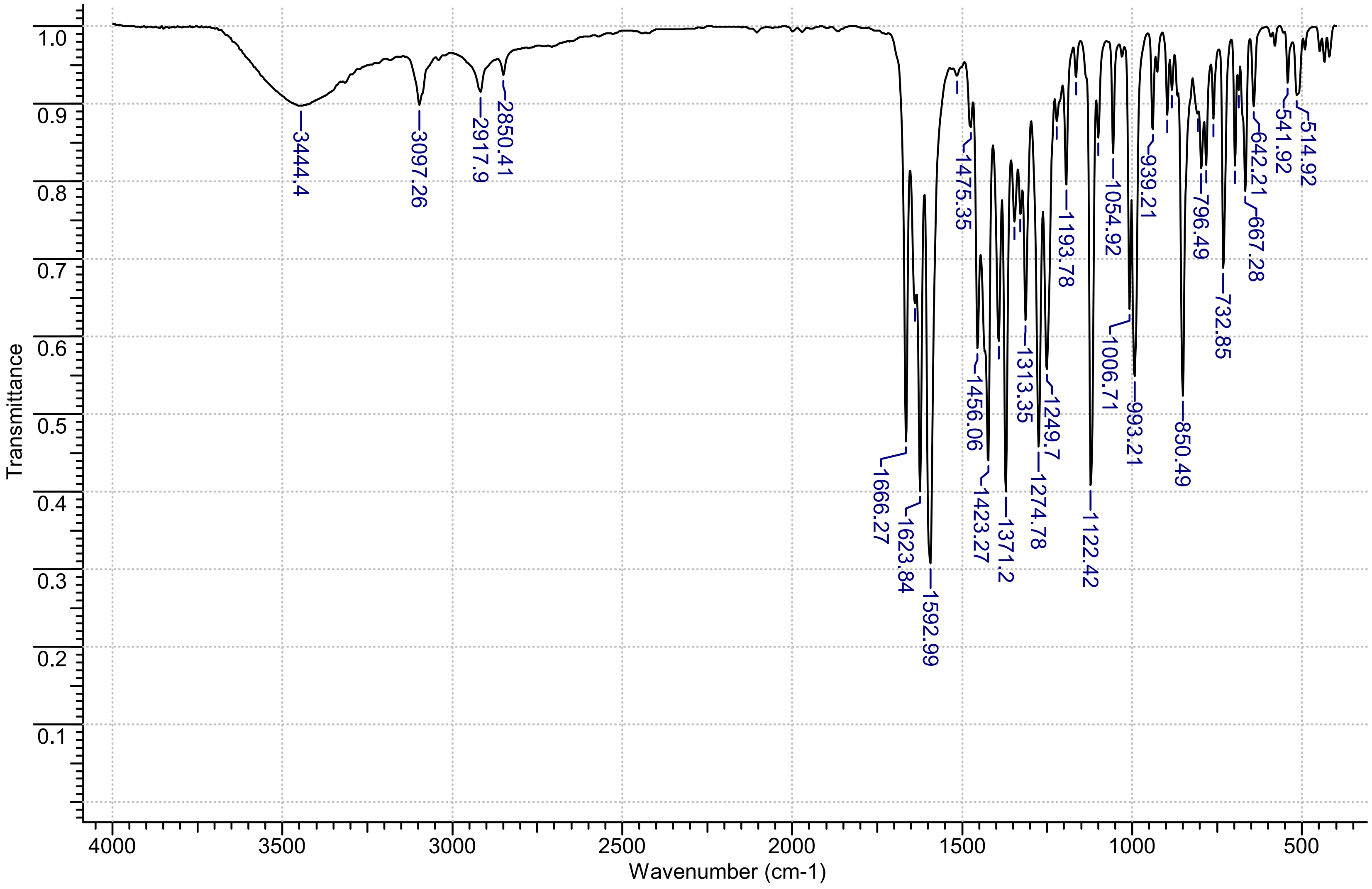

UV spectra of selected compounds

1-(Furan-2-yl)-4-hydroxyanthracene-9,10-dione (22)

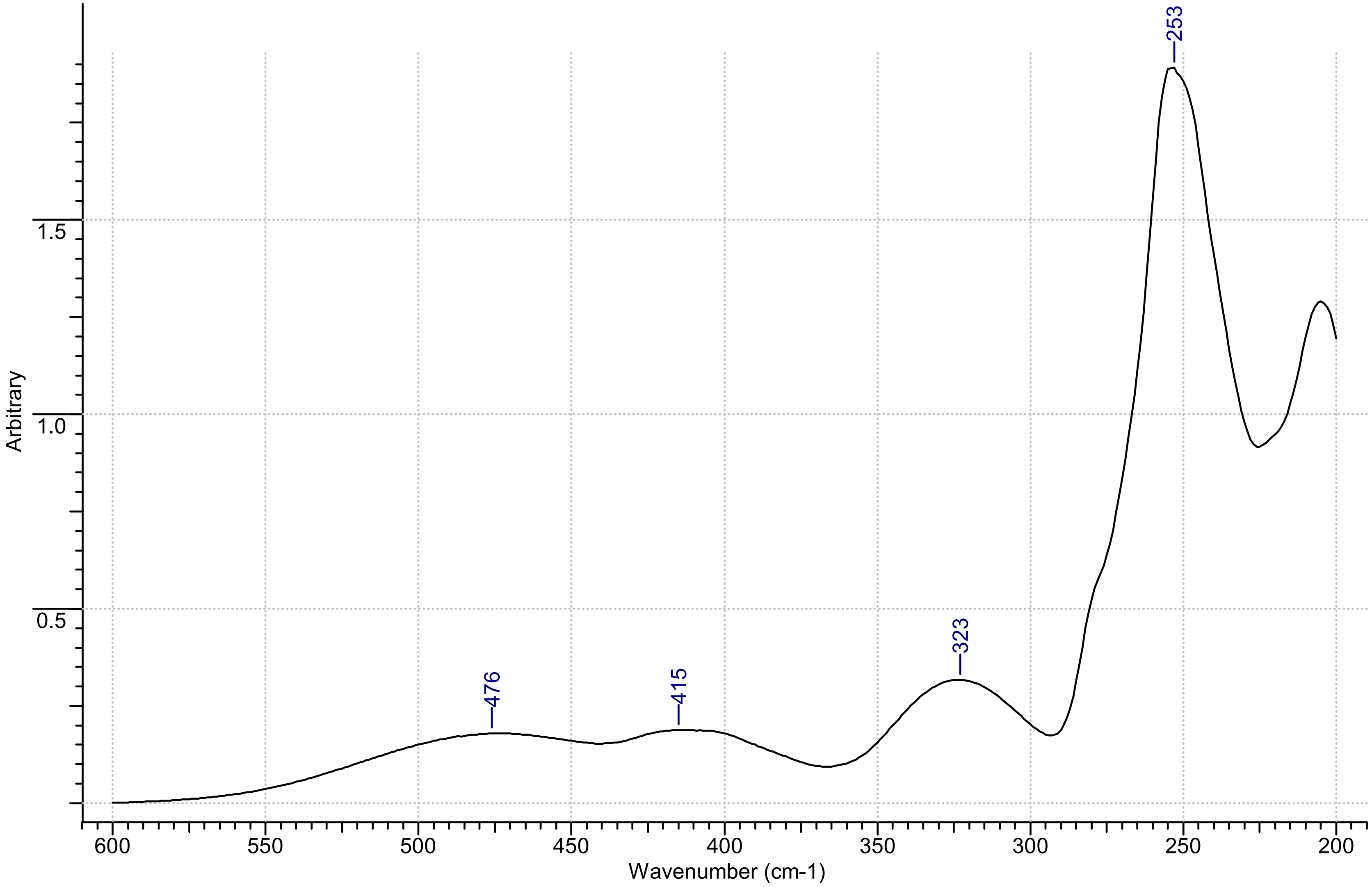

1-Hydroxy-2-phenylanthracene-9,10-dione (25)

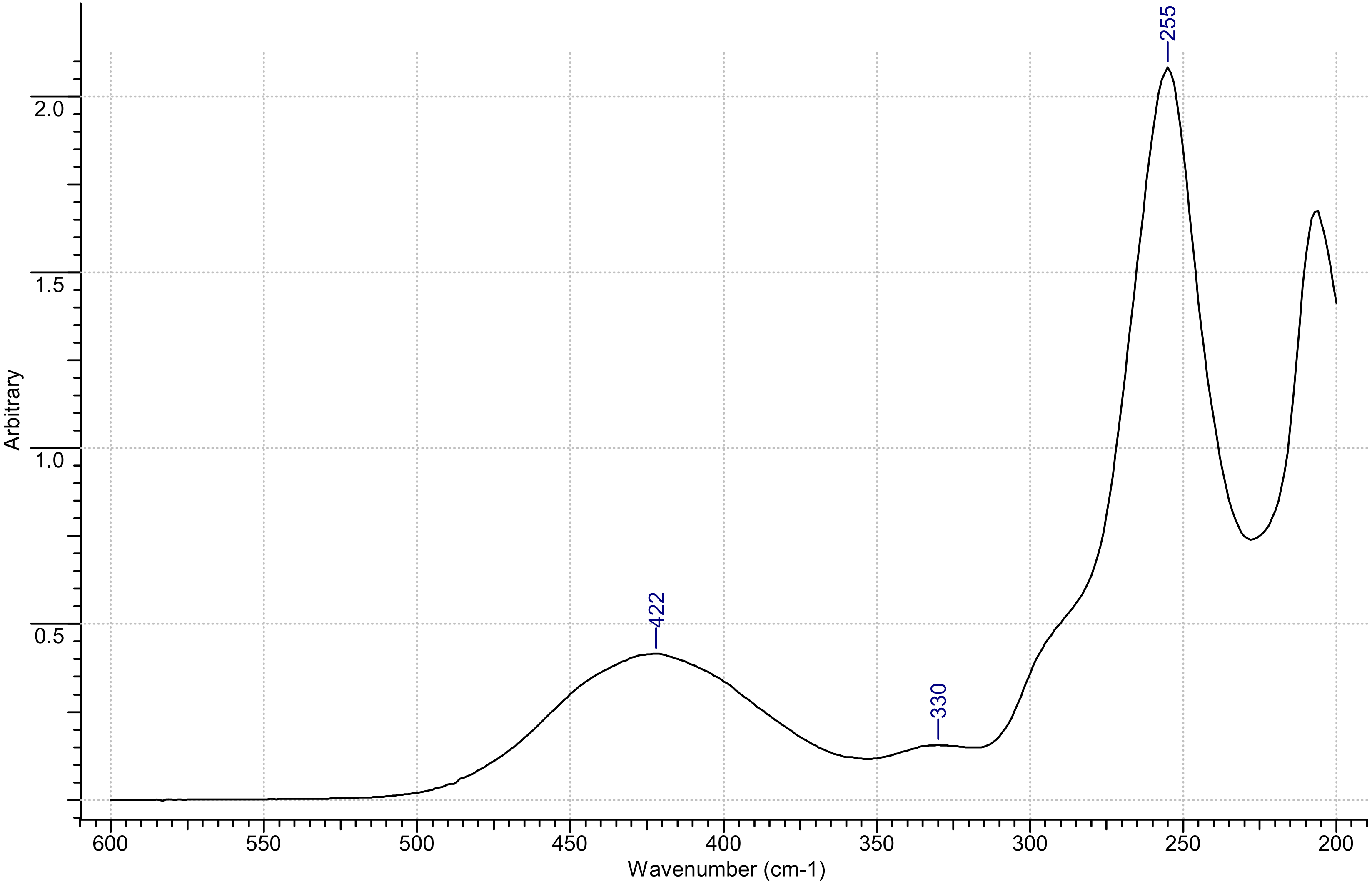

2-(3,5-Difluorophenyl)-1-hydroxyanthracene-9,10-dione (29)

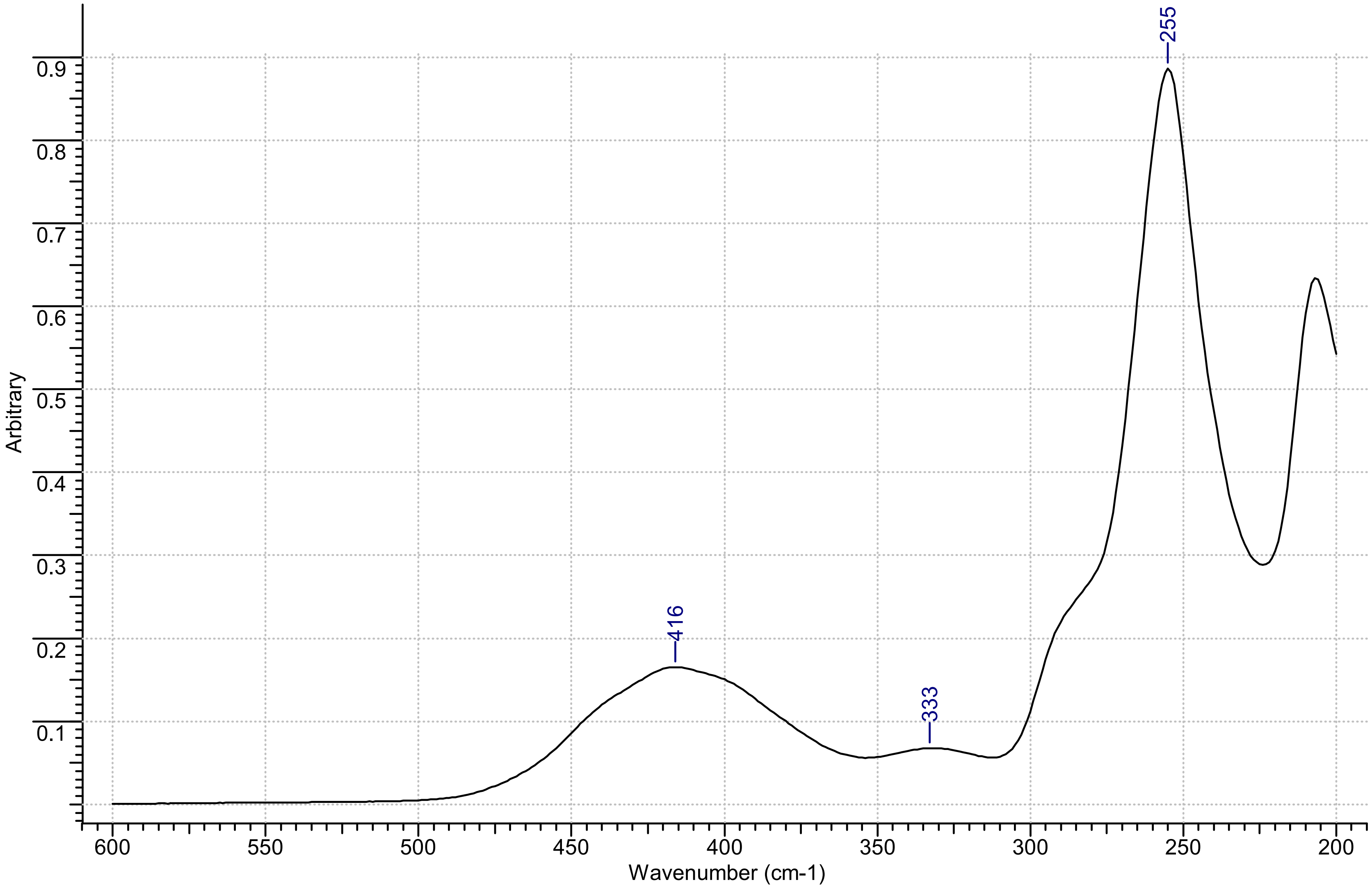

1-Hydroxy-2,4-di-(*o*-tolyl)anthracene-9,10-dione (34)

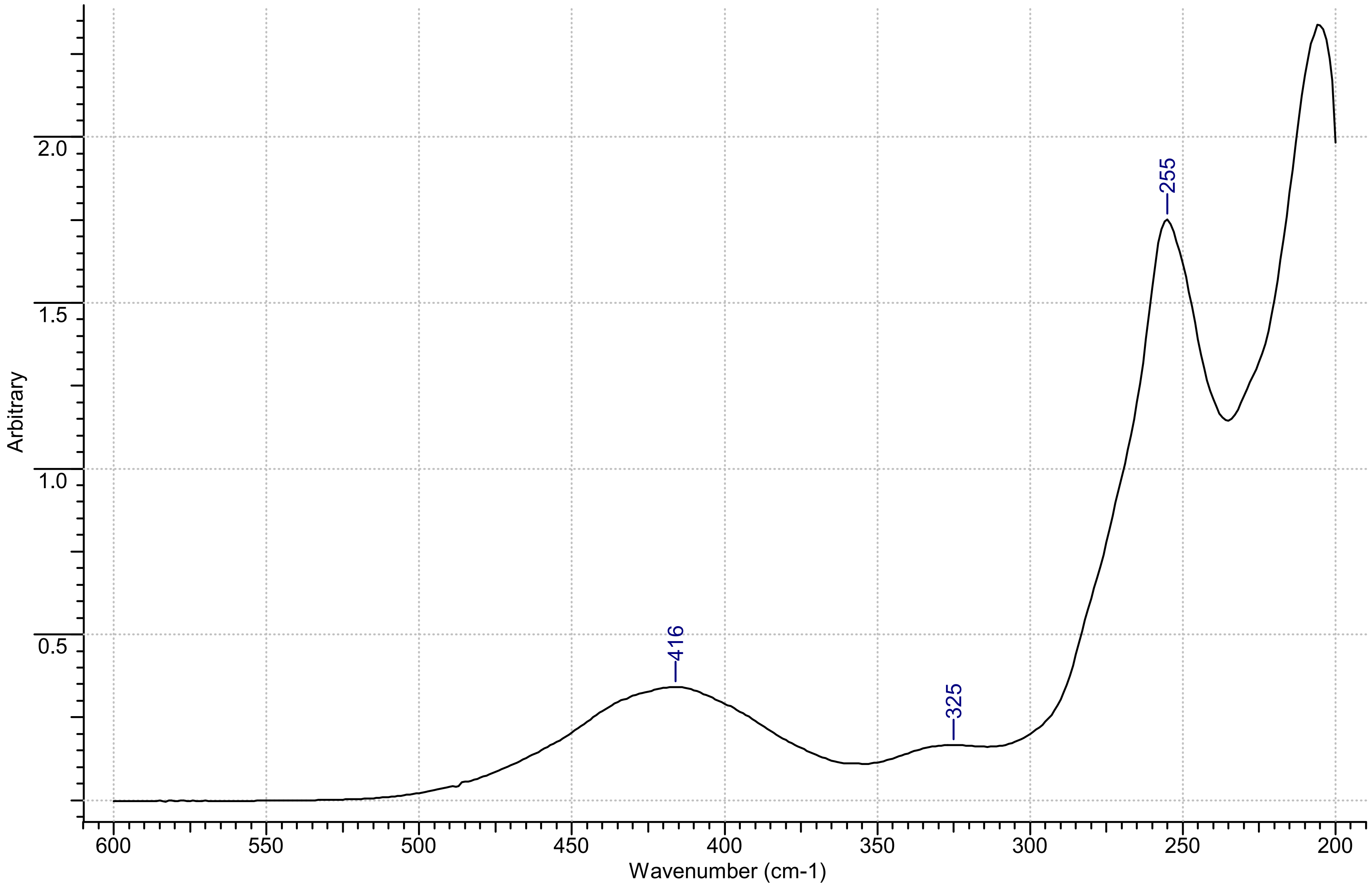

1-Hydroxy-4-phenyl-2-(3,4,5-trimethoxyphenyl)anthracene-9,10-dione (43)

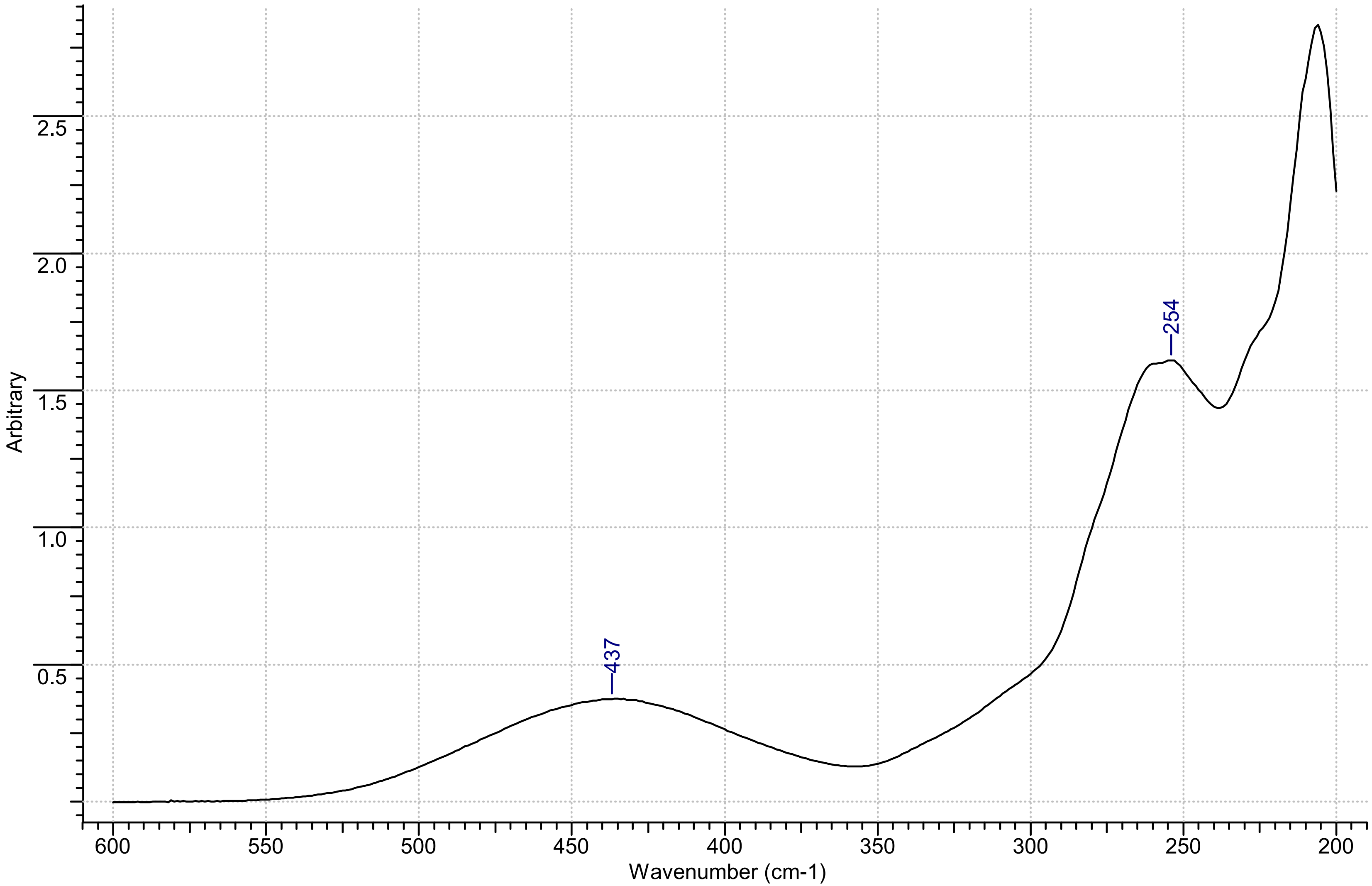

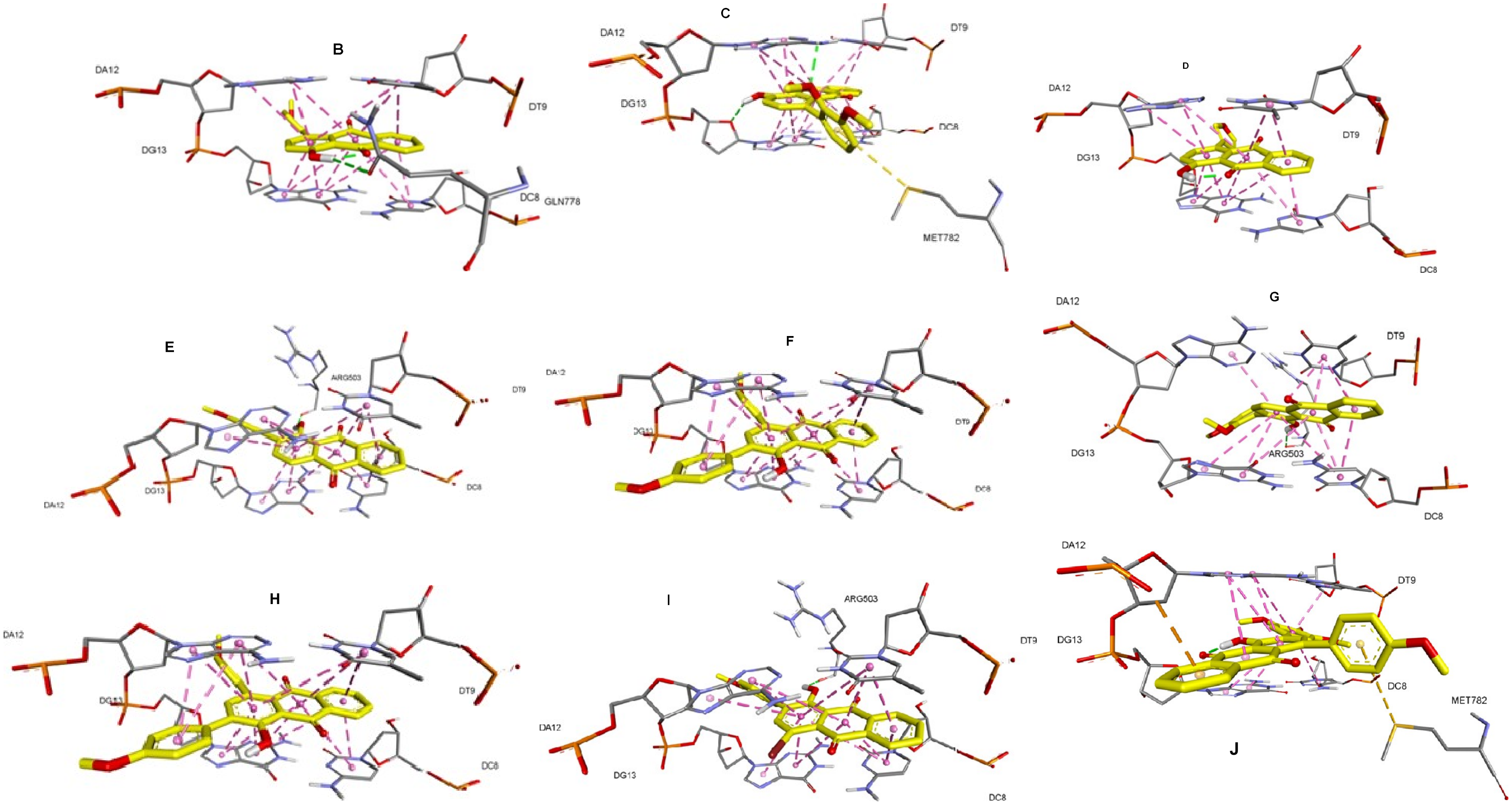

Figure 2S. Docking of new derivatives **15**, **16**, **23**, **25**, **27**, **28**, **35, 40** and **44** in topoisomerase IIβ-DNA complex binding site. B – **15**, C – **16**, D **23**, E **25**, F **27**, G **28**, H – **35**, I – **40**, **a**nd J **44**. Non-covalent interactions of molecules are shown by dotted lines: green - hydrogen bonds, orange – electrostatic interactions, yellow – sulphur interactions, purple – stacking interactions.
